# Iron-Catalyzed Synthesis of α-Azido α-Amino Esters via the Alkylazidation of Alkenes

**DOI:** 10.1021/acs.orglett.3c02153

**Published:** 2023-09-08

**Authors:** Pierre Palamini, Emmanuelle M. D. Allouche, Jerome Waser

**Affiliations:** Laboratory of Catalysis and Organic Synthesis, Institut des Sciences et Ingénierie Chimique, Ecole Polytechnique Fédérale de Lausanne, CH-1015 Lausanne, Switzerland

## Abstract

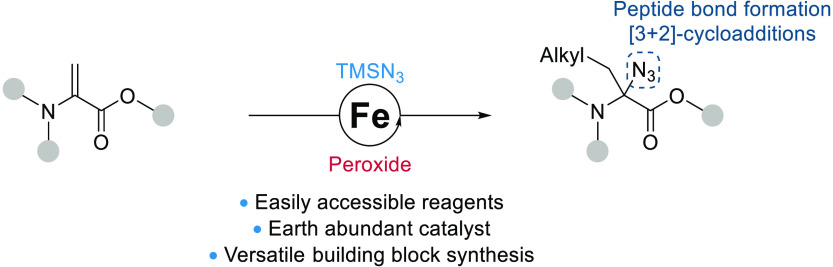

An iron-catalyzed alkylazidation of dehydroamino acids using peroxides as alkyl radical precursors is described. Non-natural azidated amino esters bearing an α-alkyl chain could be obtained in 18–94% yields using TMSN_3_ as an azide source. The obtained α-alkyl-α-azide α-amino esters could be further functionalized through cycloaddition or azide reduction with amide couplings to afford aminal-type peptides, α-triazolo amino acids, and tetrahydro-triazolopyridine, showing the great versatility of this now easily accessible class of amino acids.

Structurally diverse non-natural amino acids and peptides are of great interest for the pharmaceutical industry.^1^ As a consequence, the synthesis of nonproteinogenic α-alkyl α-amino acids has been widely studied since the beginning of modern synthetic chemistry.^2^ Quaternary amino acids bearing two carbon substituents at the α position have also attracted interest due to their effect on the stability and conformation of peptides.^3^ This has led to their incorporation in APIs such as decernotinib^4^ or carbidopa.^5^ In contrast, amino acids bearing an extra heteroatom at the α position have been investigated less frequently (Scheme 1a). Derivatives with substituents such as alkoxy,^6^ sulfone,^7^ or halogen^8^ groups have been mainly used as glycine α-imino ester surrogates for the synthesis of more complex nonproteinogenic amino acids,^9^ although examples for the elaboration of more complex peptides^10^ or use as bioactive compounds are also reported.^11^ α-Nitrogen-amino acids have also been described, mainly as α-amino-glycines.^12^ Interestingly, the stability of α-amino-glycines can be controlled by the substituent on nitrogen, which has led to their use as pro-drugs.^13^ An α-amino glycine can also be found in the core of bioactive benzodiazepine derivatives.^14^ In contrast, α-alkylamino-substituted derivatives, although they have been studied since the 1940s, have been investigated much less frequently.^15^ Nevertheless, potential pharmaceutical applications have been reported in the patent literature.^16^ The scarcity of reports about these most substituted derivatives can be tentatively attributed to the lack of synthetic methods for accessing them efficiently.

**Scheme 1:**
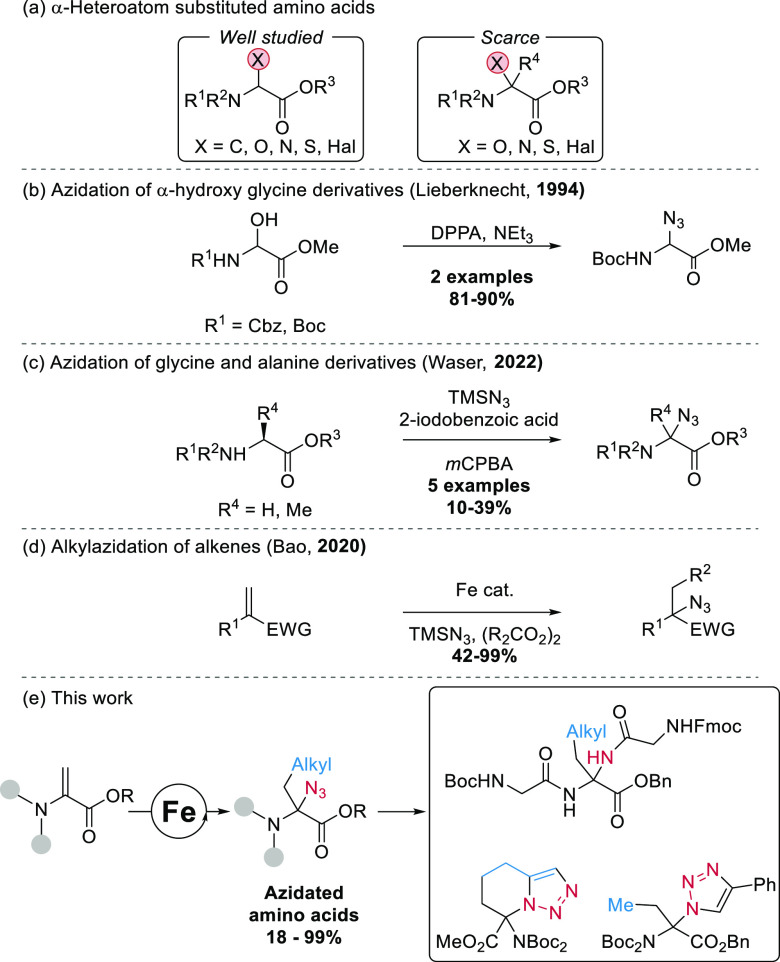
(a) α-Heteroatom-Substituted Amino Acids, (b) Azidation of α-Hydroxy Glycines, (c) Azidation of Glycine and Alanine Derivatives, (d) Alkylazidation of Electron-Deficient Alkenes, and (e) Carboazidation of Dehydroamino Acids on the Way to New Peptide Building Blocks

Among nitrogen-based functional groups, azides are important intermediates in the synthesis of natural products and pharmaceuticals and have found broad applications in chemical biology.^17^ They can be transformed into amines, amides, triazoles, imidazoles, and other functionalities.^18^ Only a few groups reported the synthesis of α-azide-substituted α-amino acids. Lieberknecht reported in 1994 the synthesis of α-azido glycines from α-hydroxy glycines (Scheme 1b).^19^ Our group reported in 2022 the azidation of glycine and alanine derivatives in moderate yields using TMSN_3_, 2-iodobenzoic acid, and *m*CPBA (Scheme 1c).^20^ α-Azido glycines could be latter transformed into dipeptides bearing an N-terminal amino glycine unit or reacted with nucleophiles to afford monosubstituted amino acids.^12^

Our strategy for the synthesis of C,N-disubstituted amino acids relies on the use of dehydroamino acids. Monofunctionalization of dehydroamino acids (Dhas) through conjugated addition has been achieved through radical addition,^21^ although only a few examples of difunctionalization such as alkoxybromination,^22^ halofluorination,^23^ carbofluorination,^24^ and germylperoxidation^25^ have been described.^26^ In addition, arylazidation has been reported on a single substrate.^27^ Inspired by works of Liu,^28^ Luo,^29^ and Bao^30^ on the carboazidation of alkenes using an iron catalyst and peroxides (Scheme 1d), we wondered if this transformation could be achieved on dehydroalanines to give general access to more substituted α-azido-α-amino acids.

Herein, we report the successful implementation of this strategy (Scheme 1e). Using cheap nontoxic iron salts as catalysts, key building blocks for the construction of unprecedented aminal-type peptides, [7,7]-substituted tetrahydro-triazolopyridine and α-alkyl-α-triazole α-amino acids could be accessed in high yields. Aminal-type peptides are important compounds in medicinal chemistry, but in most derivatives, there is only a single alkyl group on the aminal carbon.^31^ Therefore, our work allows the significant expansion of the chemical space of this class of compounds.

We started our investigation using *N*Boc_2_-Dha-OMe **1a**(32) as a model substrate and commercially available lauroyl peroxide (LPO) **2a** as a radical source. Reaction in DME at 38 °C for 2 h in the presence of 5 mol % Fe(OTf)_2_ afforded **3a** in 74% yield (Table 1, entry 1). Decreasing the temperature to room temperature (21 °C) led to an increase in the yield to 94% (entry 2). When using *tert*-butyl peroxybenzoate (TBPB, **2b**) as a methyl radical source, the temperature had to be decreased to 0 °C to obtain **3b** in 92% yield (entries 3 and 4). Various solvents were well tolerated (entries 5–8 and Table S1). In particular, 2-MeTHF (entries 5 and 9) and EtOAc (entry 6) as greener solvents gave comparable yields with lauroyl peroxide, although the yield was lower for methylation. Other sources of iron were examined, affording lower yields (entries 10–12).

**Table 1:**
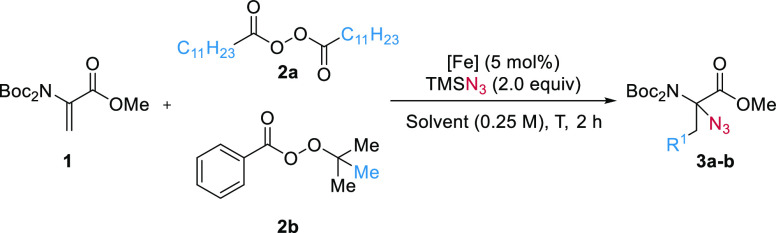
Optimization of the Alkylazidation Reaction^a^

entry	R^1^	product	[Fe]	solvent	*T* (°C)	yield (%)^b^
1	C_11_H_23_	**3a**	Fe(OTf)_2_	DME	38	74
2	C_11_H_23_	**3a**	Fe(OTf)_2_	DME	21	94 (94)^c^
3	CH_3_	**3b**	Fe(OTf)_2_	DME	21	79
4	CH_3_	**3b**	Fe(OTf)_2_	DME	0	92 (92)^c^
5	C_11_H_23_	**3a**	Fe(OTf)_2_	2-MeTHF	21	94
6	C_11_H_23_	**3a**	Fe(OTf)_2_	EtOAc	21	89
7	C_11_H_23_	**3a**	Fe(OTf)_2_	DCM	21	85
8	C_11_H_23_	**3a**	Fe(OTf)_2_	acetone	21	81
9	CH_3_	**3b**	Fe(OTf)_2_	2-MeTHF	0	76
10	C_11_H_23_	**3a**	Fe(OAc)_2_	DME	21	20
11	C_11_H_23_	**3b**	FeCl_2_	DME	21	79
12	C_11_H_23_	**3a**	FeCl_3_	DME	21	37

a Reaction conditions: Dha **1a** (1.0 equiv), TMSN_3_ (2.0 equiv), and lauroyl peroxide (LPO) or *tert*-butyl peroxybenzoate (TBPB) (2.0 equiv) under a N_2_ atmosphere on a 0.25 mmol scale.

b NMR yield determined using mesitylene as the internal standard.

c Isolated yield.

With the optimal conditions in hand, we explored the scope of amino and ester groups using LPO **2a**, TBPB **2b**, and isobutyryl peroxide (**2c**) as alkyl radical precursors for the transfer of undecyl, methyl and isopropyl radicals, respectively (Scheme 2). *N*Boc_2_-Dha **1a** afforded **3a**–**3c** in 94%, 92%, and 94% yields, respectively, whereas *N*HBoc-Dha **1b** led to an 81% yield for the introduction of the C_11_ chain and a 76% yield for methylation. Azide **3c** could be obtained in 81% yield on a 5 mmol scale. *N*HFmoc-Dha **1c** afforded **3f** in 87% yield, and *N*HAc-Dha **1d** led to 69%, 52%, and 45% yields of **3g**–**3i**, respectively. Different ester groups were tolerated. *N*Boc_2_-Dha-O*tBu***1e** afforded **3j** in 81% yield and **3k** in 63% yield. *N*Boc_2_-Dha-OBn **1g** afforded **3l** in 79% yield, azidated homoalanine **3m** in 86% yield, and azidated leucine **3n** in 65% yield. Finally, *N*HBoc-Dha-OBn **7** afforded azidated homoalanine **3o** in 79% yield.

**Scheme 2:**
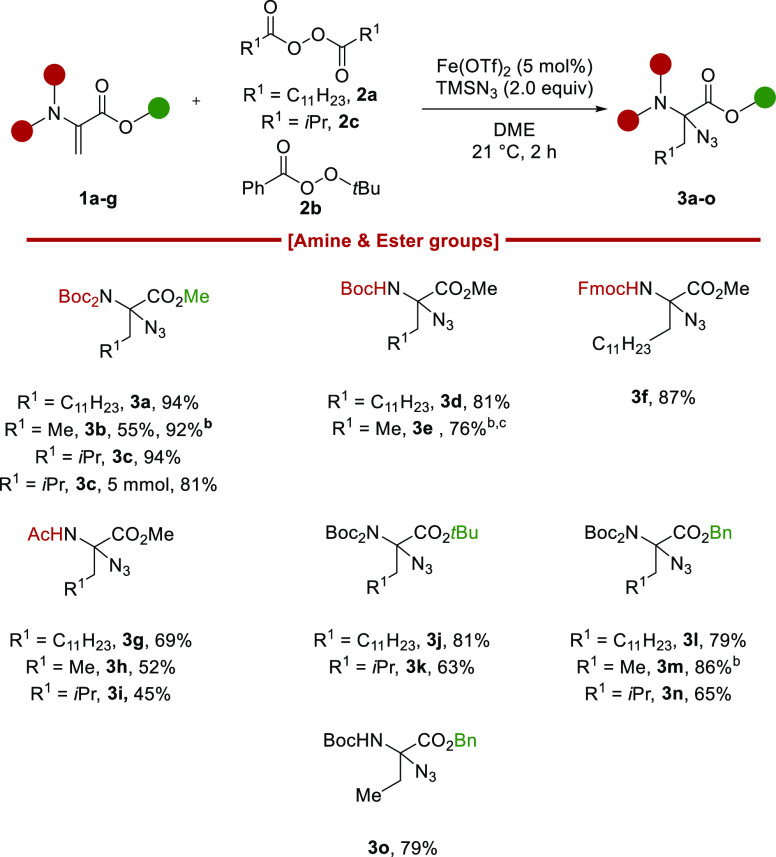
Scope of Amino and Ester Groups Reaction conditions: Dha **1** (1.0 equiv), TMSN_3_ (2.0 equiv), peroxide **2** (2.0 equiv), and DME (0.25 M) at 21 °C for 2 h. Reactions were carried out under a N_2_ atmosphere on a 0.25 mmol scale. At 0 °C. With 1.5 mmol.

Having established the compatibility of the reaction with various ester and amine groups, we next explored the different alkyl groups that could be introduced into dehydroalanine **1a**. Two types of easily accessible peroxides could be used as alkyl precursors: symmetric diacyl peroxides of type A and *tert*-butyl peroxide esters of type B (Scheme 3).^33^ Using the *tert*-butyl peroxide ester of acetic acid (**2d**), **3b** could be obtained in 55% yield, while using TBPB **2b**, a 92% yield was obtained. Nonproteinogenic novarline **3p** could be isolated in 95% yield. A tertiary radical and a neopentyl radical were successfully introduced, affording α-neopentylglycine **3q** in 50% yield and **3r** in 86% yield. For **3q**, no product was obtained with a diacyl peroxide of type A.

**Scheme 3:**
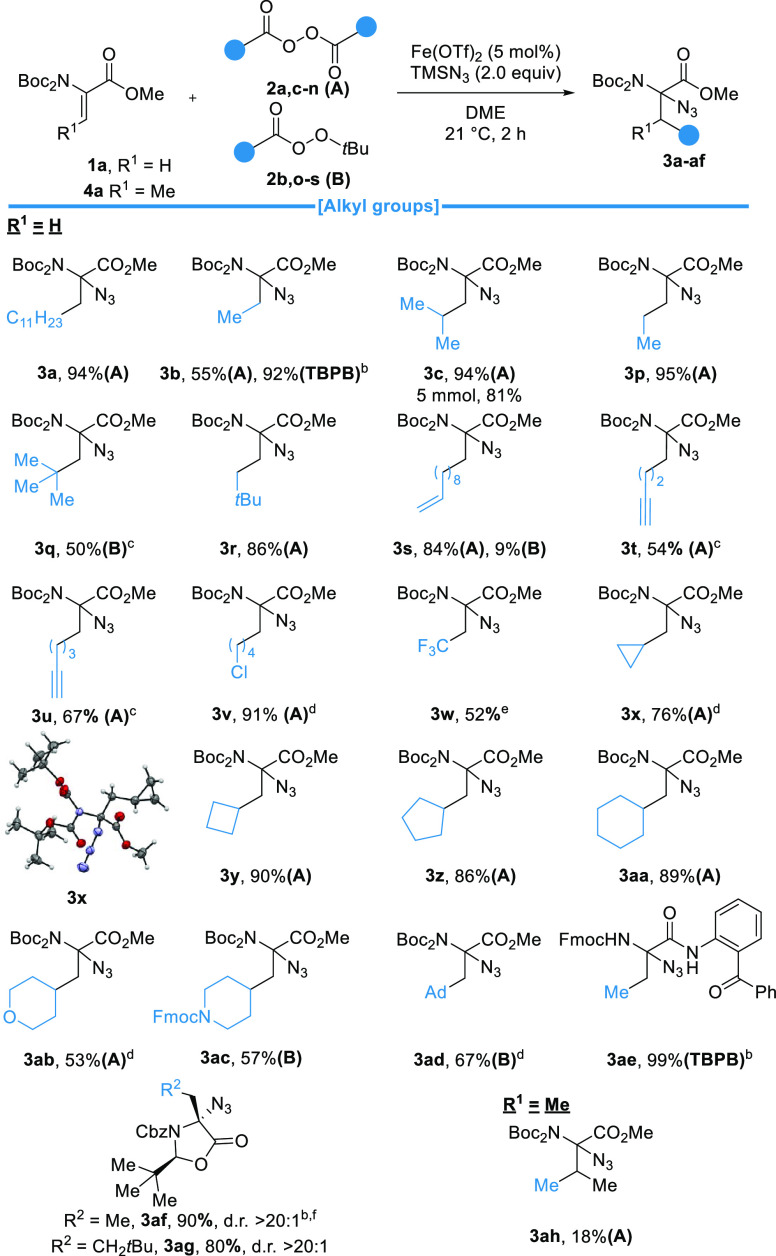
Scope of Alkyl Groups Reaction conditions: Dha **1** (1.0 equiv), TMSN_3_ (2.0 equiv), peroxide **2** (2.0 equiv), and DME (0.25 M) at 21 °C for 2 h. Reactions were carried out under a N_2_ atmosphere on a 0.25 mmol scale. At 0 °C. For 6 h. For 4 h. At 40 °C using Togni II reagent. With 7 mmol.

The introduction of an unsaturated bond was also possible. **3s** was isolated in 84% yield using a type A peroxide, while only a low level of product formation was observed using a type B peroxide. An alkyne was also compatible as **3t** and **3u** were obtained in 54% and 67% yields, respectively. A chlorinated alkyl chain could be introduced, affording **3v** in 91% yield. A trifluoromethyl radical could also be added using Togni II reagent to afford **3w** in 52% yield.^34^ Cycloalkanes were also successfully introduced. Cyclopropane **3x**, cyclobutane **3y**, cyclopentane **3z**, and cyclohexane **3aa** were obtained in 76%, 90%, 86%, and 89% yields, respectively. Heterocycles such as tetrahydropyran **3ab** and Fmoc-protected piperidine **3ac** could be produced in 53% and 57% yields, respectively. An adamantyl radical could also be added, affording **3ad** in 67% yield. More complex amide **3ae** could also be obtained in quantitative yield. Using Karady–Beckwith chiral Dha **1j**,^35^**3af** and **3ag** could be obtained in good yields as a single diastereoisomer. Furthermore, starting from dehydrobutyrine, azidated valine **3ah** could be obtained in a moderate yield of 18%. Interestingly, through analysis of the N–O distances of the X-ray structures of **3x** and **3ad**, we observed interactions between the azide and the OMe of the ester, as well as one of the two carbonyls of the carbamate (Scheme 3 and Figure S5).^36^

Having in hand diverse α-alkyl-α-azide α-amino acids, we explored their modification. Azidated homoalanines **3m** and **3o** could be transformed into triazoles **5a** and **5b**, respectively, through a copper-catalyzed alkyne–azide cycloaddition in 82–85% yields (Scheme 4a).

**Scheme 4:**
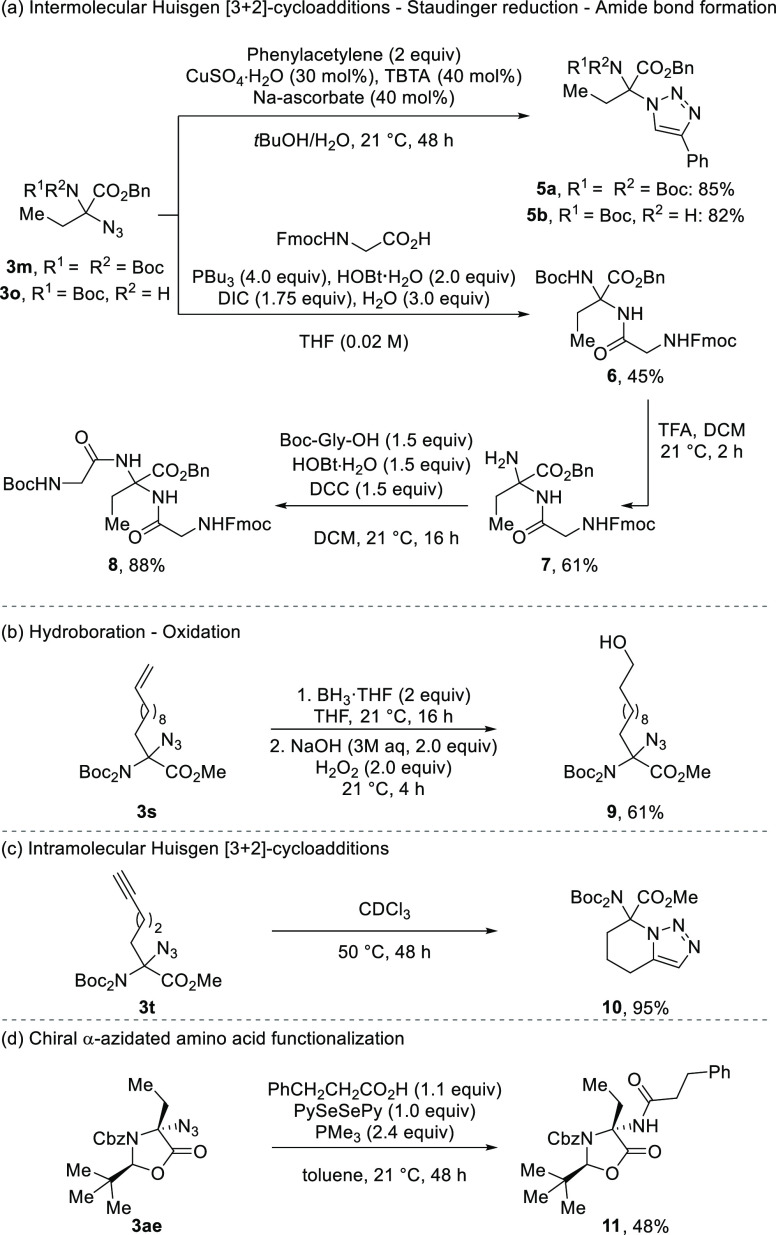
Product Modifications See the Supporting Information for experimental details.

One-pot Staudinger reduction–amide coupling with Fmoc-glycine afforded aminal **6** in 45% yield.^37^ Further deprotection allowed us to isolate amine **7** in 61% yield. The free amine was coupled with Boc-glycine to afford unprecedented aminal-type tripeptide **8** in 88% yield (Scheme 4a). We then investigated the stability of compounds **3o** and **8** under various conditions. Both were stable in CH_3_CN, CH_3_CN/H_2_O, DMSO, and DMSO/H_2_O over 24 or 48 h at different pH values (4, 7, and 9) (Figures S1 and S2). Modification of the introduced alkyl chain was also possible. A hydroboration–oxidation sequence of **3s** afforded **9** in 61% yield (Scheme 4b). Furthermore, intramolecular alkyne–azide cycloaddition of **3t** afforded 7,7-disubstituted 4,5,6,7-tetrahydro[1,2,3]triazolo[1,5-*a*]pyridine **10** in 95% yield (Scheme 4c). Finally, chiral **3ae** could be transformed into amide **11** in 48% yield through a Staudinger–Vilarrasa reaction (Scheme 4d).^38,39^

In conclusion, we developed conditions for accessing α-azido amino acids through iron catalysis using easily accessible peroxides as alkyl precursors. Various α-alkyl-α-azide α-amino esters were obtained in moderate to excellent yields. The products could be functionalized, leading to various unprecedented scaffolds such as α-triazole amino acids, a 7,7-disubstituted 4,5,6,7-tetrahydro[1,2,3]triazolo[1,5-*a*]pyridine, and aminal-type peptides. Our work therefore contributed to significantly expand the chemical space of accessible α-alkyl α-nitrogen-substituted amino acids and peptides.

## Data Availability

The data underlying this study are available in the published article and its Supporting Information. Raw NMR, MS, and IR data are available free of charge from zenodo.org (DOI: 10.5281/zenodo.8183015).

## Supporting Information Available

Experimental procedures, analytical data for all new compounds, and copies of NMR spectra (PDF)
