# Supplementary material for: Iron-Catalyzed Synthesis of α-Azido α-Amino Esters via the Alkylazidation of Alkenes
Source: Org Lett. 2023 Sep 8;25(37):6791–5. doi: 10.1021/acs.orglett.3c02153 (PMC10521020; doi:10.1021/acs.orglett.3c02153)

Iron-catalyzed synthesis of α-azido α-amino esters via the alkylazidation of alkenes

Pierre Palamini, Emmanuelle M. D. Allouche and Jerome Waser*

Laboratory of Catalysis and Organic Synthesis, Institute of Chemical Sciences and Engineering, Ecole Polytechnique Fédérale de Lausanne, EPFL SB ISIC LCSO, BCH 4306, 1015 Lausanne, Switzerland.

jerome.waser@epfl.ch

(97 pages)

Table of contents

1. General information 3
2. Starting materials preparation 4
   2.1 Synthesis of dehydroamino acids....4
   2.2 Synthesis of alkyl peroxides 11
   2.3 Synthesis of 2,2'-dipyridyldiselenide....18
3. Optimization of the alkylzidation of dehydroamino acids 19
   Procedure 19
4. Scope of the alkylazidation of dehydroamino acids....20
   4.1 General procedures....20
   4.2 Characterization data 20
5. Scale-up 34
6. Speculative Mechanism....35
7. Product modification 35
   5.1 Intermolecular Huisgen [3+2]-cycloadditions....35
   5.2 Staudinger – Amide coupling 36
   5.3 Hydroboration- oxidation....38
   5.4 Intramolecular Huisgen [3+2]-cycloadditions....39
   5.5 Chiral α-azidated amino acids functionalization....39
8. Stability of **3o** and **8**....41
   Stability of **3o** 41
   Stability of **8**....42
9. Crystal structures....43
   7.1 Crystal structure of **3x** 43
   7.2 Crystal structure of **3ad**....45
   9.3 Conformational locking analysis 47
10. References 48
11. NMR spectra 49

1. General information

All reactions were carried out under nitrogen. Reactions requiring heating were carried out using DrySyn heating block. For flash chromatography, distilled technical grade solvents were used. THF, CH_3CN, Et_2O, CH_2Cl_2 and toluene were dried by passage over activated alumina under nitrogen atmosphere (H_2O content <10 ppm, Karl-Fischer titration). All chemicals were purchased from Acros, Aldrich, Combi-blocks, Fluka, Fluorochem, Merck, TCI or VWR and used as such unless stated otherwise. Chromatographic purification was performed as flash chromatography using Silicycle silica 40-63 µm (230-400 mesh) or basic alumina (Acros, Brockmann activity I, 40-300 µm, 60A), using the solvents indicated as eluent with 0.1-0.5 bar pressure unless stated otherwise. TLC was performed on Merck silica gel 60 F254 TLC glass plates and visualized with UV light and potassium permanganate, *p*-anisaldehyde or ceric ammonium molybdate. ^{1}H-NMR spectra were recorded on a Bruker DPX-400 400 MHz spectrometer in chloroform-d, DMSO-d_6. All signals are reported in ppm using the residual solvent signal as internal reference (chloroform-d: 7.26 ppm, DMSO-d_6: 2.50 ppm)). The data is being reported as (s = singlet, d = doublet, t = triplet, q = quadruplet, qi = quintet, m = multiplet or unresolved, br s = broad signal, coupling constant(s) in Hz, integration, assignment). ^{13}C-NMR spectra were recorded with {^{1}H} decoupling on a Bruker DPX-400 101 MHz spectrometer in chloroform-d, DMSO-d_6. All signals are reported in ppm using the residual solvent signal as internal reference (chloroform-d: 77.0 ppm, DMSO-d_6: 39.5 ppm). ^{19}F-NMR spectra were recorded with {^{1}H} decoupling on a Bruker DPX-400 376 MHz spectrometer in chloroform-d, DMSO-d_6. Structural assignments were made with additional information from gCOSY, gHSQC, and gHMBC experiments. High resolution mass spectrometric measurements were performed by the mass spectrometry service of ISIC at the EPFL. IR spectra were recorded on an Alpha-P Bruker FT-IR Spectrometer. Absorbance frequencies are reported in reciprocal centimeters (cm^{-1}) with indicated relative intensities: s (strong, 0-33% T); m (medium, 34-66% T); w (weak, 67-100% T). Electrospray-ionisation HRMS data were acquired on a Q-Tof Ultima mass spectrometer (Waters) or a Q-Tof 6530 Accurate mass spectrometer (Agilent) operated in the positive ionization mode and fitted with a standard Z-spray ion source equipped with the Lock-Spray interface. Data from the Lock-Spray were used to calculate a correction factor for the mass scale and provide accurate mass information of the analyte. Data were processed using the MassLynx 4.1 software. Atmospheric pressure photo-ionisation (APPI) HRMS measurements were done on a LTQ Orbitrap Elite instrument (Thermofisher) operated in the positive ionization mode. The raw data obtained from the Q-TOF Waters instrument does not take into account the mass of the electron for the ion, the obtained raw data has been corrected by removing (positive ionization) or adding (negative ionization) the mass of the electron (0.5 mDa). Melting points were measured on a Büchi B-540 and are uncorrected. X-ray analyses of compounds **3x** and **3ad** were performed by Dr. R. Scopelliti and Dr. F. Fadaei Tirani at the EPF Lausanne.

2. Starting materials preparation

2.1 Synthesis of dehydroamino acids

NHBoc-Ser-OtBu S1

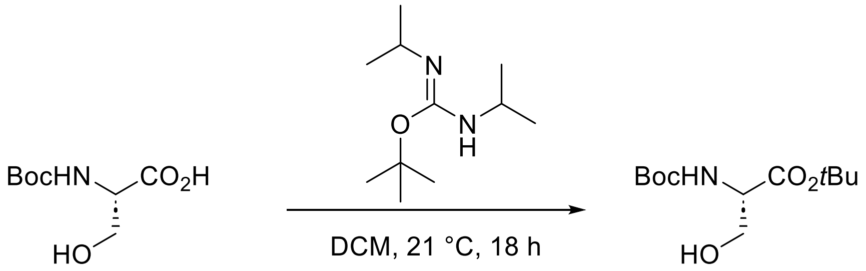

Following a reported procedure,[1] Boc-Ser-OH (500 mg, 2.44 mmol, 1.00 equiv.) was dissolved in DCM (5 mL) and cooled to 0 °C under nitrogen. *tert*-Butyl N,N'-diisopropylcarbamate (1.84 mL, 7.31 mmol, 3.0 equiv.) was added in three portions over 5 min. The reaction mixture was stirred overnight at 21 °C. The reaction mixture was stirred with hexanes (7 mL) for 30 min, then filtered through celite and concentrated under reduced pressure. The crude was purified by column chromatography (SiO_2, pentane:EtOAc = 75:25 to 1:1) to afford Boc-Ser-O*tBu* **S1** (454 mg, 1.74 mmol, 71%) as a colorless oil. **R_f** (pentane:EtOAc = 6:4) = 0.62. **^{1}H NMR** (400 MHz, $CDCl_3$) δ 5.41 (s, 1H, N*H*), 4.25 (s, 1H, CH_2CH), 3.94 – 3.84 (m, 2H, CH_2), 2.35 (s, 1H, O*H*), 1.48 (s, 9H, $C(CH_3)_3$), 1.45 (s, 9H, $C(CH_3)_3$). **^{13}C NMR** (101 MHz, $CDCl_3$) δ 169.7, 155.9, 82.7, 80.2, 64.2, 56.4, 28.3, 28.0. Spectroscopic data was consistent with the values reported in the literature.[1]

NHBoc-Ser-OBn S2

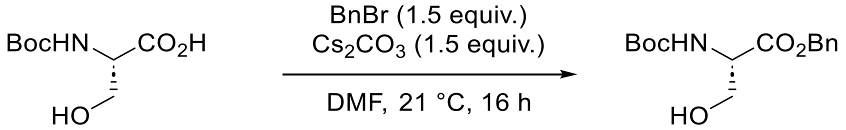

Following a reported procedure,[2] cesium carbonate (9.75 g, 30.0 mmol, 1.50 equiv.) was added to a solution of Boc-Ser-OH (4.10 g, 20.0 mmol, 1.00 equiv.) in DMF (40 mL). The reaction mixture was stirred at 21 °C for 30 min. Benzyl bromide (3.56 mL, 30.0 mmol, 1.50 equiv.) was then added dropwise and the resulting solution was stirred at 21 °C for 16 h. The mixture was then diluted with ethyl acetate (35 mL). The organic layer was washed successively with a saturated aqueous solution of NH_4Cl (30 mL), $NaHCO_3$ (30 mL) and brine (30 mL), dried over $MgSO_4$ and concentrated under reduced pressure. The crude was purified by column chromatography (SiO_2, pentane:EtOAc = 100:0 to 1:1) to afford Boc-Ser-OBn **S2** (2.10 g, 7.10 mmol, 35%) as a colorless oil. **R_f** (pentane:EtOAc = 8:2) = 0.45. **^{1}H NMR** (400 MHz, $CDCl_3$) δ 7.42 – 7.29 (m, 5H, 5 x Ar*H*), 5.43 (s, 1H, N*H*), 5.22 (d, *J* = 2.5 Hz, 2H, CH_2Ar), 4.43 (s, 1H, CH_2CH), 4.05 – 3.87 (m, 2H, CH_2CH), 2.14 (s, 1H, O*H*), 1.44 (s, 9H, $C(CH_3)_3$). **^{13}C NMR** (101 MHz, $CDCl_3$) δ 170.8, 155.9, 135.3, 128.8, 128.7, 128.3, 80.5, 67.6, 63.8, 56.0, 28.4. Spectroscopic data was consistent with the values reported in the literature.[2]

---

[1] C. Hall, H. Wolfe, A. Wells, H.-C. Chien, C. Colas, A. Schlessinger, K. M. Giacomini, A. A. Thomas, *Bioorg. Med. Chem. Lett.* **2019**, *29*, 2254–2258.

[2] F. Friscourt, C. J. Fahrni, G.-J. Boons, *J. Am. Chem. Soc.* **2012**, *134*, 18809–18815.

General procedures for the synthesis of dehydroamino acids

Procedure A

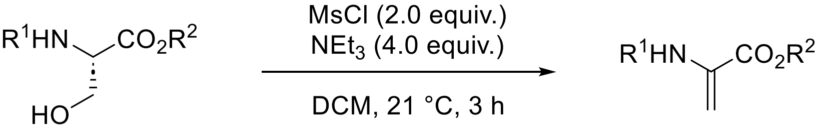

Following a reported procedure,[3] MsCl (2.0 equiv.) was added dropwise to a solution of serine (1.0 equiv.) and Et_3N (4.0 equiv.) in DCM. The reaction was stirred at 21 °C for 3 hours, then quenched with saturated $NaHCO_3$ solution and extracted with DCM. The organic layer was dried over anhydrous $MgSO_4$ and concentred under reduced pressure. The crude was purified by column chromatography.

***N*HBoc-Dha-*O*Me 1b**

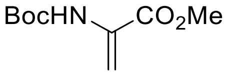

Synthesized following general procedure A, starting from commercially available Boc-Ser-OMe (1.00 mL, 4.69 mmol). Purification by column chromatography (SiO_2, pentane:EtOAc = 100:0 to 8:2) afforded Boc-Dha-OMe **1b** (0.940 g, 4.67 mmol, 100%) as a colorless oil. **^{1}H NMR** (400 MHz, $CDCl_3$) δ 7.00 (s, 1H, N*H*), 6.15 (s, 1H, C*H$_a$*), 5.72 (d, *J* = 1.5 Hz, 1H, C*H$_b$*), 3.82 (s, 3H, OC*H$_3$*), 1.47 (s, 9H, C(C*H$_3$*)$_3$). **^{13}C NMR** (101 MHz, $CDCl_3$) δ 164.6, 152.7, 131.4, 105.3, 80.8, 53.0, 28.4. Spectroscopic data was consistent with the values reported in the literature.[4]

***N*HBoc-Dha-*O*Bn 1g**

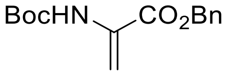

Synthesized following general procedure A, starting from Boc-Ser-OBn **S2** (14.8 g, 50.1 mmol). Purification by column chromatography (SiO_2, pentane:EtOAc = 98:2 to 95:5) afforded *N*HBoc-Dha-OBn **1g** (4.76 g, 17.2 mmol, 34%) as a white solid. **R_f** (pentane:EtOAc = 97:3) = 0.59. **^{1}H NMR** (400 MHz, $CDCl_3$) δ 7.43 – 7.30 (m, 5H, 5 x Ar*H*), 7.03 (s, 1H, N*H*), 6.18 (s, 1H, C*H$_a$*), 5.79 (d, *J* = 1.5 Hz, 1H, C*H$_b$*), 5.26 (s, 2H, C*H$_2$*Ph), 1.48 (s, 9H, C(C*H$_3$*)$_3$). **^{13}C NMR** (101 MHz, $CDCl_3$) δ 164.0, 152.7, 135.3, 131.5, 128.8, 128.7, 128.3, 105.6, 80.9, 67.8, 28.4. Spectroscopic data was consistent with the values reported in the literature.[5]

***N*HFmoc-Dha-*O*Me 1c**

[3] N. Sabat, F. Soualmia, P. Retailleau, A. Benjdia, O. Berteau, X. Guinchard, *Org. Lett.* **2020**, *22*, 4344–4349.
[4] J.-A. Shin, J. Kim, H. Lee, S. Ha, H.-Y. Lee, *J. Org. Chem.* **2019**, *84*, 4558–4565.
[5] I. S. Kondratov, M. Ya. Bugera, N. A. Tolmachova, C. G. Daniliuc, G. Haufe, *J. Fluor. Chem.* **2018**, *211*, 100–108.

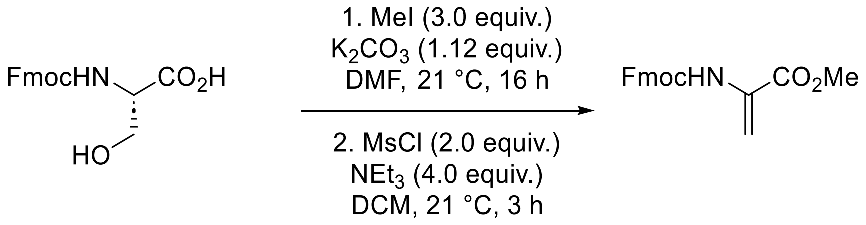

Following a reported procedure,[6] Fmoc-Ser-OH (1.00 g, 3.10 mmol, 1.00 equiv.) and potassium carbonate (498 mg, 3.60 mmol, 1.12 equiv.) were dissolved in DMF (10 mL). The mixture was stirred for 30 min at 21 °C and cooled to 0 °C. Then methyl iodide (0.560 mL, 9.00 mmol, 3.00 equiv.) was added. After 16 h, the reaction mixture was diluted with water (100 mL) and extracted three times with Et_2O (50 mL). The organic layer was combined, dried over $MgSO_4$ and concentrated under reduced pressure to afford Fmoc-Ser-OMe. The crude was used without any further purification. Following general procedure A, Fmoc-Dha-OMe **1c** (527 mg, 1.63 mmol, 53%) was obtained as a white solid from Fmoc-Ser-OMe (1.04 g, 3.05 mmol) after purification by column chromatography (SiO_2, pentane:EtOAc = 100:0 to 8:2). $\mathbf{R_f}$ (pentane:EtOAc = 95:5) = 0.23. **^{1}H NMR** (400 MHz, $CDCl_3$) δ 7.78 (d, 2H, 2 x Ar*H*), 7.60 (d, *J* = 0.9 Hz, 2H, 2 x Ar*H*), 7.42 (t, *J* = 0.9 Hz, 2H, 2 x Ar*H*), 7.33 (td, *J* = 7.4, 1.2 Hz, 2H, 2 x Ar*H*), 6.24 (s, 1H, CH_a), 5.80 (s, 1H, CH_b), 4.46 (d, *J* = 7.0 Hz, 2H, CH_2), 4.26 (t, 1H, C*H*), 3.86 (s, 3H, CH_3). **^{13}C NMR** (101 MHz, $CDCl_3$) δ 164.5, 158.0, 143.8, 141.5, 131.1, 128.0, 127.3, 125.2, 120.2, 106.4, 67.3, 53.2, 47.1. Spectroscopic data was consistent with the values reported in the literature.[7]

*N*HBoc-Dhb-*O*Me S3

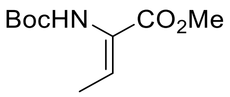

Synthesized following general procedure A, starting from Boc-Thr-OMe (3.23 g , 13.8 mmol). Purification by column chromatography (SiO_2, pentane:EtOAc = 100:0 to 8:2) afforded *N*HBoc-Dhb-OMe **S3** (1.67 g, 5.30 mmol, 38%) as a colorless oil. $\mathbf{R_f}$ (pentane:EtOAc = 9:1) = 0.72. **^{1}H NMR** (400 MHz, $CDCl_3$) δ 6.67 (q, *J* = 7.2 Hz, 1H, C*H*), 5.97 (s, 1H, N*H*), 3.77 (s, 3H, OCH_3), 1.80 (d, *J* = 7.2 Hz, 3H, CH_3), 1.46 (s, 9H, C(CH_3)$_3$). **^{13}C NMR** (101 MHz, $CDCl_3$) δ 165.4, 153.1, 132.1, 126.7, 80.5, 52.3, 28.2, 14.3. Spectroscopic data was consistent with the values reported in the literature.[8]

<u>Procedure B</u>

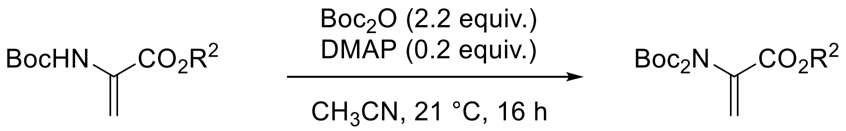

[6] F. Zhang, W. Zhang, Y. Zhang, D. P. Curran, G. Liu, *J. Org. Chem.* **2009**, *74*, 2594–2597.
[7] S. Koch, D. Schollmeyer, H. Löwe, H. Kunz, *Chem. - Eur. J.* **2013**, *19*, 7020–7041.
[8] G. Occhialini, V. Palani, A. E. Wendlandt, *J. Am. Chem. Soc.* **2022**, *144*, 145–152.

Following a reported procedure,[9] to a solution of Boc-Dha (1.00 equiv.) in CH_3CN were added Boc_2O (2.20 equiv.) and DMAP (0.20 equiv.) The reaction was stirred at 21 °C for 16 hours. The solvent was removed under reduced pressure and the crude material was purified by column chromatography.

N**Boc$_2$-Dha-*****O*****Me 1a**

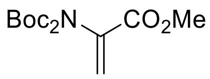

Synthesized following general procedure B, starting from Boc-Dha-OMe **1b** (1.18 g, 5.86 mmol). Purification by column chromatography (SiO_2, pentane:EtOAc = 95:5 to 8:2) afforded *N*Boc$_2$-Dha-OMe **1a** (1.43 g, 4.75 mmol, 81%) as a white solid. **R_f** (pentane:EtOAc = 9:1) = 0.37. **^{1}H NMR** (400 MHz, $CDCl_3$) δ 6.34 (s, 1H, CH_a), 5.64 (s, 1H, CH_b), 3.79 (s, 3H, OCH_3), 1.46 (s, 18H, 2 x C(CH_3)$_3$). **^{13}C NMR** (101 MHz, $CDCl_3$) δ 164.2, 150.8, 136.2, 124.8, 83.3, 52.5, 28.0. Spectroscopic data was consistent with the values reported in the literature.[9]

N**Boc$_2$-Dha-*****OtBu*** **1e**

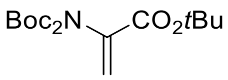

Synthesized following general procedure B, starting from Boc-Dha-OBn **S1** (470 mg, 1.93 mmol). Purification by column chromatography (SiO_2, pentane:EtOAc = 95:5 to 8:2) afforded *N*Boc$_2$-Dha-O*t*Bu **1e** (439 mg, 1.28 mmol, 66%) as a white solid. **R_f** (pentane:EtOAc = 9:1) = 0.33. **^{1}H NMR** (400 MHz, $CDCl_3$) δ 6.26 (s, 1H, CH_a), 5.56 (s, 1H, CH_b), 1.50 (s, 9H, C(CH_3)$_3$), 1.46 (s, 18H, 2 x C(CH_3)$_3$). **^{13}C NMR** (101 MHz, $CDCl_3$) δ 162.4, 150.7, 137.4, 123.8, 82.8, 81.6, 28.0, 27.9. Spectroscopic data was consistent with the values reported in the literature.[10]

N**Boc$_2$-Dha-*****O*****Bn 1f**

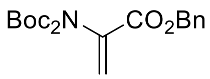

Synthesized following general procedure B, starting from Boc-Dha-OBn **1h** (1.97 g, 7.10 mmol). Purification by column chromatography (SiO_2, pentane:EtOAc = 95:5 to 9:1) afforded *N*Boc$_2$-Dha-OBn **1f** (2.12 g, 5.61 mmol, 79%) as a white solid. **R_f** (pentane:EtOAc = 95:5) = 0.41. **^{1}H NMR** (400 MHz, $CDCl_3$) δ 7.40 – 7.29 (m, 5H, 5 x Ar*H*), 6.39 (s, 1H, C*H*), 5.67 (s, 1H, C*H*), 5.24 (s, 2H, OCH_2Ph), 1.41 (s, 18H, 2 x C(CH_3)$_3$). **^{13}C NMR** (101 MHz, $CDCl_3$): δ 163.3, 150.6, 136.2, 135.5, 128.5, 128.3, 128.2, 15.1, 83.1, 67.0, 27.8. Spectroscopic data was consistent with the values reported in the literature.[5]

N**Boc$_2$-Dhb-*****O*****Me 1h**

[9] R. Petracca, K. A. Bowen, L. McSweeney, S. O'Flaherty, V. Genna, B. Twamley, M. Devocelle, E. M. Scanlan, *Org. Lett.* **2019**, *21*, 3281–3285.

[10] S. B. Vogensen, R. P. Clausen, J. R. Greenwood, T. N. Johansen, D. S. Pickering, B. Nielsen, B. Ebert, P. Krogsgaard-Larsen, *J. Med. Chem.* **2005**, *48*, 3438–3442.

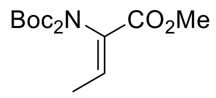

Synthesized following general procedure B, starting from Boc-Dhb-OMe **S3** (1.97 g, 7.10 mmol). Purification by column chromatography (SiO_2, pentane:EtOAc = 95:5 to 9:1) afforded *N*Boc$_2$-Dha-OBn **1h** (2.12 g, 5.61 mmol, 79%) as a white solid. **R$_f$** (pentane/EtOAc = 95:5) = 0.41. **^{1}H NMR** (400 MHz, $CDCl_3$) δ 6.89 (q, *J* = 7.1 Hz, 1H, C*H*), 3.76 (s, 3H, OC*H$_3$*), 1.76 (d, *J* = 7.1 Hz, 3H, C*H$_3$*), 1.45 (s, 18H, 2 x C(C*H$_3$*)$_3$). **^{13}C NMR** (101 MHz, $CDCl_3$) δ 164.6, 150.6, 136.8, 130.4, 82.9, 52.3, 28.0, 13.5. Spectroscopic data was consistent with the values reported in the literature.[11]

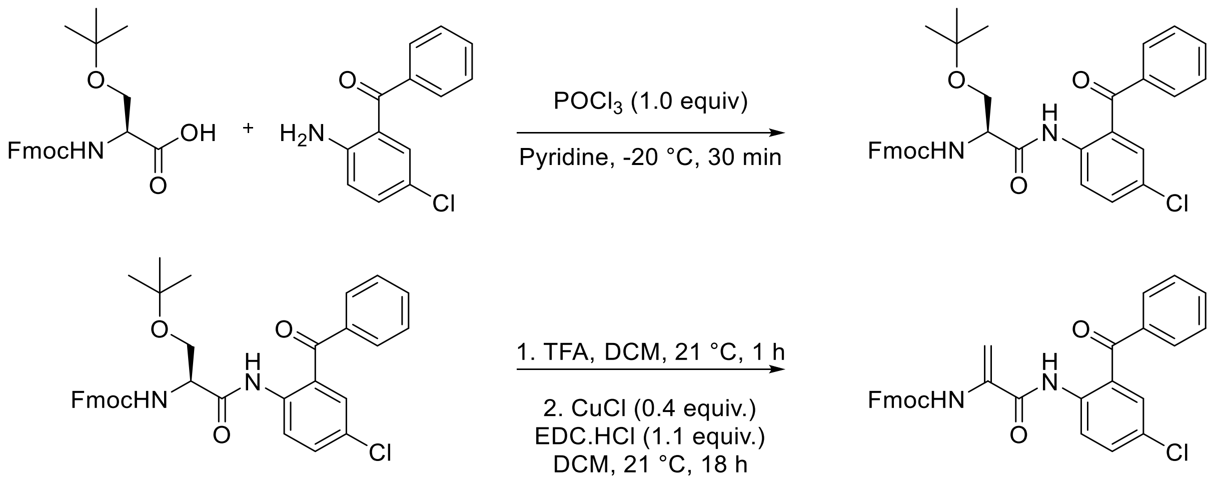

(9H-Fluoren-9-yl)methyl-(R)-(1-((2-benzoyl-4-chlorophenyl)amino)-3-(tert-butoxy)-1-oxopropan-2-yl)carbamate S4

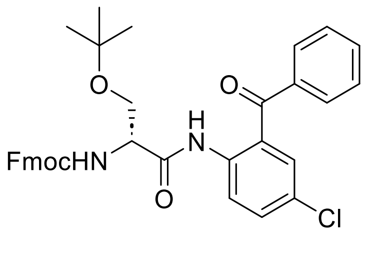

Following a reported procedure,[12] Fmoc-Ser(O*t*Bu)-OH (1.15 g, 3.00 mmol, 1.0 equiv.) and 2-amino-5-chlorobenzophenone (695 mg, 3.00 mmol, 1.00 equiv.) were dissolve in pyridine (7.5 mL) at -20 °C. Phosphoryl chloride (280 µL, 3.00 mmol, 1.00 equiv.) was added dropwise using a syringe pump over 10 min. The solution was stirred for 30 min. Ice cold water was then added and the mixture was extracted three times with EtOAc (3 x 50 mL). The organic layers were gathered, washed with sat. $NaHCO_3$ and brine, dried over $MgSO_4$, filtered off and the solvents were removed under reduced pressure. The crude product was purified by column chromatography (SiO_2, pentane:EtOAc = 8:2 to 6:4) to afford (9H-Fluoren-9-yl)methyl-(R)-(1-((2-benzoyl-4-chlorophenyl)amino)-3-(tert-butoxy)-1-oxopropan-2-yl)carbamate **S4** (1.17 g, 1.97 mmol, 66%) as white solid. **R$_f$** (pentane:EtOAc = 6:4) = 0.70. **^{1}H NMR** (400 MHz, $CDCl_3$) δ

[11] P. M. T. Ferreira, H. L. S. Maia, L. S. Monteiro, J. Sacramento, *J. Chem. Soc. Perkin 1* **1999**, 3697–3703.
[12] S. Heinrich, M. Altenkämper, B. Bechem, J. Perruchon, R. Ortmann, H.-M. Dahse, Y. Wang, M. Lanzer, M. Schlitzer, *Eur. J. Med. Chem.* **2011**, *46*, 1331–1342.

11.25 (s, 1H, N*H*), 8.62 (d, *J* = 8.8 Hz, 1H, Ar*H*), 7.81 – 7.68 (m, 3H, 3 x Ar*H*), 7.67 – 7.49 (m, 6H, 6 x Ar*H*), 7.49 – 7.34 (m, 4H, 4 x Ar*H*), 7.33 – 7.26 (m, 2H, 2 x Ar*H*), 5.87 (d, *J* = 7.2 Hz, 1H, N*H*), 4.58 – 4.40 (m, 2H, CH_{Fmoc} and NC*H*), 4.29 (d, *J* = 6.3 Hz, 2H, CH_{2Fmoc}), 3.98 (dd, J = 9.7, 2.7 Hz, 1H, OCH_a), 3.58 (dd, *J* = 9.1, 5.0 Hz, 1H, OCH_b), 1.15 (s, 9H, C(CH_3)$_3$). **^{13}C NMR** (101 MHz, $CDCl_3$) δ 197.8, 169.9, 156.4, 143.8, 141.43, 141.38, 138.3, 137.9, 133.8, 133.0, 132.6, 130.07, 128.6, 127.8, 127.8, 127.2, 125.6, 123.3, 120.1, 74.1, 67.8, 61.8, 56.7, 47.3, 27.5. **IR** (vmax, cm^{-1}) 3414 (w), 3317 (w), 2974 (m), 1728 (s), 1695 (s), 1506 (s), 1250 (s), 741 (s). **m.p**. 78-80 °C**. HRMS** (ESI/QTOF) m/z: $[M+H]^+$ Calcd for $C_{35}H_{34}ClN_2O_5^+$ 597.2151; Found 597.2162.

(9H-Fluoren-9-yl)methyl-(3-((2-benzoyl-4-chlorophenyl)amino)-3-oxoprop-1-en-2-yl)carbamate 1i

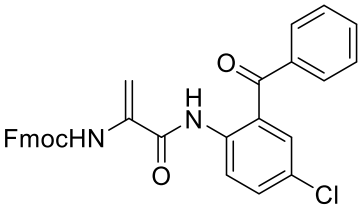

TFA (2.5 mL, 33.0 mmol, 30.0 equiv.) was added to a solution (9H-Fluoren-9-yl)methyl-(R)-(1-((2-benzoyl-4-chlorophenyl)amino)-3-(tert-butoxy)-1-oxopropan-2-yl)carbamate **S4** (657 mg, 1.10 mmol, 1.00 equiv.) in 2.5 mL of DCM at 0 °C. The mixture was stirred 1 h at 21 °C. The mixture was then basified with $NaHCO_3$ and extracted with DCM (3 x 30 mL). The organic layers were gathered, washed with brine, dried over $MgSO_4$, filtered off and the solvents were removed under reduced pressure to afford the intermediate alcohol used without any further purifications. EDC.HCl (211 mg, 1.10 mmol, 1.10 equiv) and CuCl (39.6 mg, 0.400 mmol, 0.400 equiv.) were added to a solution of the crude in DCM (10 mL). The mixture was stirred 18 h at 21 °C. The solution was then filtered over celite and the solvents were removed under reduced pressure. Purification by column chromatography (SiO_2, pentane:EtOAc = 97:3 to 9:1) afforded (9H-fluoren-9-yl)methyl-(3-((2-benzoyl-4-chlorophenyl)amino)-3-oxoprop-1-en-2-yl)carbamate **1i** (300 mg, 0.574 mmol, 52%) as a white solid. $\mathbf{R_f}$ (pentane:EtOAc =9:1) = 0.33. **^{1}H NMR** (400 MHz, $CDCl_3$) δ 11.80 (s, 1H, N*H*), 8.67 (dt, *J* = 9.2, 1.3 Hz, 1H, Ar*H*), 7.78 (d, *J* = 7.5 Hz, 2H, 2 x Ar*H*), 7.74 – 7.70 (m, 2H, 2 x Ar*H*), 7.68 – 7.57 (m, 6H, 5 x Ar*H* and N*H*), 7.58 – 7.49 (m, 2H, 2 x Ar*H*), 7.41 (tt, *J* = 7.5, 1.0 Hz, 2H, 2 x Ar*H*), 7.33 (td, *J* = 7.4, 1.2 Hz, 2H, 2 x Ar*H*), 6.33 (s, 1H, CH_a), 5.62 (dd, *J* = 2.7, 1.6 Hz, 1H, CH_a), 4.47 (d, *J* = 7.2 Hz, 2H, CH_{2Fmoc}), 4.27 (t, *J* = 7.0 Hz, 1H, CH_{Fmoc}). **^{13}C NMR** (101 MHz, $CDCl_3$) δ 199.0, 162.4, 153.5, 143.8, 141.5, 138.8, 137.9, 134.6, 134.5, 133.4, 133.3, 130.0, 128.8, 128.2, 128.0, 127.3, 125.2, 124.6, 122.8, 120.2, 101.0, 67.4, 47.1. **IR** (vmax, cm^{-1}) 3385 (w), 3061 (w), 1735 (m), 1677 (m), 1634 (m), 1497 (s), 1295 (s), 1210 (s), 952 (s), 738 (s). **m.p**. 179-181 °C**. HRMS** (ESI/QTOF) m/z: $[M+H]^+$ Calcd for $C_{31}H_{24}ClN_2O_4^+$ 523.1419; Found 523.1396.

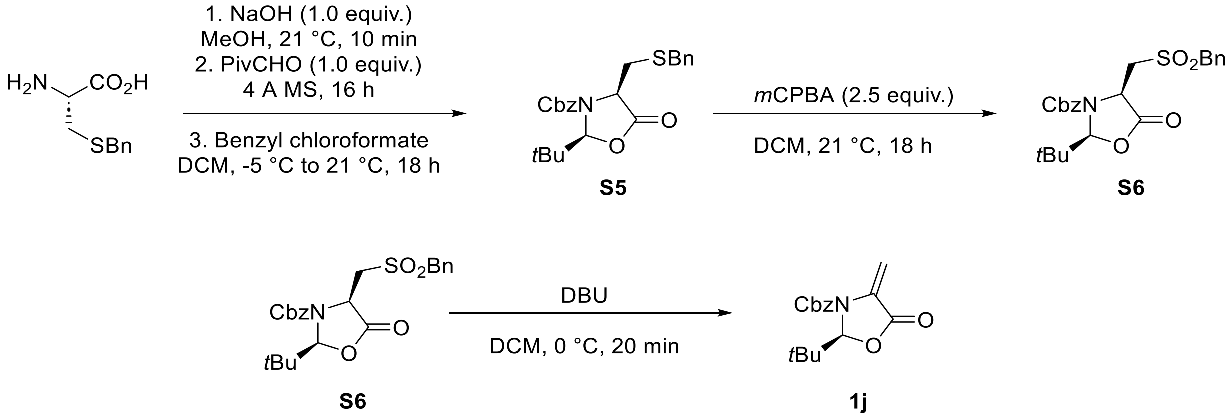

Benzyl (2S,4R)-4-((benzylthio)methyl)-2-(tert-butyl)-5-oxooxazolidine-3-carboxylate S5

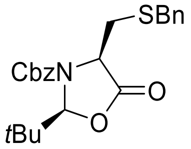

Following a reported procedure,[13] *S*-benzyl-L-cysteine (35.0 g, 166 mmol, 1.00 equiv.) was suspended in anhydrous methanol (550 mL) and NaOH (6.63 g, 166 mmol, 1.00 equiv.) was added in one portion under N_2. The resulting mixture was stirred at room temperature for 20 minutes until all solids were dissolved. Pivaldehyde (21.6 mL, 199 mmol mol, 1.20 equiv) and activated molecular sieves 4A (110 g) were added. The reaction mixture was stirred for 16 h at 21 °C, filtered through a plug of celite, washed with methanol and the solvent were removed under reduced pressure and the solid was dried under high vacuum for 24 h. The resulting white solid was dissolved in DCM (800 mL) and the solution cooled to −5 °C. Benzyl chloroformate (35.5 mL, 249 mmol, 1.5 equiv) was added dropwise over 2 h at −30 °C to the stirred solution using a syringe pump. The mixture was stirred at 0 °C for a further 18 h, then warmed to 21 °C and stirred for an additional 16 h. The mixture was washed with aqueous sodium hydroxide solution (1 M, 200 mL) and brine, and the organic layer dried over $MgSO_4$, filtered and concentrated under reduced pressure. The crude was purified by column chromatography (SiO_2, pentane:EtOAc = 95:5 to 9:1) to afford benzyl (2S,4R)-4-((benzylthio)methyl)-2-(*tert*-butyl)-5-oxooxazolidine-3-carboxylate **S5** (24.6 g, 59.4 mmol, 36%) as colorless oil. $\mathbf{R_f}$ (pentane:EtOAc = 9:1) = 0.49. $\mathbf{^1H}$ **NMR** (400 MHz, $CDCl_3$) δ 7.40 – 7.33 (m, 5H, 5 x Ar*H*), 7.32 – 7.16 (m, 5H, 5 x Ar*H*), 5.53 (s, 1H, *t*BuCH), 5.25 – 5.14 (m, 2H, OCH_2), 4.53 (dd, *J* = 8.0, 6.1 Hz, 1H, CH_2CH), 3.77 (q, *J* = 13.5 Hz, 2H, SCH_2Ph), 2.92 (dd, *J* = 13.9, 8.0 Hz, 1H, SCH_a), 2.77 (dd, *J* = 13.9, 6.1 Hz, 1H, SCH_b), 0.91 (s, 9H, C(CH_3)$_3$). $\mathbf{^{13}C}$ **NMR** (101 MHz, $CDCl_3$) δ 171.4, 156.0, 137.9, 135.2, 129.2, 128.9, 128.71, 128.66, 127.3, 96.5, 68.7, 57.7, 37.1, 36.7, 33.5, 25.0. Spectroscopic data was consistent with the values reported in the literature.[13]

[13] D. Reich, A. Trowbridge, M. J. Gaunt, *Angew. Chem. Int. Ed.* **2020**, *59*, 2256–2261.

Benzyl (S)-2-(*tert*-butyl)-4-methylene-5-oxooxazolidine-3-carboxylate 1j

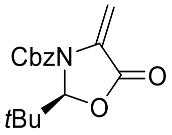

To a stirred solution of benzyl (2S,4R)-4-((benzylthio)methyl)-2-(tert-butyl)-5-oxooxazolidine-3-carboxylate **S5** (24.6 g, 59.4 mmol, 1.00 equiv) in DCM (300 mL) was added *m*CPBA (<77 %, 33.3 g, 149 mmol, 2.50 equiv) portionwise. The solution was stirred at 21 °C for 16 h and then washed with aq. NaOH (1 M, 3 x 100mL). The organic layer was dried over $MgSO_4$, filtered and concentrated under reduced pressure to afford crude benzyl (2S,4R)-4-((benzylsulfonyl)methyl)-2-(tert-butyl)-5-oxooxazolidine-3-carboxylate **S6** as a white solid. The crude was used without any further purification. A stirred solution of crude benzyl (2S,4R)-4-((benzylsulfonyl)methyl)-2-(tert-butyl)-5-oxooxazolidine-3- carboxylate in DCM (150 mL) was cooled to 0 °C, then DBU (9.75mL, 65.3 mmol, 1.10 equiv) was added dropwise using a styringe pump over the course of 10 min. The reaction was stirred for a further 5 minutes. The mixture was then quenched by addition of sat. aq. NH_4Cl (100 mL) at 0 °C, and the aqueous phase extracted with dichloromethane (3 x 100 mL). The combined organic layers were dried over $MgSO_4$, filtered and concentrated under reduced pressure. The crude product was purified by column chromatography (SiO_2, pentane:EtOAc = 99:1 to 9:1) to afford benzyl (S)-2-(*tert*-butyl)-4-methylene-5-oxooxazolidine-3-carboxylate **1j** (12.0 g, 41.5 mmol, 70%) as a white solid. $\mathbf{R_f}$ (pentane:EtOAc = 95:5) = 0.54. **^{1}H NMR** (400 MHz, $CDCl_3$) δ 7.44 – 7.33 (m, 5H, 5 x Ar*H*), 5.72 (d, *J* = 1.1 Hz, 1H, C*H$_a$*), 5.68 (s, 2H, *NCH and CH$_a$*), 5.26 (d, *J* = 1.6 Hz, 2H, PhC*H$_2$*), 0.93 (s, 9H, C(C*H$_3$*)$_3$). **^{13}C NMR** (101 MHz, $CDCl_3$) δ 164.7, 152.5, 134.8, 130.3, 129.0, 128.9, 128.8, 104.6, 94.1, 68.9, 38.8, 24.5. Spectroscopic data was consistent with the values reported in the literature.[13]

2.2 Synthesis of alkyl peroxides

WARNING: Any reaction involving peroxides should be carried out with precaution as they are potential explosives. A blast shield should be used while the reaction is on going and kevlar gloves should be worn while handling peroxides.

Lauroyl peroxide **2a** and tert-butyl peroxybenzoate **2b** were purchased from Thermo Scientific Alfa Aesar.

Procedure C

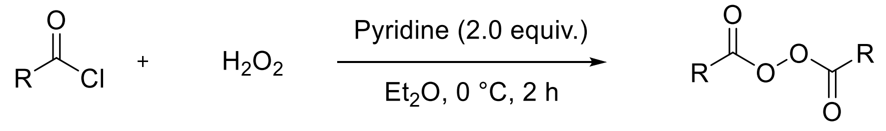

1.0 equiv. 0.55 equiv.

Following a reported procedure,[14] a solution of pyridine (2.0 equiv.) in Et_2O was cooled to -20 °C and H_2O_2 (30% in water, 0.55 equiv.) was added dropwise. The mixture was rapidly stirred so that the two-phase system was finely dispersed. The acid chloride (1.0 equiv.) was then

[14] L. Ge, W. Jian, H. Zhou, S. Chen, C. Ye, F. Yu, B. Qian, Y. Li, H. Bao, *Chem. - Asian J.* **2018**, *13*, 2522–2528.

added dropwise. The mixture was then stirred for additional 2 hours at 0 °C and carefully neutralized with a chilled 10 % H_2SO_4 solution. Et_2O was added and the peroxide was extracted keeping at all time the temperature as 0 °C. The aqueous layer was extracted further with Et_2O. The organic layers were gathered, washed with chilled 10 % H_2SO_4 solution, chilled sat. $NaHCO_3$ solution, and finally with brine. The solution was dried over $MgSO_4$ and concentrated under reduced pressure at 0-10 °C. The residue was purified by column chromatography to afford the desired product.

Procedure D

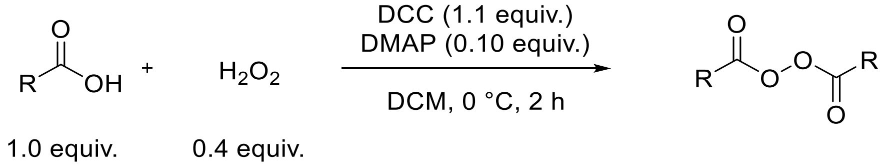

Following a reported procedure,[15] a solution of H_2O_2 (30% in water, 0.40 equiv.) and acid (1.0 equiv.) in DCM (0.3 M) was stirred a 0 °C for 10 min. DMAP (0.1 equiv.) and DCC (1.1 equiv.) were sequentially added and the mixture was stirred 2 h at 0 °C. After addition of hexane (4 x DCM quantity), the mixture was filtered, dried over $MgSO_4$, filtered and solvents were reduced under reduced pressure at 0-10 °C. The residue was purified by column chromatography to afford the desired product.

Procedure E

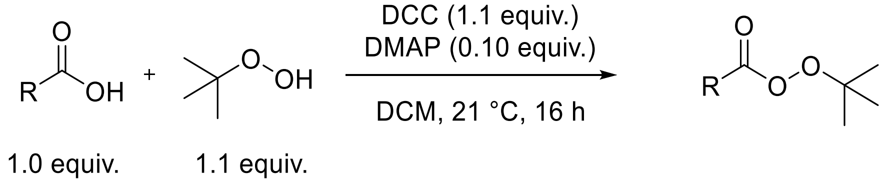

Following a reported procedure,[14] a solution of carboxylic acid (1.0 equiv.) and DMAP (0.1 equiv.) in DCM was cooled to 0 °C and TBPB (70% in water, 1.1 equiv.) was added dropwise. The reaction mixture was stirred for 10 min. DCC (1.1 equiv), and the resulting mixture was stirred at 0 °C for 30 min, then at 21 °C overnight. The reaction solution was filtered. The filtrate was concentrated and purified by column chromatography to afford the desired peroxide.

Diisobutyryl peroxide 2c

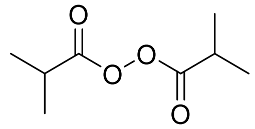

Synthesized following general procedure C, starting from isobutyryl chloride (1.05 mL, 10.0 mmol). Purification by column chromatography (SiO_2, pentane:Et_2O = 100:0 to 98:2) afforded isobutyroyl peroxide **2c** (557 mg, 3.20 mmol, 64%) as a colorless liquid. **R_f** (pentane:Et_2O =

[15] A. Gómez-Palomino, M. Pérez-Palau, P. Romea, F. Urpí, M. Del Olmo, T. Hesse, S. Fleckenstein, E. Gómez-Bengoa, L. Sotorríos, M. Font-Bardia, *Org. Lett.* **2020**, *22*, 199–203.

95:5) = 0.50. **^{1}H NMR** (400 MHz, $CDCl_3$) δ 2.77 (hept, *J* = 7.0 Hz, 2H, 2 x C*H*), 1.31 (s, 12H, 4 x C*H₃*). **^{13}C NMR** (101 MHz, $CDCl_3$) δ 172.9, 31.2, 19.2. Spectroscopic data was consistent with the values reported in the literature.[16]

Diacetyl peroxide 2d

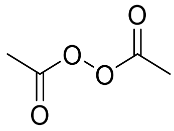

Synthesized following general procedure C, starting from acetyl chloride (2.14 m , 30.0 mmol). Purification by column chromatography (SiO_2, pentane:DCM = 7:3 to 3:7) afforded diacetyl peroxide **2d** (1.01 g, 8.55 mmol, 57%) as a white solid. **R_f** (pentane:DCM = 1:1) = 0.43. **^{1}H NMR** (400 MHz, $CDCl_3$) δ 2.19 (s, 6H, 2 x CH_3). **^{13}C NMR** (101 MHz, $CDCl_3$) δ 166.3, 16.7. Spectroscopic data was consistent with the values reported in the literature.[17]

Diipropionyl peroxide 2e

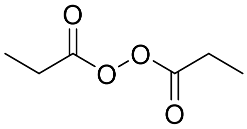

Synthesized following general procedure C, starting from propionyl chloride (2.62 mL, 30.0 mmol). Purification by column chromatography (SiO_2, pentane:DCM = 8:2 to 1:1) afforded dipropionyl peroxide **2e** (1.80 g, 12.0 mmol, 82%) as a colorless liquid. **R_f** (pentane:DCM = 1:1) = 0.42. **^{1}H NMR** (400 MHz, $CDCl_3$) δ 2.46 (q, *J* = 7.5 Hz, 4H, 2 x CH_2), 1.25 (t, *J* = 7.5 Hz, 6H, 4H, 2 x CH_3). **^{13}C NMR** (101 MHz, $CDCl_3$) δ 170.1, 23.7, 9.1. Spectroscopic data was consistent with the values reported in the literature.[16]

Dineopentyl peroxide 2f

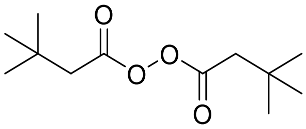

Synthesized following general procedure C, starting from 3,3-dimethylbutanoic acid chloride **2f** (2.00 mL , 14.4 mmol). Purification by column chromatography (SiO_2, pentane:Et_2O = 100:0 to 98:2) afforded dineopentyl peroxide **2f** (1.51 g, 6.56 mmol, 91%) as a white solid. **R_f** (pentane: Et_2O = 95:5) = 0.61. **^{1}H NMR** (400 MHz, $CDCl_3$) δ 2.31 (s, 4H, 2 x CH_2), 1.10 (s, 18H, 6 x CH_3). **^{13}C NMR** (101 MHz, $CDCl_3$) δ 167.6, 43.8, 31.2, 29.6. Spectroscopic data was consistent with the values reported in the literature.[16]

Di(undec-10-enoyl) peroxide 2g

[16] H. Tian, W. Xu, Y. Liu, Q. Wang, *Chem. Commun.* **2019**, *55*, 14813–14816.
[17] S.-M. Hyun, M. Yuan, A. Maity, O. Gutierrez, D. C. Powers, *Chem* **2019**, *5*, 2388–2404.

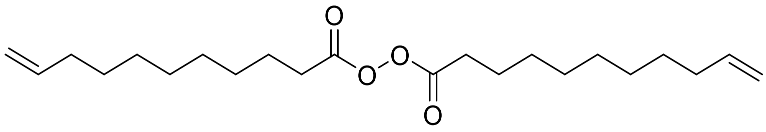

Synthesized following general procedure C, starting from 10-undecenoyl chloride (2.22 mL, 10.0 mmol). Purification by column chromatography (SiO_2, pentane:DCM = 8:2 to 1:1) afforded di-undec-10-enoyl peroxide **2g** (534 mg, 1.46 mmol, 39%) as a colorless oil. **R_f** (pentane: DCM = 1:1) = 0.65. **^{1}H NMR** (400 MHz, $CDCl_3$) δ 5.88 – 5.73 (m, 2H, 2 x $C_{sp2}H$), 5.04 – 4.89 (m, 4H, 2 x $C_{sp2}H_2$), 2.42 (t, *J* = 7.5 Hz, 4H, 2 x $COCH_2$), 2.09 – 1.98 (m, 4H, 2 x CH_2), 1.71 (p, *J* = 7.3 Hz, 4H, 2 x CH_2), 1.42 – 1.19 (m, 20H, 10 x CH_2). **^{13}C NMR** (101 MHz, $CDCl_3$) δ 169.4, 139.3, 114.3, 33.9, 30.2, 29.3, 29.18, 29.15, 29.04, 29.00, 25.0. **IR** (vmax, cm^{-1}) 3076 (w), 2928 (s), 2859 (m), 1815 (s), 1782 (s), 1638 (w), 1457 (m), 1058 (s). **HRMS** (nanochip-ESI/LTQ-Orbitrap) m/z: $[M+Na]^+$ Calcd for $C_{22}H_{38}NaO_4^+$ 389.2662; Found 389.2648.

Di(4-pentanoyl) peroxide 2h

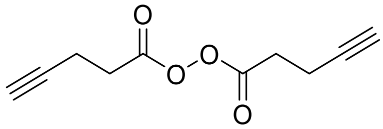

Synthesized following general procedure D, starting from 4-pentynoic acid (1.96 g, 20.0 mmol). Purification by column chromatography (SiO_2, pentane:DCM = 6:4 to 4:6) afforded di(4-pentynoyl) peroxide **2h** (804 mg, 4.14 mmol, 52%) as a white solid. **R_f** (pentane: DCM = 1:1) = 0.33. **^{1}H NMR** (400 MHz, $CDCl_3$) δ 2.70 (ddd, *J* = 8.0, 6.8, 1.4 Hz, 4H, 2 x $OCCH_2$), 2.60 (dddd, *J* = 8.0, 6.9, 2.5, 1.4 Hz, 4H, 2 x CH_2), 2.04 (t, *J* = 2.6 Hz, 2H, 2 x CH). **^{13}C NMR** (101 MHz, $CDCl_3$) δ 167.5, 81.2, 70.2, 29.5, 14.5. **m.p.** 52-54 °C. **IR** (vmax, cm^{-1}) 3281 (m), 2931 (m), 2856 (m), 2118 (s), 1806 (m), 1781 (m), 1451 (m), 1081 (s), 892 (m). HRMS (APPI/LTQ-Orbitrap) m/z: $[M+H]^+$ Calcd for $C_{10}H_{11}O_4^+$ 195.0652; Found 195.0652.

Di(5-hexynoyl) peroxide 2i

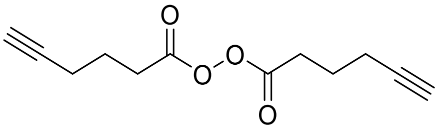

Synthesized following general procedure D, starting from 4-hexynoic acid (2.44 g, 21.8 mmol). Purification by column chromatography (SiO_2, pentane:DCM = 8:2 to 4:6) afforded di(5-hexynoyl) peroxide **2i** (1.81 g, 8.14 mmol, 94%) as a colorless liquid. **R_f** (pentane: DCM = 1:1) = 0.34. **^{1}H NMR** (400 MHz, $CDCl_3$) δ 2.60 (t, *J* = 7.3 Hz, 4H, 2 x $OCCH_2$), 2.33 (td, *J* = 6.9, 2.6 Hz, 4H, 2 x $CCCH_2$), 2.00 (t, *J* = 2.6 Hz, 2H, 2 x CCH), 1.94 (p, *J* = 7.1 Hz, 4H, 2 x CH_2). **^{13}C NMR** (101 MHz, $CDCl_3$) δ 168.8, 82.6, 69.9, 28.8, 23.7, 17.8. Spectroscopic data was consistent with the values reported in the literature.[15]

Di(5-chloropentanoyl) peroxide 2j

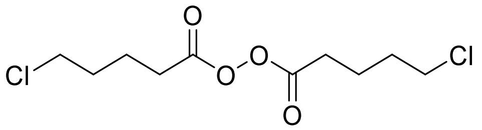

Synthesized following general procedure C, starting from 5-chlorovaleroyl chloride (1.29 mL, 10.0 mmol). Purification by column chromatography (SiO_2, pentane:Et_2O = 100:0 to 95:5) afforded 5-chloropentanoyl peroxide **2j** (942 mg, 3.47 mmol, 69%) as a colorless liquid. **R$_f$** (pentane: Et_2O = 95:5) = 0.19. **^{1}H NMR** (400 MHz, $CDCl_3$) δ 3.62 – 3.50 (m, 4H), 2.55 – 2.42 (m, 4H), 1.96 – 1.82 (m, 8H). **^{13}C NMR** (101 MHz, $CDCl_3$) δ 168.8, 44.3, 31.5, 29.3, 22.2. Spectroscopic data was consistent with the values reported in the literature.[14]

Di(cyclopropanecarboxyl) peroxide 2k

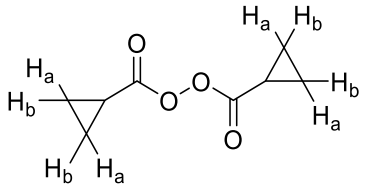

Synthesized following general procedure C, starting from cyclopropanecarbonyl chloride (1.81 mL, 20.0 mmol). Purification by column chromatography (SiO_2, pentane:Et_2O = 100:0 to 95:5) afforded Isopropryl peroxide **2k** (942 mg, 3.47 mmol, 56%) as a white solid. **R$_f$** (pentane: Et_2O = 95:5) = 0.24. **^{1}H NMR** (400 MHz, $CDCl_3$) δ 1.73 (tt, *J* = 8.0, 4.6 Hz, 2H, 2 x C*H*), 1.19 – 1.10 (m, 4H, 4 x C*H$_a$*), 1.08 – 1.00 (m, 4H, 4 x C*H$_b$*). **^{13}C NMR** (101 MHz, $CDCl_3$) δ 171.1, 9.6, 9.5. Spectroscopic data was consistent with the values reported in the literature.**Error! Bookmark not defined.**

Di(cyclobutanecarboxyl) peroxide 2l

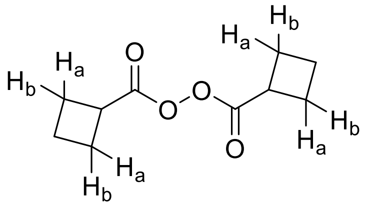

Synthesized following general procedure C, starting from cyclobutanecarbonyl chloride (4.56 mL, 40.0 mmol). Purification by column chromatography (SiO_2, pentane:DCM = 9:1 to 7:3) afforded Isopropryl peroxide **2l** (1.80 g, 9.10 mmol, 46%) as a colorless liquid. **R$_f$** (pentane: DCM = 7:3) = 0.53. **^{1}H NMR** (400 MHz, $CDCl_3$) δ 3.33 – 3.23 (m, 2H, 2 x C*H*), 2.49 – 2.37 (m, 4H, 4 x C*H$_a$*), 2.37 – 2.23 (m, 4H, 4 x C*H$_b$*), 2.12 – 1.96 (m, 4H, 2 x C*H$_2$*). **^{13}C NMR** (101 MHz, $CDCl_3$) δ 171.3, 34.3, 25.6, 19.1. Spectroscopic data was consistent with the values reported in the literature.**Error! Bookmark not defined.**

Di(cyclopentanecarboxyl) peroxide 2m

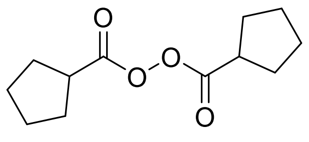

Synthesized following general procedure C, starting from cyclopentanecarbonyl chloride (4.83 mL, 40.0 mmol). Purification by column chromatography (SiO_2, pentane:DCM = 9:1 to 7:3) afforded Isopropryl peroxide **2m** (2.10 g, 9.28 mmol, 46%) as a colorless liquid. **R$_f$** (pentane: DCM = 8:2) = 0.38. **^{1}H NMR** (400 MHz, $CDCl_3$) δ 2.93 – 2.78 (m, 2H, 2 x C*H*), 2.05 – 1.85 (m, 8H, 4 x CH_2), 1.84 – 1.70 (m, 4H, 2 x CH_2), 1.68 – 1.54 (m, 4H, 2 x CH_2). **^{13}C NMR** (101 MHz, $CDCl_3$) δ 171.3, 43.6, 30.1, 26.0. Spectroscopic data was consistent with the values reported in the literature.**Error! Bookmark not defined.**

Di(cyclohexanecarboxyl) peroxide 2n

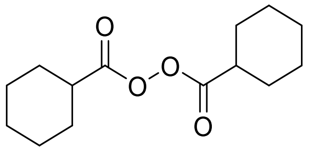

Synthesized following general procedure C, starting from cyclopentanecarbonyl chloride (5.36 mL, 40.0 mmol). Purification by column chromatography (SiO_2, pentane:DCM = 9:1 to 7:3) afforded Isopropryl peroxide **2n** (2.23 g, 12.8 mmol, 44%) as a colorless liquid. **R$_f$** (pentane: DCM = 8:2) = 0.39. **^{1}H NMR** (400 MHz, $CDCl_3$) δ 2.51 (tt, *J* = 11.1, 3.7 Hz, 2H), 2.02 – 1.91 (m, 4H), 1.84 – 1.74 (m, 4H), 1.69 – 1.50 (m, 4H), 1.38 – 1.22 (m, 8H). **^{13}C NMR** (101 MHz, $CDCl_3$) δ 170.5, 43.1, 31.1, 28.4, 25.1. Spectroscopic data was consistent with the values reported in the literature.**Error! Bookmark not defined.**

***tert*-Butyl peroxypivalate 2o**

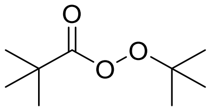

Synthesized following general procedure E, starting from pivalic acid (1.13 mL, 9.80 mmol). Purification by column chromatography (SiO_2, pentane:EtOAc = 99:1 to 98:2) afforded *tert*-butyl peroxypivalate **2o** (611 mg, 3.51 mmol, 36%) as a white solid. **R$_f$** (pentane: EtOAc = 98:2) = 0.50. **^{1}H NMR** (400 MHz, $CDCl_3$) δ 1.32 (s, 9H, C(CH_3)$_3$), 1.25 (s, 9H, OC(CH_3)$_3$). **^{13}C NMR** (101 MHz, $CDCl_3$) δ 175.1, 83.4, 38.9, 27.3, 26.1. Spectroscopic data was consistent with the values reported in the literature.[18]

***tert*-Butyl undec-10-enoyl peroxide 2p**

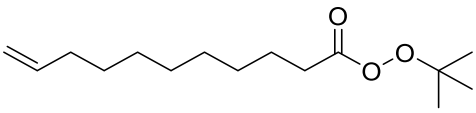

Synthesized following general procedure E, starting from undec-10-enoic acid (921 mg, 5.00 mmol). Purification by column chromatography (SiO_2, pentane:DCM = 8:2 to 4:6) afforded *tert*-butyl undec-10-enoyl peroxide **2p** (1.21 g, 4.74 mmol, 95%) as a colorless liquid. **R$_f$** (pentane: DCM = 1:1) = 0.47. **^{1}H NMR** (400 MHz, $CDCl_3$) δ 5.80 (ddt, *J* = 16.9, 10.2, 6.7 Hz, 1H, $C_{sp^2}H$), 5.04 – 4.88 (m, 2H, $C_{sp^2}H_2$), 2.30 (t, *J* = 7.5 Hz, 2H, OCCH_2), 2.08 – 1.98 (m, 2H, $C_{sp^2}CH_2$),

[18] C. Ye, B. Qian, Y. Li, M. Su, D. Li, H. Bao, *Org. Lett.* **2018**, *20*, 3202–3205.

1.77 – 1.60 (m, 2H, OCCH_2CH_2), 1.32 (s, 19H, C(CH_3)$_3$ and 5 x CH_2). **^{13}C NMR** (101 MHz, $CDCl_3$) δ 171.3, 139.3, 114.3, 83.4, 33.9, 31.5, 29.4, 29.22, 29.20, 29.15, 29.0, 26.3, 25.1. **IR** (vmax, cm^{-1}) 2986 (m), 2925 (s), 2856 (m), 2121 (m), 1778 (s), 1368 (m), 1191 (m), 1076 (m). **HRMS** (ESI/QTOF) m/z: [M+Na]+ Calcd for $C_{15}H_{28}NaO_3^+$ 279.1931; Found 279.1937.

***tert*-Butyl tetrahydropyrane-4-carboperoxoate 2q**

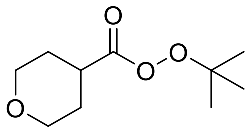

Synthesized following general procedure E, starting from tetrahydropyran-4-carboxylic acid (2.60 g, 20.0 mmol). Purification by column chromatography (SiO_2, pentane:EtOAc = 95:5 to 8:2) afforded *tert*-butyl tetrahydropyrane-4-carboperoxoate **2q** (2.95 g, 14.6 mmol, 73%) as a colorless liquid. **R_f** (pentane: EtOAc = 9:1) = 0.38. **^{1}H NMR** (400 MHz, $CDCl_3$) δ 3.98 (dt, *J* = 11.8, 3.7 Hz, 2H, 2 x OCH_a), 3.49 – 3.39 (m, 2H, 2 x OCH_b), 2.63 (tt, *J* = 10.2, 5.0 Hz, 1H, C*H*), 1.87 – 1.80 (m, 4H, 2 x CH_2), 1.32 (s, 9H, C(CH_3)$_3$). **^{13}C NMR** (101 MHz, $CDCl_3$) δ 171.7, 83.7, 67.0, 38.5, 28.7, 26.3. Spectroscopic data was consistent with the values reported in the literature.[18]

1-(((9H-Fluoren-9-yl)methoxy)carbonyl)piperidine-4-*tert*butyl peroxide 2r

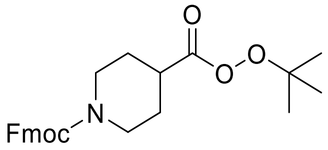

Synthesized following general procedure E, starting from 1-(((9H-fluoren-9-yl)methoxy)carbonyl)piperidine-4-carboxylic acid (3.51 g, 10.0 mmol). Purification by column chromatography (SiO_2, pentane:EtOAc = 9:1 to 7:3) afforded 1-(((9H-fluoren-9-yl)methoxy)carbonyl)piperidine-4-tertbutyl peroxide **2r** (2.88 g, 6.80 mmol, 68%) as a visqueous oil. **R_f** (pentane: EtOAc = 8:2) = 0.43. **^{1}H NMR** (400 MHz, $CDCl_3$) δ 7.77 (d, *J* = 7.5 Hz, 2H, 2 x Ar*H*), 7.57 (dd, *J* = 7.5, 1.1 Hz, 2H, 2 x Ar*H*), 7.40 (t, *J* = 7.3 Hz, 2H, 2 x Ar*H*), 7.32 (td, *J* = 7.5, 1.3 Hz, 2H, 2 x Ar*H*), 4.45 (s, 2H, CH_2Fmoc), 4.24 (t, *J* = 6.7 Hz, 1H, C*H*Fmoc), 4.00 (s, 2H, 2 x NCH_a), 2.92 (t, *J* = 10.9 Hz, 2H, 2 x NCH_b), 2.55 (tt, *J* = 10.9, 4.0 Hz, 1H, C*H*), 1.87 (s, 2H, 2 x CHCH_a), 1.68 (s, 2H, 2 x CHCH_b), 1.33 (s, 9H, C(CH_3)$_3$). **^{13}C NMR** (101 MHz, $CDCl_3$) δ 171.6, 155.2, 144.1, 141.5, 127.8, 127.2, 125.1, 120.1, 83.8, 67.4, 47.5, 43.2, 39.2, 28.0, 26.3. **IR** (vmax, cm^{-1}) 3065 (w), 2981 (m), 2939 (w), 2859 (w), 2117 (w), 1768 (s), 1698 (s), 1476 (m), 1451 (m), 1429 (m), 1368 (m), 1220 (m), 1134 (s), 1076 (m), 1022 (m). **HRMS** (ESI/QTOF) m/z: $[M+Na]^+$ Calcd for $C_{25}H_{29}NNaO_5^+$ 446.1938; Found 446.1935.

***tert*-Butyl adamantyl peroxide 2s**

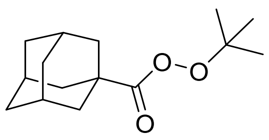

Synthesized following general procedure E, starting from adamantane carboxylic acid (901 mg, 5.00 mmol). Purification by column chromatography (SiO_2, pentane:EtOAc = 8:2 to 1:1)

afforded *tert*-butyl adamantyl peroxide **2s** (734 mg, 2.91 mmol, 58%) as a white solid. **R_f** (pentane: EtOAc = 7:3) = 0.44. **^{1}H NMR** (400 MHz, $CDCl_3$) δ 2.06 – 2.00 (m, 3H, 3 x C*H*), 1.98 – 1.94 (m, 6H, 3 x CC*H*$_2$), 1.78 – 1.68 (m, 6H, 3 x C*H*$_2$), 1.32 (s, 9H, C(C*H*$_3$)$_3$). **^{13}C NMR** (101 MHz, $CDCl_3$) δ 174.4, 83.5, 41.3, 39.0, 36.5, 28.0, 26.3. Spectroscopic data was consistent with the values reported in the literature.[18]

2.3 Synthesis of 2,2'-dipyridyldiselenide

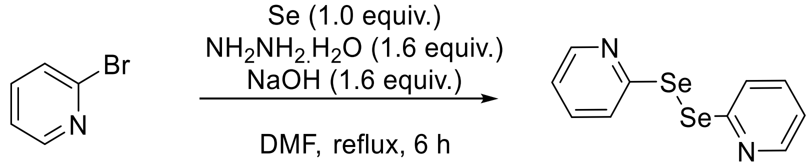

Following a reported procedure,[19] to a rapidly stirred solution of NaOH (2.0 g, 40 mmol, 1.6 equiv) and selenium powder (1.98 g, 25.0 mmol, 1.0 equiv) in DMF (100 mL) was added hydrazine hydrate (1.9 mL, 40 mmol, 1.6 equiv) dropwise at 21 °C. The mixture was stirred for two hours. 2-bromopyridine (2.4 mL, 40 mmol, 1.0 equiv) was added drop-wise to the reaction mixture and refluxed for four hours. The reaction was stopped and diluted with water. The mixture was extracted three times with EtOAc. The organic phases were gathered, washed with LiCl (10% aq. solution). The solution was dried over $MgSO_4$ and concentrated under reduced pressure. Purification by column chromatography (SiO_2, pentane:EtOAc = 9:1 to 7:3) afforded 2,2'-dipyridyldiselenide (740 mg, 2.36 mmol, 19%) as a orange solid. **R_f** (pentane:EtOAc = 1:1) = 0.59. **^{1}H NMR** (400 MHz, $CDCl_3$) δ 8.46 (ddd, *J* = 4.9, 2.0, 1.0 Hz, 2H), 7.80 (dt, *J* = 8.0, 1.1 Hz, 2H), 7.54 (ddd, *J* = 8.0, 7.5, 1.9 Hz, 2H), 7.12 – 7.03 (m, 2H). **^{13}C NMR** (101 MHz, $CDCl_3$) δ 154.7, 149.9, 137.7, 123.8, 121.5. Spectroscopic data was consistent with the values reported in the literature.[19]

[19] M. A. Rizvi, S. Guru, T. Naqvi, M. Kumar, N. Kumbhar, S. Akhoon, S. Banday, S. K. Singh, S. Bhushan, G. Mustafa Peerzada, B. A. Shah, *Bioorg. Med. Chem. Lett.* **2014**, *24*, 3440–3446.

3. Optimization of the alkylzidation of dehydroamino acids

Procedure

To an oven-dried vial, $Fe(OTf)_2$ (5 mol%) was added in a glovebox. The vial was removed from the glovebox and placed under nitrogen. In another oven-dried vial, *N*Boc$_2$-Dha-*O*Me **1a** (0.25 mmol, 1.0 equiv.) and LPO (2.0 equiv.) were dissolved in DME (0.25 M) under nitrogen atmosphere. $TMSN_3$ (2.00 equiv.) was then added. The solution was added to the catalyst vial via syringe and the deep purple mixture was stirred vigorously for 2 hours at 21 °C. The reaction mixture was diluted with EtOAc and filtered through celite/silica. The yield was obtained by ^1H NMR using mesitylene as internal standard (using signal at 2.03 – 1.86 ppm).

Table S1. Optimization of the alkylazidation reaction[a]

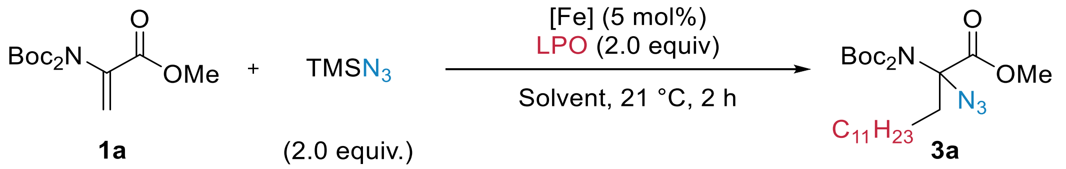

1a (2.0 equiv.) **3a**

Entry	*Catalyst*	*Solvent*	*C (mol.L^{-1})*	*Yield (%)*
1	$Fe(OTf)_2$	DME	0.25	94 (94)[b]
2[c]	$Fe(OTf)_2$	DME	0.25	70
3[d]	$Fe(OTf)_2$	DME	0.25	83
4[e]	$Fe(OTf)_2$	DME	0.25	25
5[f]	$Fe(OTf)_2$	DME	0.25	45
6	$Fe(OTf)_2$	2-MeTHF	0.25	94 (92)[b]
7	$Fe(OTf)_2$	THF	0.25	88
8	$Fe(OTf)_2$	EtOAc	0.25	89
9	$Fe(OTf)_2$	Et_2O	0.25	91
10	$Fe(OTf)_2$	DCM	0.25	85
11	$Fe(OTf)_2$	Acetone	0.25	81
12	$Fe(OTf)_2$	Toluene	0.25	78
13	$Fe(OTf)_2$	CH_3CN	0.25	traces
14	$Fe(OTf)_2$	iPrOH	0.25	traces
15	$Fe(OTf)_2$	DMF	0.25	23
16	$Fe(OTf)_3$	2-MeTHF	0.25	22[b]
17	$Fe(acac)_3$	2-MeTHF	0.25	traces
18	$Fe(OAc)_2$	2-MeTHF	0.25	14
19	$FeCl_3$	2-MeTHF	0.25	70
20	$FeCl_2$	2-MeTHF	0.25	60
21	$Fe(OTf)_2$	2-MeTHF	0.1	93
22	$Fe(OTf)_2$	2-MeTHF	0.5	83
23	$Fe(OTf)_2$	DME	0.1	77

Yields determined by ^1H NMR using mesitylene as internal standard. [b]Isolated yield.[c]1 equiv. of $TMSN_3$ and LPO. [c]1.5 equiv. of $TMSN_3$ and LPO. [d]1 mol% $Fe(OTf)_2$. [e]2.5 mol% $Fe(OTf)_2$.

4. Scope of the alkylazidation of dehydroamino acids

4.1 General procedures

General Procedure F - For solid peroxides

To an oven-dried vial, $Fe(OTf)_2$ (5 mol%) was added in a glovebox. The vial was removed from the glovebox and placed under nitrogen. To another oven-dried vial, dehydroamino acid (1.0 equiv.) and solid peroxide (2.0 equiv.) were dissolved in DME (0.25 M) under nitrogen atmosphere. $TMSN_3$ (2.00 equiv.) was then added. The solution was added to the catalyst vial via syringe and the deep purple mixture was stirred vigorously for 2 hours at 21 °C. The reaction mixture was diluted with EtOAc and filtered through celite/silica. Flash chromatography afforded the desired alkylazidated product.

General Procedure G - For liquid peroxides

To an oven-dried vial, $Fe(OTf)_2$ (5 mol%) was added in a glovebox. The vial was removed from the glovebox and placed under nitrogen. To two different oven-dried vial, dehydroamino acid (1.0 equiv.) and liquid peroxide (2.0 equiv.) were added under nitrogen atmosphere. DME was added to the peroxide vial and it was transferred to the dehydroamino acid vial via syringe. $TMSN_3$ (2.00 equiv.) was then added. The solution was added to the catalyst vial via syringe and the deep purple mixture was stirred vigorously for 2 hours at 21 °C. The reaction mixture was diluted with EtOAc and filtered through celite/silica (1:1). Flash chromatography afforded the desired alkylazidated product.

4.2 Characterization data

Methyl 2-azido-2-(bis(*tert*-butoxycarbonyl)amino)tetradecanoate 3a

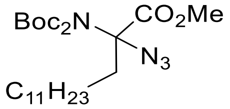

Synthesized following precedure F, starting from *N*Boc$_2$-Dha-*O*Me **1a** (75.3 mg, 0.250 mmol) and LPO (199 mg, 0.500 mmol, 2.00 equiv.). Purification by column chromatography (SiO_2, pentane:EtOAc = 100:0 to 96:4) afforded methyl 2-azido-2-(bis(*tert*-butoxycarbonyl)amino)tetradecanoate **3a** (117 mg, 0.235 mmol, 94%) as a colorless oil. **R$_f$** (pentane: EtOAc = 96:4) = 0.41. **^{1}H NMR** (400 MHz, $CDCl_3$) δ 3.82 (s, 3H, OCH_3), 2.03 – 1.86 (m, 2H, CH_2CN$_3$), 1.51 (s, 18H, 2 x C(CH_3)$_3$), 1.48 – 1.37 (m, 1H, CH_aCH$_2$CN$_3$), 1.34 – 1.17 (m, 18H, C$_9$$H_{18}$), 1.16 – 1.04 (m, 1H, C$H_bCH_2CN_3$), 0.88 (t, *J* = 6.9 Hz, 3H, CH_3). **^{13}C NMR** (101 MHz, $CDCl_3$) δ 168.0, 151.8, 84.2, 80.1, 53.3, 36.1, 32.1, 29.76, 29.70, 29.56, 29.48, 29.44, 29.38, 27.9, 23.9, 22.8, 14.3 (1C not resolved). **IR** (vmax, cm^{-1}) 2979 (w), 2925 (m), 2854 (m), 2118 (m), 1756 (s), 1719 (s), 1458 (m), 1369 (m), 1341 (s), 1249 (s), 1164 (s), 1127 (s). **HRMS** (ESI/QTOF) m/z: [M+Na]+ Calcd for $C_{25}H_{46}N_4NaO_6^+$ 521.3310; Found 521.3317.

Methyl 2-azido-2-(bis(*tert*-butoxycarbonyl)amino)butanoate 3b

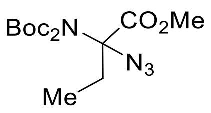

Synthesized following precedure H, starting from *N*Boc$_2$-Dha-*O*Me **1a** (75.3 g, 0.250 mmol) and TBPB (95 µL, 0.50 mmol, 2.0 equiv.) at 0 °C. Purification by column chromatography (SiO_2, pentane:EtOAc = 100:0 to 95:5) afforded methyl 2-azido-2-(bis(*tert*-butoxycarbonyl)amino)butanoate **3b** (82 mg, 0.23 mmol, 92%) as a colorless oil. **R$_f$** (pentane: EtOAc = 96:4) = 0.19. **^{1}H NMR** (400 MHz, $CDCl_3$) δ 3.82 (s, 3H, OC*H$_3$*), 2.14 – 1.92 (m, 2H, C*H$_2$*CH$_3$), 1.51 (s, 18H, 2 x C(C*H$_3$*)$_3$), 0.92 (t, *J* = 7.4 Hz, 3H, CH$_2$C*H$_3$*). **^{13}C NMR** (101 MHz, $CDCl_3$) δ 167.9 (Cq), 151.8 (Cq), 84.3 (Cq), 80.7 (Cq), 53.3 (CH_3), 29.4 (CH_2), 27.9 (CH_3), 8.5 (CH_3). **IR** (ν_{max}, cm^{-1}) 2981 (w), 2947 (w), 2117 (s), 1755 (s), 1720 (s), 1458 (w), 1342 (s), 1246 (s), 1165 (s), 1126 (s). **HRMS** (ESI/QTOF) m/z: $[M+Na]^+$ Calcd for $C_{15}H_{26}N_4NaO_6^+$ 381.1745; Found 381.1743.

Methyl 2-azido-2-(bis(*tert*-butoxycarbonyl)amino)-4-methylpentanoate 3c

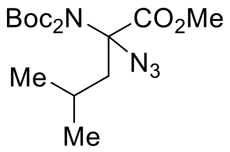

Synthesized following precedure H, starting from *N*Boc$_2$-Dha-*O*Me **1a** (75.3 mg, 0.250 mmol) and diisobutyryl peroxide **2d** (87.1 mg, 0.500 mmol, 2.00 equiv.). Purification by column chromatography (SiO_2, pentane:EtOAc = 100:0 to 96:4) afforded methyl 2-azido-2-(bis(*tert*-butoxycarbonyl)amino)-4-methylpentanoate **3c** (90.6 mg, 0.234 mmol, 94%) as a colorless oil. **R$_f$** (pentane: EtOAc = 95:5) = 0.28. **^{1}H NMR** (400 MHz, $CDCl_3$) δ 3.81 (s, 3H, OC*H$_3$*), 1.97 – 1.81 (m, 2H, C*H$_2$*), 1.72 – 1.62 (m, 1H, C*H*), 1.51 (s, 18H, 2 x C(C*H$_3$*)$_3$), 0.99 (d, *J* = 6.7 Hz, 3H, C*H$_3$*), 0.87 (d, *J* = 6.7 Hz, 3H , C*H$_3$*). **^{13}C NMR** (101 MHz, $CDCl_3$) δ 167.9, 151.7, 84.2, 80.0, 53.2, 44.0, 27.9, 24.4, 24.0, 23.5. **IR** (vmax, cm^{-1}) 2980 (m), 2939 (w), 2878 (w), 2125 (s), 1753 (s), 1717 (s), 1370 (m), 1339 (m), 1249 (s), 1161 (s), 1128 (s). **HRMS** (ESI/QTOF) m/z: $[M+Na]^+$ Calcd for $C_{17}H_{30}N_4NaO_6^+$ 409.2058; Found 409.2051.

Methyl 2-azido-2-((*tert*-butoxycarbonyl)amino)tetradecanoate 3d

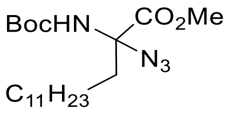

Synthesized following precedure F, starting from *NH*Boc-Dha-*O*Me **1b** (50.3 mg, 0.250 mmol) and LPO (199 mg, 0.500 mmol, 2.00 equiv.). Purification by column chromatography (SiO_2, pentane:EtOAc = 100:0 to 96:4) afforded methyl 2-azido-2-((*tert*-butoxycarbonyl)amino)tetradecanoate **3d** (81.2 mg, 0.0880 mmol, 81%) as a colorless oil. **R$_f$** (pentane: EtOAc = 96:4) = 0.28. **^{1}H NMR** (400 MHz, $CDCl_3$) δ 5.48 (br, 1H, N*H*), 3.84 (s, 3H, OC*H$_3$*), 2.16 – 1.94 (m, 1H, C*H$_a$*), 1.85 – 1.71 (m, 1H, C*H$_b$*), 1.46 (s, 9H, C(C*H$_3$*)$_3$), 1.35 – 1.19 (m, 20H, 10 x C*H$_2$*), 0.88 (t, *J* = 6.9 Hz, 3H, CH$_2$C*H$_3$*). **^{13}C NMR** (101 MHz, $CDCl_3$) δ 169.3, 153.9, 81.5, 77.0, 53.5, 32.1, 29.8, 29.7, 29.6, 29.5, 29.4, 29.2, 28.2, 23.5, 22.8, 14.3. (2C not resolved). **IR** (vmax, cm^{-1}) 3371 (w), 2954 (m), 2924 (s), 2856 (s), 2118 (s), 1752 (s), 1713 (s), 1457 (m), 1368

(m), 1249 (m), 1159 (s). **HRMS** (ESI/QTOF) m/z: $[M+Na]^+$ Calcd for $C_{20}H_{38}N_4NaO_4^+$ 421.2785; Found 421.2778.

Methyl 2-azido-2-((*tert*-butoxycarbonyl)amino)butanoate 3e

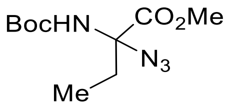

Synthesized following precedure H, starting from *NH*Boc-Dha-*O*Me **1b** (369 mg, 1.50 mmol) and TBPB (577 µL, 3.00 mmol, 2.0 equiv.) at 0 °C. Purification by column chromatography (1/3 Al_2O_3, 2/3 SiO_2, pentane:EtOAc = 100:0 to 95:5) afforded methyl 2-azido-2-((*tert*-butoxycarbonyl)amino)butanoate **3e** (347 mg, 1.15 mmol, 76%) as a colorless oil. $\mathbf{R_f}$ (pentane: EtOAc = 96:4) = 0.16. **^{1}H NMR** (400 MHz, $CDCl_3$) δ 5.50 (s, 1H, N*H*), 3.85 (s, 3H, OC*H₃*), 2.12 (s, 1H, C*H*$_a$), 1.91 – 1.76 (m, 1H, C*H*$_b$), 1.45 (s, 9H, $C(CH_3)_3$), 0.91 (t, *J* = 7.5 Hz, 3H, C*H*$_3$). **^{13}C NMR** (101 MHz, $CDCl_3$) δ 169.2, 153.9, 81.5, 77.3, 53.5, 30.2, 28.2, 8.1. **IR** (vmax, cm^{-1}) 3250 (w), 3155 (w), 2980 (m), 2111 (s), 1755 (s), 1709 (s), 1497 (w), 1456 (m), 1435 (m), 1384 (m), 1367 (s), 1242 (s), 1156 (s). **HRMS** (ESI/QTOF) m/z: $[M+Na]^+$ Calcd for $C_{10}H_{18}N_4NaO_4^+$ 281.1220; Found 281.1220.

Methyl 2-((((9H-fluoren-9-yl)methoxy)carbonyl)amino)-2-azidotetradecanoate 3f

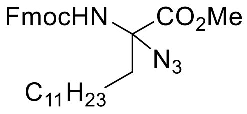

Synthesized following precedure H, starting from *N*HFmoc-Dha-*O*Me **1c** (80.8 mg, 0.250 mmol) and LPO (199 mg, 0.500 mmol, 2.00 equiv.). Purification by column chromatography (SiO_2, pentane:EtOAc = 95:5 to 9:1) afforded methyl 2-((((9H-fluoren-9-yl)methoxy)carbonyl)amino)-2-azidotetradecanoate **3f** (113 mg, 0.217 mmol, 87%) as a colorless oil. $\mathbf{R_f}$ (pentane: EtOAc = 9:1) = 0.42. **^{1}H NMR** (400 MHz, $CDCl_3$) δ 7.77 (d, *J* = 7.5 Hz, 2H, 2 x Ar*H*), 7.59 (d, *J* = 7.4 Hz, 2H, 2 x Ar*H*), 7.41 (t, *J* = 7.5 Hz, 2H, 2 x Ar*H*), 7.32 (td, *J* = 7.5, 1.3 Hz, 2H, 2 x Ar*H*), 5.80 (s, 1H, N*H*), 4.47 (d, *J* = 7.0 Hz, 2H, CH_{2Fmoc}), 4.24 (t, *J* = 6.8 Hz, 1H, CH_{Fmoc}), 3.85 (s, 3H, OCH_3), 2.22 (br, 1H, C*H*$_a$), 1.86 (br, *J* = 6.4 Hz, 1H, C*H*$_b$), 1.33 – 1.18 (m, 20H, $C_{10}H_{20}$), 0.92 – 0.84 (t, 3H, CH_3). **^{13}C NMR** (101 MHz, $CDCl_3$) δ 169.1, 154.6, 143.75, 143.66, 141.5, 128.0, 127.3, 125.1, 120.2, 67.4, 53.8, 47.2, 36.0, 32.1, 29.9, 29.8, 29.7, 29.6, 29.5, 29.4, 29.2, 23.7, 22.8, 14.3. (1C not resolved). **IR** (vmax, cm^{-1}) 2923 (s), 2853 (s), 2117 (m), 1739 (m), 1709 (s), 1523 (m), 1455 (m), 1242 (m). **HRMS** (ESI/QTOF) m/z: $[M+Na]^+$ Calcd for $C_{30}H_40N_4NaO_4^+$ 543.2942; Found 543.2938.

Methyl 2-acetamido-2-azidotetradecanoate 3g

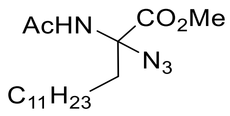

Synthesized following precedure G, starting from *N*HAc-Dha-*O*Me (35.8 mg, 0.250 mmol) and LPO (199 mg, 0.500 mmol, 2.00 equiv.). Purification by column chromatography (SiO_2, pentane:EtOAc = 8:2 to 6:4) afforded methyl 2-acetamido-2-azidotetradecanoate **3g** (58.5

mg, 0.172 mmol, 69%) as a amorphous white solid. **R$_f$** (pentane: EtOAc = 7:3) = 0.30. **^{1}H NMR** (400 MHz, $CDCl_3$) δ 6.34 (s, 1H, N*H*), 3.85 (s, 3H, OC*H$_3$*), 2.20 – 2.10 (m, 1H, C*H$_a$*), 2.06 (s, 3H, CH_{3Ac}), 1.86 – 1.75 (m, 1H, C*H$_a$*), 1.31 – 1.20 (m, 20H, $C_{10}H_{20}$), 0.93 – 0.82 (m, 3H, C*H$_3$*). **^{13}C NMR** (101 MHz, $CDCl_3$) δ 170.12, 169.2, 76.3, 53.7, 36.4, 32.1, 29.8, 29.7, 29.6, 29.5, 29.44, 29.3, 23.6, 23.2, 22.8, 14.3. (1C not resolved). **IR** (vmax, cm^{-1}) 3295 (w), 3055 (w), 2980 (w), 2943 (w), 2116 (s), 1746 (s), 1660 (s), 1533 (s), 1440 (m), 1372 (m), 1239 (s), 1134 (m). **m.p.** 66-68 °C. **HRMS** (ESI/QTOF) m/z: [M+Na]$^+$ Calcd for $C_{17}H_{32}N_4NaO_3^+$ 363.2367; Found 363.2368.

Methyl 2-acetamido-2-azidobutanoate 3h

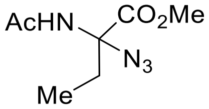

Synthesized following precedure H, starting from *N*HAc-Dha-*O*Me (716 mg, 5.00 mmol) and TBPB (1.94 mL, 10.0 mmol, 2.0 equiv.) at 0 °C. Purification by column chromatography (SiO_2, pentane:EtOAc = 8:2 to 4:6) afforded methyl methyl 2-acetamido-2-azidobutanoate **3h** (518 mg, 2.59 mmol, 52%) as a white solid. **R$_f$** (pentane: EtOAc = 96:4) = 0.19. **^{1}H NMR** (400 MHz, $CDCl_3$) δ 6.30 (s, 1H, N*H*), 3.86 (s, 3H, OC*H$_3$*), 2.35 – 2.15 (m, 1H, C*H$_a$*), 2.07 (s, 3H, CH_{3Ac}), 1.92 – 1.80 (m, 1H, C*H$_b$*), 0.92 (t, *J* = 7.4 Hz, 3H, CH_2CH_3). **^{13}C NMR** (101 MHz, $CDCl_3$) δ 170.2, 169.1, 76.7, 53.7, 29.7, 23.2, 8.1. **IR** (vmax, cm^{-1}) 3285 (w), 2117 (s), 1743 (m), 1663 (s), 1534 (m), 1239 (s), 1134 (m), 998 (m). **m.p**. 70-72°C. **HRMS** (ESI/QTOF) m/z: [M+Na]$^+$ Calcd for $C_7H_{12}N_4NaO_3^+$ 223.0802; Found 223.0804.

Methyl 2-acetamido-2-azido-4-methylpentanoate 3i

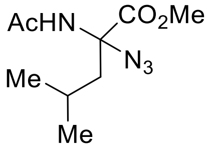

Synthesized following precedure H, starting from *N*HAc-Dha-*O*Me (35.8 mg, 0.250 mmol) and diisobutyryl peroxide **2d** (87.1 mg, 0.500 mmol, 2.00 equiv.). Purification by column chromatography (SiO_2, pentane:EtOAc = 8:2 to 1:1) afforded methyl 2-acetamido-2-azido-4-methylpentanoate **3i** (25.9 mg, 0.114 mmol, 45%) as a white solid. **R$_f$** (pentane: EtOAc = 6:4) = 0.29. **^{1}H NMR** (400 MHz, $CDCl_3$) δ 6.40 (s, 1H, N*H*), 3.85 (s, 3H, OC*H$_3$*), 2.24 (dd, *J* = 13.6, 6.0 Hz, 1H, C*H$_a$*), 2.06 (s, 3H, CH_{3Ac}), 1.78 (dd, *J* = 13.7, 6.4 Hz, 1H, C*H$_b$*), 1.71 (dt, *J* = 12.9, 6.5 Hz, 1H, C*H*), 0.93 (d, *J* = 2.3 Hz, 3H, C*H$_3$*), 0.91 (d, *J* = 2.3 Hz, 3H, C*H$_3$*).**^{13}C NMR** (101 MHz, $CDCl_3$) δ 170.1, 169.5, 75.9, 53.6, 44.0, 24.4, 23.4, 23.3. **IR** (vmax, cm^{-1}) 3279 (w), 2961 (m), 2121 (s), 1753 (s), 1656 (m), 1541 (m), 1244 (s), 1231 (s), 1152 (m), 1025 (m). **m.p.** 83-85 °C. **HRMS** (ESI/QTOF) m/z: [M+Na]$^+$ Calcd for $C_9H_{16}N_4NaO_3^+$ 251.1115; Found 251.1115.

***tert*-Butyl 2-azido-2-(bis(*tert*-butoxycarbonyl)amino)tetradecanoate 3j**

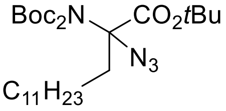

Synthesized following precedure H, starting from *N*Boc$_2$-Dha-*Ot*Bu **1e** (85.8 mg, 0.250 mmol) and LPO (199 mg, 0.500 mmol, 2.00 equiv.). Purification by column chromatography (SiO$_2$, pentane:EtOAc = 100:0 to 98:2) afforded *tert*-Butyl 2-azido-2-(bis(*tert*-butoxycarbonyl)amino)tetradecanoate **3j** (109 mg, 0.202 mmol, 81%) as a colorless oil. **R$_f$** (pentane: EtOAc = 98:2) = 0.35. **^{1}H NMR** (400 MHz, CDCl$_3$) δ 1.97 – 1.82 (m, 2H, C*CH$_2$*), 1.64 – 1.37 (m, 28H, CH$_3$C$_9$*H$_{18}$*C*H$_a$* and , C(C*H$_3$*)$_3$), 1.24 (s, 19H, C*H$_b$* and 2 x , C(C*H$_3$*)$_3$), 0.88 (t, *J* = 6.9 Hz, 3H, C*H$_3$*). **^{13}C NMR** (101 MHz, CDCl$_3$) δ 165.9, 151.9, 83.6, 83.2, 80.2, 36.0, 32.1, 29.78, 29.7, 29.56, 29.51, 29.49, 29.4, 27.94, 27.89, 23.6, 22.8, 14.3. (1C not resolved). **IR** (vmax, cm^{-1}) 2980 (m), 2928 (m), 2855 (m), 2124 (m), 1753 (s), 1718 (s), 1457 (m), 1394 (m), 1369 (s), 1344 (s), 1250 (s), 1152 (s). **HRMS** (ESI/QTOF) m/z: [M+Na]+ Calcd for $C_{28}H_{52}N_4NaO_6^+$ 563.3779; Found 563.3781.

***tert*-Butyl 2-azido-2-(bis(*tert*-butoxycarbonyl)amino)-4-methylpentanoate 3k**

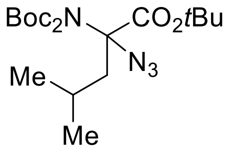

Synthesized following precedure H, starting from *N*Boc$_2$-Dha-*Ot*Bu **1e** (85.5 mg, 0.250 mmol) and diisobutyryl peroxide **2d** (87.1 mg, 0.500 mmol, 2.00 equiv.). Purification by column chromatography (SiO$_2$, pentane:EtOAc = 100:0 to 96:4) afforded *tert*-Butyl 2-azido-2-(bis(*tert*-butoxycarbonyl)amino)-4-methylpentanoate **3k** (67.1 mg, 0.157 mmol, 63%) as a colorless oil. **R$_f$** (pentane: EtOAc = 95:5) = 0.61. **^{1}H NMR** (400 MHz, CDCl$_3$) δ 1.84 (t, 2H, C*H$_2$*), 1.82 – 1.71 (m, 1H, C*H*), 1.50 (s, 27H, 3 x C(C*H$_3$*)$_3$), 1.01 (d, *J* = 6.5 Hz, 3H, C*H$_3$*), 0.92 (d, *J* = 6.7 Hz, 3H, C*H$_3$*). **^{13}C NMR** (101 MHz, CDCl$_3$) δ 165.9, 151.8, 83.6, 83.4, 80.2, 43.7, 28.0, 27.9, 24.6, 24.3, 23.3. **IR** (vmax, cm^{-1}) 2979 (m), 2932 (w), 2124 (m), 1752 (s), 1717 (s), 1369 (m), 1339 (m), 1249 (s), 1153 (s), 1133 (s). **HRMS** (ESI/QTOF) m/z: [M+Na]$^+$ Calcd for $C_{20}H_{36}N_4NaO_6^+$ 451.2527; Found 451.2521.

Benzyl 2-azido-2-(bis(tert-butoxycarbonyl)amino)tetradecanoate 3l

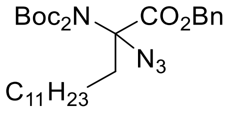

Synthesized following precedure F, starting from *N*Boc$_2$-Dha-*O*Bn **1f** (98 mg, 0.26 mmol) and LPO (199 mg, 0.500 mmol, 2.0 equiv.). Purification by column chromatography (SiO$_2$, pentane:EtOAc = 100:0 to 96:4) afforded benzyl 2-azido-2-(bis(*tert*-butoxycarbonyl) amino) tetradecanoate **3l** (118 mg, 0.256 mmol, 79%) as an amorphous solid. **R$_f$** (pentane: EtOAc = 96:4) = 0.38. **^{1}H NMR** (400 MHz, CDCl$_3$) δ 7.41 – 7.30 (m, 5H, 5 x Ar*H*), 5.31 – 5.18 (m, 2H, C*H$_2$*Ar), 1.92 (qdd, *J* = 13.2, 11.4, 4.8 Hz, 2H, C*H$_2$*CH$_2$), 1.48 (s, 18H, 2 x C(C*H$_3$*)$_3$), 1.42 – 1.33 (m, 1H, CH$_2$C*H$_a$*), 1.35 – 1.09 (m, 18H, C$_9$*H$_{18}$*), 1.06 – 0.93 (m, 1H, CH$_2$C*H$_b$*), 0.92 – 0.84 (m, 3H, CH$_2$C*H$_3$*). **^{13}C NMR** (101 MHz, CDCl$_3$) δ 167.3, 151.8, 135.3, 128.6, 128.6, 128.5, 84.2, 80.0, 68.1, 36.0, 32.1, 29.78, 29.76, 29.7, 29.53, 29.50, 29.4, 29.3, 27.8, 23.7, 22.8, 14.3. **IR** (vmax, cm^{-1}) 2986 (m), 2928 (m), 2849 (m), 2125 (s), 1761 (s), 1717 (s), 1343 (s), 1253 (s), 1127 (s). **HRMS** (ESI/QTOF) m/z: [M+Na]$^+$ Calcd for $C_{31}H_{50}N_4NaO_6^+$ 597.3623; Found 597.3622.

Benzyl 2-azido-2-(bis(*tert*-butoxycarbonyl)amino)butanoate 3m

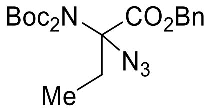

Synthesized following precedure H, starting from *N*Boc$_2$-Dha-*O*Bn **1f** (94.4 mg, 0.250 mmol) and TBPB (95.1 µL, 0.500 mmol, 2.0 equiv.) at 0 °C. Purification by column chromatography (SiO_2, pentane:EtOAc = 100:0 to 96:4) afforded benzyl 2-azido-2-(bis(*tert*-butoxycarbonyl) amino)butanoate **3m** (93.6 mg, 0.215 mmol, 86%) as a colorless oil. **R$_f$** (pentane: EtOAc = 96:4) = 0.44. **^{1}H NMR** (400 MHz, $CDCl_3$) δ 7.43 – 7.30 (m, 5H, 5 x Ar*H*), 5.25 (s, 2H, C*H$_2$*Ar), 2.12 – 1.90 (m, 2H, C*H$_2$*CH$_3$*)*, 1.48 (s, 18H, 2 x C(C*H$_3$*)$_3$), 0.85 (t, *J* = 7.4 Hz, 3H, CH$_2$C*H$_3$*). **^{13}C NMR** (101 MHz, $CDCl_3$) δ 167.2, 151.8, 135.3, 128.6, 128.5, 84.2, 80.6, 68.1, 29.3, 27.8, 8.4. (1C not resolved). **IR** (νmax, cm^{-1}) 2979 (m), 2929 (m), 2856 (m), 2125 (s), 1758 (s), 1721 (s), 1339 (s), 1253 (s), 1127 (s). m.p. 45-47 °C. **HRMS** (ESI/QTOF) m/z: $[M+Na]^+$ Calcd for $C_{21}H_{30}N_4NaO_6^+$ 457.2058; Found 457.2065.

Methyl 2-azido-2-(bis(*tert*-butoxycarbonyl)amino)-4-methylpentanoate 3n

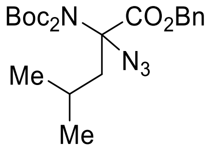

Synthesized following precedure G, starting from *N*Boc$_2$-Dha-*O*Bn **1f** (94.4 mg, 0.250 mmol) and diisobutyryl peroxide **2d** (87.1 mg, 0.500 mmol, 2.0 equiv.). Purification by column chromatography (SiO_2, pentane:EtOAc = 100:0 to 96:4) afforded benzyl 2-azido-2-(bis(*tert*-butoxycarbonyl) amino))-4-methylpentanoate **3n** (75.2 mg, 0.187 mmol, 65%) as a amorphous solid. **R$_f$** (pentane: EtOAc = 95:5) = 0.41. **^{1}H NMR** (400 MHz, $CDCl_3$) δ 7.42 – 7.27 (m, 5H, 5 x Ar*H*), 5.28 – 5.17 (m, 2H, C*H$_2$*Ar), 1.96 – 1.79 (m, 2H, C*H$_2$*CH), 1.66 – 1.59 (m, 1H, CH$_2$C*H*), 1.48 (s, 18H, 2 x C(C*H$_3$*)$_3$), 0.96 (d, *J* = 6.7 Hz, 3H, C*H$_3$*), 0.79 (d, *J* = 6.7 Hz, 3H, C*H$_3$*). **^{13}C NMR** (101 MHz, $CDCl_3$) δ 167.3, 151.7, 135.2, 128.6, 128.5, 128.5, 84.1, 79.9, 68.0, 43.8, 27.8, 24.3, 24.0, 23.4. **IR** (νmax, cm^{-1}) 2980 (m), 2124 (s), 1761 (s), 1714 (s), 1370 (s), 1343 (s), 1253 (s), 1127 (s). **HRMS** (ESI/QTOF) m/z: $[M+Na]^+$ Calcd for $C_{23}H_{34}N_4NaO_6^+$ 485.2371; Found 485.2381.

Benzyl 2-azido-2-((tert-butoxycarbonyl)amino)butanoate 3o

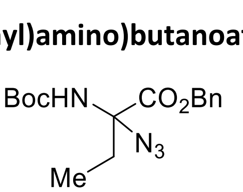

Synthesized following precedure G, starting from *N*HBoc$_2$-Dha-*O*Bn **1g** (69.3 g, 0.250 mmol) and TBPB (95.1 µL, 0.500 mmol, 2.0 equiv.) at 0 °C. Purification by column chromatography (1/3 Al_2O_3, 2/3 SiO_2, pentane:EtOAc = 100:0 to 95:5) afforded benzyl 2-azido-2-((*tert*-butoxycarbonyl) amino)butanoate **3o** (66.1, 0.198 mmol, 79%) as an amorphous solid. **R$_f$** (pentane: EtOAc = 92:8) = 0.43. **^{1}H NMR** (400 MHz, $CDCl_3$) δ 7.42 – 7.29 (m, 5H, 5 x Ar*H*), 5.50 (s, 1H, N*H*), 5.34 – 5.11 (m, 2H, C*H$_{2Bn}$*), 2.24 – 1.98 (m, 1H, C*H$_a$*), 1.92 – 1.76 (m, 1H, C*H$_b$*), 1.43

(s, 9H, C(CH_3)$_3$), 0.86 (t, J = 7.4 Hz, 3H, CH_3). **^{13}C NMR** (101 MHz, $CDCl_3$) δ 168.5, 153.9, 135.0, 128.73, 128.68, 81.4, 77.5, 68.4, 30.2, 28.2, 7.9 (1C not resolved). **IR** (vmax, cm^{-1}) 3364 (w), 2979 (m), 2117 (s), 1721 (s), 1717 (s), 1716 (s), 1507 (s), 1369 (m), 1249 (s), 1155 (s). **HRMS** (ESI/QTOF) m/z: $[M+Na]^+$ Calcd for. $C_{16}H_{22}N_4NaO_4^+$ 357.1533; Found 357.1533.

Methyl 2-azido-2-(bis(*tert*-butoxycarbonyl)amino)pentanoate 3p

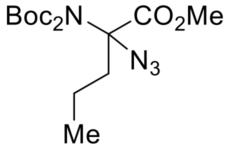

Synthesized following precedure G, starting from *N*Boc$_2$-Dha-*O*Me **1a** (75.3 mg, 0.250 mmol) and diipropionyl peroxide **2e** (73.1 mg, 0.500 mmol, 2.0 equiv.). Purification by column chromatography (SiO_2, pentane:EtOAc = 100:0 to 96:4) afforded methyl 2-azido-2-(bis(*tert*-butoxycarbonyl)amino)pentanoate **3p** (88.4 mg, 0.237 mmol, 95%) as a colorless oil. **R_f** (pentane: EtOAc = 95:5) = 0.36. **^{1}H NMR** (400 MHz, $CDCl_3$) δ 3.81 (s, 3H, OCH_3), 2.02 – 1.86 (m, 2H, CH_2), 1.51 (s, 18H, 2 x C(CH_3)$_3$), 1.49 – 1.41 (m, 1H, CH_a), 1.23 – 1.08 (m, 1H, CH_b), 0.90 (t, *J* = 7.3 Hz, 3H, CH_3). **^{13}C NMR** (101 MHz, $CDCl_3$) δ 168.0, 151.8, 84.3, 80.1, 53.3, 38.1, 27.9, 17.4, 13.9. **IR** (vmax, cm^{-1}) 2981 (m), 2124 (s), 1757 (s), 1714 (s), 1335 (m), 1250 (s), 1130 (s). **HRMS** (ESI/QTOF) m/z: $[M+Na]^+$ Calcd for $C_{16}H_{28}N_4NaO_6^+$ 395.1901; Found 395.1903.

Methyl 2-azido-2-(bis(*tert*-butoxycarbonyl)amino)-4,4-dimethylpentanoate 3q

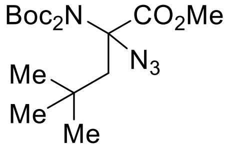

Synthesized following precedure G, starting from *N*Boc$_2$-Dha-*O*Me **1a** (75.3 mg, 0.250 mmol) and *tert*-butyl peroxypivalate **2o** (87.1 mg, 0.500 mmol, 2.0 equiv.) for 6 h. Purification by column chromatography (SiO_2, pentane:EtOAc = 98:2 to 95:5) afforded methyl 2-azido-2-(bis(*tert*-butoxycarbonyl)amino)-4,4-dimethylpentanoate **3q** (50.2 mg, 0.125 mmol, 50%) as a white solid. **R_f** (pentane: EtOAc = 95:5) = 0.53. **^{1}H NMR** (400 MHz, $CDCl_3$) δ 3.80 (s, 3H, OCH_3), 1.96 (q, *J* = 13.9 Hz, 2H, CH_2), 1.50 (s, 18H, 2 x C(CH_3)$_3$), 0.97 (s, 9H, C(CH_3)$_3$). **^{13}C NMR** (101 MHz, $CDCl_3$) δ 168.1, 151.6, 84.2, 78.9, 53.0, 47.2, 30.9, 30.1, 27.8. **IR** (vmax, cm^{-1}) 2989 (m), 2911 (w), 2125 (s), 1757 (s), 1719 (s), 1370 (s), 1340 (s), 1249 (s), 1126 (s). **m.p.** 42-43 °C. **HRMS** (ESI/QTOF) m/z: $[M+Na]^+$ Calcd for $C_{18}H_{32}N_4NaO_6^+$ 423.2214; Found 423.2213.

Methyl 2-azido-2-(bis(*tert*-butoxycarbonyl)amino)-5,5-dimethylhexanoate 3r

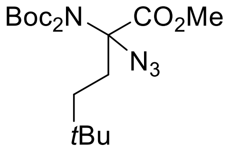

Synthesized following precedure F, starting from *N*Boc$_2$-Dha-*O*Me **1a** (75.3 mg, 0.250 mmol) and dineopentyl peroxide **2f** (115 mg, 0.500 mmol, 2.0 equiv.). Purification by column chromatography (SiO_2, pentane:EtOAc = 100:0 to 96:4) afforded methyl 2-azido-2-(bis(*tert*-

butoxycarbonyl)amino)-5,5-dimethylhexanoate **3r** (88.8 mg, 0.214 mmol, 86%) as a colorless oil. **R$_f$** (pentane: EtOAc = 96:4) = 0.27. **^{1}H NMR** (400 MHz, $CDCl_3$) δ 3.82 (s, 3H, OC*H$_3$*), 2.05 – 1.84 (m, 2H, C*H$_2$*CH_2CH_3), 1.51 (s, 18H, 2 x C(C*H$_3$*)$_3$), 1.41 – 1.31 (m, 1H, CH_2C*H$_a$*CH_3), 1.02 – 0.91 (m, 1H, CH_2C*H$_b$*CH_3), 0.85 (s, 9H, C(C*H$_3$*)$_3$). **^{13}C NMR** (101 MHz, $CDCl_3$) δ 167.9, 151.7, 84.2, 80.2, 53.3, 37.1, 31.5, 30.1, 29.2, 27.8. **IR** (vmax, cm^{-1}) 2982 (m), 2961 (m), 2117 (s), 1757 (s), 1718 (s), 1369 (m), 1343 (s), 1242 (s), 1173 (s), 1123 (s). m.p. 40-42 °C. **HRMS** (nanochip-ESI/LTQ-Orbitrap) m/z: [M+Na]$^+$ Calcd for $C_{19}H_{34}N_4NaO_6^+$ 437.2371; Found 437.2366.

Methyl 2-azido-2-(bis(*tert*-butoxycarbonyl)amino)tridec-12-enoate 3s

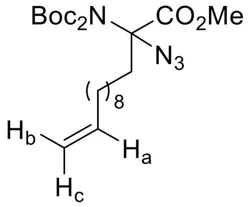

Synthesized following precedure F, starting from *N*Boc$_2$-Dha-*O*Me **1a** (75.3 mg, 0.250 mmol) and (A) di(undec-10-enoyl) peroxide **2g** (183 mg, 0.500 mmol, 2.0 equiv.) (B) *tert*-Butyl undec-10-enoyl peroxide **2p** (128 mg, 0.500 mmol, 2.0 equiv.) for 2 h. Purification by column chromatography (SiO_2, pentane:EtOAc = 100:0 to 96:4) afforded methyl 2-azido-2-(bis(*tert*-butoxycarbonyl)amino)tridec-12-enoate **3s** (A, 101 mg, 0.209 mmol, 84%)(B, 11.3 mg, 0.0234 mmol, 9%) as a colorless oil. **R$_f$** (pentane: EtOAc = 96:4) = 0.34. **^{1}H NMR** (400 MHz, $CDCl_3$) δ 5.80 (ddt, *J* = 16.9, 10.2, 6.7 Hz, 1H, *H$_a$*), 4.98 (ddt, *J* = 17.1, 2.1, 1.6 Hz, 1H, *H$_b$*), 4.92 (ddt, *J* = 10.2, 2.4, 1.2 Hz, 1H, *H$_c$*), 3.82 (s, 3H, OC*H$_3$*), 2.06 – 1.99 (m, 2H, CC*H$_2$*), 1.99 – 1.88 (m, 2H, C_{sp2}C*H$_2$*), 1.51 (s, 18H, 2 x C(C*H$_3$*)$_3$), 1.45 (dd, *J* = 7.3, 3.3 Hz, 1H, C*H$_{a'}$*), 1.35 (d, *J* = 4.6 Hz, 2H, C*H$_2$*), 1.25 (d, *J* = 4.6 Hz, 10H, 5 x C*H$_2$*), 1.17 – 1.05 (m, 1H, C*H$_{b'}$*). **^{13}C NMR** (101 MHz, $CDCl_3$) δ 168.0, 151.8, 139.4, 114.3, 84.3, 80.1, 53.3, 36.1, 33.9, 29.52, 29.49, 29.42, 29.36, 29.2, 29.1, 27.9, 23.9. **IR** (vmax, cm^{-1}) 3079 (w), 2982 (m), 2932 (m), 2856 (m), 2118 (s), 1758 (s), 1718 (s), 1335 (m), 1249 (s), 1162 (s), 1127 (s). **HRMS** (ESI/QTOF) m/z: [M+Na]$^+$ Calcd for $C_{24}H_{42}N_4NaO_6^+$ 505.2997; Found 505.2997.

Methyl 2-azido-2-(bis(*tert*-butoxycarbonyl)amino)hept-6-ynoate 3t

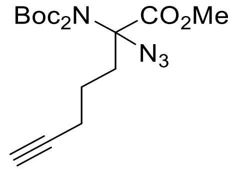

Synthesized following precedure F, starting from *N*Boc$_2$-Dha-*O*Me **1a** (75.3 mg, 0.250 mmol) and di(4-pentynoyl) peroxide **2h** (97 mg, 0.50 mmol, 2.0 equiv.) for 6 h. Purification by column chromatography (SiO_2, pentane:EtOAc = 97:3 to 95:5) afforded methyl 2-azido-2-(bis(*tert*-butoxycarbonyl)amino)hept-6-ynoate **3t** (54 mg, 0.14 mmol, 54%) as a colorless oil. R$_f$ (pentane: EtOAc = 94:6) = 0.28. **^{1}H NMR** (400 MHz, $CDCl_3$) δ 3.82 (s, 3H, OC*H$_3$*), 2.39 – 2.31 (m, 1H, C*H$_2$*), 2.19 (td, *J* = 7.0, 2.6 Hz, 2H, C*H$_2$*), 2.10 (ddd, *J* = 16.9, 11.5, 4.8 Hz, 1H, C*H$_2$*), 1.95 (t, *J* = 2.6 Hz, 1H, C_{sp}*H*), 1.75 – 1.64 (m, 2H, C*H$_2$*), 1.51 (s, 18H, 2 x C(C*H$_3$*)$_3$). **^{13}C NMR** (101 MHz, $CDCl_3$) δ 167.8, 151.8, 84.4, 83.1, 79.9, 69.4, 53.4, 35.2, 27.8, 22.9, 18.3. **IR** (vmax, cm^{-1}) 3285

(w), 2986 (m), 2122 (s), 1755 (s), 1717 (s), 1372 (m), 1340 (s), 1247 (s), 1123 (s). **HRMS** (ESI/QTOF) m/z: $[M+Na]^+$ Calcd for $C_{18}H_{28}N_4NaO_6^+$ 419.1901; Found 419.1901.

Methyl 2-azido-2-(bis(*tert*-butoxycarbonyl)amino)oct-7-ynoate 3u

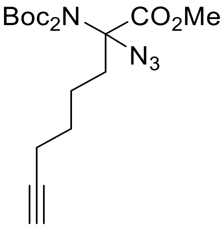

Synthesized following precedure F, starting from *N*Boc₂-Dha-*O*Me **1a** (75.3 mg, 0.250 mmol) and di(4-pentynoyl) peroxide **2i** (111 mg, 0.500 mmol, 2.0 equiv.) for 6 h. Purification by column chromatography (SiO_2, pentane:EtOAc = 100:0 to 96:4) afforded methyl 2-azido-2-(bis(tert-butoxycarbonyl)amino)oct-7-ynoate **3u** (68.8 mg, 0.168 mmol, 67%) as a colorless oil. $\mathbf{R_f}$ (pentane: EtOAc = 96:4) = 0.30. **^{1}H NMR** (400 MHz, $CDCl_3$) δ 3.83 (s, 3H, OCH_3), 2.17 (td, *J* = 7.0, 2.6 Hz, 2H, CH_2), 2.03 – 1.95 (m, 2H, CH_2), 1.93 (t, *J* = 2.6 Hz, 1H, $C_{sp}H$), 1.51 (s, 22H, 2 x C(CH_3)$_3$ and 2 x CH_2). **^{13}C NMR** (101 MHz, $CDCl_3$) δ 167.9, 151.8, 84.3, 83.9, 80.1, 68.8, 53.3, 35.6, 28.2, 27.9, 23.1, 18.3. **IR** (vmax, cm^{-1}) 3296 (w), 2986 (w), 2874 (w), 2117 (m), 1753 (m), 1716 (s), 1341 (m), 1235 (s), 1123 (s). **HRMS** (ESI/QTOF) m/z: $[M+Na]^+$ Calcd for $C_{19}H_{30}N_4NaO_6^+$ 433.2058; Found 433.2055.

Methyl 2-azido-2-(bis(*tert*-butoxycarbonyl)amino)-7-chloroheptanoate 3v

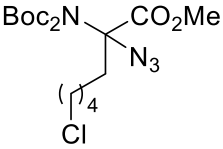

Synthesized following precedure F, starting from *N*Boc₂-Dha-*O*Me **1a** (75.3 mg, 0.250 mmol) and 5-chloropentanoyl peroxide **2j** (136 mg, 0.500 mmol, 2.0 equiv.) for 4 h. Purification by column chromatography (SiO_2, pentane:EtOAc = 100:0 to 96:4) afforded methyl 2-azido-2-(bis(*tert*-butoxycarbonyl)amino)-7-chloroheptanoate **3v** (99.0 mg, 0.228 mmol, 91%) as a colorless oil. $\mathbf{R_f}$ (pentane: EtOAc = 96:4) = 0.19. **^{1}H NMR** (400 MHz, $CDCl_3$) δ 3.82 (s, 3H, OCH_3), 3.51 (t, *J* = 6.6 Hz, 2H, CH_2Cl), 2.06 – 1.86 (m, 2H, CH_2), 1.79 – 1.70 (m, 2H, CH_2), 1.51 (s, 19H, 2 x C(CH_3)$_3$ and CH_a), 1.48 – 1.36 (m, 2H, CH_2), 1.21 – 1.10 (m, 1H, CH_b). **^{13}C NMR** (101 MHz, $CDCl_3$) δ 167.9, 151.8, 84.4, 80.1, 53.4, 44.9, 35.9, 32.3, 27.9, 26.7, 23.3. **IR** (vmax, cm^{-1}) 2981 (m), 2944 (w), 2118 (s), 1761 (s), 1718 (s), 1370 (m), 1343 (s), 1251 (s), 1162 (s), 1126 (s). **HRMS** (ESI/QTOF) m/z: $[M+Na]^+$ Calcd for $C_{18}H_{31}ClN_4NaO_6^+$ 457.1824; Found 457.1834.

Methyl 2-azido-2-(bis(*tert*-butoxycarbonyl)amino)-4,4,4-trifluorobutanoate 3w

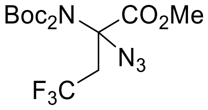

Synthesized following precedure F, starting from *N*Boc₂-Dha-*O*Me **1a** (75.3 mg, 0.250 mmol) and Togni II (158 mg, 0.500 mmol, 2.0 equiv.) for 2 h at 60 °C. Purification by column

chromatography (SiO_2, pentane:EtOAc = 100:0 to 96:4) afforded methyl 2-azido-2-(bis(*tert*-butoxycarbonyl)amino)-4,4,4-trifluorobutanoate **3w** (53.9 mg, 0.131 mmol, 52%) as a orange oil. **R_f** (pentane: EtOAc = 96:4) = 0.36. **^{1}H NMR** (400 MHz, $CDCl_3$) δ 3.86 (s, 3H, OCH_3), 3.20 (dq, J = 15.2, 10.0 Hz, 1H, CH_a), 2.94 (dq, J = 15.3, 10.0 Hz, 1H, CH_b), 1.51 (s, 18H, 2 x $C(CH_3)_3$). **^{19}F NMR** (376 MHz, $CDCl_3$) δ -61.4. **^{13}C NMR** (101 MHz, $CDCl_3$) δ 166.2, 151.7, 124.0 (q, J = 277.7 Hz), 84.9, 76.6 (d, J = 2.6 Hz), 53.9, 39.9 (q, J = 29.7 Hz), 27.8. **IR** (vmax, cm^{-1}) 2984 (w), 2129 (m), 1757 (s), 1722 (s), 1372 (m), 1254 (m), 1152 (s), 1130 (s). **HRMS** (ESI/QTOF) m/z: $[M+Na]^+$ Calcd for $C_{15}H_{23}F_3N_4NaO_6^+$ 435.1462; Found 435.1461.

Methyl 2-azido-2-(bis(*tert*-butoxycarbonyl)amino)-3-cyclopropylpropanoate 3x

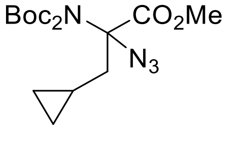

Synthesized following precedure F, starting from *N*Boc$_2$-Dha-*O*Me **1a** (75.3 mg, 0.250 mmol) and di(cyclopropanecarboxyl) peroxide **2k** (85.1 mg, 0.500 mmol, 2.0 equiv.) for 4 h. Purification by column chromatography (SiO_2, pentane:EtOAc = 100:0 to 96:4) afforded methyl 2-azido-2-(bis(*tert*-butoxycarbonyl)amino)-3-cyclopropylpropanoate **3x** (73.5 mg, 0.191 mmol, 76%) as a colorless oil. **R_f** (pentane: EtOAc = 96:4) = 0.33. **^{1}H NMR** (400 MHz, $CDCl_3$) δ 3.83 (s, 3H, OCH_3), 2.25 (dd, J = 13.6, 5.0 Hz, 1H, CCH_a), 1.59 (dd, J = 13.6, 8.5 Hz, 1H, CCH_b), 1.51 (s, 18H, 2 x $C(CH_3)_3$), 0.79 (dddd, J = 13.4, 8.4, 5.0, 3.5 Hz, 1Hz, CH_{Cy}), 0.58 – 0.40 (m, 2H, 2 x CH_{Cy}), 0.12 (dtd, J = 9.2, 5.2, 4.1 Hz, 1H, CH_{Cy}), 0.08 – -0.01 (m, 1H, CH_{Cy}). **^{13}C NMR** (101 MHz, $CDCl_3$) δ 167.9, 151.8, 84.3, 80.2, 53.2, 40.9, 27.9, 6.0, 4.9, 4.0. **IR** (vmax, cm^{-1}) 2979 (m), 2932 (w), 2117 (s), 1752 (s), 1717 (s), 1372 (m), 1340 (s), 1246 (s), 1160 (s), 1124 (s). m.p. 56-58 °C. **HRMS** (ESI/QTOF) m/z: $[M+Na]^+$ Calcd for $C_{17}H_{28}N_4NaO_6^+$ 407.1901; Found 407.1912.

Methyl 2-azido-2-(bis(*tert*-butoxycarbonyl)amino)-3-cyclobutylpropanoate 3y

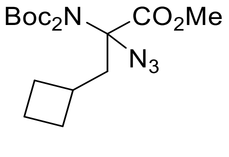

Synthesized following precedure F, starting from *N*Boc$_2$-Dha-*O*Me **1a** (75.3 mg, 0.250 mmol) and di(cyclobutanecarboxyl) peroxide **2l** (99.1 mg, 0.500 mmol, 2.0 equiv.). Purification by column chromatography (SiO_2, pentane:EtOAc = 100:0 to 96:4) afforded methyl 2-azido-2-(bis(*tert*-butoxycarbonyl)amino)-3-cyclobutylpropanoate **3y** (89.6 mg, 0.225 mmol, 90%) as a colorless oil. **R_f** (pentane: EtOAc = 96:4) = 0.30. **^{1}H NMR** (400 MHz, $CDCl_3$) δ 3.78 (s, 3H, OCH_3), 2.48 – 2.32 (m, 1H, CH), 2.16 – 1.89 (m, 4H, 4 x CH_{Cy}), 1.87 – 1.78 (m, 1H, CH_{Cy}), 1.74 – 1.64 (m, 2H, 2 CH_{Cy}), 1.63 – 1.53 (m, 1H, CH_{Cy}), 1.50 (s, 18H, 2 x $C(CH_3)_3$). **^{13}C NMR** (101 MHz, $CDCl_3$) δ 167.7, 151.7, 84.3, 79.2, 53.2, 42.8, 31.0, 29.1, 29.0, 27.9, 18.9. **IR** (vmax, cm^{-1}) 2982 (m), 2950 (w), 2122 (s), 1757 (s), 1717 (s), 1340 (s), 1249 (s), 1161 (s), 1119 (s). **HRMS** (ESI/QTOF) m/z: $[M+Na]^+$ Calcd for C18H30N4NaO6$^+$ 421.2058; Found 421.2061.

Methyl 2-azido-2-(bis(*tert*-butoxycarbonyl)amino)-3-cyclopentylpropanoate 3z

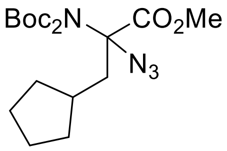

Synthesized following precedure F, starting from *N*Boc$_2$-Dha-*O*Me **1a** (75.3 mg, 0.250 mmol) and di(cyclopentanecarboxyl) peroxide **2m** (113 mg, 0.500 mmol, 2.0 equiv.). Purification by column chromatography (SiO_2, pentane:EtOAc = 100:0 to 96:4) afforded methyl 2-azido-2-(bis(*tert*-butoxycarbonyl)amino)-3-cyclopropylpropanoate **3z** (88.8 mg, 0.215 mmol, 86%) as a colorless oil. **R$_f$** (pentane: EtOAc = 96:4) = 0.43. **^{1}H NMR** (400 MHz, $CDCl_3$) δ 3.81 (s, 3H, O*CH$_3$*), 2.13 – 1.97 (m, 2H, *CH$_2$*), 1.96 – 1.85 (m, 1H, *CH$_{Cy}$*), 1.84 – 1.68 (m, 1H, *CH$_{Cy}$*), 1.57 (ddq, *J* = 10.7, 4.6, 2.2 Hz, 2H, 2 x *CH$_{Cy}$*), 1.51 (s, 21H, 2 x C(*CH$_3$*)$_3$ and 3 x *CH$_{Cy}$*), 1.19 – 1.07 (m, 1H, *CH$_{Cy}$*), 1.07 – 0.94 (m, 1H, *CH$_{Cy}$*). **^{13}C NMR** (101 MHz, $CDCl_3$) δ 168.0, 151.6, 84.1, 79.9, 53.1, 41.7, 35.5, 33.7, 33.1, 27.7, 24.83, 24.75. **IR** (vmax, cm^{-1}) 2982 (m), 2955 (m), 2871 (w), 2121 (s), 1761 (s), 1717 (s), 1339 (m), 1242 (s), 1161 (s), 1124 (s). **HRMS** (ESI/QTOF) m/z: $[M+Na]^+$ Calcd for $C_{19}H_{32}N_4NaO_6^+$ 435.2214; Found 435.2218.

Methyl 2-azido-2-(bis(tert-butoxycarbonyl)amino)-3-cyclohexylpropanoate 3aa

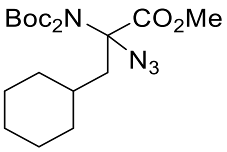

Synthesized following precedure F, starting from *N*Boc$_2$-Dha-*O*Me **1a** (75.3 mg, 0.250 mmol) and di(cyclohexanecarboxyl) peroxide **2n** (127 mg, 0.500 mmol, 2.0 equiv.). Purification by column chromatography (SiO_2, pentane:EtOAc = 100:0 to 96:4) afforded methyl 2-azido-2-(bis(*tert*-butoxycarbonyl)amino)-3-cyclohexylpropanoate **3aa** (94.8 mg, 0.222 mmol, 89%) as a white solid. **R$_f$** (pentane: EtOAc = 96:4) = 0.39. ^{1}H NMR (400 MHz, $CDCl_3$) δ 3.80 (s, 3H, O*CH$_3$*), 1.96 – 1.76 (m, 3H, C*H$_2$* and C*H$_{Cy}$*), 1.69 – 1.54 (m, 2H, 2 x C*H$_{Cy}$*), 1.50 (s, 19H, 2 x C(C*H$_3$*)$_3$ and *CH$_{Cy}$*), 1.44 – 1.29 (m, 1H, C*H$_{Cy}$*), 1.29 – 1.02 (m, 4H, 4 x C*H$_{Cy}$*), 1.02 – 0.82 (m, 2H, 2 x C*H$_{Cy}$*). **^{13}C NMR** (101 MHz, $CDCl_3$) δ 168.0, 151.7, 84.2, 79.9, 53.1, 42.8, 34.2, 34.1, 33.3, 27.8, 26.3, 26.19, 26.16. **IR** (vmax, cm^{-1}) 2979 (w), 2932 (m), 2856 (w), 2118 (m), 1757 (s), 1717 (s), 1339 (m), 1245 (s), 1122 (s). **m.p.** 66-68 °C. **HRMS** (ESI/QTOF) m/z: $[M+Na]^+$ Calcd for $C_{20}H_{34}N_4NaO_6^+$ 449.2371; Found 449.2373.

Methyl 2-azido-2-(bis(*tert*-butoxycarbonyl)amino)-3-(tetrahydro-2H-pyran-4-yl)propanoate 3ab

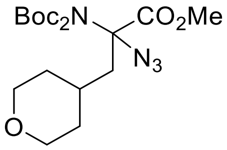

Synthesized following precedure H, starting from *N*Boc$_2$-Dha-*O*Me **1a** (75.3 mg, 0.250 mmol) and *tert*-butyl tetrahydropyrane-4-carboperoxoate **2q** (101 mg, 0.500 mmol, 2.0 equiv.) for 4 h. Purification by column chromatography (SiO_2, pentane:EtOAc = 95:5 to 7:3) afforded methyl 2-azido-2-(bis(tert-butoxycarbonyl)amino)-3-(tetrahydro-2H-pyran-4-yl)propanoate

3ab (56.5 mg, 0.132 mmol, 53%) as a colorless oil. **R$_f$** (pentane: EtOAc = 8:2) = 0.48. **^{1}H NMR** (400 MHz, $CDCl_3$) δ 3.93 – 3.85 (m, 2H, 2 x OC*H$_a$*), 3.82 (s, 3H, OC*H$_3$*), 3.39 – 3.29 (m, 2H, 2 x OC*H$_a$*), 1.98 (dd, *J* = 13.8, 6.7 Hz, 1H, C*H$_{Cy}$*), 1.91 – 1.78 (m, 2H, 2 x C*H$_{Cy}$*), 1.68 – 1.54 (m, 1H, C*H$_{Cy}$*), 1.50 (d, *J* = 2.9 Hz, 19H, 2 x C(C*H$_3$*)$_3$ and C*H$_{Cy}$*), 1.39 – 1.16 (m, 2H, 2 x C*H$_{Cy}$*). **^{13}C NMR** (101 MHz, $CDCl_3$) δ 167.9, 151.7, 84.4, 79.7, 67.94, 67.86, 53.3, 42.4, 33.8, 31.0, 27.9. **IR** (vmax, cm^{-1}) 2986 (m), 2931 (w), 2852 (w), 2121 (s), 1757 (s), 1717 (s), 1339 (s), 1243 (s), 1125 (s). **HRMS** (ESI/QTOF) m/z: [M+Na]$^+$ Calcd for $C_{19}H_{32}N_4NaO_7^+$ 451.2163; Found 451.2166.

(9H-fluoren-9-yl)methyl 4-(2-azido-2-(bis(*tert*-butoxycarbonyl)amino)-3-methoxy-3-oxopropyl)piperidine-1-carboxylate 3ac

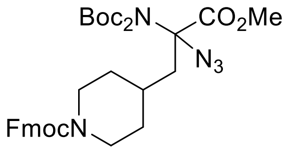

Synthesized following precedure F, starting from *N*Boc$_2$-Dha-*O*Me **1a** (75.3 mg, 0.250 mmol) and 1-(((9H-fluoren-9-yl)methoxy)carbonyl)piperidine-4-*tert*-butyl peroxide **2r** (212 mg, 0.500 mmol, 2.0 equiv.). Purification by column chromatography (SiO_2, pentane:EtOAc = 95:5 to 7:3) afforded (9H-fluoren-9-yl)methyl 4-(2-azido-2-(bis(*tert*-butoxycarbonyl)amino)-3-methoxy-3-oxopropyl)piperidine-1-carboxylate **3ac** (92.9 mg, 0.143 mmol, 57%) as a colorless oil. **R$_f$** (pentane: EtOAc = 75:25) = 0.29. ^{1}H NMR (400 MHz, $CDCl_3$) δ 7.76 (d, *J* = 7.5 Hz, 2H, 2 x Ar*H*), 7.57 (d, *J* = 7.4 Hz, 2H, 2 x Ar*H*), 7.40 (t, *J* = 7.4 Hz, 2H, 2 x Ar*H*), 7.31 (t, *J* = 7.5 Hz, 2H, 2 x Ar*H*), 4.41 (s, 2H, C*H$_{2Fmoc}$*), 4.23 (t, *J* = 6.8 Hz, 1H, C*H$_{Fmoc}$*), 4.16 – 3.93 (m, 2H, 2 x NC*H*), 3.84 (s, 3H, OC*H$_3$),* 2.75 (s, 2H, 2 x NC*H*), 2.02 – 1.81 (m, 3H, C*H$_2$* and C*H*), 1.52 (s, 20H, 2 x C(C*H$_3$*)$_3$ and C*H$_2$*), 1.10 (s, 2H, C*H$_2$*). **^{13}C NMR** (101 MHz, $CDCl_3$) δ 167.9, 155.3, 151.7, 144.2, 141.5, 127.8, 127.2, 125.1, 120.1, 84.5, 79.7, 67.2, 53.4, 47.6, 44.1, 42.0, 32.82, 32.75, 27.9. **IR** (vmax, cm^{-1}) 2982 (w), 2935 (w), 2124 (s), 1757 (s), 1715 (s), 1703 (s), 1452 (m), 1343 (m), 1260 (s), 1130 (s). **HRMS** (ESI/QTOF) m/z: [M+Na]$^+$ Calcd for $C_{34}H_{43}N_5NaO_8^+$ 672.3004; Found 672.3012.

Methyl 3-((3r,5r,7r)-adamantan-1-yl)-2-azido-2-(bis(*tert*-butoxycarbonyl)amino) propanoate 3ad

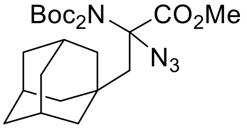

Synthesized following precedure F, starting from *N*Boc$_2$-Dha-*O*Me **1a** (75.3 mg, 0.250 mmol) and *tert*-butyl adamantyl peroxide **2s** (126 mg, 0.500 mmol, 2.0 equiv.) for 4 h. Purification by column chromatography (SiO_2, pentane:EtOAc = 100:0 to 96:4) afforded methyl 3-((3r,5r,7r)-adamantan-1-yl)-2-azido-2-(bis(*tert*-butoxycarbonyl)amino) propanoate **3ad** (80.1 mg, 0.167 mmol, 67%) as a white solid. **^{1}H NMR** (400 MHz, $CDCl_3$) δ 3.80 (s, 3H, OC*H$_3$*), 1.94 – 1.88 (m, 3H, 3 x C*H$_{Ad}$*), 1.87 – 1.76 (m, 2H, C*H$_2$*), 1.69 – 1.58 (m, 10H, 5 x C*H$_{2Ad}$*), 1.57 – 1.54 (m, 2H, C*H$_{2Ad}$*), 1.50 (s, 18H, 2 x C(C*H$_3$*)$_3$). **^{13}C NMR** (101 MHz, $CDCl_3$) δ 168.2, 151.5, 84.2, 78.3, 53.1, 48.6, 42.2, 36.8, 33.1, 28.7, 27.8. **IR** (vmax, cm^{-1}) 2979 (m), 2904 (m), 2849 (w), 2121 (s), 1757 (s), 1718 (s), 1339 (s), 1249 (s), 1164 (s), 1123 (s). **m. p.** 78-80. °C. **HRMS** (ESI/QTOF) m/z: [M+Na]$^+$ Calcd for $C_{24}H_{38}N_4NaO_6^+$ 501.2684; Found 501.2685.

(9H-fluoren-9-yl)methyl (2-azido-1-((2-benzoyl-4-chlorophenyl)amino)-1-oxobutan-2-yl) carbamate 3ae

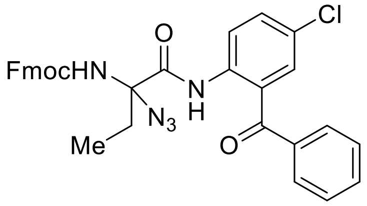

Synthesized following precedure G, starting from (9H-Fluoren-9-yl)methyl-(3-((2-benzoyl-4-chlorophenyl)amino)-3-oxoprop-1-en-2-yl)carbamate **1i** (131 mg, 0.250 mmol) and TBPB (**2b**) (95.1 µL, 0.500 mmol, 2.0 equiv.) at 0 °C. Purification by column chromatography (SiO_2, pentane:EtOAc = 95:5 to 8:2) afforded (9H-fluoren-9-yl)methyl (2-azido-1-((2-benzoyl-4-chlorophenyl)amino)-1-oxobutan-2-yl) carbamate **3ae** (143 mg, 0.247 mmol, 99%) as a colorless sticky oil. **R_f** (pentane: EtOAc = 8:2) = 0.34. **^{1}H NMR** (400 MHz, $CDCl_3$) δ 11.53 (s, 1H, N*H*), 8.67 – 8.58 (m, 1H, Ar*H*), 7.85 – 7.29 (m, 15H, 15 x Ar*H*), 6.05 (s, 1H, N*H*), 4.44 (qd, J = 10.7, 6.9 Hz, 2H, CH_{2Fmoc}), 4.20 (s, 1H, CH_{Fmoc}), 2.33 (s, 1H, CH_a), 2.12 – 1.95 (m, 1H, CH_b), 1.02 (t, J = 7.5 Hz, 3H, CH_3). **^{13}C NMR** (101 MHz, $CDCl_3$) δ 198.0, 167.3, 154.7, 143.6, 141.4, 137.8, 137.7, 134.0, 133.1, 132.9, 130.1, 128.7, 128.4, 127.9, 127.24, 127.23, 125.7, 125.1, 123.2, 120.1, 78.4, 67.7, 47.1, 8.1. (1C not resolved). **IR** (vmax, cm^{-1}) 3317 (w), 3058 (w), 2985 (w), 2121 (s), 1727 (m), 1706 (s), 1505 (s), 1245 (s), 741 (s). **HRMS** (ESI/QTOF) m/z: $[M+Na]^+$ Calcd for $C_{32}H_{26}ClN_5NaO_4^+$ 602.1566; Found 602.1568.

Benzyl (2S)-4-azido-2-(*tert*-butyl)-4-ethyl-5-oxooxazolidine-3-carboxylate 3af

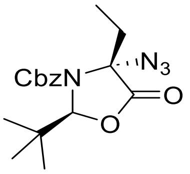

Synthesized following precedure F, starting from benzyl (S)-2-(*tert*-butyl)-4-methylene-5-oxooxazolidine-3-carboxylate **1j** (2.03, 7.00 mmol) and TBPB **2b** (2.66 mL, 14.0 mmol, 2.00 equiv.). Purification by column chromatography (SiO_2, pentane:EtOAc = 100:0 to 97:3) afforded Benzyl (2S)-4-azido-2-(*tert*-butyl)-4-ethyl-5-oxooxazolidine-3-carboxylate **3af** (2.2 g, 6.3 mmol, 90%) as a colorless oil and a single diastereoisomer. **R_f** (pentane: EtOAc = 97.5:2.5) = 0.23. **^{1}H NMR** (400 MHz, $CDCl_3$) δ 7.45 – 7.33 (m, 5H, 5 x Ar*H*), 5.56 (s, 1H, C*H*), 5.28 – 5.16 (m, 2H, CH_{2Cbz}), 2.42 (q, *J* = 7.5 Hz, 2H, CH_2), 1.09 (t, *J* = 7.5 Hz, 3H, CH_3), 0.96 (s, 9H, C(CH_3)$_3$). **^{13}C NMR** (101 MHz, $CDCl_3$) δ 168.0, 155.2, 134.9, 128.9, 128.9, 128.7, 95.0, 79.1, 68.8, 38.1, 27.8, 25.5, 9.3. **IR** (vmax, cm^{-1}) 2971 (m), 2909 (w), 2107 (s), 1800 (s), 1736 (s), 1389 (m), 1328 (m), 1287 (s), 1224 (s), 1202 (s), 1044 (s). **HRMS** (ESI/QTOF) m/z: $[M+Na]^+$ Calcd for $C_{17}H_{22}N_4NaO_4^+$ 369.1533; Found 369.1537. $[\alpha]_D^{25}$ = -18.8 (c = 0.44, $CHCl_3$)

Benzyl (2S)-4-azido-2-(*tert*-butyl)-4-(3,3-dimethylbutyl)-5-oxooxazolidine-3-carboxylate 3ag

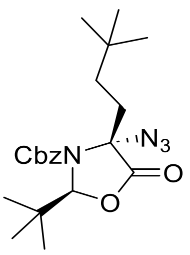

Synthesized following precedure F, starting from benzyl (S)-2-(*tert*-butyl)-4-methylene-5-oxooxazolidine-3-carboxylate **1j** (79 mg, 0.27 mmol) and dineopentyl peroxyde **2f** (115 mg, 0.540 mmol, 2.0 equiv.). Purification by column chromatography (SiO_2, pentane:EtOAc = 100:0 to 98:2) afforded benzyl (2S)-4-azido-2-(*tert*-butyl)-4-(3,3-dimethylbutyl)-5-oxooxazolidine-3-carboxylate **3ag** (87.5 mg, 0.217 mmol, 80%) as a colorless oil and a single diastereoisomer. $\mathbf{R_f}$ (pentane: EtOAc = 97.5:2.5) = 0.57. **^{1}H NMR** (400 MHz, $CDCl_3$) δ 7.43 – 7.31 (m, 5H, 5 x Ar*H*), 5.55 (s, 1H, C*Ht*Bu), 5.27 (d, *J* = 11.9 Hz, 1H, CH_{aBn}), 5.17 (d, *J* = 11.8 Hz, 1H, CH_{bBn}), 2.38 – 2.22 (m, 2H, CH_2), 1.53 – 1.44 (m, 1H, CH_a), 1.21 – 1.05 (m, 1H, CH_b), 0.96 (s, 9H, $C(CH_3)_3$), 0.84 (s, 9H, $C(CH_3)_3$). **^{13}C NMR** (101 MHz, $CDCl_3$) δ 168.1, 155.2, 134.8, 129.0, 128.88, 128.85, 95.0, 78.9, 68.9, 38.14, 38.08, 30.3, 29.8, 29.1, 25.6. **IR** (vmax, cm^{-1}) 2959 (s), 2867 (m), 2110 (s), 1802 (s), 1732 (s), 1231 (s), 1181 (s), 1040 (s). **HRMS** (ESI/QTOF) m/z: $[M+Na]^+$ Calcd for $C_{21}H_{30}N_4NaO_4^+$ 425.2159; Found 425.2152. $[\alpha]_D^{25}$ = -2.4 (c = 0.39, $CHCl_3$)

Methyl 2-azido-2-(bis(*tert*-butoxycarbonyl)amino)-3-methylbutanoate 3ah

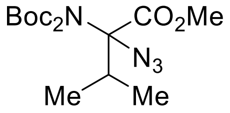

Synthesized following precedure G, starting from *N*Boc_2-Dhb-*O*Me **1h** (78.8 mg, 0.250 mmol) and diacetyl peroxide **2d** (59 mg, 0.50 mmol, 2.0 equiv.). Purification by column chromatography (SiO_2, pentane:EtOAc = 100:0 to 95:5) afforded methyl 2-azido-2-(bis(*tert*-butoxycarbonyl)amino)-3-methylbutanoate **3ah** (16.6 mg, 0.0445 mmol, 18%) as a colorless oil. $\mathbf{R_f}$ (pentane: EtOAc = 95:5) = 0.41. **^{1}H NMR** (400 MHz, $CDCl_3$) δ 3.81 (s, 3H, OCH_3), 2.62 (p, *J* = 6.8 Hz, 1H, C*H*), 1.51 (s, 18H, 2 x $C(CH_3)_3$), 1.03 (d, *J* = 6.8 Hz, 3H, CH_3), 0.96 (d, *J* = 6.9 Hz, 3H, CH_3). **^{13}C NMR** (101 MHz, $CDCl_3$) δ 166.6, 152.5, 84.6, 84.2, 52.8, 34.2, 27.9, 18.2, 17.4. **IR** (vmax, cm^{-1}) 2982 (m), 2938 (w), 2117 (s), 1750 (s), 1721 (s), 1339 (m), 1232 (s), 1166 (s), 1130 (s). **m.p.** 73-75 °C. **HRMS** (ESI/QTOF) m/z: $[M+Na]^+$ Calcd for $C_{16}H_{28}N_4NaO_6^+$ 395.1901; Found 395.1904.

5. Scale-up

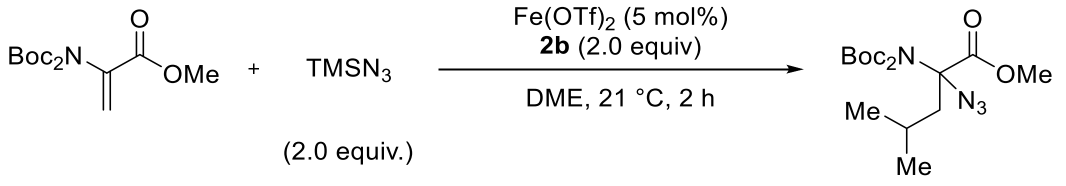

1a **3c**

To an oven-dried vial, $Fe(OTf)_2$ (93.2 mg, 0.250 mmol, 0.05 equiv.) was added in a glovebox. The vial was removed from the glovebox and placed under nitrogen. To two different oven-dried vial, *N*Boc$_2$-Dha-*O*Me **1a** (1.51 g, 5.00 mmol, 1.00 equiv.) and diisobutyryl peroxide **2c** (1.74 g, 10.0 mmol, 2.00 equiv.) were added under nitrogen atmosphere. DME (20.0 mL) was added to the peroxide vial and it was transferred to the dehydroamino acid vial via syringe. $TMSN_3$ (1.41 mL, 10.0 mmol, 2.00 equiv.) was then added. The solution was added to the catalyst vial via syringe and the deep purple mixture was stirred vigorously for 2 hours at 21 °C. The reaction mixture was diluted with EtOAc and filtered through celite/silica (1:1). Purification by column chromatography (SiO_2, pentane:EtOAc = 100:0 to 96:4) afforded methyl 2-azido-2-(bis(*tert*-butoxycarbonyl)amino)-4-methylpentanoate **3c** (1.57 g, 4.07 mmol, 81%) as a colorless oil. $\mathbf{R_f}$ (pentane: EtOAc = 95:5) = 0.28. ^{1}**H NMR** (400 MHz, $CDCl_3$) δ 3.81 (s, 3H, OC*H$_3$*), 1.97 – 1.81 (m, 2H, C*H$_2$*), 1.72 – 1.62 (m, 1H, C*H*), 1.51 (s, 18H, 2 x C(C*H$_3$*)$_3$), 0.99 (d, *J* = 6.7 Hz, 3H, C*H$_3$*), 0.87 (d, *J* = 6.7 Hz, 3H, C*H$_3$*). ^{13}**C NMR** (101 MHz, $CDCl_3$) δ 167.9, 151.7, 84.2, 80.0, 53.2, 44.0, 27.9, 24.4, 24.0, 23.5. **IR** (vmax, cm^{-1}) 2980 (m), 2939 (w), 2878 (w), 2125 (s), 1753 (s), 1717 (s), 1370 (m), 1339 (m), 1249 (s), 1161 (s), 1128 (s). **HRMS** (ESI/QTOF) m/z: $[M+Na]^+$ Calcd for $C_{17}H_{30}N_4NaO_6^+$ 409.2058; Found 409.2051.

6. Speculative Mechanism[20]

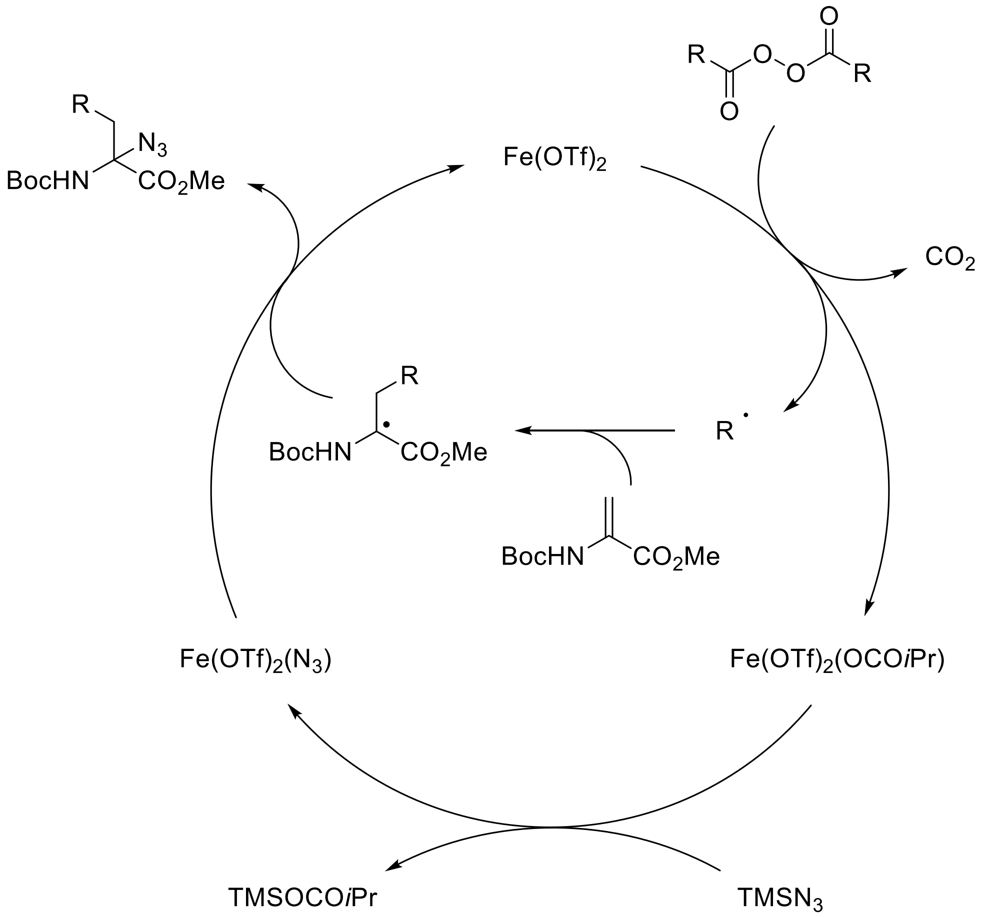

7. Product modification

5.1 Intermolecular Huisgen [3+2]-cycloadditions

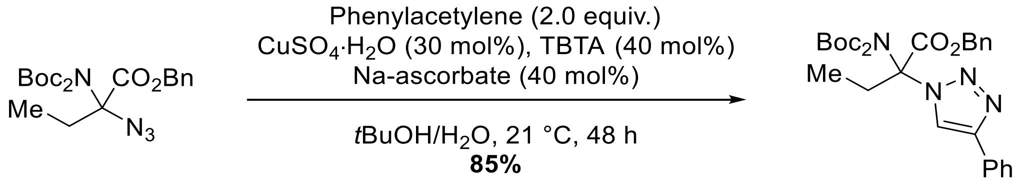

3m **5a**

Phenylacetylene (44 µL, 0.40 mmol, 2.0 equiv.) was added to a solution of **3m** (87 mg, 0.20 mmol), $CuSO_4.H_2O$ (15 mg, 0.060 mmol, 0.30 equiv.), sodium L-ascorbate (12 mg, 0.060 mmol, 0.30 equiv.) and TBTA (32 mg, 0.060 mmol, 0.30 equiv.) in *t*BuOH (2.0 mL) and H_2O (0.60 mL). The mixture was stirred at 21 °C for 48 h. The reaction was diluted with water and extracted 3 times with EtOAc. The organic layers were gathered, washed with brine, dried over $MgSO_4$, filtered off and solvents were removed under reduced pressure. Purification by column

[20] R. Wei, H. Xiong, C. Ye, Y. Li, H. Bao, *Org. Lett.* **2020**, *22*, 3195–3199.

chromatography (SiO_2, pentane:EtOAc = 98:2 to 9:1) afforded benzyl 2-(bis(*tert*-butoxycarbonyl)amino)-2-(4-phenyl-1H-1,2,3-triazol-1-yl)butanoate **5a** (90.8 mg, 0.169 mmol, 85%) as an amorphous colorless solid. **R_f** (pentane: EtOAc = 96:4) = 0.42. **^{1}H NMR** (400 MHz, $CDCl_3$) δ 8.38 (s, 1H, Ar$H_{triazole}$), 7.88 – 7.84 (m, 2H, 2 x Ar*H*), 7.46 – 7.27 (m, 8H, 8 x Ar*H*), 5.36 – 5.24 (m, 2H, CH_{2Bn}), 3.31 (dq, *J* = 14.6, 7.3 Hz, 1H, CH_a), 2.47 (dq, *J* = 13.7, 7.3 Hz, 1H, CH_b), 1.41 (s, 18H, 2 x C(CH_3)$_3$), 0.94 (t, *J* = 7.3 Hz, 3H, CH_3). **^{13}C NMR** (101 MHz, $CDCl_3$) δ 166.3, 151.5, 146.9, 134.9, 131.1, 128.9, 128.72, 128.70, 128.6, 128.0, 125.9, 122.1, 84.5, 79.6, 68.6, 28.9, 27.8, 8.8. **IR** (vmax, cm^{-1}) 3178 (w), 2979 (m), 1757 (s), 1714 (s), 1335 (m), 1249 (m), 1224 (m), 1158 (s), 1126 (s). **HRMS** (ESI/QTOF) m/z: [M+H]$^+$ Calcd for $C_{29}H_{37}N_4O_6^+$ 537.2708; Found 537.2717.

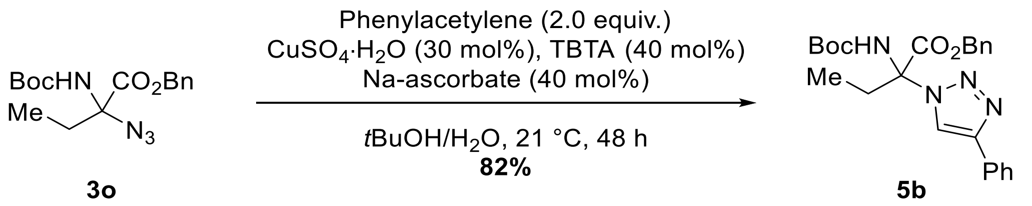

Phenylacetylene (44 µL, 0.40 mmol, 2.0 equiv.) was added to a solution of **3o** (67 mg, 0.20 mmol), $CuSO_4.H_2O$ (15 mg, 0.060 mmol, 0.30 equiv.), sodium L-ascorbate (12 mg, 0.060 mmol, 0.30 equiv.) and TBTA (32 mg, 0.060 mmol, 0.30 equiv.) in *t*BuOH (2.0 mL) and H_2O (0.60 mL). The mixture was stirred at 21 °C for 48 h. The reaction was diluted with water and extracted 3 times with EtOAc. The organic layers were gathered, washed with brine, dried over $MgSO_4$, filtered off and solvents were removed under reduced pressure. Purification by column chromatography (SiO_2, pentane:EtOAc = 95:5 to 8:2) afforded benzyl 2-((*tert*-butoxycarbonyl)amino)-2-(4-phenyl-1H-1,2,3-triazol-1-yl)butanoate **5b** (71.6 mg, 0.164 mmol, 82%) as a amorphous yellowish solid. **R_f** (pentane: EtOAc = 8:2) = 0.38. **^{1}H NMR** (400 MHz, $CDCl_3$) δ 8.25 (s, 1H, Ar$H_{triazole}$), 7.90 – 7.79 (m, 2H, 2 x Ar*H*), 7.42 (t, *J* = 7.5 Hz, 2H, 2 x Ar*H*), 7.38 – 7.28 (m, 6H, 6 x Ar*H*), 6.17 (s, 1H, N*H*), 5.33 (d, *J* = 12.0 Hz, 1H, CH_{aBn}), 5.21 (d, *J* = 12.0 Hz, 1H, CH_{bBn}), 3.04 – 2.85 (m, 1H, CH_a), 2.79 – 2.63 (m, 1H, CH_a), 1.36 (s, 9H, C(CH_3)$_3$), 0.90 (t, *J* = 7.4 Hz, 3H, CH_3). **^{13}C NMR** (101 MHz, $CDCl_3$) δ 167.3, 153.5, 146.9, 134.5, 130.8, 128.9, 128.8, 128.7, 128.2, 125.9, 120.5, 81.5, 77.2, 69.0, 28.8, 28.2, 8.0. (1C not resolved). **IR** (vmax, cm^{-1}) 3383 (w), 2982 (m), 2123 (w), 1753 (s), 1721 (s), 1509 (m), 1458 (m), 1256 (m), 1155 (s). **HRMS** (ESI/QTOF) m/z: [M+Na]$^+$ Calcd for $C_{24}H_{28}N_4NaO_4^+$ 459.2003; Found 459.2005.

5.2 Staudinger – Amide coupling

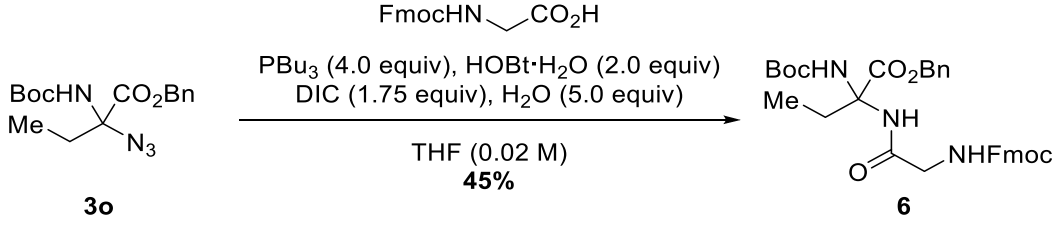

Following the reported procedure,[21] Fmoc-Gly-OH (1.2 g, 4.0 mmol, 2.00 equiv) and HOBt.H_2O (613 mg, 4.00 mmol, 2.00 equiv) were dissolved in THF (60 mL). The mixture was cooled down

[21] A. Charafeddine, H. Chapuis, P. Strazewski, *Org. Lett.* **2007**, *9*, 2787–2790.

to 0 °C and stirred for 10 minutes. DIC (626 µL, 4.00 mmol, 2.00 equiv) was added and the mixture was stirred another 10 minutes. A solution of benzyl 2-azido-2-((*tert*-butoxycarbonyl)amino)butanoate **3o** (669 mg, 2.00 mmol, 1.00 equiv) and P(*nBu*)$_3$ (1.97 mL, 8.00 mmol, 4.00 equiv) in THF (20 mL) was added followed by water (180 µL, 10.0 mL, 5.00 equiv.) and the mixture was stirred for 14 h at room temperature. Water and MeOH were added and the solvents were removed under reduced pressure. Purification by column chromatography (Al_2O_3, pentane:EtOAc = 9:1 to 0:100) afforded benzyl 2-(2-((((9H-fluoren-9-yl)methoxy)carbonyl)amino)acetamido)-2-((*tert*-butoxy carbonyl)amino)butanoate **6** (526 mg, 0.896 mmol, 45%) as a white solid. **R$_f$** (SiO_2, pentane: EtOAc = 6:4) = 0.35. **R$_f$** (Al_2O_3, pentane: EtOAc = 6:4) = 0.26. **^{1}H NMR** (400 MHz, $CDCl_3$) δ 7.77 (d, *J* = 7.5 Hz, 2H, 2 x Ar*H*), 7.60 (d, *J* = 7.5 Hz, 2H, 2 x Ar*H*), 7.40 (t, 2H, 2 x Ar*H*), 7.36 – 7.28 (m, 7H, 7 x Ar*H*), 5.97 (s, 1H, N*H*), 5.41 – 5.32 (m, 1H, N*H*), 5.30 – 5.15 (m, 2H, CH_{2Bn}), 4.45 – 4.35 (m, 2H, CH_{2Fmoc}), 4.23 (t, *J* = 7.2 Hz, 1H, CH_{Fmoc}), 3.89 (d, *J* = 5.6 Hz, 2H, CH_{2Gly}), 2.47 – 2.32 (m, 1H, CH_a), 2.19 – 2.07 (m, 1H, CH_a), 1.38 (s, 9H, C(CH_3)$_3$), 0.72 (t, *J* = 7.5 Hz, 3H, CH_3). (1 NH not resolved). **^{13}C NMR** (101 MHz, $CDCl_3$) δ 169.8, 168.3, 156.5, 154.1, 143.9, 141.4, 135.0, 128.74, 128.71, 127.9, 127.2, 125.3, 120.1, 80.7, 71.0, 68.6, 67.5, 47.2, 44.5, 29.9, 28.3, 7.8. (1C not resolved). **IR** (vmax, cm^{-1}) 3339 (w), 2968 (m), 2932 (m), 2120 (w), 1717 (s), 1684 (s), 1509 (s), 1247 (s), 1170 (m), 1145 (s). **m.p.** 84-86 °C. **HRMS** (ESI/QTOF) m/z: [M+Na]$^+$ Calcd for $C_{33}H_{37}N_3NaO_7^+$ 610.2524; Found 610.2531.

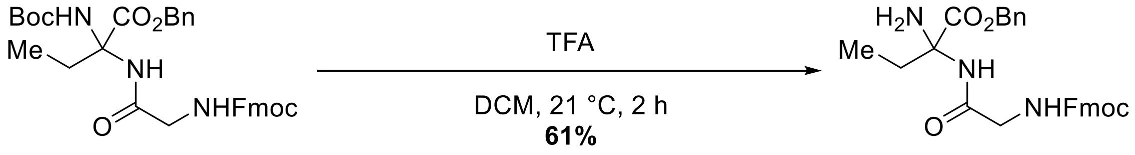

6 **7**

TFA (2.3 mL, 30 mmol, 50.0 equiv.) was added to a solution of benzyl 2-(2-((((9H-fluoren-9-yl)methoxy)carbonyl)amino)acetamido)-2-aminobutanoate **6** (353 mg, 0.600 mmol, 1.00 equiv.) in 6 mL of DCM at 0 °C. The mixture was stirred 2 h. The mixture was then basified with $NaHCO_3$ and extracted with DCM (3 x 20 mL). The organic layers were gathered, washed with brine, dried over $MgSO_4$, filtered off and the solvents were removed under reduced pressure. Purification by column chromatography (SiO_2, DCM:MeOH = 100:0 to 95:5) afforded benzyl 2-(2-((((9H-fluoren-9-yl)methoxy)carbonyl)amino)acetamido)-2-aminobutanoate **7** (179 mg, 0.368 mmol, 61%) as a white solid. **R$_f$** (DCM:MeOH = 97:3) = 0.29. **^{1}H NMR** (400 MHz, $CDCl_3$) δ 7.77 (d, *J* = 7.5 Hz, 2H, 2 x Ar*H*), 7.58 (d, *J* = 7.4 Hz, 2H, 2 x Ar*H*), 7.40 (t, *J* = 7.2 Hz, 2H, 2 x Ar*H*), 7.36 – 7.28 (m, 7H, 7 x Ar*H*), 6.82 (s, 1H, N*H*), 5.37 (s, 1H, N*H*), 5.19 (d, *J* = 2.6 Hz, 2H, CH_{2Bn}), 4.42 (d, *J* = 7.0 Hz, 2H, CH_{2Fmoc}), 4.22 (t, *J* = 7.0 Hz, 1H, CH_{Fmoc}), 3.84 (d, *J* = 6.1 Hz, 2H, CH_{2Gly}), 2.42 (s, 2H, NH_2), 2.07 – 1.94 (m, 1H, CH_a), 1.91 – 1.78 (m, 1H, CH_a), 0.83 (t, *J* = 7.5 Hz, 3H, CH_3). **^{13}C NMR** (101 MHz, $CDCl_3$) δ 172.4, 169.2, 156.7, 143.8, 141.5, 135.4, 128.7, 128.7, 128.6, 127.9, 127.3, 125.2, 120.2, 71.9, 68.0, 67.5, 47.2, 44.7, 31.8, 7.9. **IR** (vmax, cm^{-1}) 3307 (w), 3036 (w), 2977 (w), 2123 (w), 1721 (s), 1683 (m), 1533 (m), 1450 (m), 1246 (m). **m.p.** 57-59 °C. **HRMS** (ESI/QTOF) m/z: [M+Na]$^+$ Calcd for $C_{28}H_{29}N_3NaO_5^+$ 510.1999; Found 510.1999.

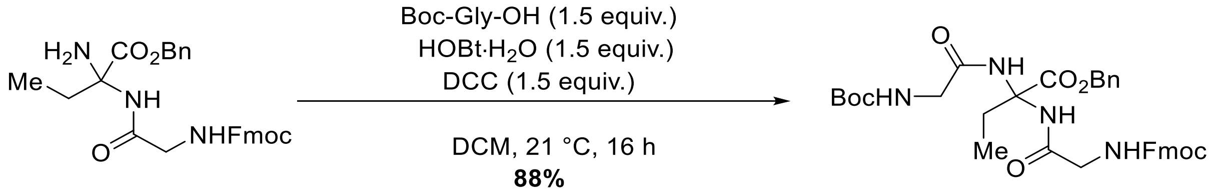

Benzyl 2-(2-((((9H-fluoren-9-yl)methoxy)carbonyl)amino)acetamido)-2-aminobutanoate **7** (73 mg, 0.15 mmol, 1 equiv.) and DCC (46 mg, 0.23 mmol, 1.5 equiv.) were added to a strongly stirred solution of Boc-Gly-OH (39 mg, 0.23 mmol, 1.5 equiv.) and HOBt.H_2O (35 mg, 0.23 mmol, 1.5 equiv.) in DCM (3 mL). The mixture was stirred for 16 h. Sat. $NaHCO_3$ was added and the mixture was extracted with DCM (3 x 20 mL). The organic layers were gathered, washed with brine, dried over $MgSO_4$, filtered off and the solvents were removed under reduced pressure. Purification by column chromatography (SiO_2, DCM:MeOH = 100:0 to 98:2) afforded benzyl 2-(2-((((9H-fluoren-9-yl)methoxy)carbonyl)amino)acetamido)-2-(2-((*tert*-butoxycarbonyl)amino)acetamido)butanoate **8** (85 mg, 0.13 mmol, 88%) as a white solid. **R_f** (DCM:MeOH = 96:4) = 0.30. **^{1}H NMR** (400 MHz, $CDCl_3$) δ 7.76 (d, *J* = 7.5 Hz, 2H, 2 x Ar*H*), 7.60 (d, *J* = 6.9 Hz, 2H, 2 x Ar*H*), 7.40 (t, *J* = 7.5 Hz, 2H, 2 x Ar*H*), 7.35 – 7.26 (m, 7H, 7 x Ar*H*), 5.37 (s, 1H, N*H*), 5.21 (s, 2H, CH_{2Bn}), 5.04 (s, 1H, N*H*), 4.40 (d, *J* = 7.5 Hz, 2H, CH_{2Fmoc}), 4.23 (t, *J* = 7.0 Hz, 1H, CH_{Fmoc}), 3.86 (d, *J* = 5.6 Hz, 2H, CH_{2Gly}), 3.74 (s, 2H, CH_{2Gly}), 2.28 (h, *J* = 6.7 Hz, 2H, CH_2), 1.44 (s, 9H, $C(CH_3)_3$), 0.71 (t, *J* = 7.5 Hz, 3H, CH_3). (2 NH not resolved). **^{13}C NMR** (101 MHz, $CDCl_3$) δ 169.4, 169.3, 168.8, 156.6, 156.0, 144.0, 141.4, 135.0, 128.74, 128.71, 128.66, 127.9, 127.2, 125.3, 120.1, 80.4, 70.4, 68.6, 67.4, 47.2, 44.3 (2C), 28.4, 28.2, 7.6. **IR** (vmax, cm^{-1}) 3330 (m), 2978 (m), 2120 (w), 1713 (s), 1683 (s), 1671 (s), 1508 (s), 1248 (s), 1145 (s). **m.p.** 92-94 °C. **HRMS** (ESI/QTOF) m/z: $[M+Na]^+$ Calcd for $C_{35}H_{40}N_4NaO_8^+$ 667.2738; Found 667.2740.

5.3 Hydroboration- oxidation

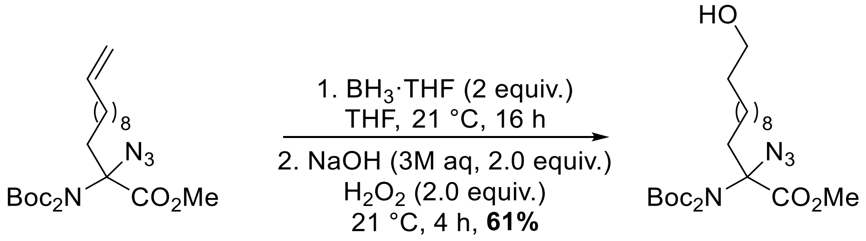

Following a reported procedure,[22] to a stirred solution of methyl 2-azido-2-(bis(*tert*-butoxycarbonyl)amino)tridec-12-enoate **3s** (48 mg, 0.10 mmol, 1.0 equiv.) in THF (0.5 mL) at 0 °C, was added dropwise BH_3.THF (1 M in THF, 0.20 mL, 0.20 mmol, 2.0 equiv.). After completion of addition, the mixture was warmed up 21 °C, and was stirred for 16 h. Then, NaOH (3 M in water, 67 µL, 0.20 mmol, 2.0 equiv.) and H_2O_2 (30% in water, 20 µL, 0.20 mmol, 2.0 equiv.) were added sequentially, and the mixture was stirred for 4 h. The mixture was extracted three times with EtOAc. The organic layers were gathered, washed with brine, dried over $MgSO_4$, filtered off and solvent were removed under reduced pressure. Purification by column chromatography (SiO_2, pentane: EtOAc = 95:5 to 7:3) afforded benzyl 2-azido-2-

[22] B. Ardiansah, H. Tanimoto, T. Tomohiro, T. Morimoto, K. Kakiuchi, *Chem. Commun.* **2021**, *57*, 8738–8741.

(bis(*tert*-butoxycarbonyl)amino)-13-hydroxytridecanoate **9** (31 mg, 0.061 mmol, 61%) as a colorless oil. **R$_f$** (pentane: EtOAc = 7:3) = 0.53. **^{1}H NMR** (400 MHz, $CDCl_3$) δ 3.82 (s, 3H, OC*H$_3$*), 3.64 (t, *J* = 6.6 Hz, 2H, C*H$_2$*OH), 2.03 – 1.87 (m, 2H, CC*H$_2$*), 1.51 (s, 22H, 2 x C(C*H$_3$*)$_3$ and C*H$_2$* and O*H* and C*H$_a$*), 1.24 (s, 14H, 7 x C*H$_2$*), 1.10 (td, *J* = 12.2, 5.6 Hz, 1H, C*H$_b$*). **^{13}C NMR** (101 MHz, $CDCl_3$) δ 168.0, 151.8, 84.3, 80.2, 63.3, 53.3, 36.1, 33.0, 29.67, 29.59, 29.54, 29.51, 29.42, 29.36, 27.9, 25.9, 23.9. **IR** (vmax, cm^{-1}) 2979 (m), 2929 (m), 2856 (m), 2125 (s), 1758 (s), 1721 (s), 1339 (s), 1253 (s), 1127 (s). **HRMS** (ESI/QTOF) m/z: [M+Na]$^+$ Calcd for $C_{24}H_{44}N_4NaO_7^+$ 523.3102; Found 523.3108.

5.4 Intramolecular Huisgen [3+2]-cycloadditions

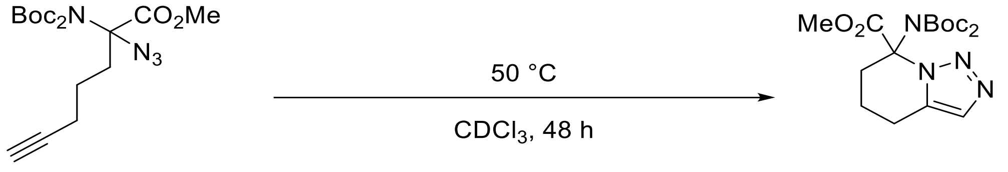

3t **10**

A solution of methyl 2-azido-2-(bis(tert-butoxycarbonyl)amino)hept-6-ynoate **3t** (79 mg, 0.20 mmol) in $CDCl_3$ (8 mL) was stirred at 50 °C for 48 h. The, solvents were removed under reduced pressure. Purification by column chromatography (SiO_2, pentane: EtOAc = 6:4 to 4:6) afforded methyl 7-(bis(*tert*-butoxycarbonyl)amino)-4,5,6,7-tetrahydro-[1,2,3]triazolo[1,5-a]pyridine-7-carboxylate **10** (75 mg, 0.19 mmol, 95%) as a white solid. **R$_f$** (pentane: EtOAc = 4:6) = 0.56. **^{1}H NMR** (400 MHz, $CDCl_3$) δ 7.44 (s, 1H, Ar*H$_{Triazole}$*), 3.81 (s, 3H, OC*H$_3$*), 2.99 – 2.89 (m, 1H, C*H*), 2.86 – 2.69 (m, 2H, 2 x C*H*), 2.62 (ddd, *J* = 13.6, 11.9, 3.3 Hz, 1H, C*H*), 2.08 (dtt, *J* = 13.1, 5.9, 3.5 Hz, 1H, C*H*), 1.95 – 1.82 (m, 1H, C*H*), 1.46 (s, 18H, 2 x C(C*H$_3$*)$_3$). **^{13}C NMR** (101 MHz, $CDCl_3$) δ 167.6, 151.7, 135.3, 130.5, 84.2, 76.6, 53.7, 31.4, 27.9, 20.0, 18.1. **IR** (vmax, cm^{-1}) 2979 (m), 1751 (s), 1728 (s), 1335 (s), 1238 (s), 1154 (s), 1120 (s). **m.p.** 102-103 °C. **HRMS** (ESI/QTOF) m/z: [M+H]$^+$ Calcd for $C_{18}H_{29}N_4O_6^+$ 397.2082; Found 397.2080.

5.5 Chiral *α*-azidated amino acids functionalization

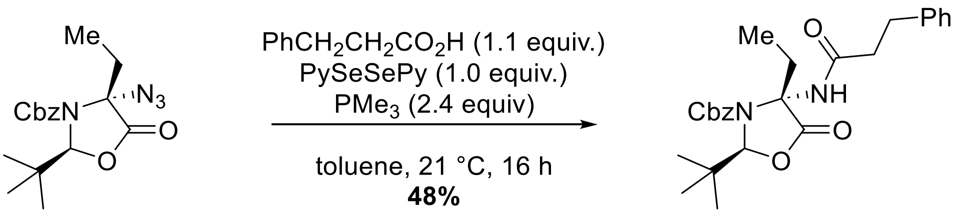

3af **11**

Following a modified reported procedure,[23] a solution of benzyl (2S)-4-azido-2-(*tert*-butyl)-4-ethyl-5-oxooxazolidine-3-carboxylate **3af** (520 mg, 1.50 mmol) and hydrocinnamic acid **(**248 mg, 1.65 mmol, 1.1 equiv.) in toluene (7.5 mL) was cooled to 0 °C. 2,2′-dipyridyl diselenide (471 mg, 1.50 mmol, 1.00 equiv.) was added in one portion. PMe_3 (1 M in THF, 3.60 mL, 2.40 equiv.) was added dropwise. After 2 h, the ice bath was removed and the solution was stirred for 16 h. Water (20 mL) was added and the mixture was stirred for 5 min. Sat. aqueous

[23] J. Burés, M. Martín, F. Urpí, J. Vilarrasa, *J. Org. Chem.* **2009**, *74*, 2203–2206.

$NaHCO_3$ (20 mL) was added and the mixture was extracted with DCM (2 x 20 mL). The organic layers were gathered, washed with 1 N HCl and brine, dried over $MgSO_4$, filtered off and the solvents were removed under reduced pressure. Purification by column chromatography (SiO_2, pentane:EtOAc = 95:5 to 85:15) afforded benzyl (2S)-2-(*tert*-butyl)-4-ethyl-5-oxo-4-(3-phenylpropanamido)oxazolidine-3-carboxylate **11** (326 mg, 0.720 mmol, 48%) as a colorless sticky oil. **R_f** (pentane:EtOAc = 8:2) = 0.43. **^{1}H NMR** (400 MHz, $CDCl_3$) δ 9.11 (s, 1H, N*H*), 7.41 – 7.25 (m, 5H, 5 x Ar*H*), 7.22 (ddt, *J* = 8.2, 4.1, 1.9 Hz, 5H, 5 x Ar*H*), 5.62 (s, 1H, C*H*tBu), 5.21 (d, *J* = 11.9 Hz, 1H, CH_{aBn}), 4.94 (d, *J* = 11.9 Hz, 1H, CH_{bBn}), 2.94 (t, *J* = 7.8 Hz, 2H, CH_2), 2.88 – 2.68 (m, 2H, CH_2), 2.62 – 2.48 (m, 1H, CH_a), 2.41 (dq, *J* = 15.2, 7.7 Hz, 1H, CH_b), 1.11 (t, *J* = 15.1 Hz, 3H, CH_3), 1.01 (s, 9H, C(CH_3)$_3$). **^{13}C NMR** (101 MHz, $CDCl_3$) δ 167.7, 155.6, 140.4, 135.2, 129.2, 128.9, 128.8, 128.6, 126.7, 95.5, 86.6, 68.3, 38.4, 34.9, 30.3, 28.4, 25.7, 9.5. (2 C not resolved). IR (νmax, cm^{-1}) 3368 (w), 2968 (m), 2907 (w), 1797 (s), 1722 (s), 1523 (m), 1456 (m), 1390 (m), 1335 (m), 1288 (m). **HRMS** (ESI/QTOF) m/z: $[M+Na]^+$ Calcd for $C_{26}H_{32}N_2NaO_5^+$ 475.2203; Found 475.2200. $[\alpha]_D^{25}$ = -65.8 (c = 0.49, $CHCl_3$).

8. Stability of 3o and 8

Stability of 3o

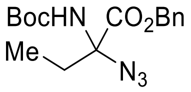

All test were performed using 0.01 mmol at a concentration of 0.05 M of **3e**. NMR recovery was measured using mesitylene as internal standard.

Table S2. Stability of 3o

Exact quantity (mg)	Solvent 1	V (mL)	Solvent 2	V (mL)	pH	Time (h)	NMR recovery (%)
3.29	CH_3CN	0.2	-	-		24	90
3.30	CH_3CN	0.2	-	-	-	48	92
3.30	CH_3CN	0.1	H_2O	0.1	-	24	90
3.32	CH_3CN	0.1	H_2O	0.1	-	48	88
3.45	DMSO	0.2	-	-	-	24	91
3.62	DMSO	0.2	-	-	-	48	89
3.30	DMSO	0.1	H_2O	0.1	-	24	94
3.30	DMSO	0.1	H_2O	0.1	-	48	98
3.42	CH_3CN	0.1	H_2O	0.1	4	48	92
3.23	CH_3CN	0.1	H_2O	0.1	7	48	92
4.05	CH_3CN	0.1	H_2O	0.1	9	48	91

Figure S1. Stability of 3o

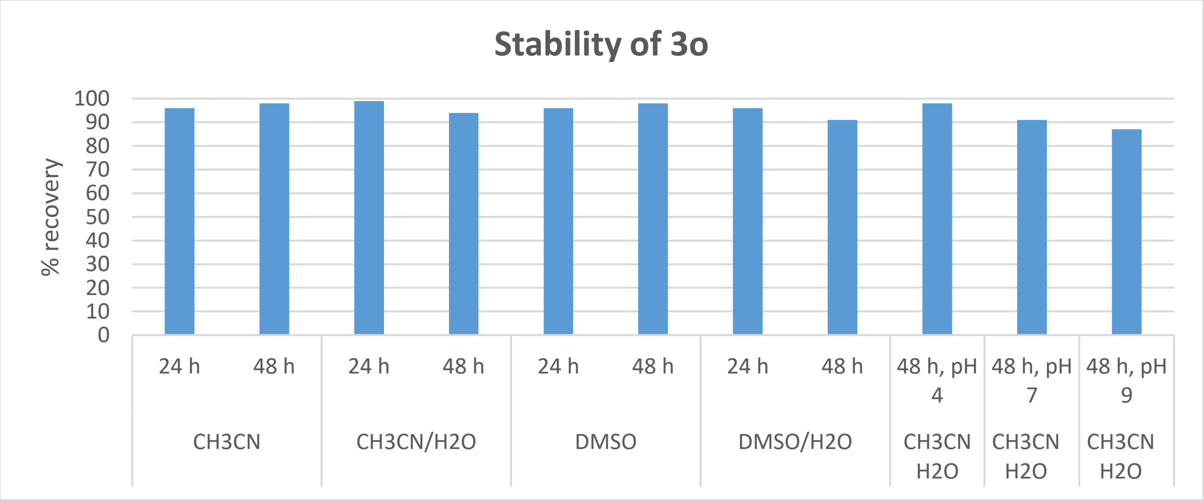

Stability of 8

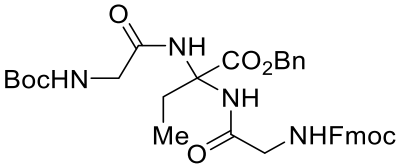

All test were performed using 0.005 mmol at a concentration of 0.05 M of **7**. NMR recovery was measured using mesitylene as internal standard.

Table S3. Stability of 8

Exact quantity (mg)	Solvent 1	V (mL)	Solvent 2	V (mL)	pH	Time (h)	NMR recovery (%)
3.28	CH_3CN	0.1	-	-		24	96
3.16	CH_3CN	0.1	-	-	-	48	98
3.08	CH_3CN	0.05	H_2O	0.05	-	24	99
3.44	CH_3CN	0.05	H_2O	0.05	-	48	94
3.45	DMSO	0.1	-	-	-	24	96
3.40	DMSO	0.1	-	-	-	48	98
3.23	DMSO	0.05	H_2O	0.05	-	24	96
3.11	DMSO	0.05	H_2O	0.05	-	48	91
3.62	CH_3CN	0.05	H_2O	0.05	4	48	98
3.23	CH_3CN	0.05	H_2O	0.05	7	48	91
3.23	CH_3CN	0.05	H_2O	0.05	9	48	87

Figure S2. Stability of 8

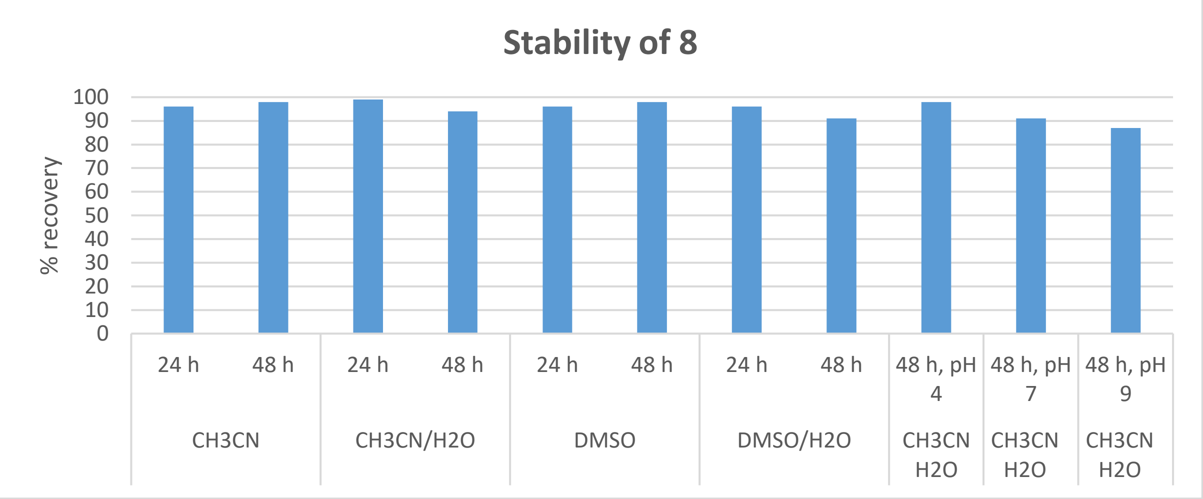

9. Crystal structures

7.1 Crystal structure of methyl 2-azido-2-(bis(tert-butoxycarbonyl)amino)-3-cyclopropylpropanoate 3x

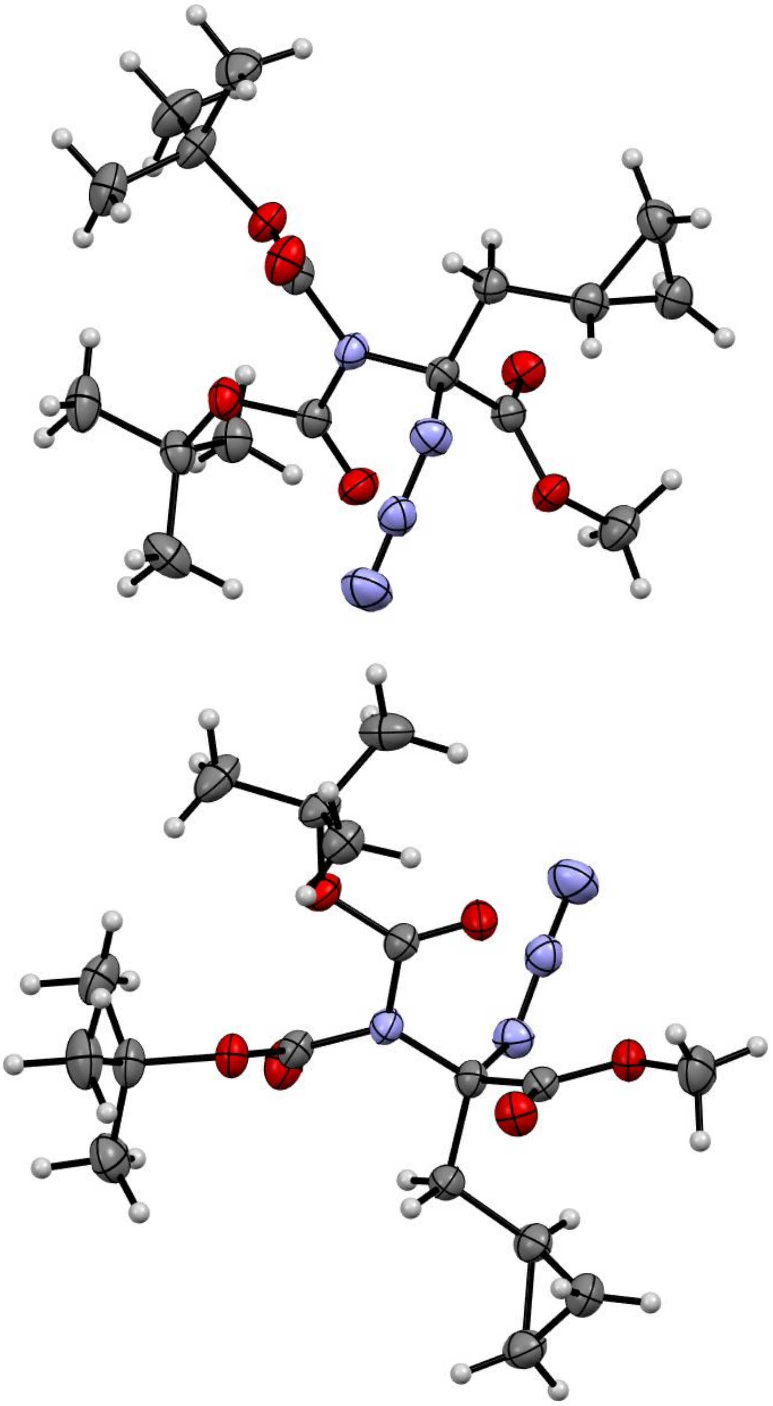

Figure S3: Ellipsoid plot (probability level 50%) of **3x**

**Compound**	**3x**
Formula	$C_{17}H_{28}N_4O_6$
$D_{calc.}$/ g cm^{-3}	1.266
μ/mm^{-1}	0.806
Formula Weight	384.43
Colour	clear pale colourless
Shape	prism-shaped
Size/mm^3	0.40×0.18×0.12
T/K	140.00(10)
Crystal System	triclinic
Space Group	P-1
a/Å	9.00727(14)
b/Å	9.2679(2)
c/Å	13.3222(3)
α/°	98.4195(17)
β/°	95.6229(15)
γ/°	111.6414(17)
V/Å^3	1008.52(4)
Z	2
Z'	1
Wavelength/Å	1.54184
Radiation type	Cu K$_\alpha$
Θ_{min}/°	3.400
Θ_{max}/°	75.658
Measured Refl's.	21589
Indep't Refl's	4100
Refl's I≥2 σ(I)	3669
R_{int}	0.0246
Parameters	357
Restraints	9
Largest Peak	0.302
Deepest Hole	-0.196
GooF	1.056
wR_2 (all data)	0.1070
wR_2	0.1043
R_1 (all data)	0.0419

Crystals were grown by preparing a solution of **3x** in MeOH, leaving the solution slowly evaporate over 2-3 days.

Analysis of the crystal: A suitable crystal with dimensions 0.40 × 0.18 × 0.12 mm^3 was selected and mounted on a XtaLAB Synergy R, DW system, HyPix-Arc 150 diffractometer. The crystal was kept at a steady T = 140.00(10) K during data collection. The structure was solved with the **ShelXT** (Sheldrick, 2015) solution program using dual methods and by using **Olex2** 1.5 (Dolomanov et al., 2009) as the graphical interface. The model was refined with **ShelXL** 2018/3 (Sheldrick, 2015) using full matrix least squares minimisation on **F^2**.

Supplementary crystallographic data for this compound have been deposited at Cambridge Crystallographic Data Centre (CCDC 2263395) and can be obtained free of charge via www.ccdc.cam.ac.uk/data_request/cif.

7.2 Crystal structure of methyl 3-((3r,5r,7r)-adamantan-1-yl)-2-azido-2-(bis(tert-butoxycarbonyl)amino) propanoate 3ad

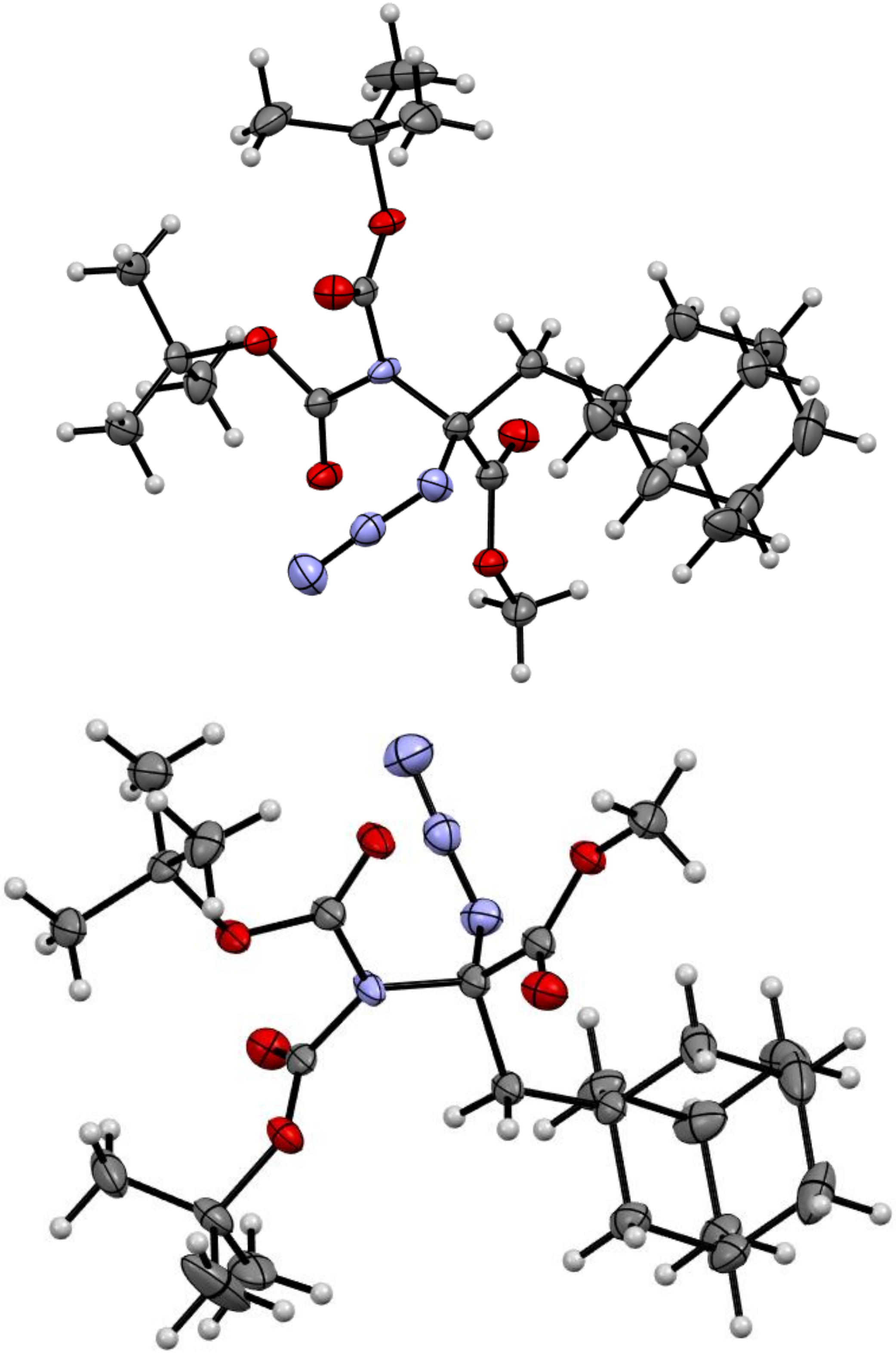

Figure S4: Ellipsoid plot (probability level 50%) of **3ad**

Compound	3ad
Formula	$C_{24}H_{38}N_4O_6$
$D_{calc.}$/ g cm^{-3}	1.257
μ/mm^{-1}	0.743
Formula Weight	478.58
Colour	clear pale colourless
Shape	irregular-shaped
Size/mm^3	0.39×0.27×0.26
T/K	140.01(10)
Crystal System	trigonal
Space Group	R-3
a/Å	19.8294(5)
b/Å	19.8294(5)
c/Å	33.4154(9)
α/°	90
β/°	90
γ/°	120
V/Å^3	11378.8(6)
Z	18
Z'	1
Wavelength/Å	1.54184
Radiation type	Cu K_α
Θ_{min}/°	2.893
Θ_{max}/°	72.334
Measured Refl's.	10079
Indep't Refl's	4853
Refl's I≥2 σ(I)	4096
R_{int}	0.0290
Parameters	315
Restraints	0
Largest Peak	0.603
Deepest Hole	-0.287
GooF	1.072
wR_2 (all data)	0.1793
wR_2	0.1705
R_1 (all data)	0.0727

Crystals were grown by preparing a solution of **3ad** in MeOH, leaving the solution slowly evaporate over 2-3 days.

Analysis of the crystal: A suitable crystal with dimensions 0.39 × 0.27 × 0.26 mm^3 was selected and mounted on a SuperNova, Dual, Cu at home/near, Atlas diffractometer. The crystal was kept at a steady T = 140.01(10) K during data collection. The structure was solved with the **ShelXT** (Sheldrick, 2015) solution program using dual methods and by using **Olex2** 1.5 (Dolomanov et al., 2009) as the graphical interface. The model was refined with **ShelXL** 2018/3 (Sheldrick, 2015) using full matrix least squares minimisation on **F^2**.

Supplementary crystallographic data for this compound have been deposited at Cambridge Crystallographic Data Centre (CCDC 2263396) and can be obtained free of charge via www.ccdc.cam.ac.uk/data_request/cif.

9.3 Conformational locking analysis

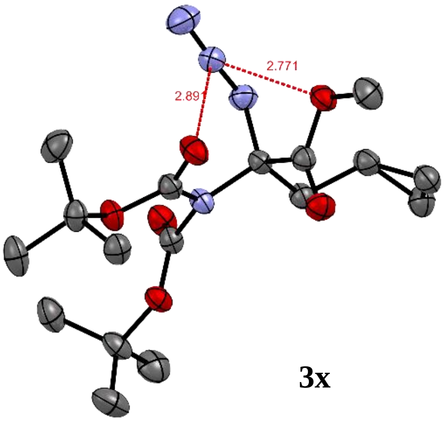

3x

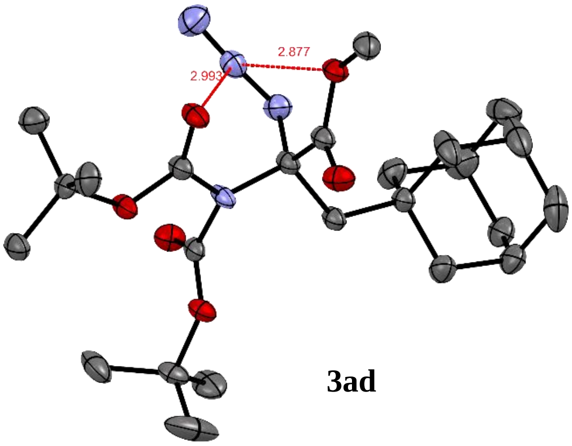

3ad

Figure S5: Crystal structure of **3x** and **3ad** showing conformational locking through N-O interaction

10.References

[1] C. Hall, H. Wolfe, A. Wells, H.-C. Chien, C. Colas, A. Schlessinger, K. M. Giacomini, A. A. Thomas, *Bioorg. Med. Chem. Lett.* **2019**, *29*, 2254–2258.

[2] F. Friscourt, C. J. Fahrni, G.-J. Boons, *J. Am. Chem. Soc.* **2012**, *134*, 18809–18815.

[3] N. Sabat, F. Soualmia, P. Retailleau, A. Benjdia, O. Berteau, X. Guinchard, *Org. Lett.* **2020**, *22*, 4344–4349.

[4] J.-A. Shin, J. Kim, H. Lee, S. Ha, H.-Y. Lee, *J. Org. Chem.* **2019**, *84*, 4558–4565.

[5] I. S. Kondratov, M. Ya. Bugera, N. A. Tolmachova, C. G. Daniliuc, G. Haufe, *J. Fluor. Chem.* **2018**, *211*, 100–108.

[6] F. Zhang, W. Zhang, Y. Zhang, D. P. Curran, G. Liu, *J. Org. Chem.* **2009**, *74*, 2594–2597.

[7] S. Koch, D. Schollmeyer, H. Löwe, H. Kunz, *Chem. - Eur. J.* **2013**, *19*, 7020–7041.

[8] G. Occhialini, V. Palani, A. E. Wendlandt, *J Am Chem Soc* **2022**, 8.

[9] R. Petracca, K. A. Bowen, L. McSweeney, S. O'Flaherty, V. Genna, B. Twamley, M. Devocelle, E. M. Scanlan, *Org. Lett.* **2019**, *21*, 3281–3285.

[10] S. B. Vogensen, R. P. Clausen, J. R. Greenwood, T. N. Johansen, D. S. Pickering, B. Nielsen, B. Ebert, P. Krogsgaard-Larsen, *J. Med. Chem.* **2005**, *48*, 3438–3442.

[11] P. M. T. Ferreira, H. L. S. Maia, L. S. Monteiro, J. Sacramento, *J. Chem. Soc. Perkin 1* **1999**, 3697–3703.

[12] S. Heinrich, M. Altenkämper, B. Bechem, J. Perruchon, R. Ortmann, H.-M. Dahse, Y. Wang, M. Lanzer, M. Schlitzer, *Eur. J. Med. Chem.* **2011**, *46*, 1331–1342.

[13] D. Reich, A. Trowbridge, M. J. Gaunt, *Angew. Chem. Int. Ed.* **2020**, *59*, 2256–2261.

[14] L. Ge, W. Jian, H. Zhou, S. Chen, C. Ye, F. Yu, B. Qian, Y. Li, H. Bao, *Chem. - Asian J.* **2018**, *13*, 2522–2528.

[15] A. Gómez-Palomino, M. Pérez-Palau, P. Romea, F. Urpí, M. Del Olmo, T. Hesse, S. Fleckenstein, E. Gómez-Bengoa, L. Sotorríos, M. Font-Bardia, *Org. Lett.* **2020**, *22*, 199–203.

[16] H. Tian, W. Xu, Y. Liu, Q. Wang, *Chem. Commun.* **2019**, *55*, 14813–14816.

[17] S.-M. Hyun, M. Yuan, A. Maity, O. Gutierrez, D. C. Powers, *Chem* **2019**, *5*, 2388–2404.

[18] C. Ye, B. Qian, Y. Li, M. Su, D. Li, H. Bao, *Org. Lett.* **2018**, *20*, 3202–3205.

[19] M. A. Rizvi, S. Guru, T. Naqvi, M. Kumar, N. Kumbhar, S. Akhoon, S. Banday, S. K. Singh, S. Bhushan, G. Mustafa Peerzada, et al., *Bioorg. Med. Chem. Lett.* **2014**, *24*, 3440–3446.

[20] R. Wei, H. Xiong, C. Ye, Y. Li, H. Bao, *Org. Lett.* **2020**, *22*, 3195–3199.

[21] A. Charafeddine, H. Chapuis, P. Strazewski, *Org. Lett.* **2007**, *9*, 2787–2790.

[22] B. Ardiansah, H. Tanimoto, T. Tomohiro, T. Morimoto, K. Kakiuchi, *Chem. Commun.* **2021**, *57*, 8738–8741.

[23] J. Burés, M. Martín, F. Urpí, J. Vilarrasa, *J. Org. Chem.* **2009**, *74*, 2203–2206.

11.NMR spectra

^{1}H NMR (400 MHz, $CDCl_3$) of compound **S4**

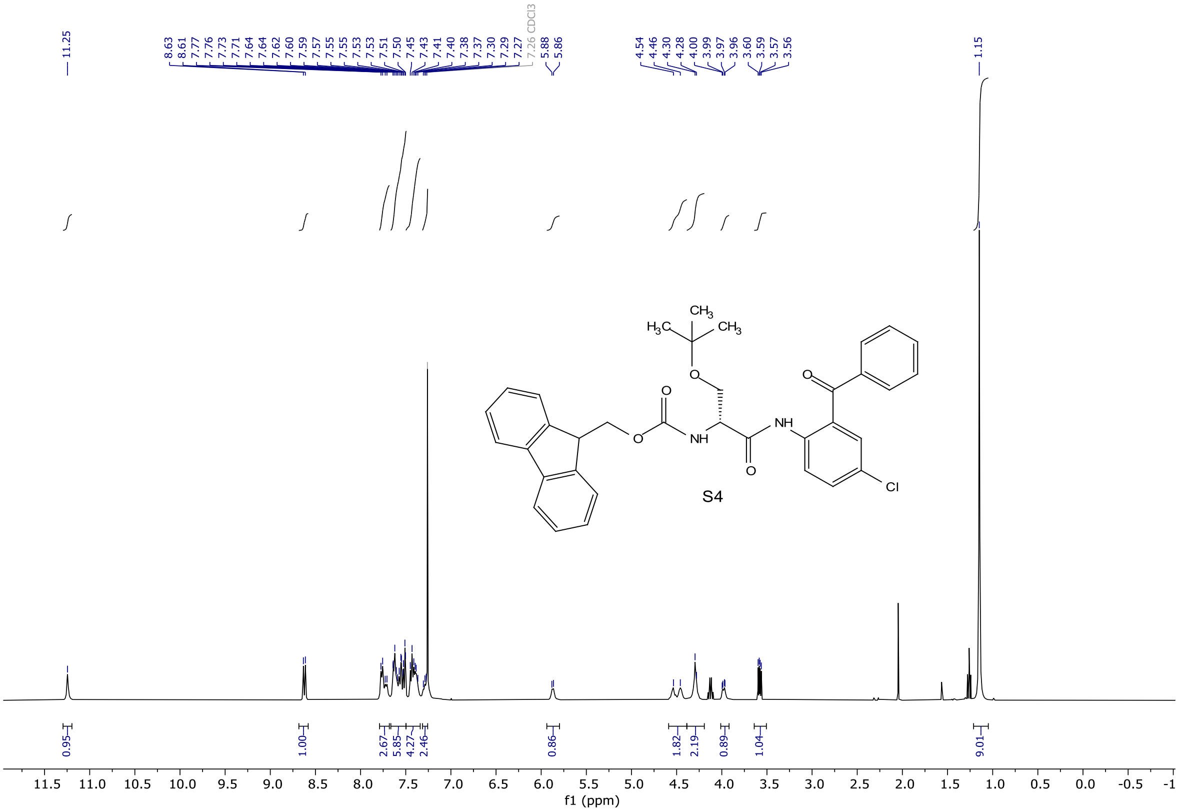

^{13}C NMR (400 MHz, $CDCl_3$) of compound **S4**

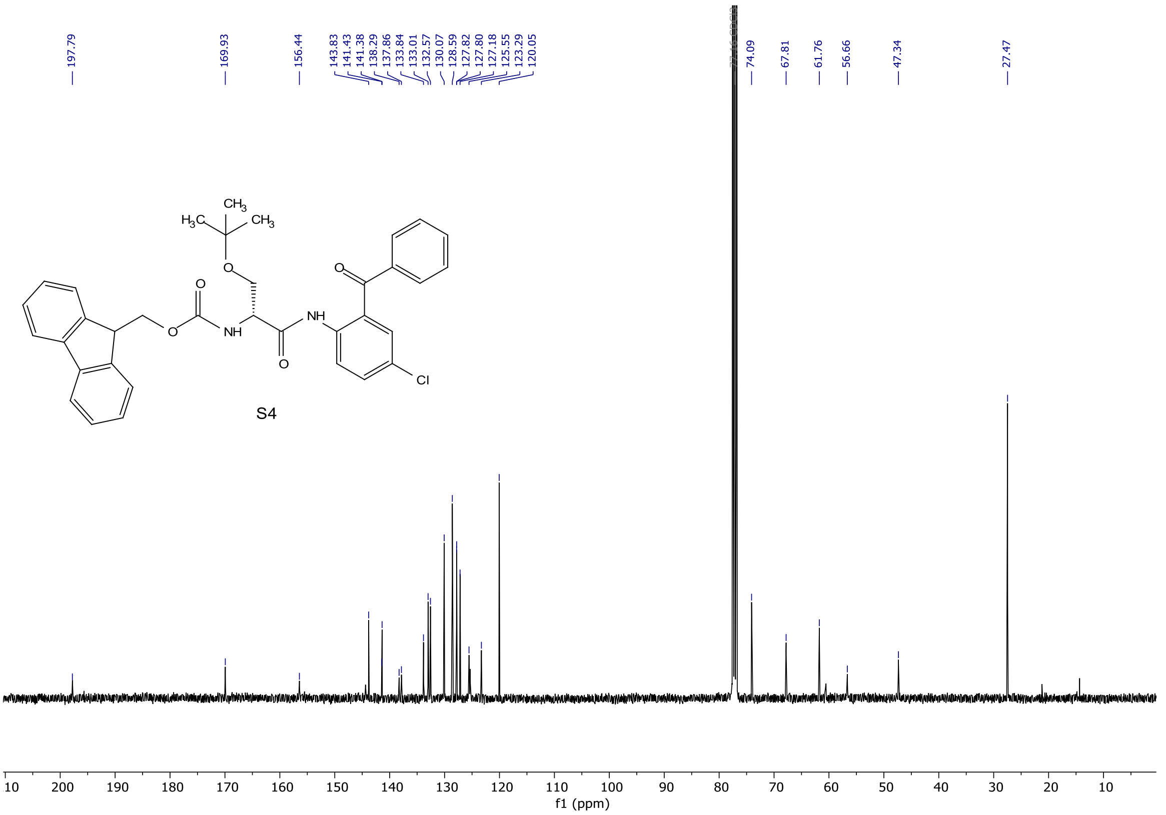

^{1}H NMR (400 MHz, $CDCl_3$) of compound **1i**

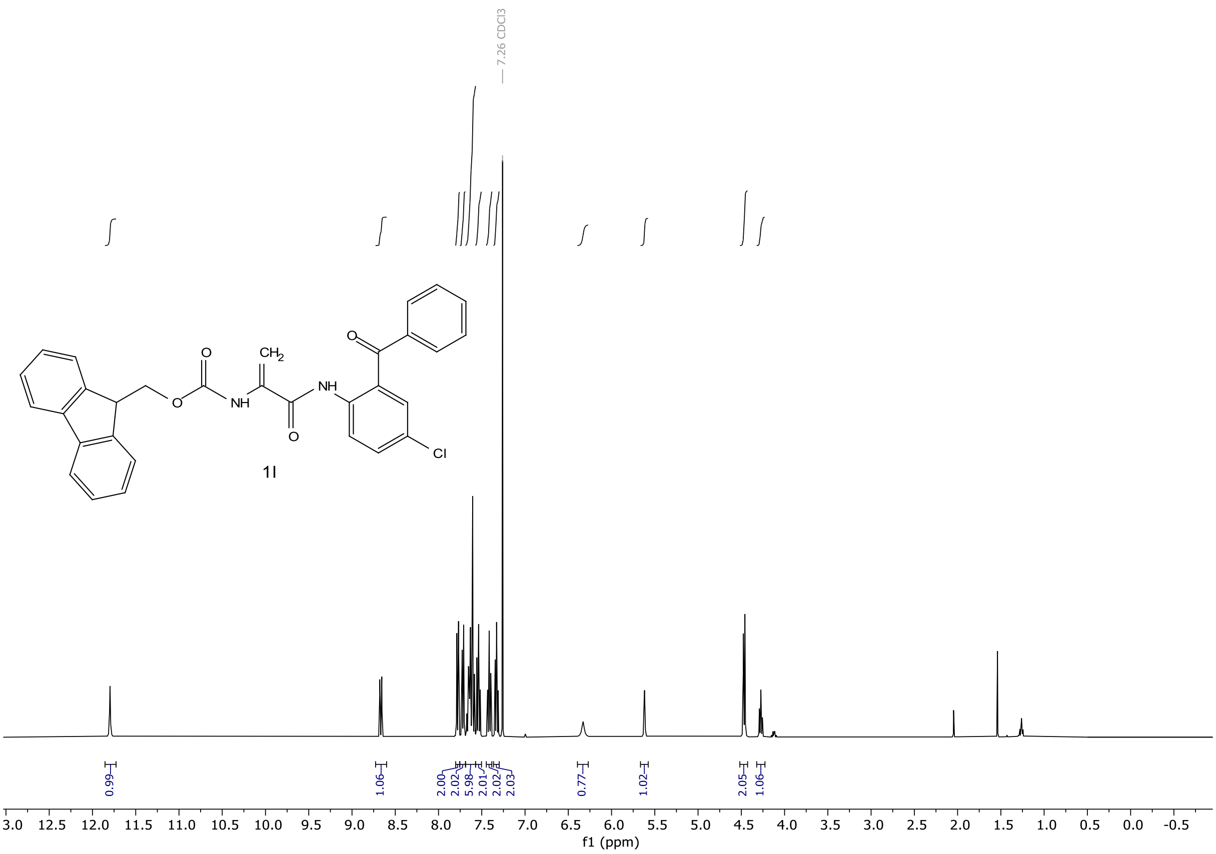

^{13}C NMR (400 MHz, $CDCl_3$) of compound **1i**

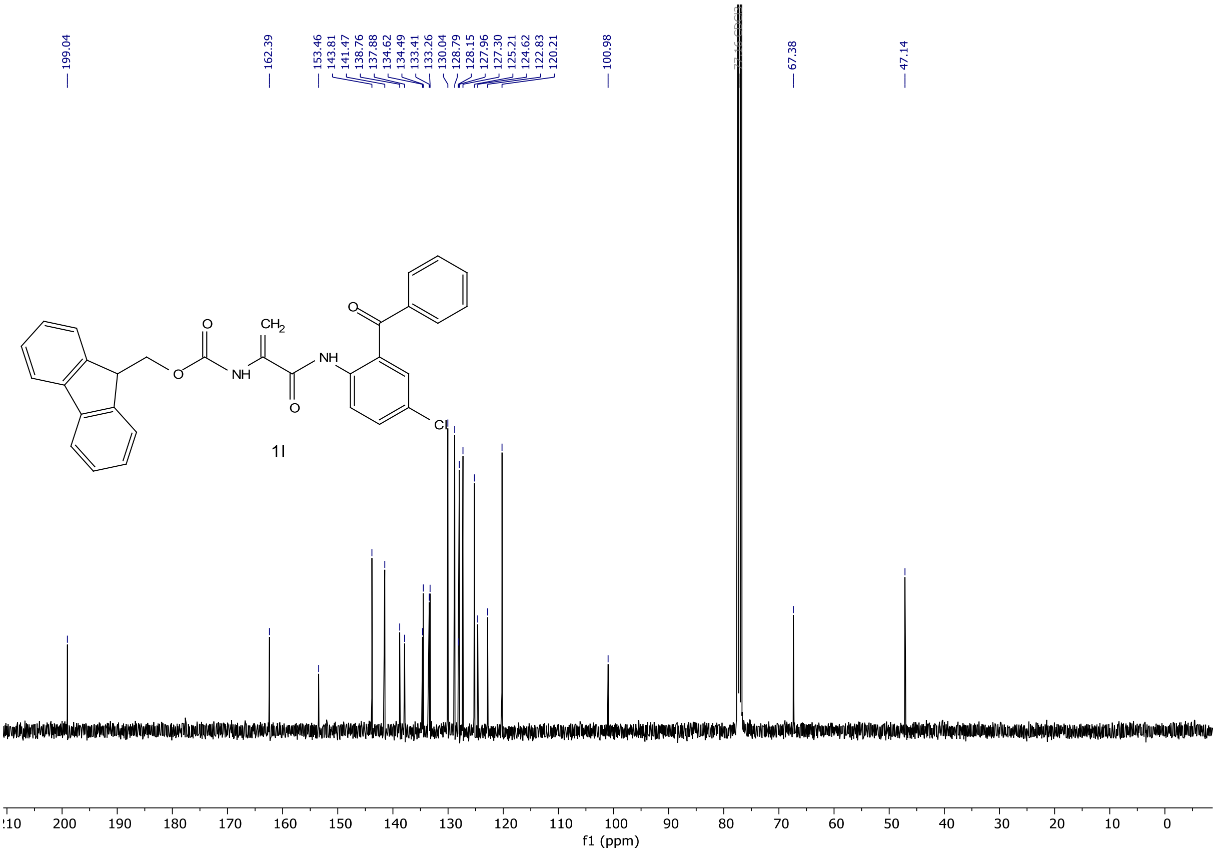

^{1}H NMR (400 MHz, $CDCl_3$) of compound **2g**

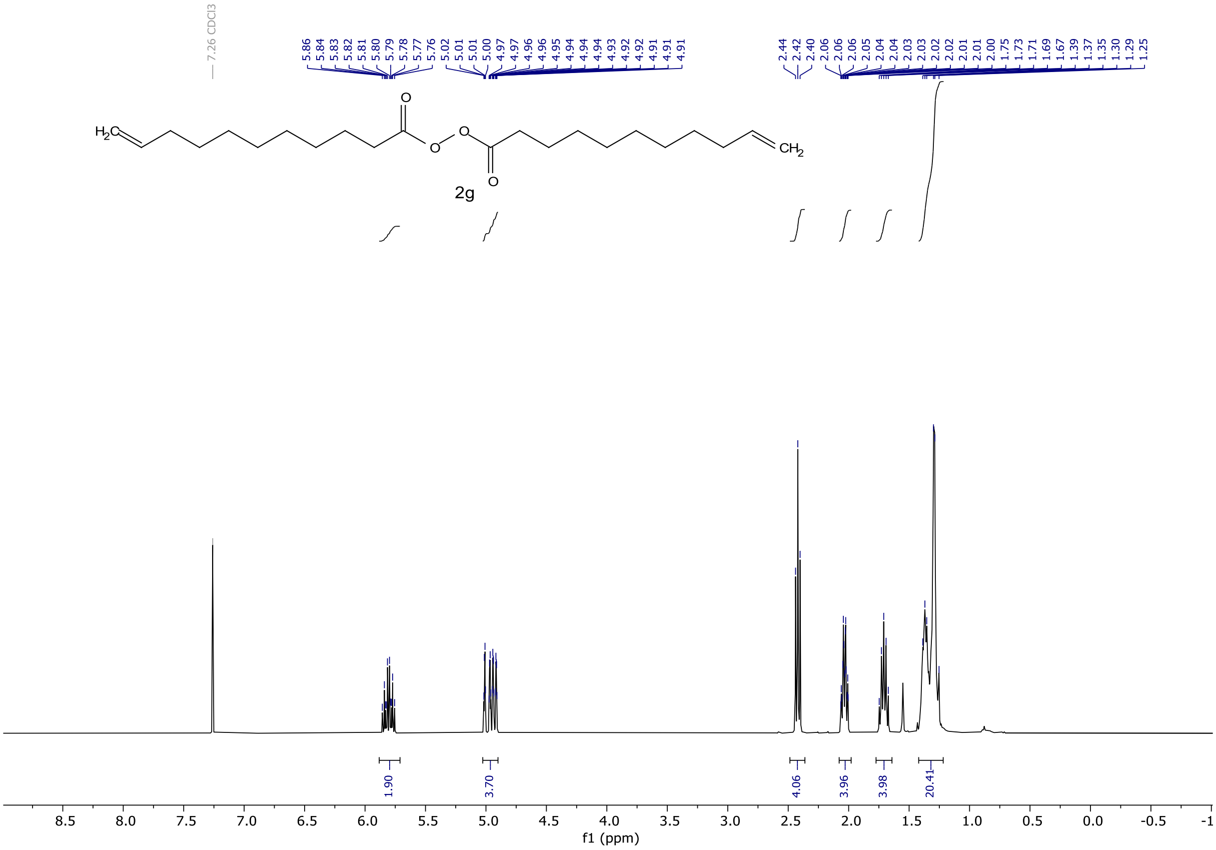

^{13}C NMR (400 MHz, $CDCl_3$) of compound **2g**

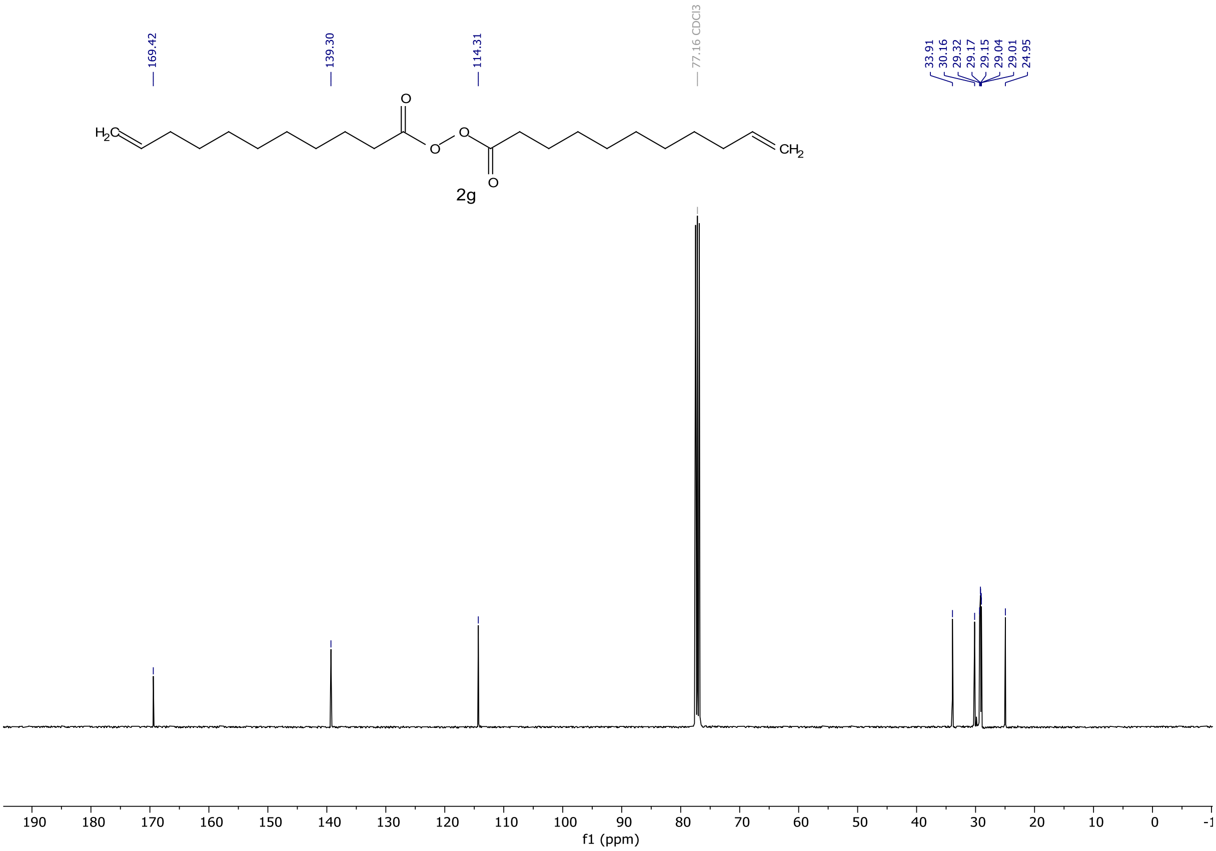

^{1}H NMR (400 MHz, $CDCl_3$) of compound **2h**

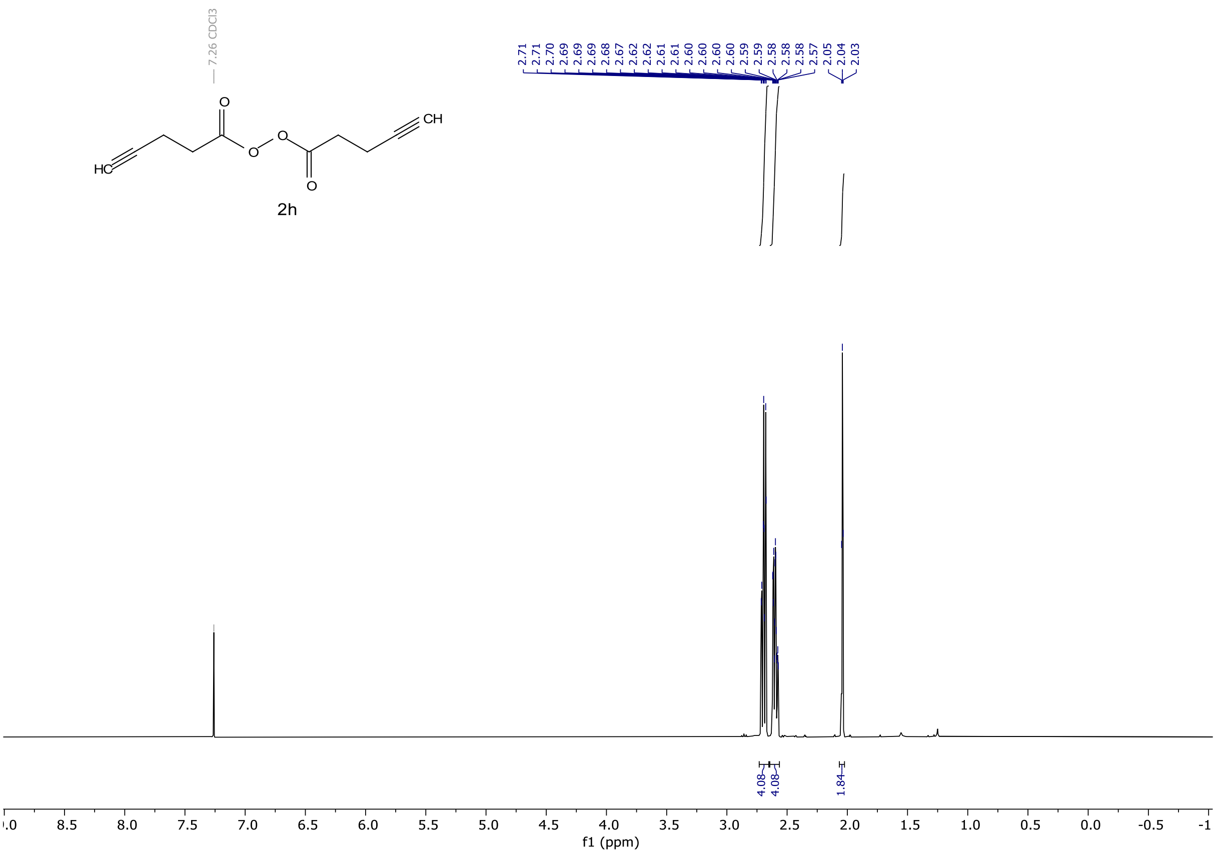

^{13}C NMR (400 MHz, $CDCl_3$) of compound **2h**

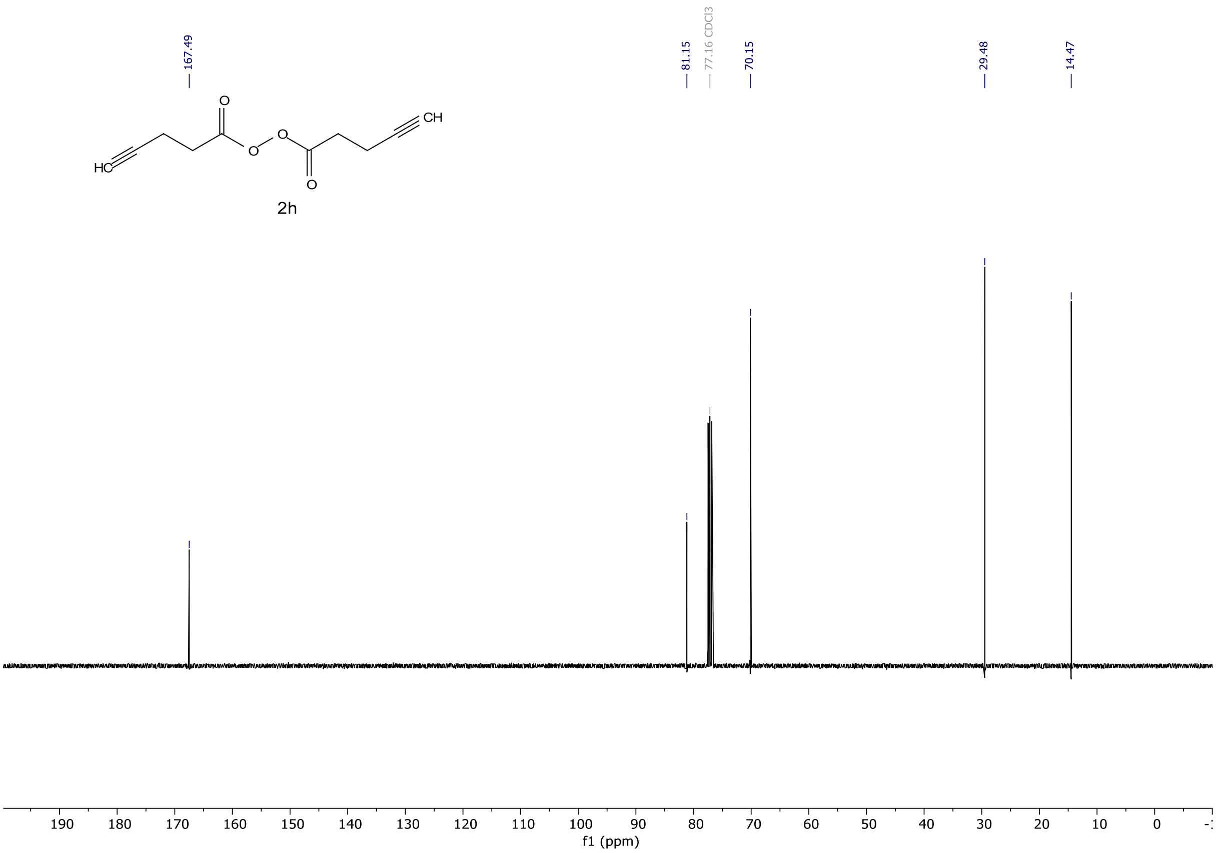

^{1}H NMR (400 MHz, $CDCl_3$) of compound **2p**

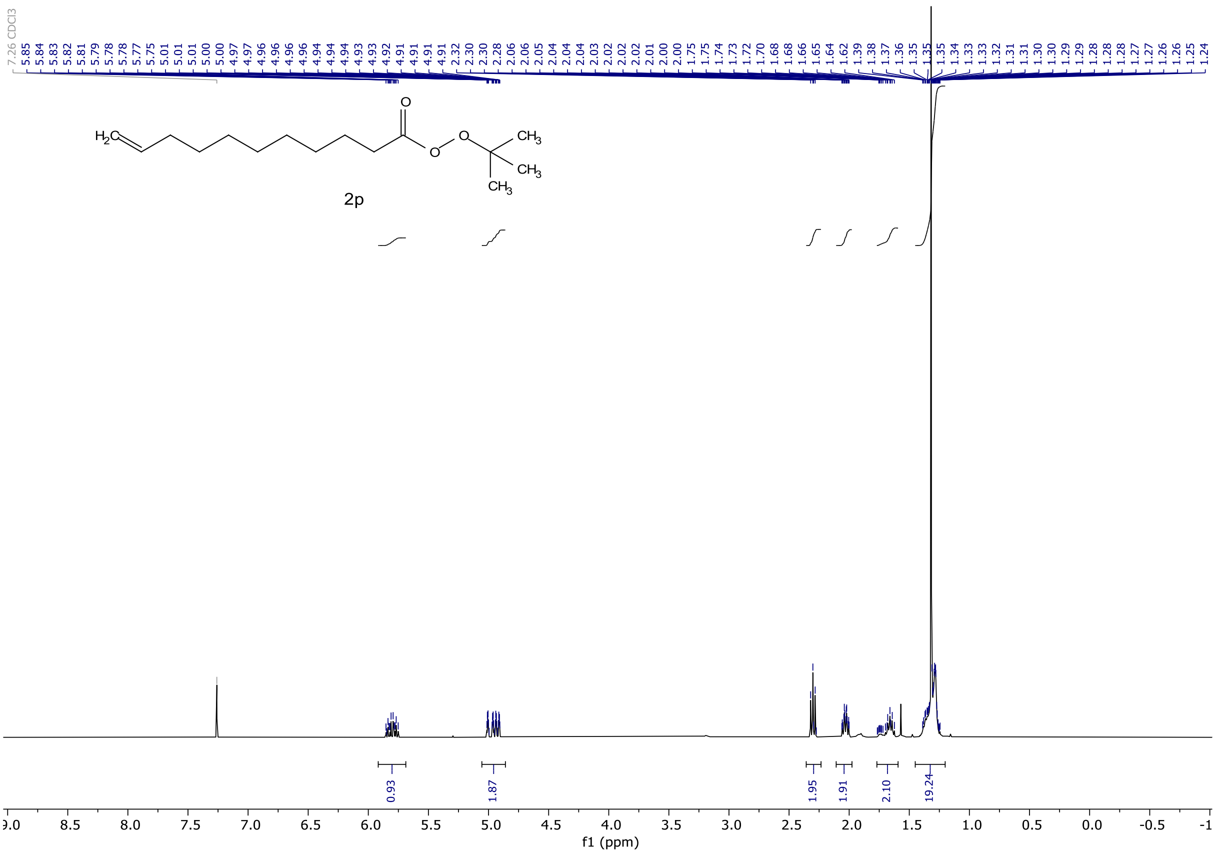

^{13}C NMR (400 MHz, $CDCl_3$) of compound **2p**

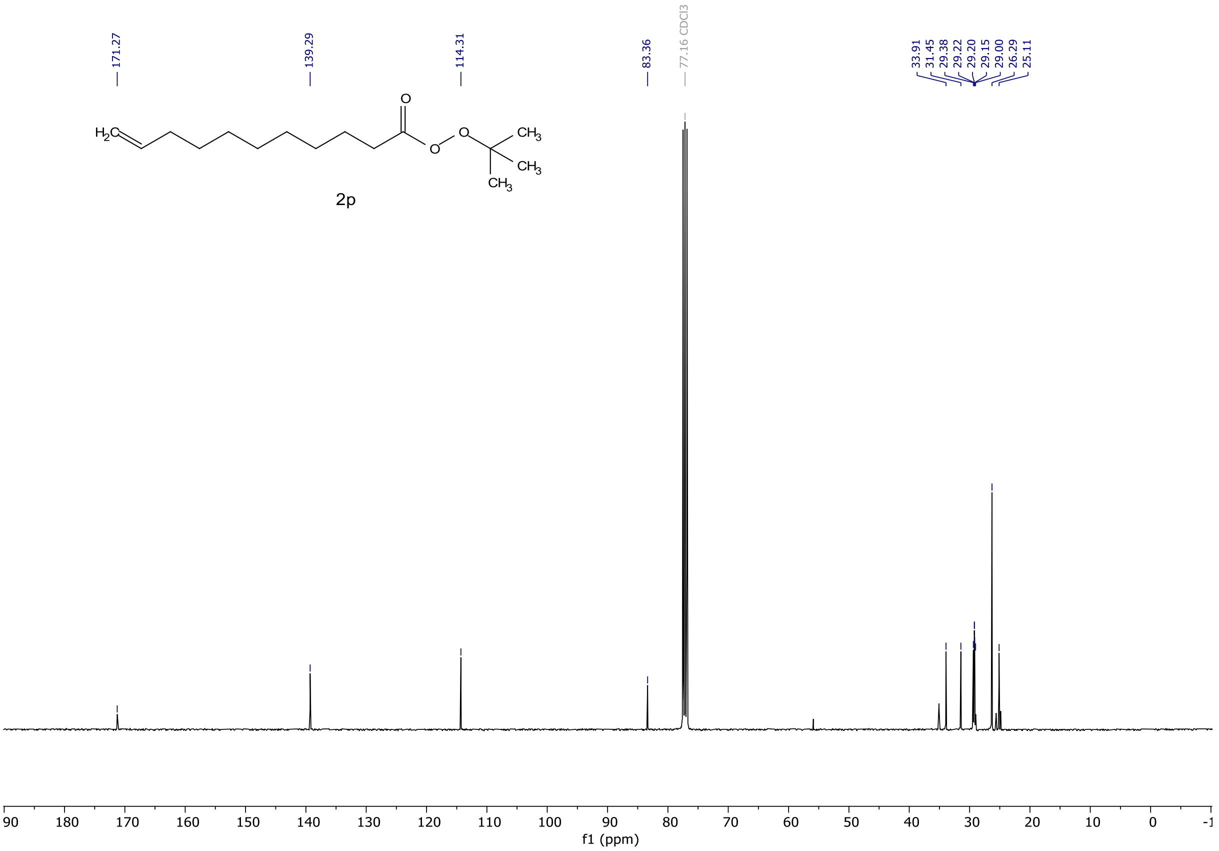

^{1}H NMR (400 MHz, $CDCl_3$) of compound **2r**

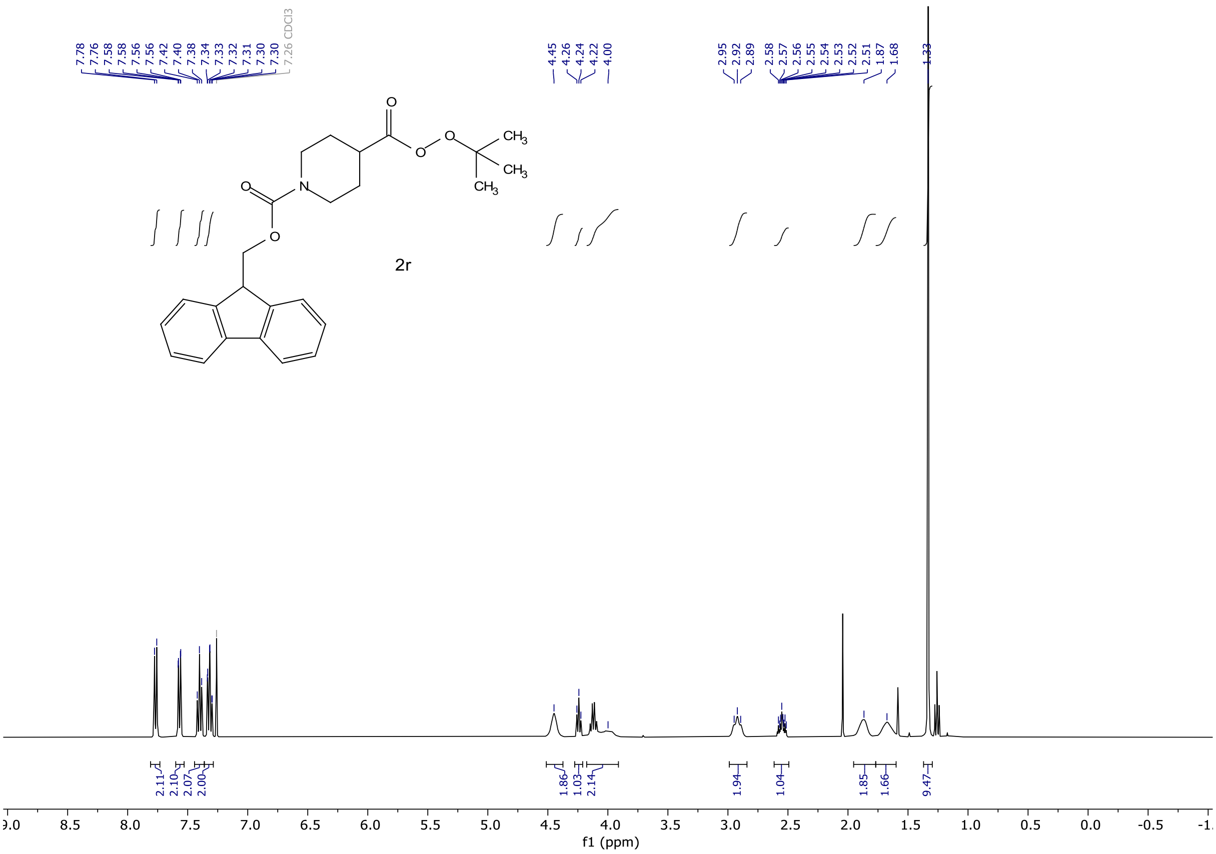

^{13}C NMR (400 MHz, $CDCl_3$) of compound **2r**

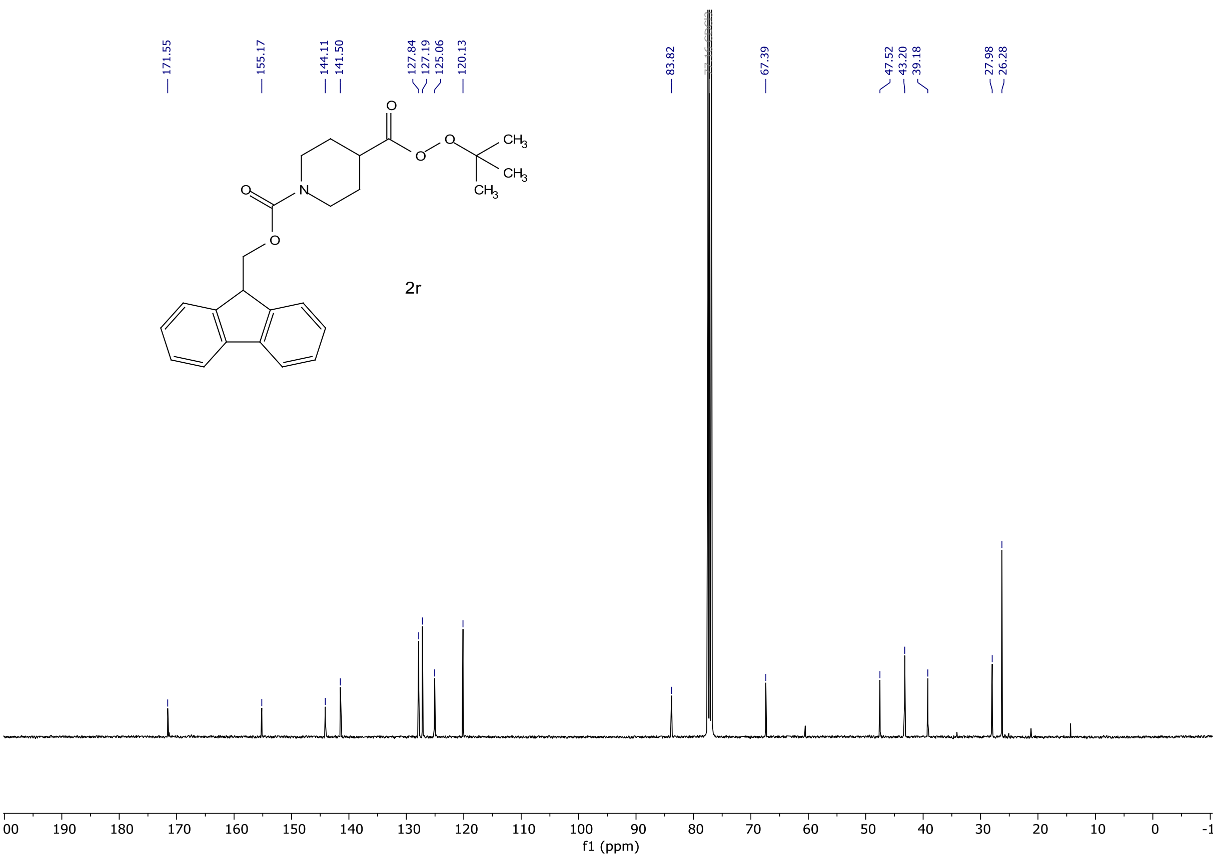

^{1}H NMR (400 MHz, $CDCl_3$) of compound **3a**

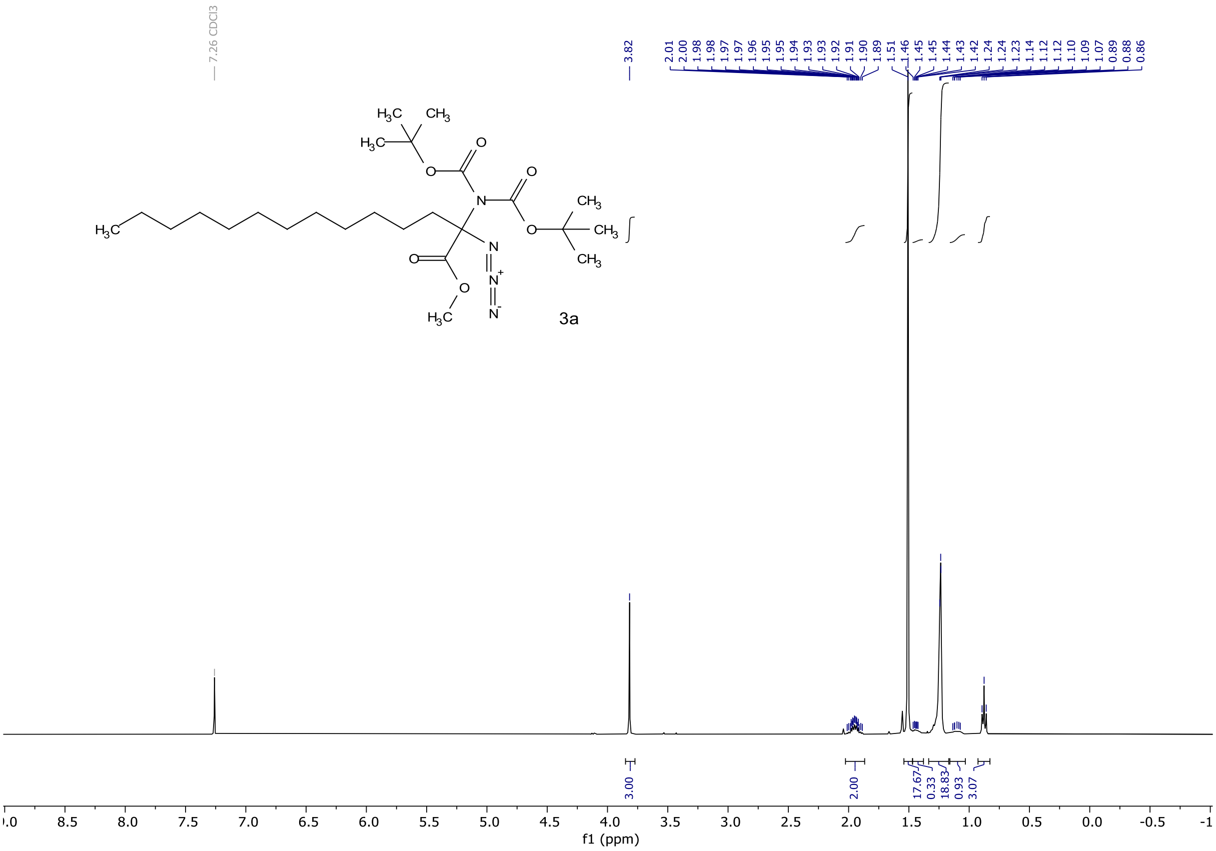

^{13}C NMR (400 MHz, $CDCl_3$) of compound **3a**

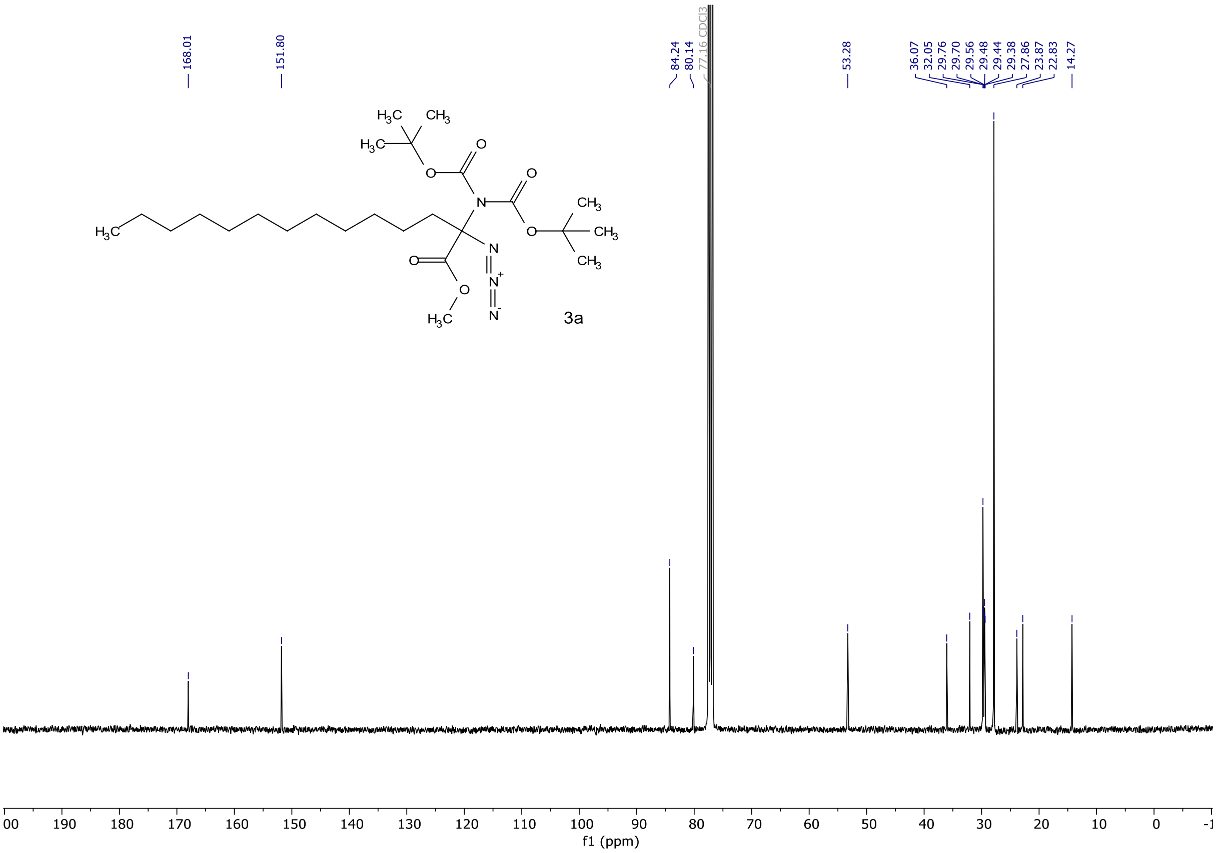

^{1}H NMR (400 MHz, $CDCl_3$) of compound **3b**

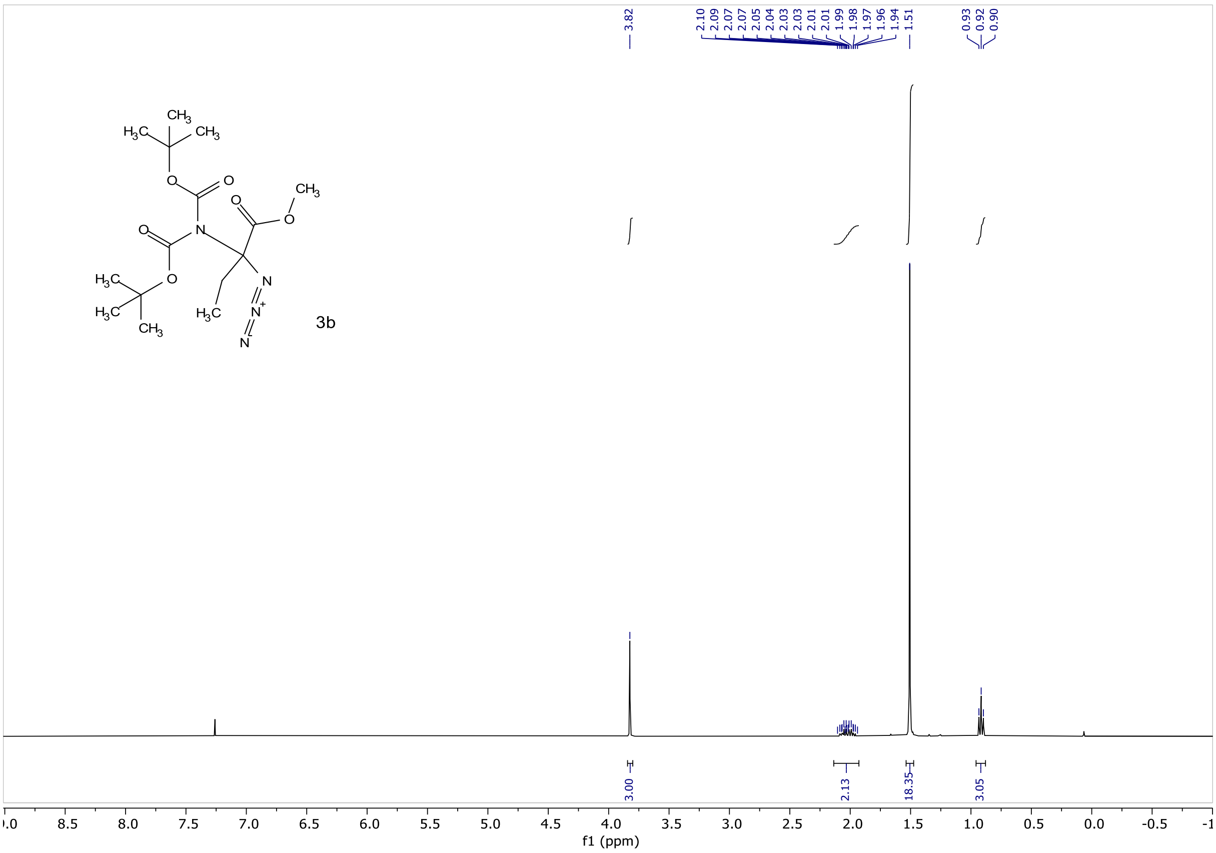

^{13}C NMR (400 MHz, $CDCl_3$) of compound **3b**

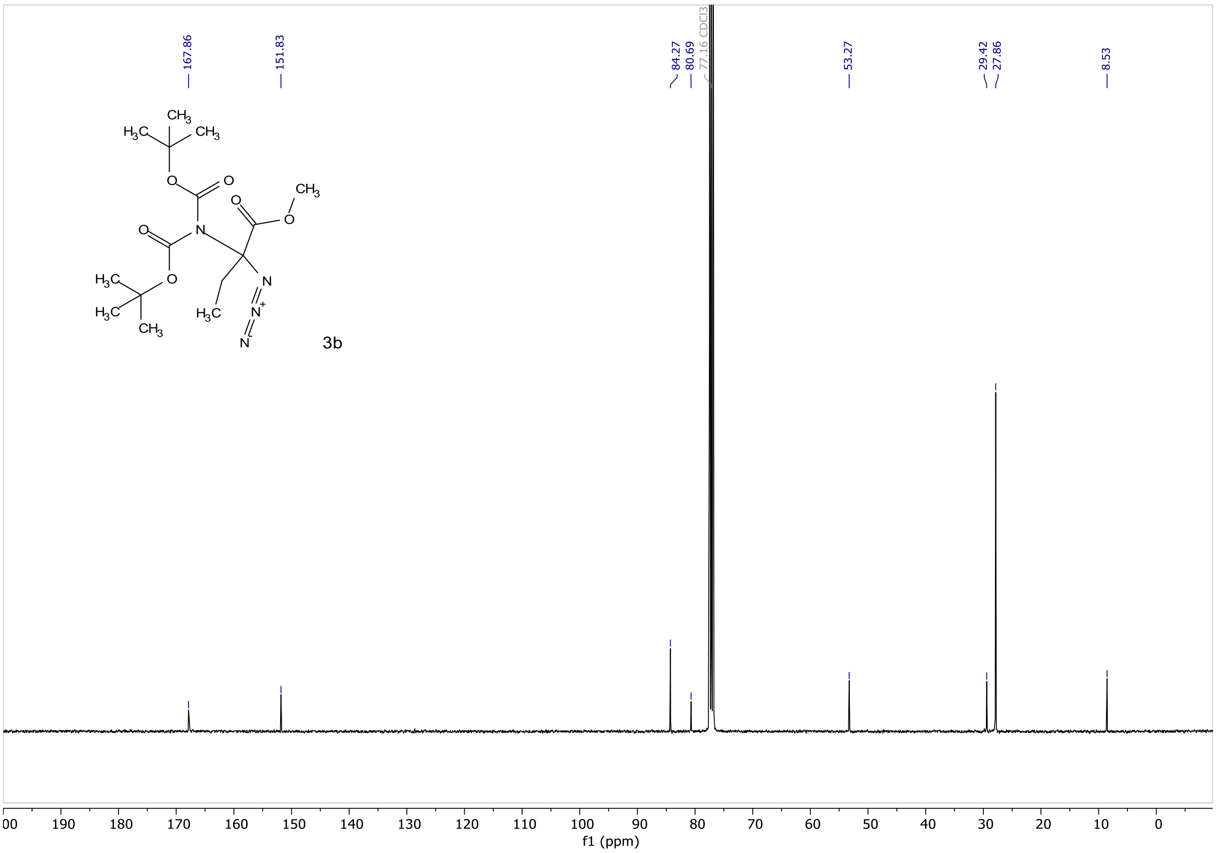

^{1}H NMR (400 MHz, $CDCl_3$) of compound **3c**

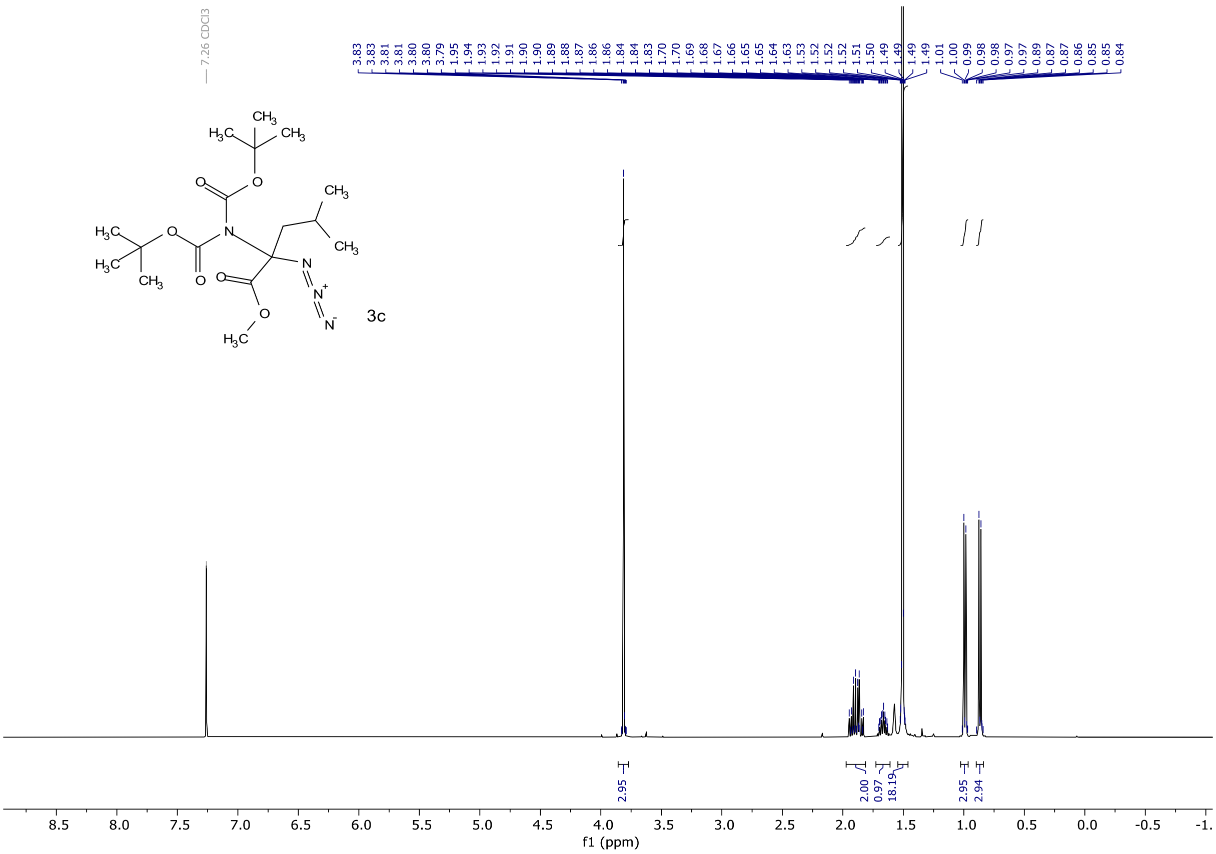

^{13}C NMR (400 MHz, $CDCl_3$) of compound **3c**

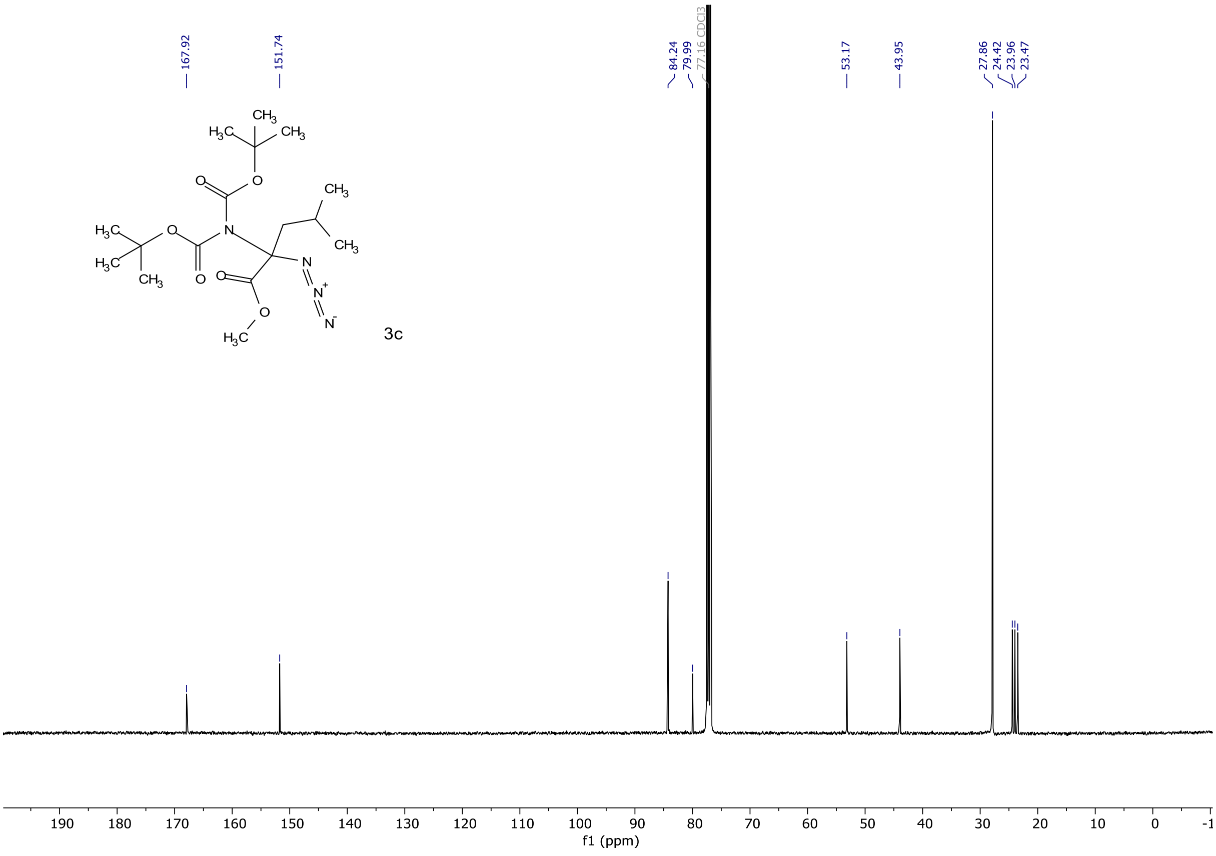

^{1}**H NMR** (400 MHz, $CDCl_3$) of compound **3d**

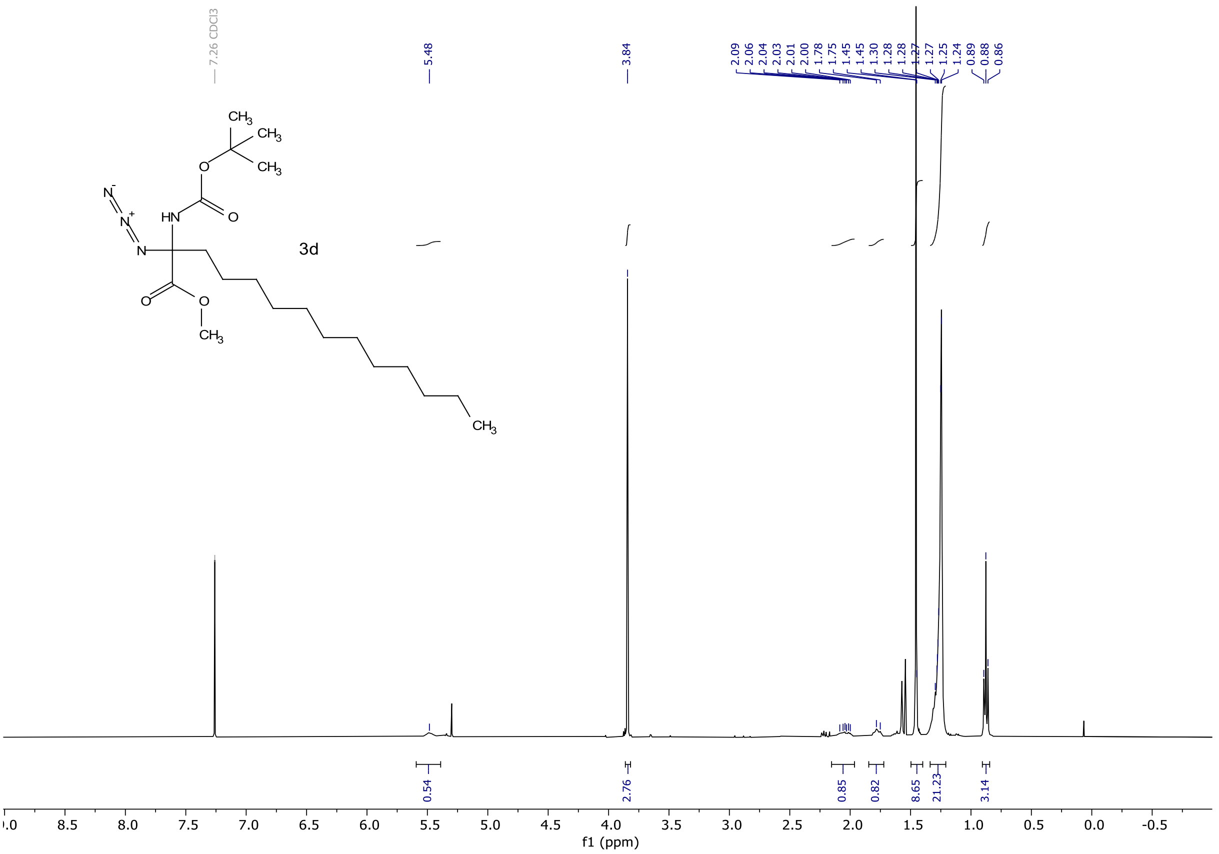

^{13}**C NMR** (400 MHz, $CDCl_3$) of compound **3d**

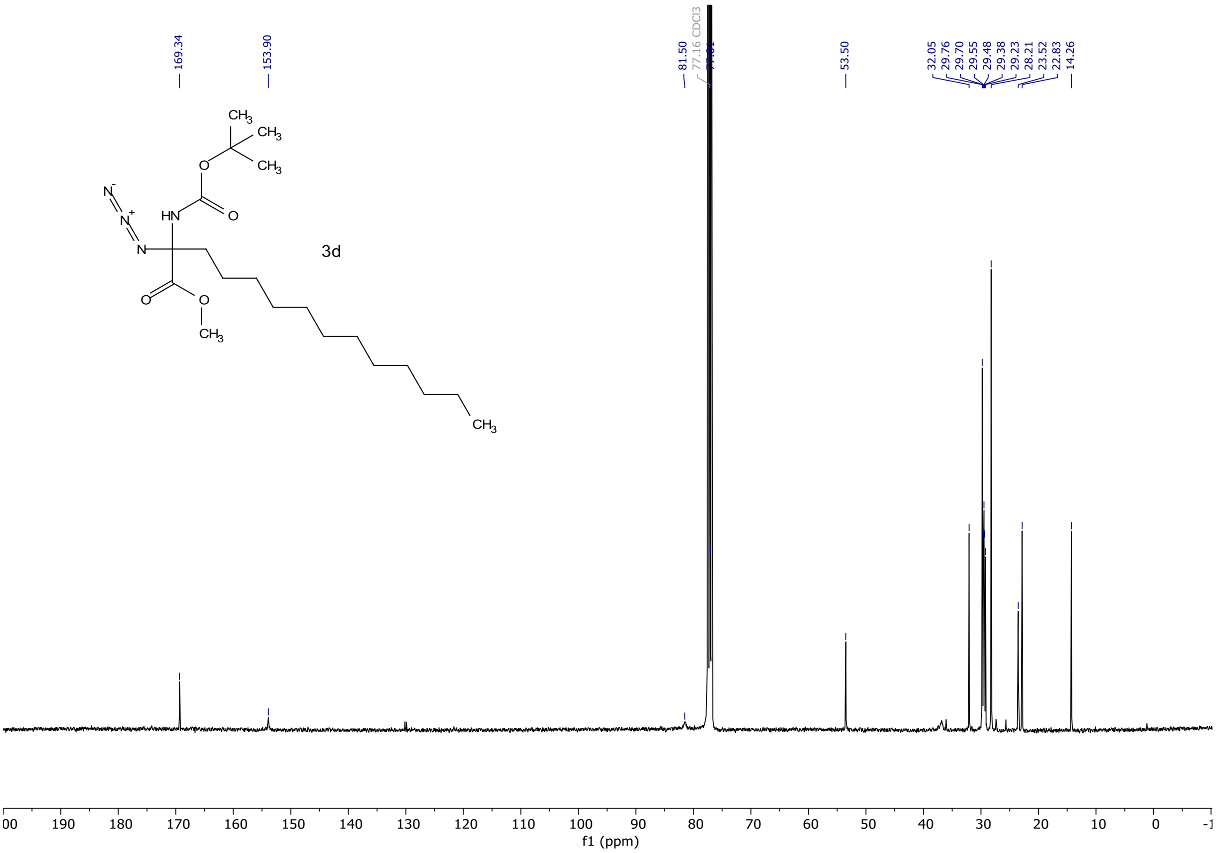

¹H NMR (400 MHz, $CDCl_3$) of compound **3e**

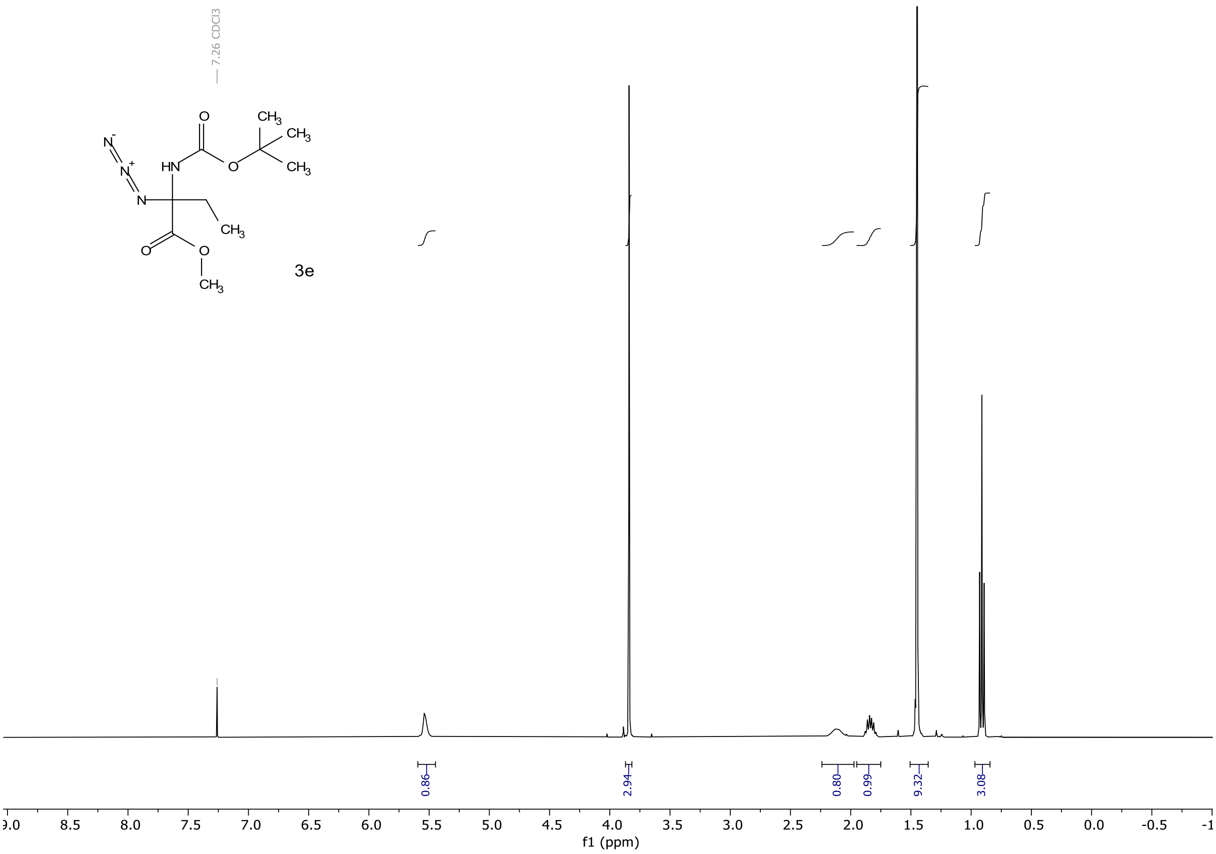

¹³C NMR (400 MHz, $CDCl_3$) of compound **z**

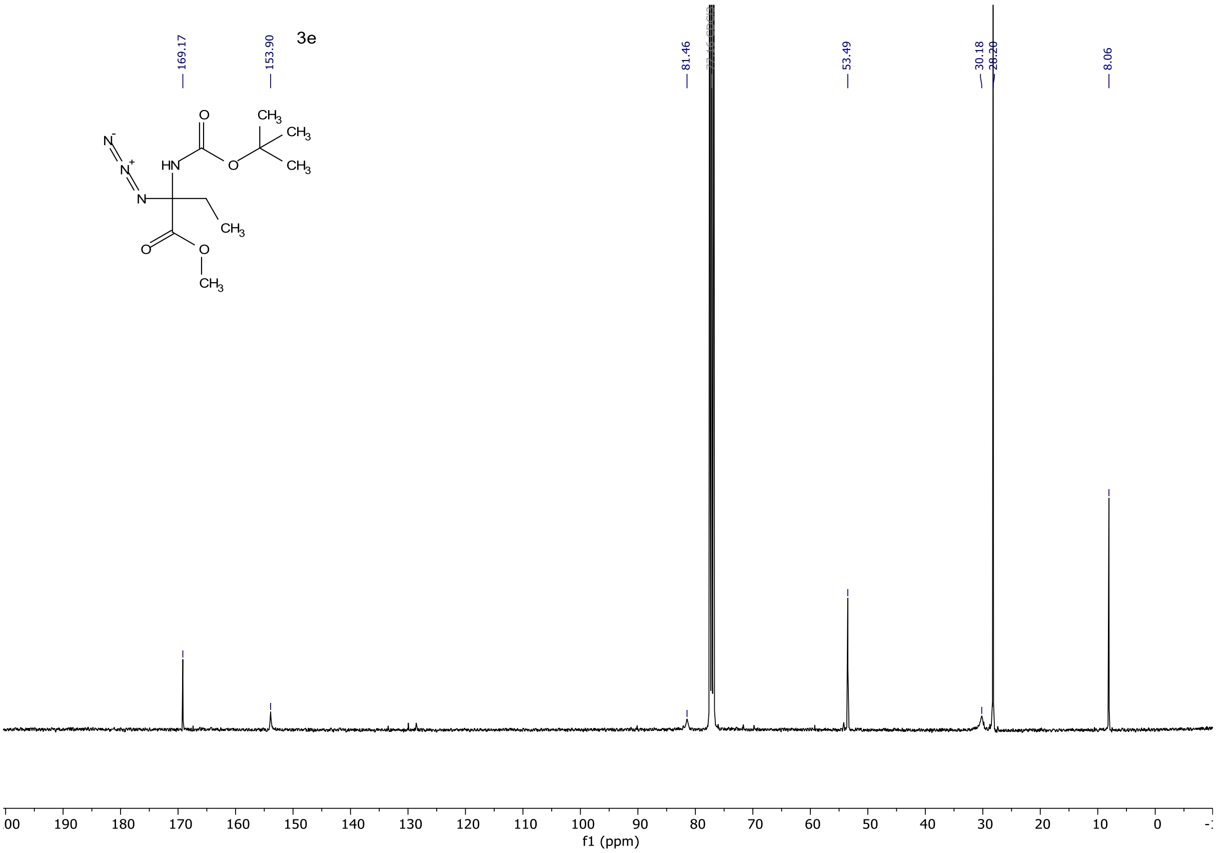

HMBC (400 MHz, $CDCl_3$) of compound **3e**

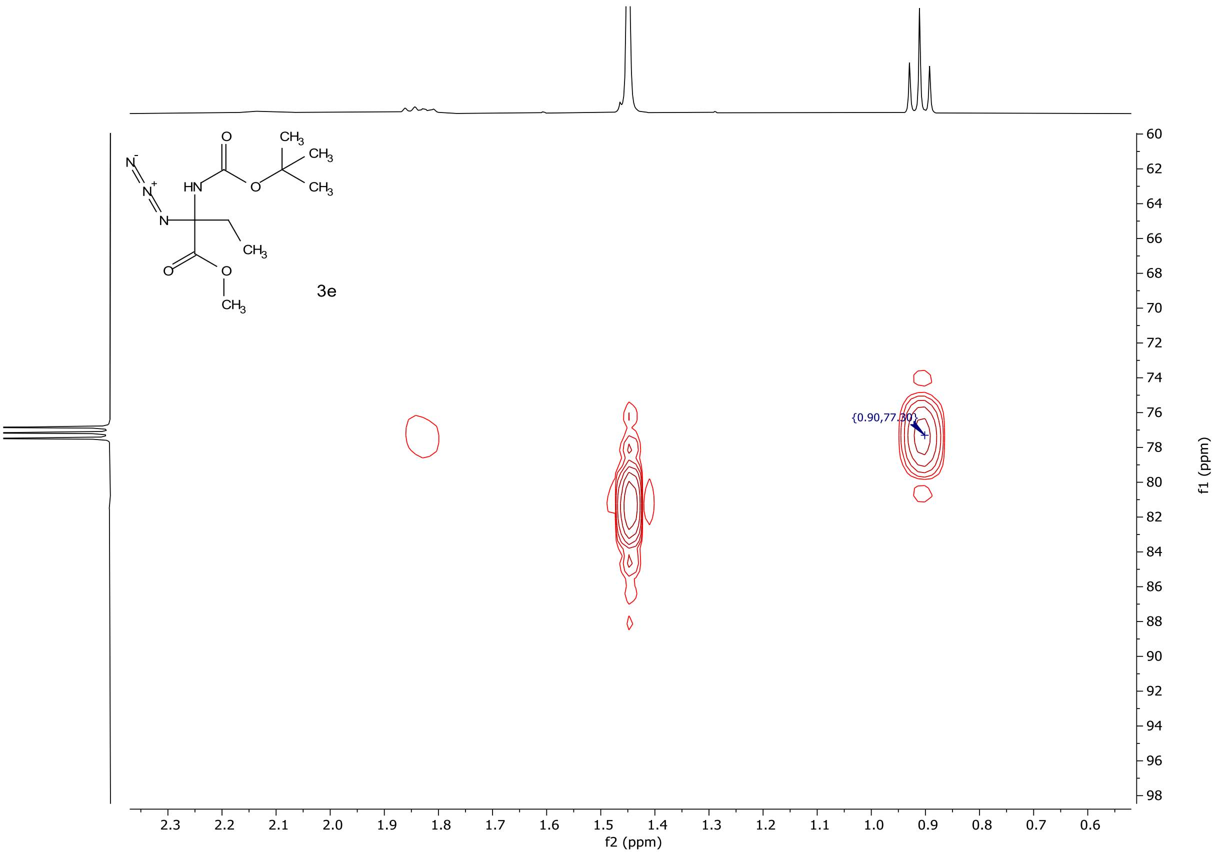

^{1}H NMR (400 MHz, $CDCl_3$) of compound **3f**

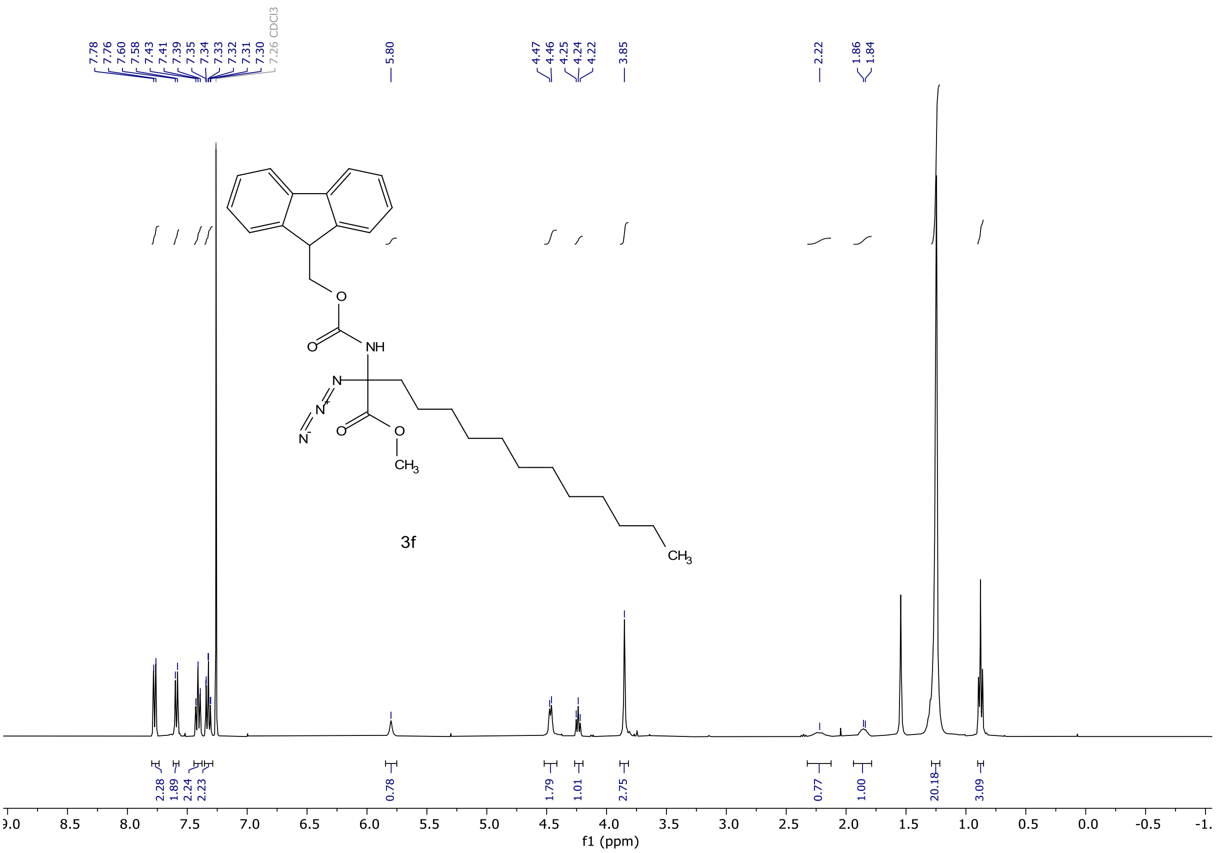

^{13}C NMR (400 MHz, $CDCl_3$) of compound **3f**

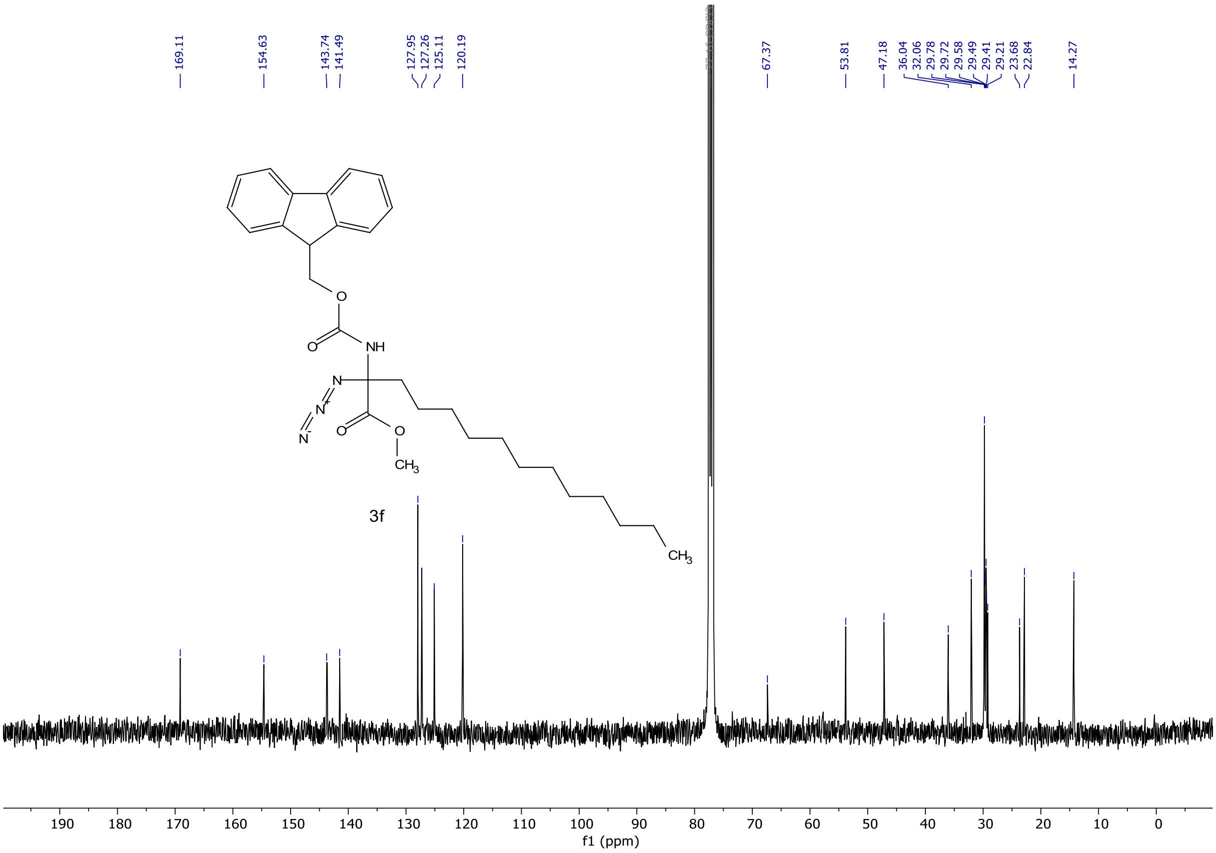

^{1}H NMR (400 MHz, $CDCl_3$) of compound **3g**

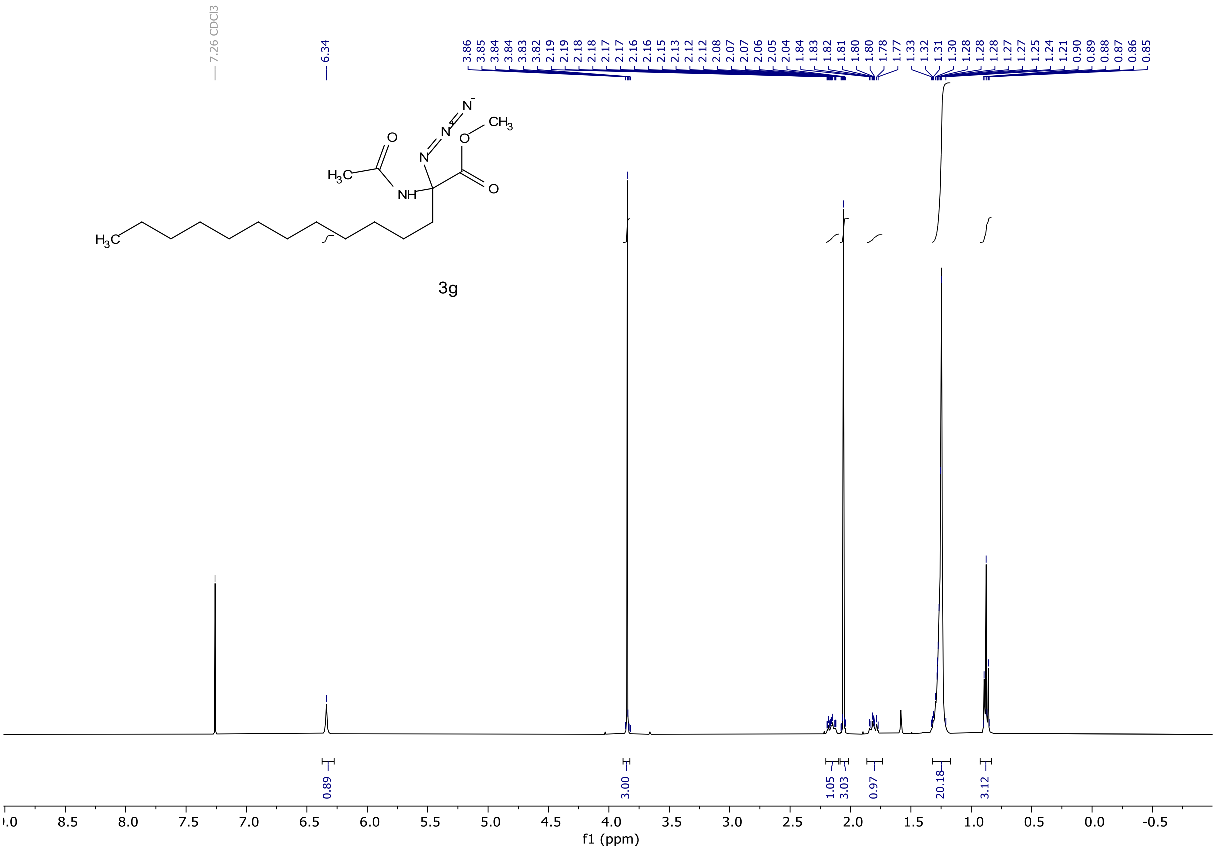

^{13}C NMR (400 MHz, $CDCl_3$) of compound **3g**

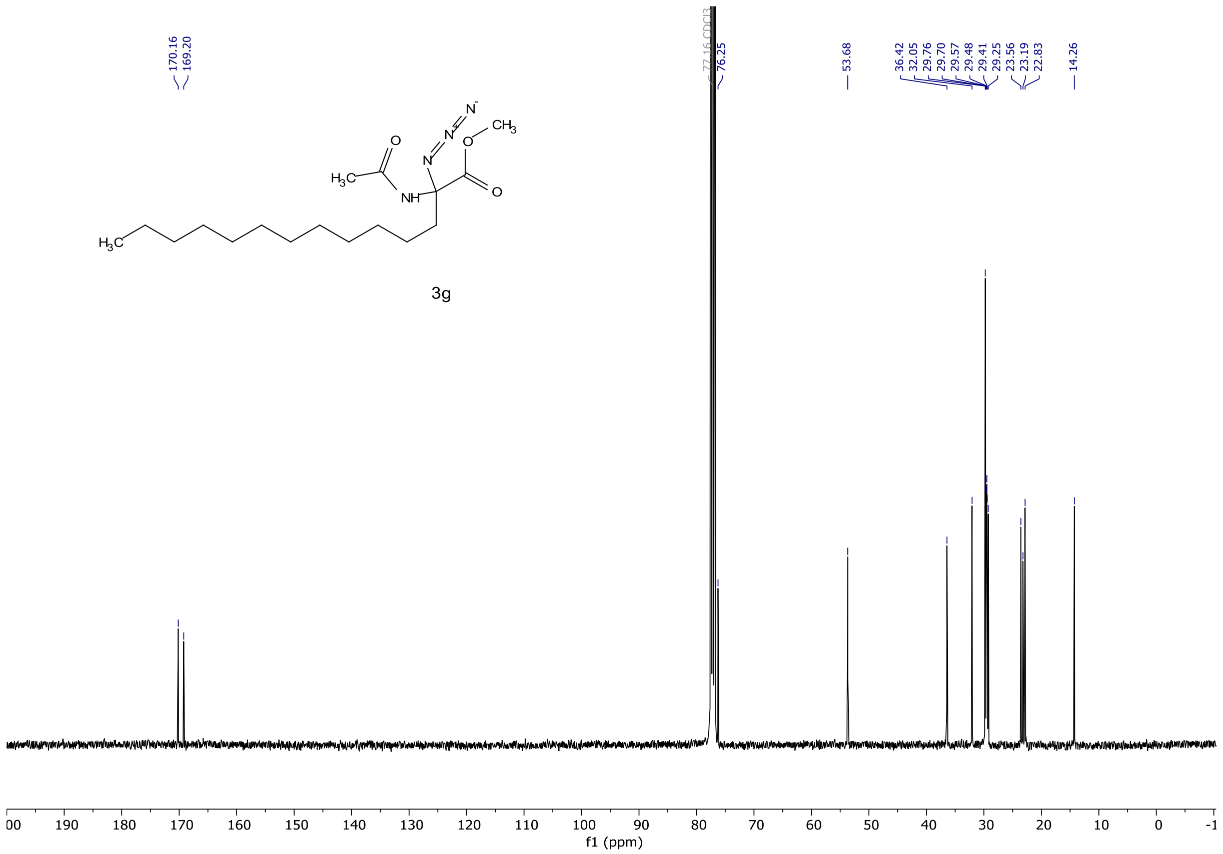

^{1}H NMR (400 MHz, $CDCl_3$) of compound **3h**

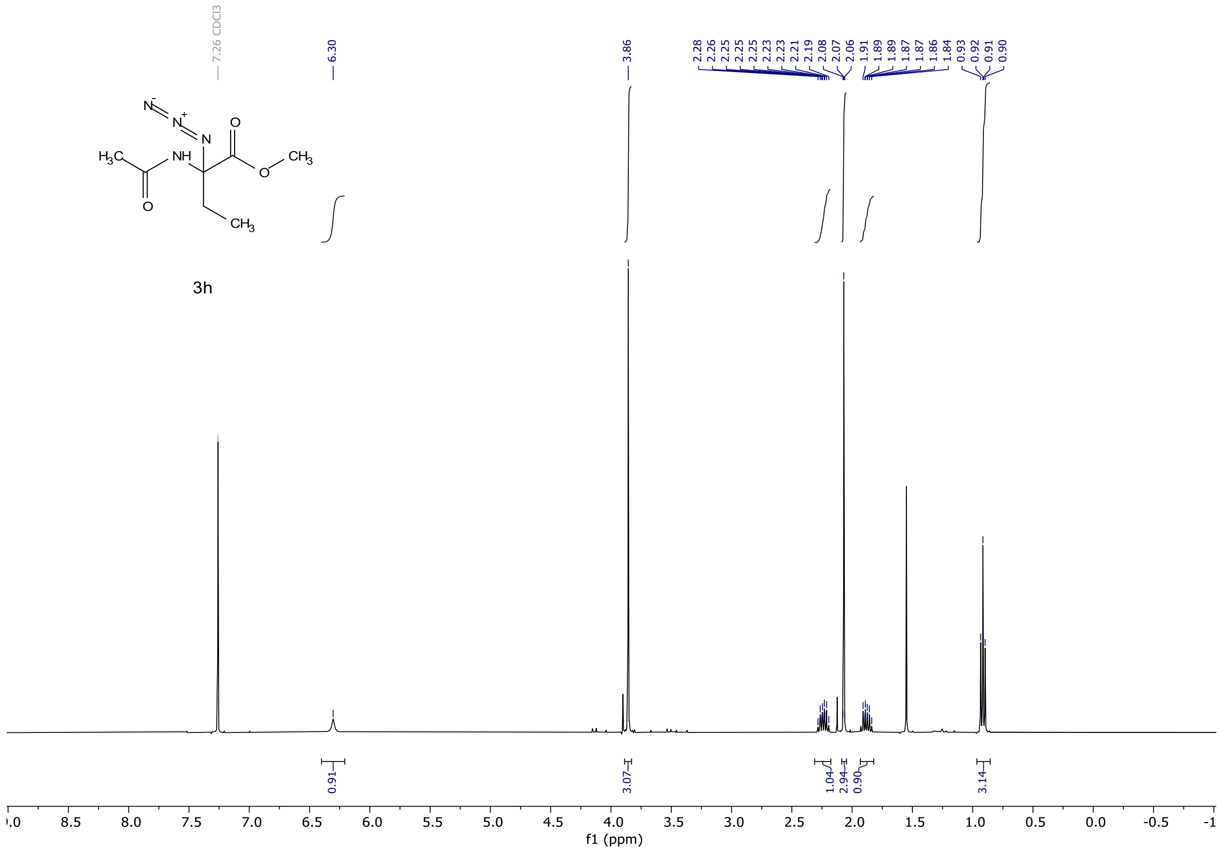

^{13}C NMR (400 MHz, $CDCl_3$) of compound **3h**

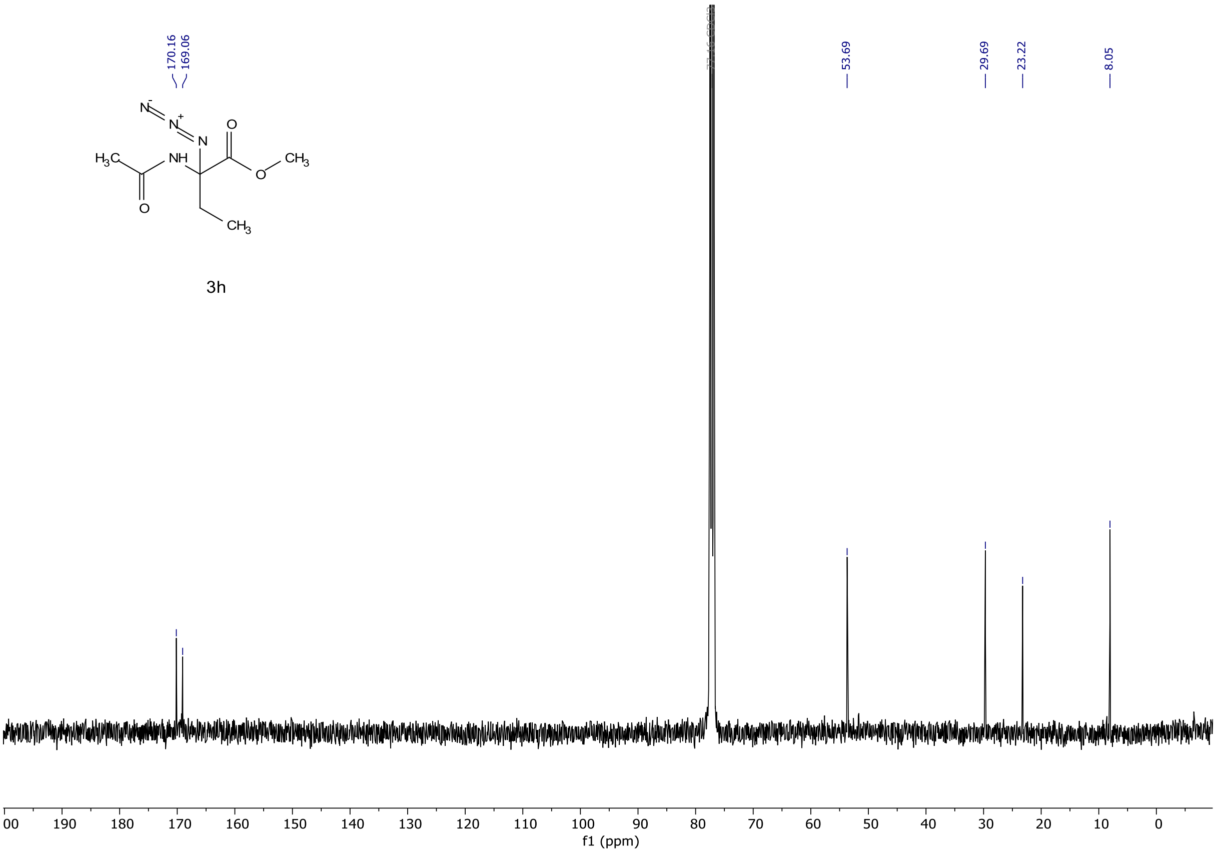

HMBC (400 MHz, $CDCl_3$) of compound **3h**

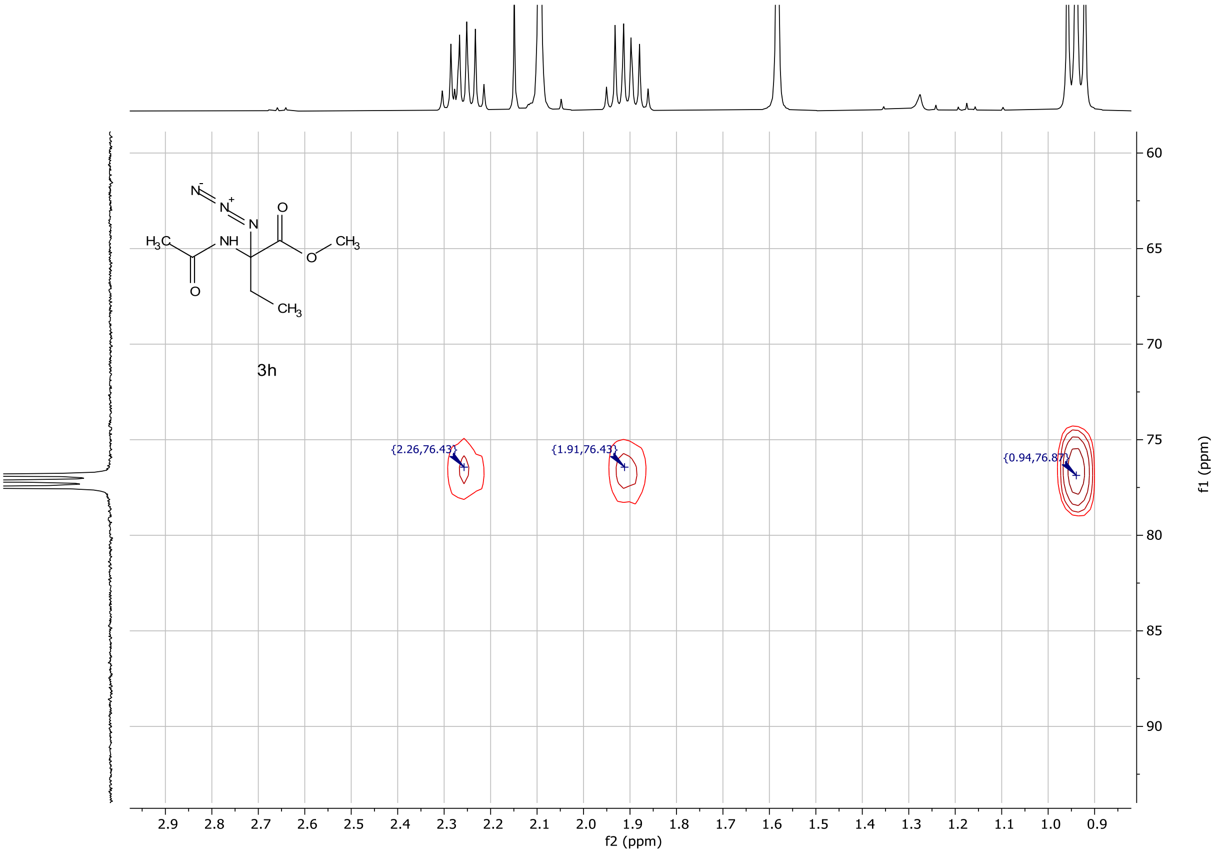

^{1}H NMR (400 MHz, $CDCl_3$) of compound **3i**

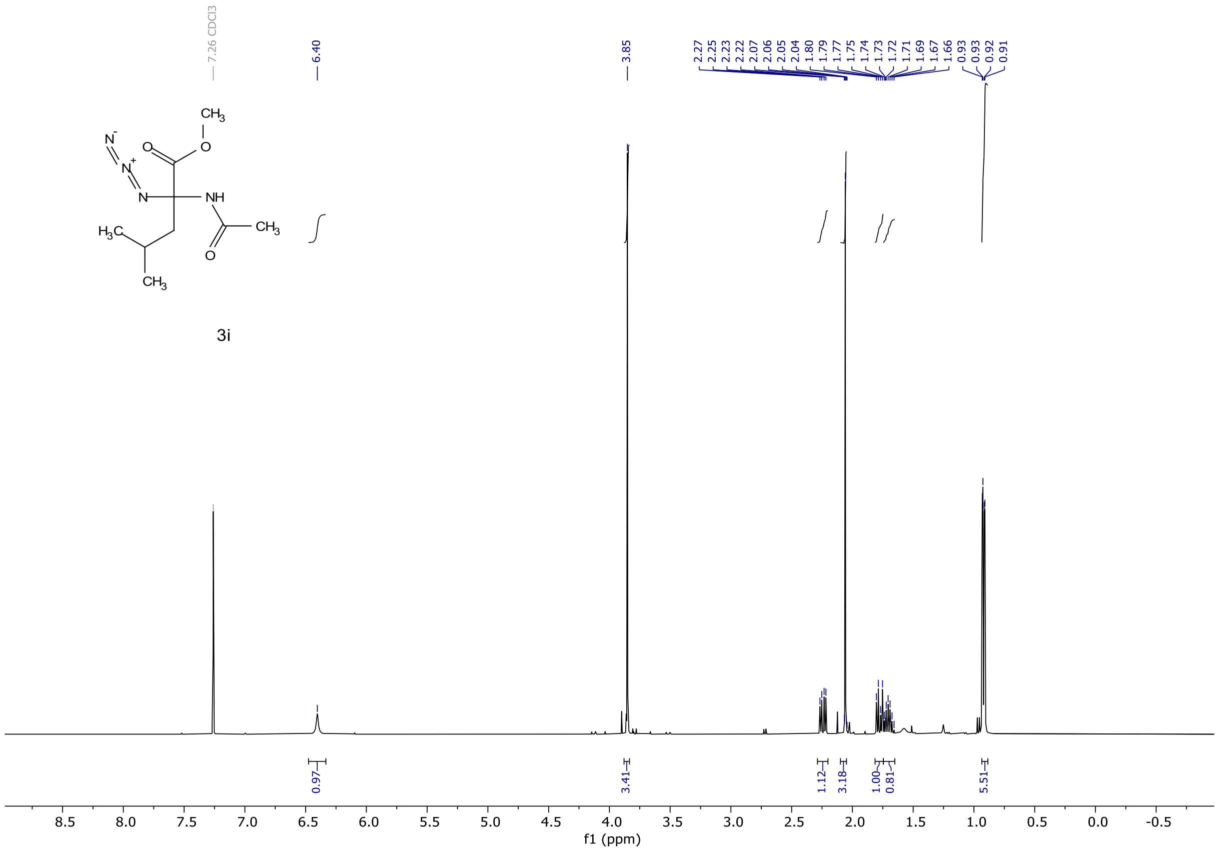

^{13}C NMR (400 MHz, $CDCl_3$) of compound **3i**

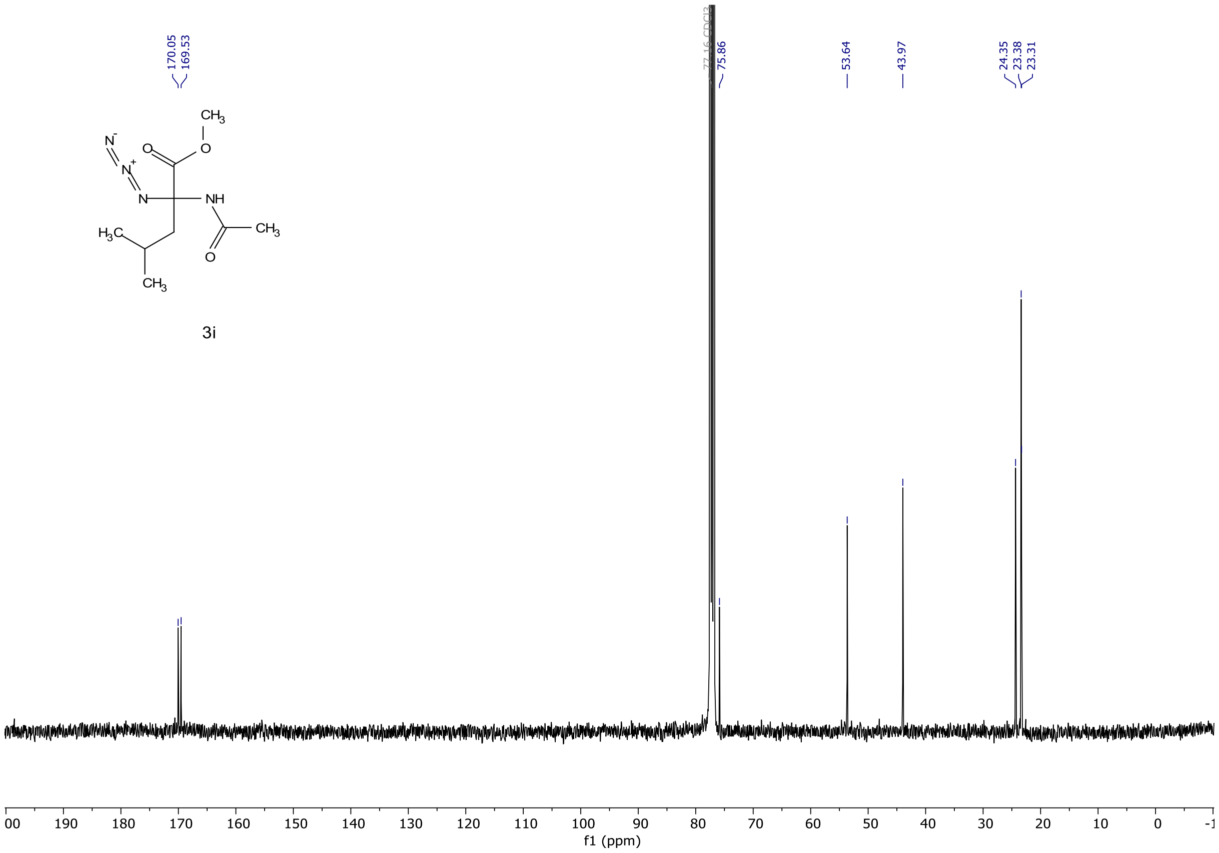

^{1}H NMR (400 MHz, $CDCl_3$) of compound **3j**

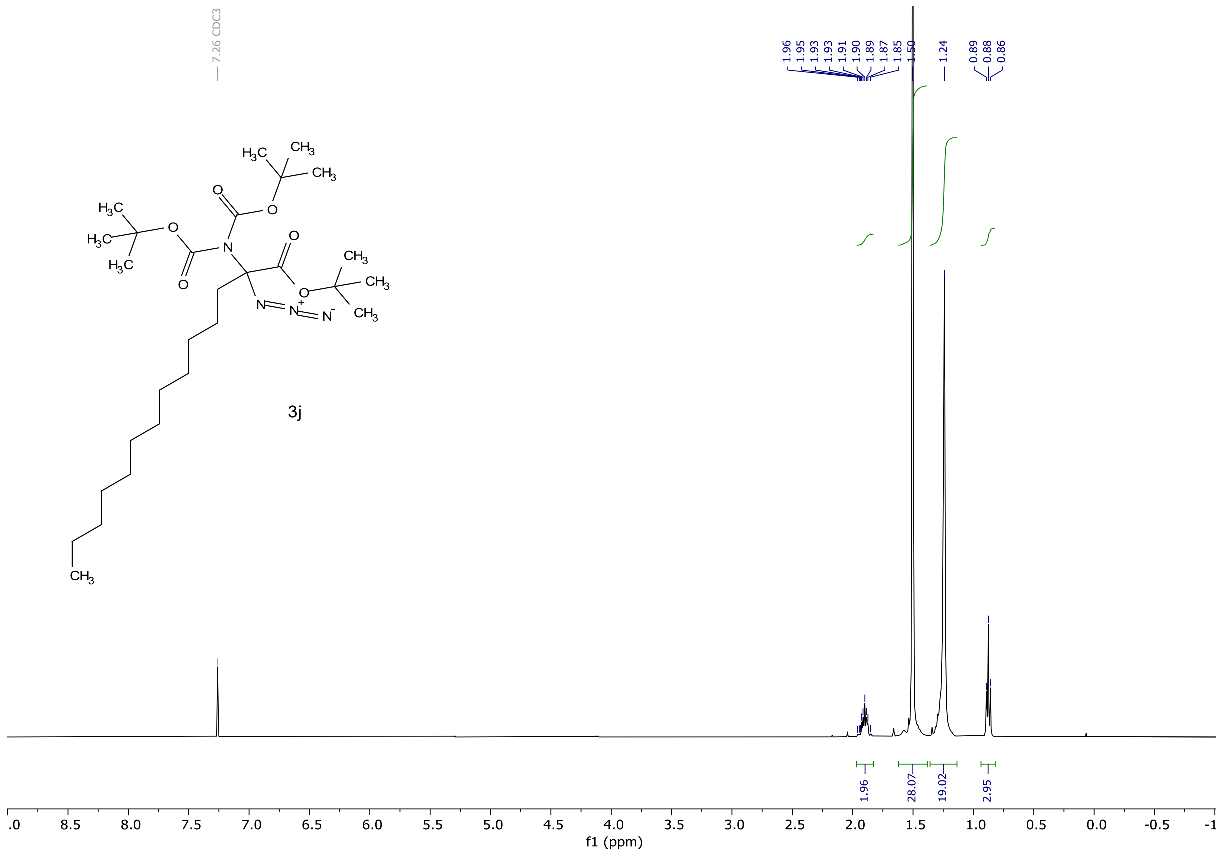

^{13}C NMR (400 MHz, $CDCl_3$) of compound **3j**

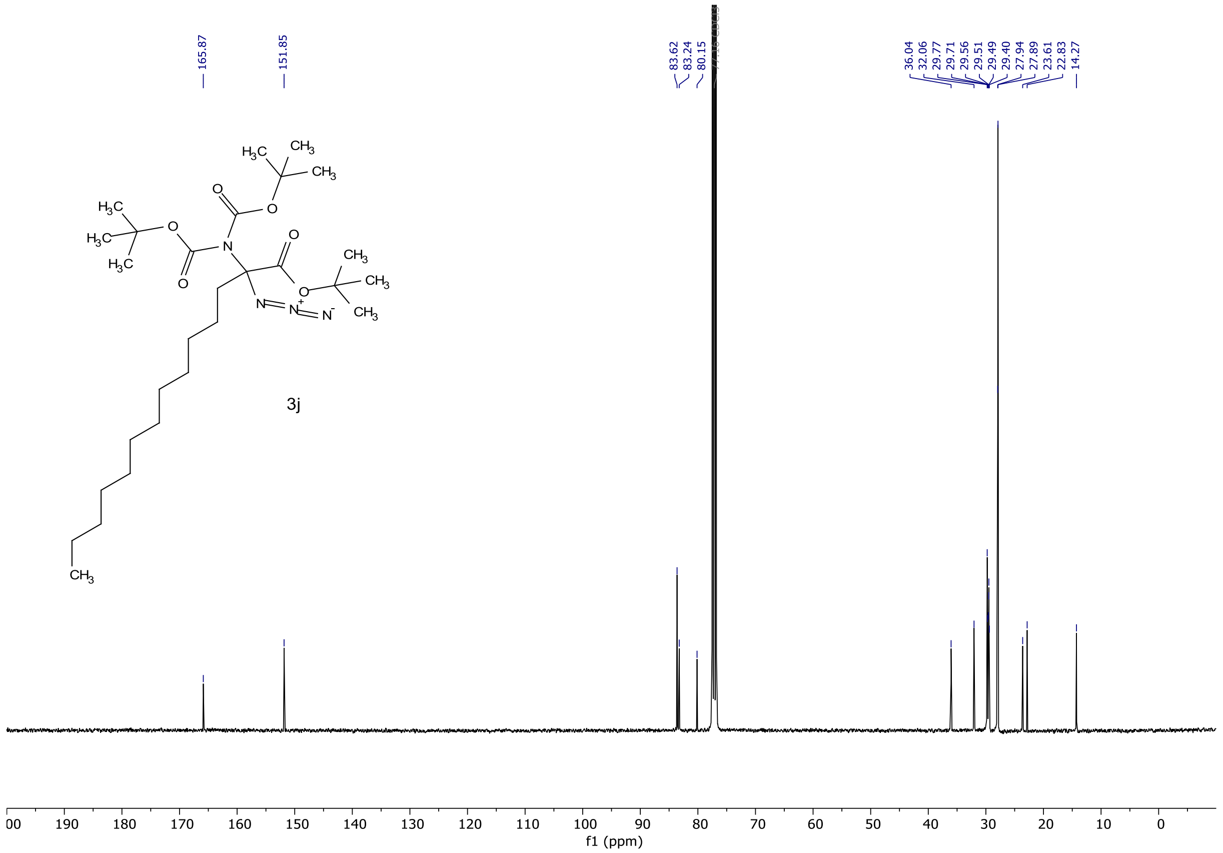

^{1}H NMR (400 MHz, $CDCl_3$) of compound **3k**

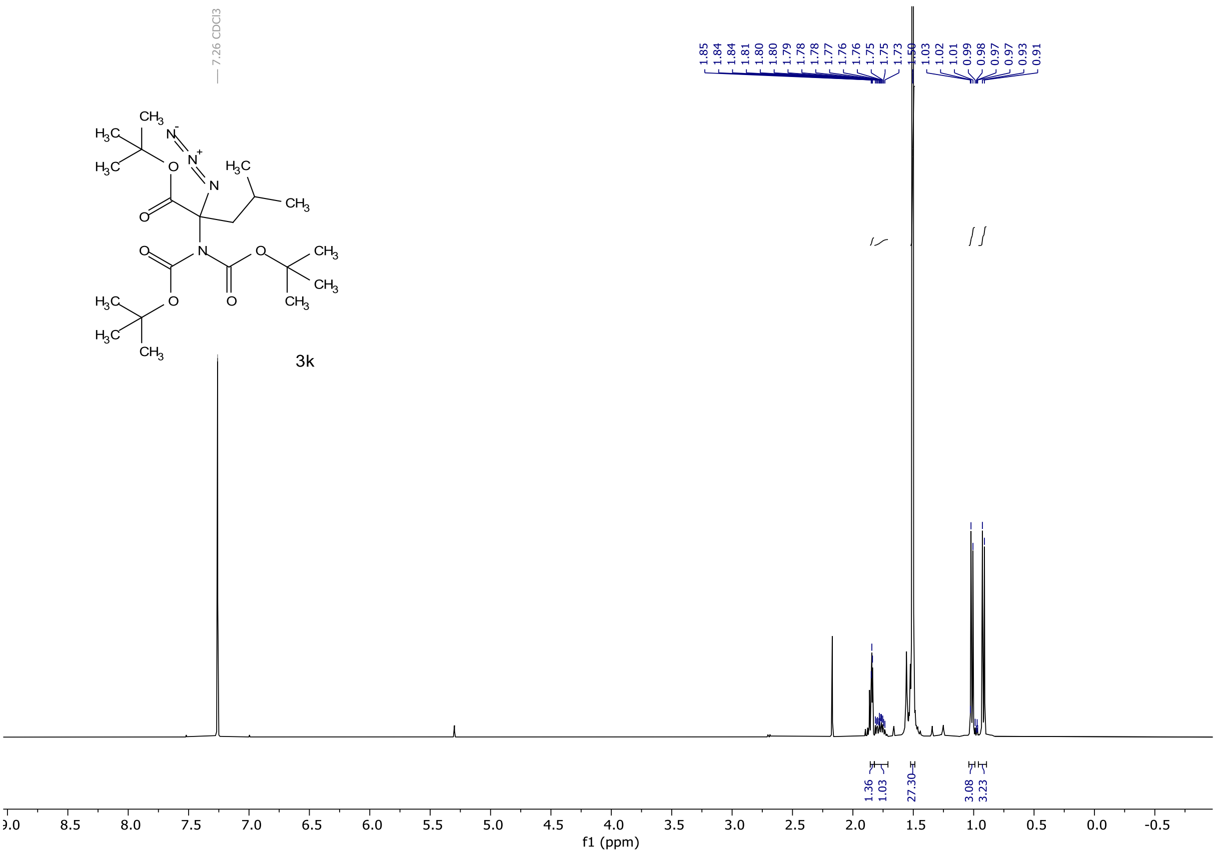

^{13}C NMR (400 MHz, $CDCl_3$) of compound **3k**

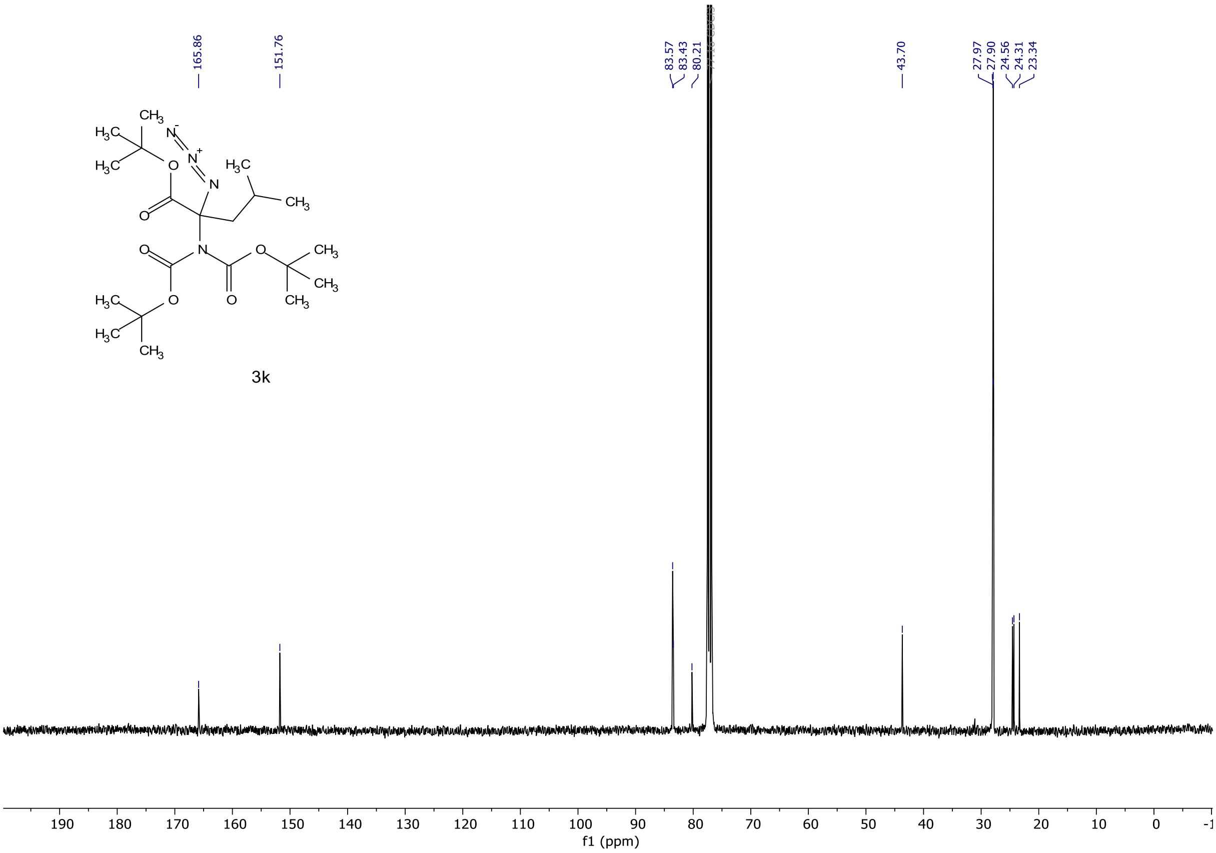

^{1}H NMR (400 MHz, $CDCl_3$) of compound **3l**

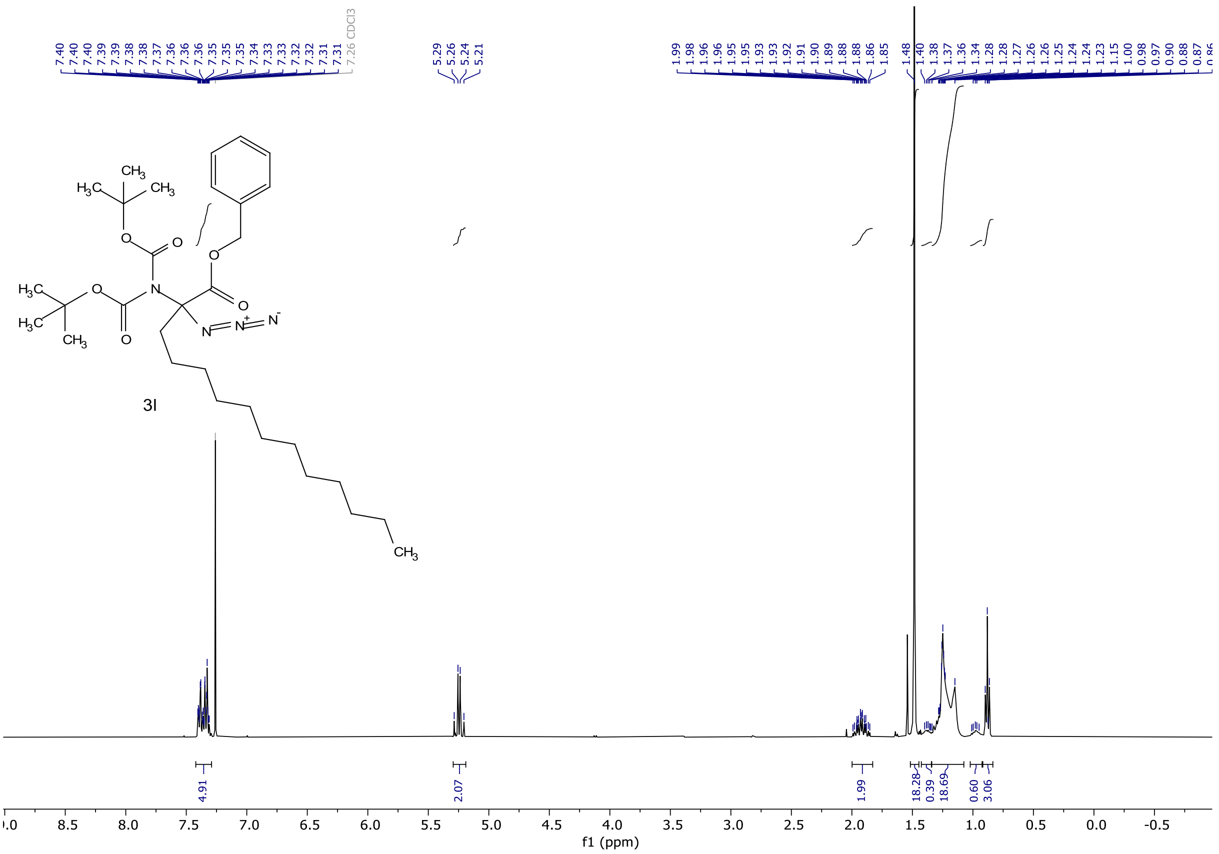

^{13}C NMR (400 MHz, $CDCl_3$) of compound **3l**

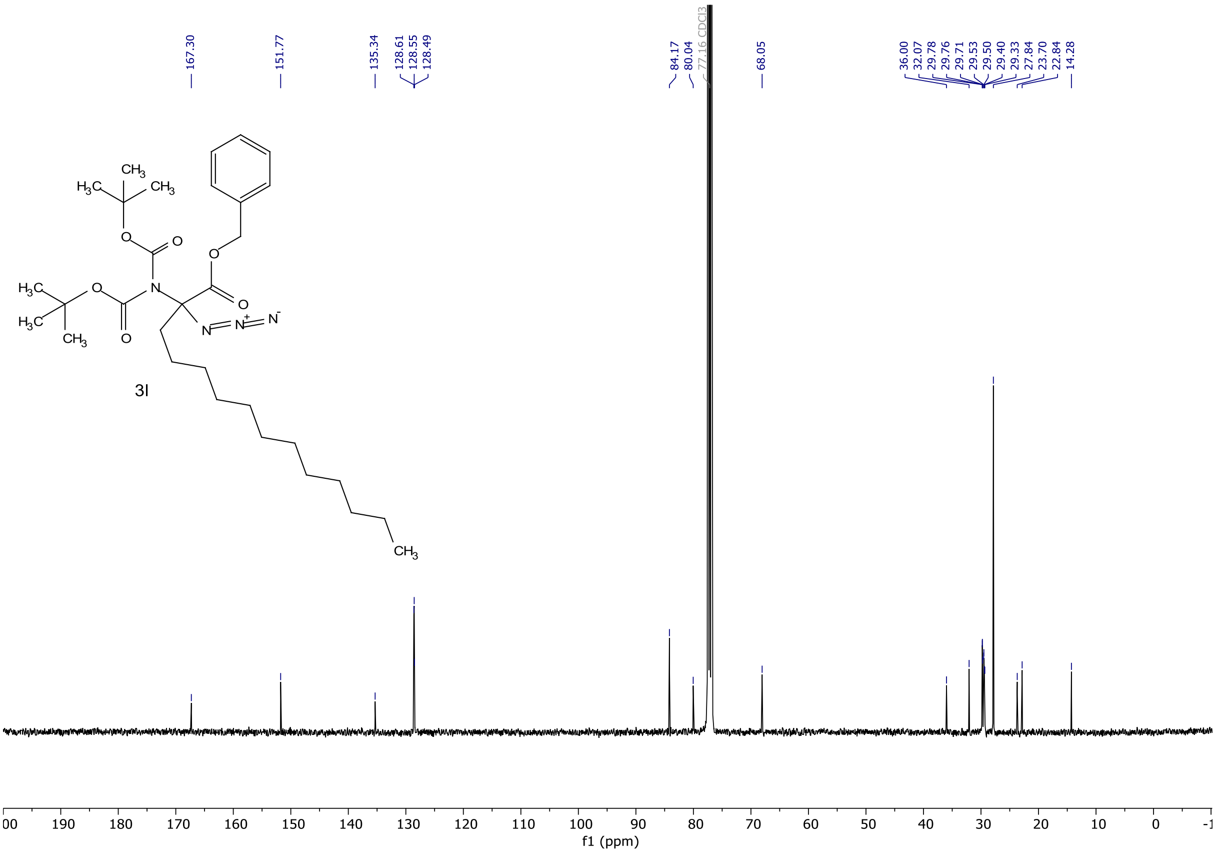

^{1}H NMR (400 MHz, $CDCl_3$) of compound **3m**

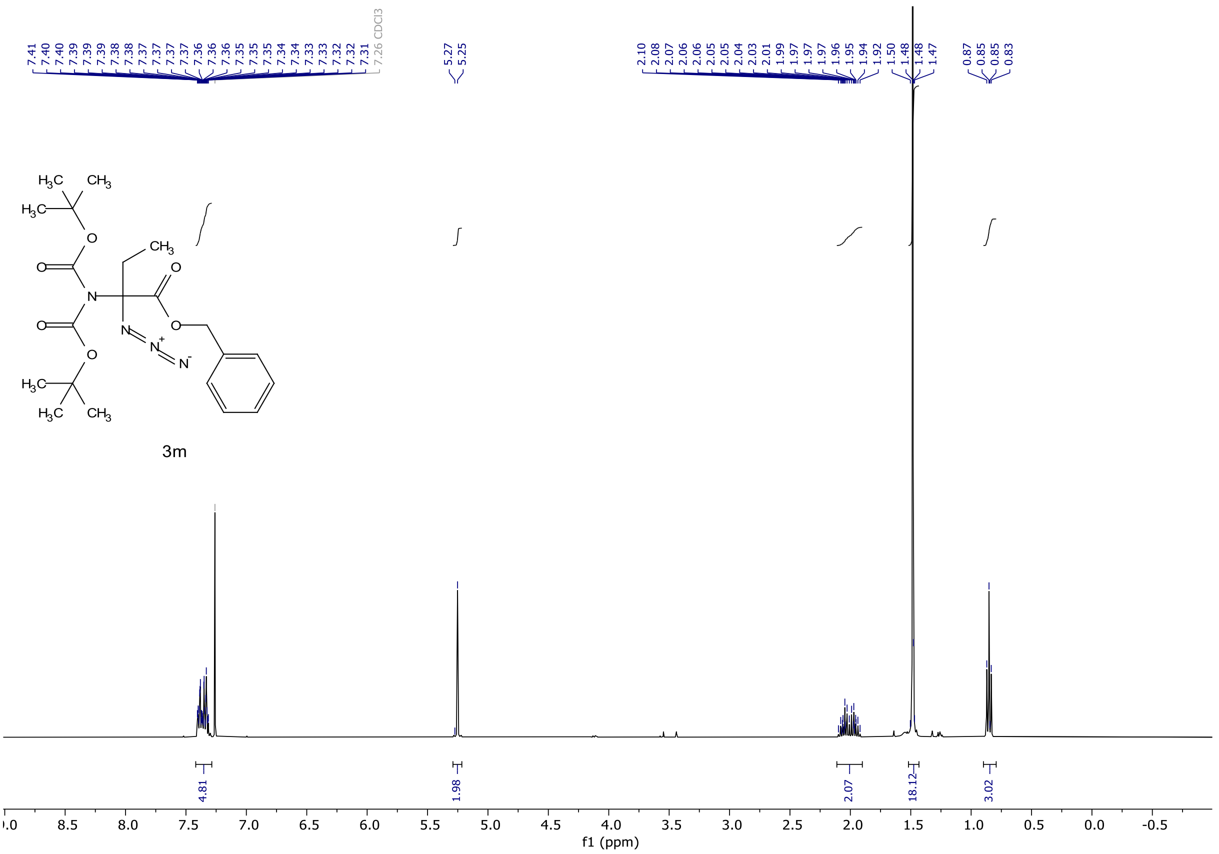

^{13}C NMR (400 MHz, $CDCl_3$) of compound **3m**

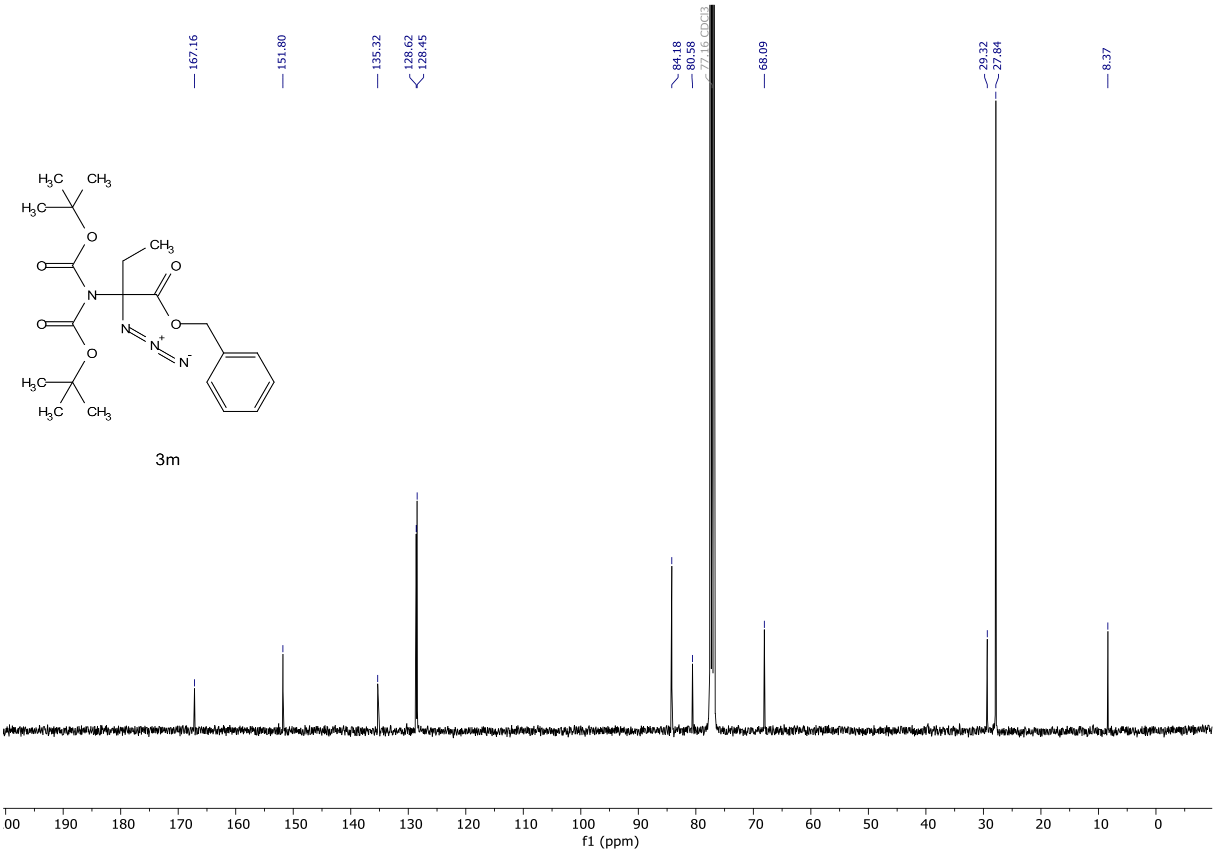

^{1}H NMR (400 MHz, $CDCl_3$) of compound **3n**

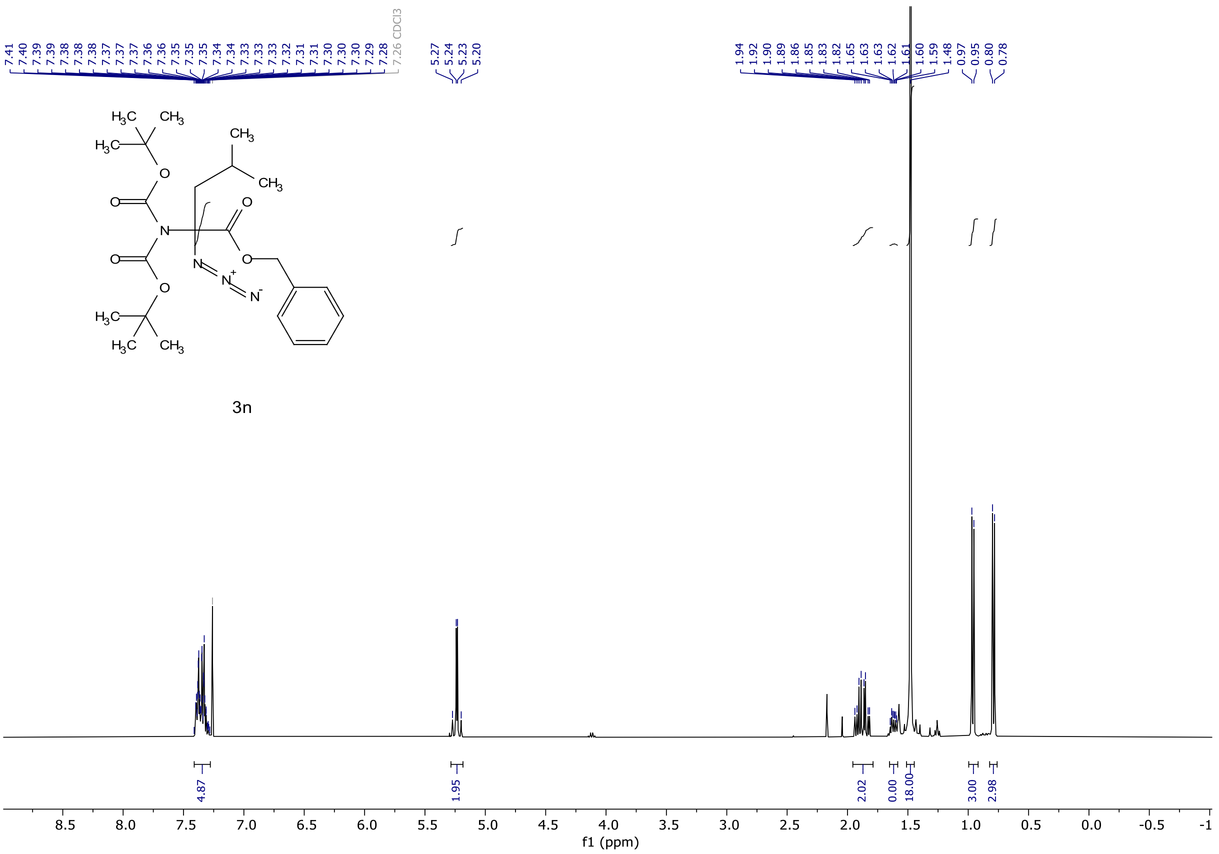

^{13}C NMR (400 MHz, $CDCl_3$) of compound **3n**

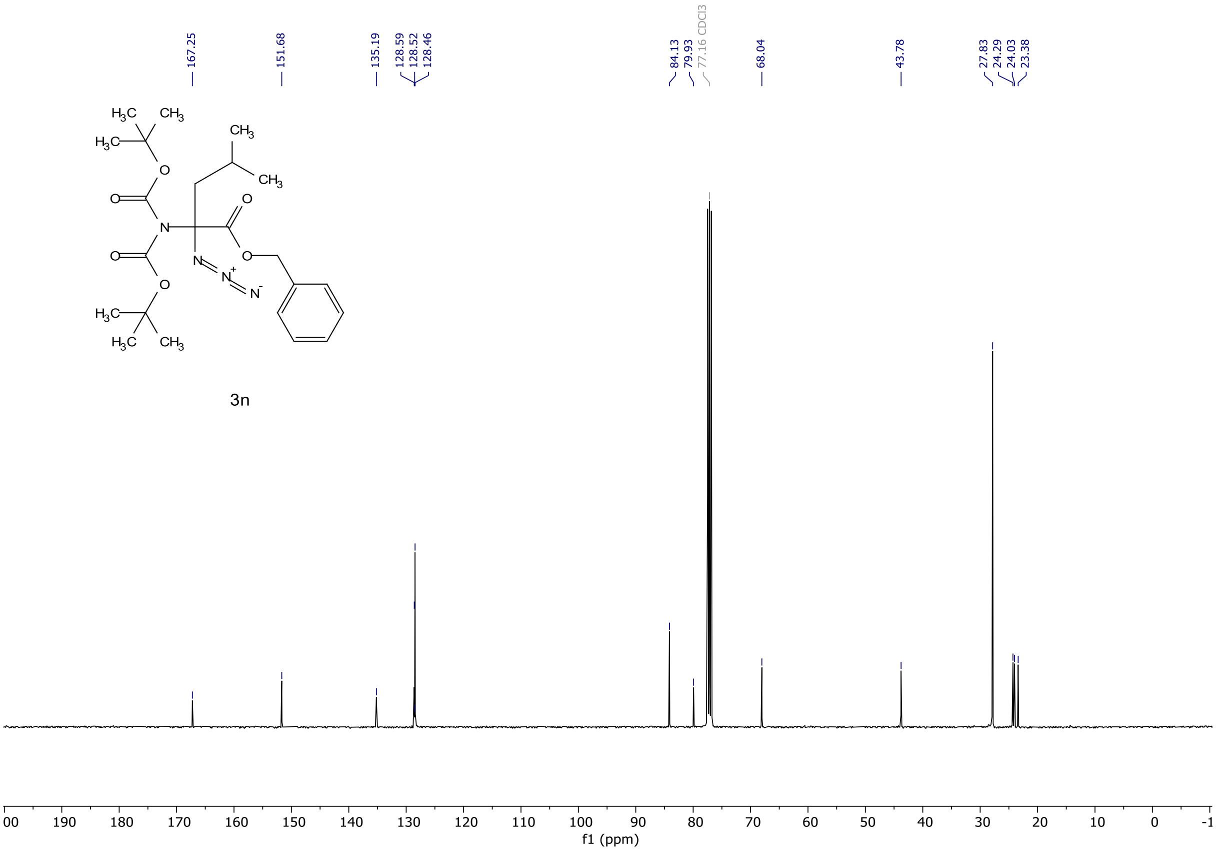

^{1}H NMR (400 MHz, $CDCl_3$) of compound **3o**

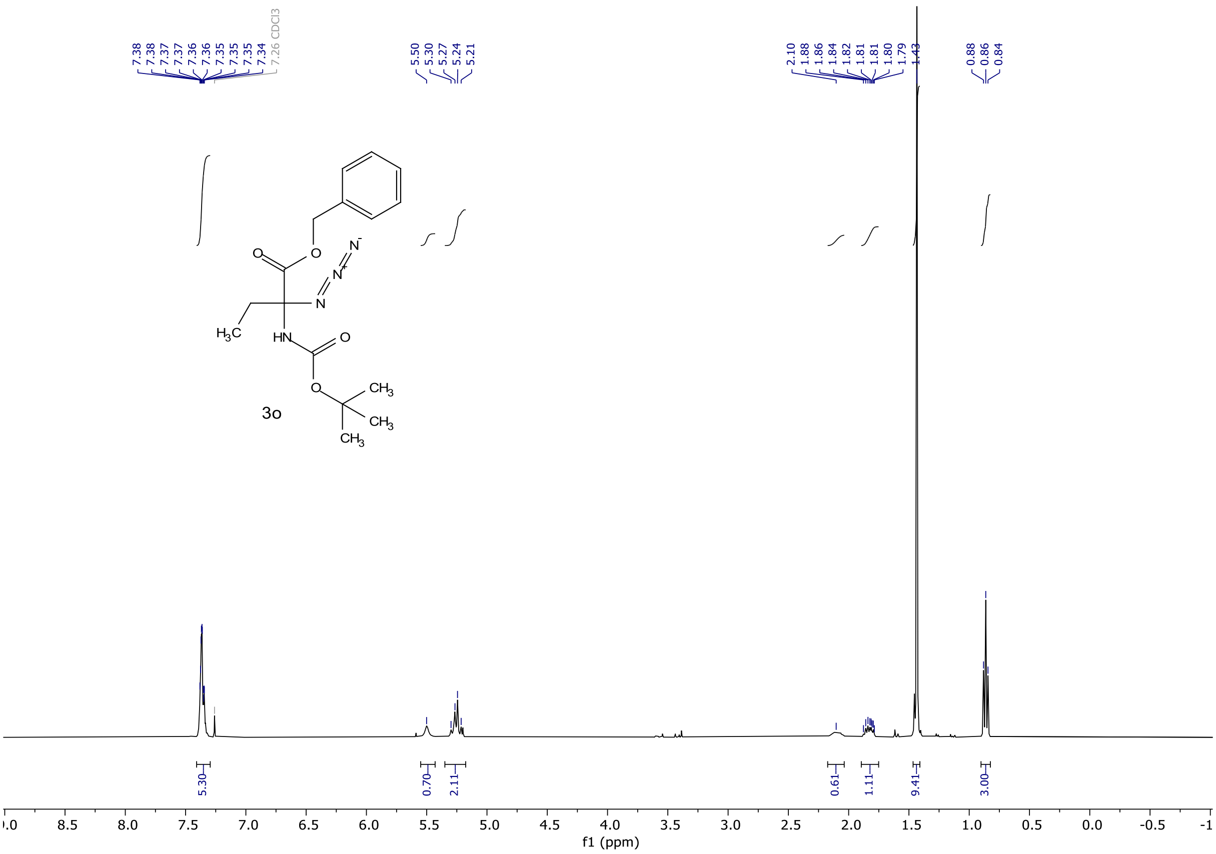

^{13}C NMR (400 MHz, $CDCl_3$) of compound **3o**

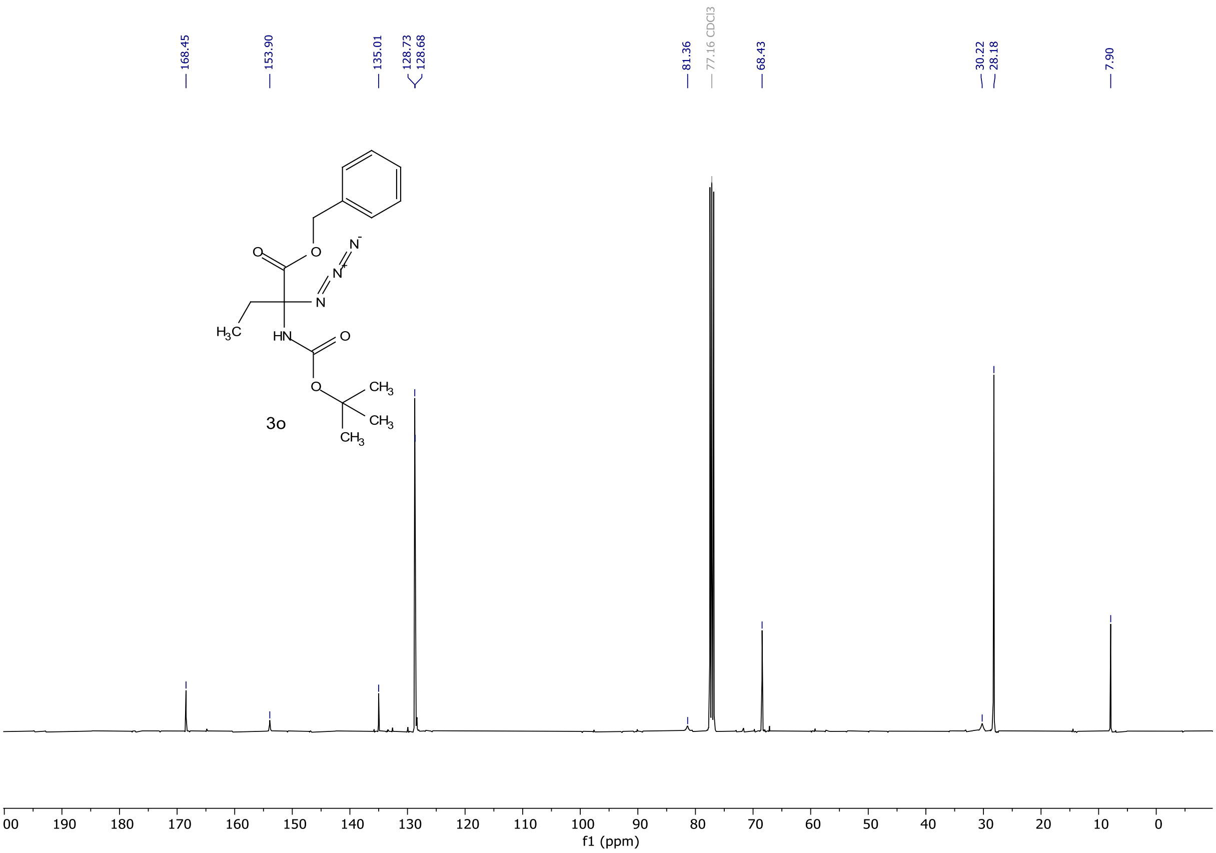

HMBC (400 MHz, CDCl$_3$) of compound **3o**

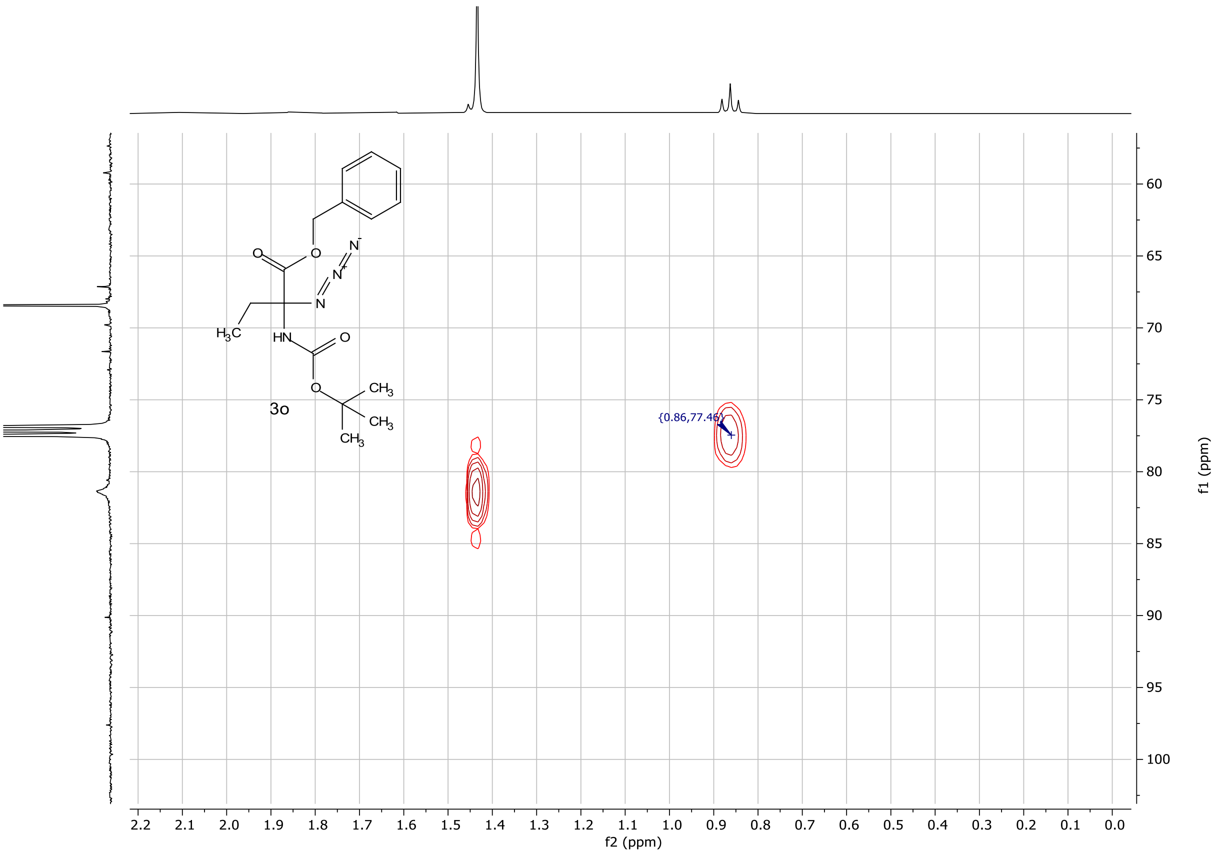

^{1}H NMR (400 MHz, CDCl$_3$) of compound **3p**

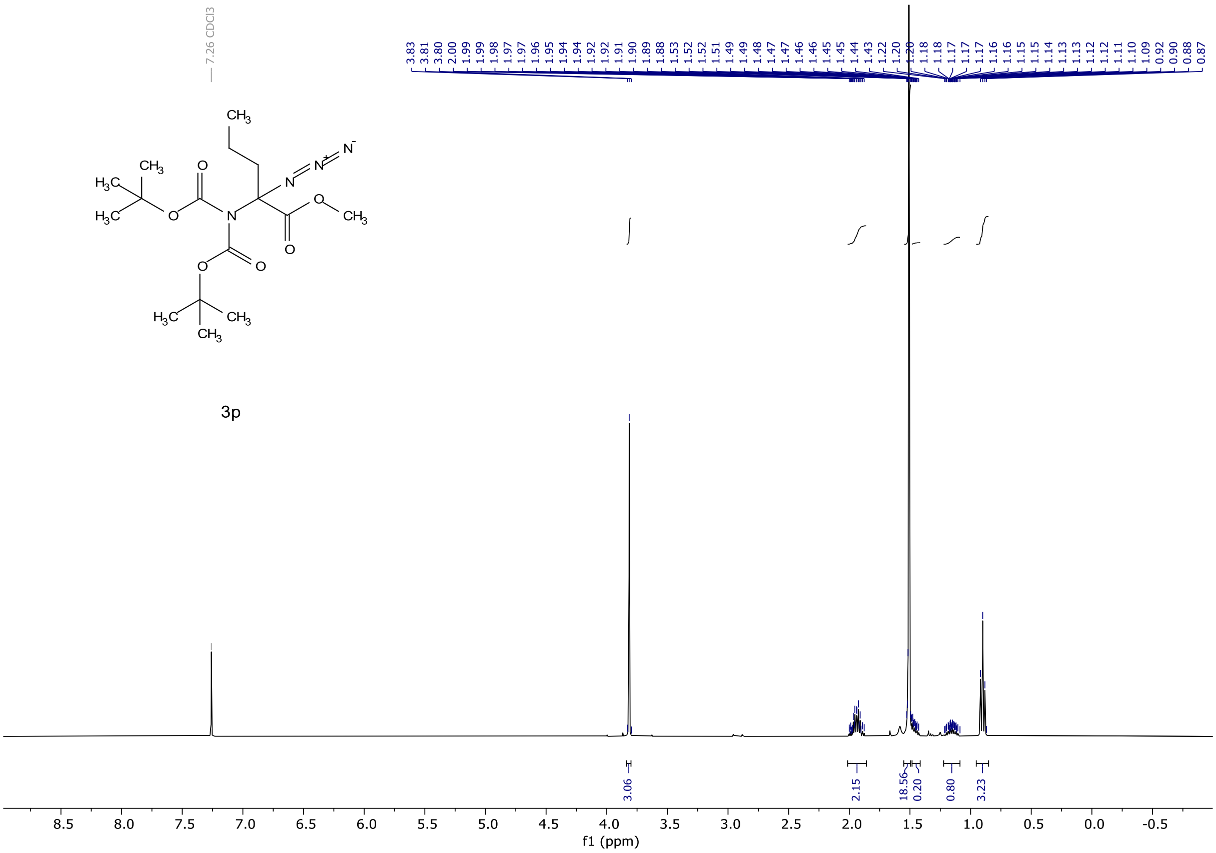

^{13}C NMR (400 MHz, $CDCl_3$) of compound **3p**

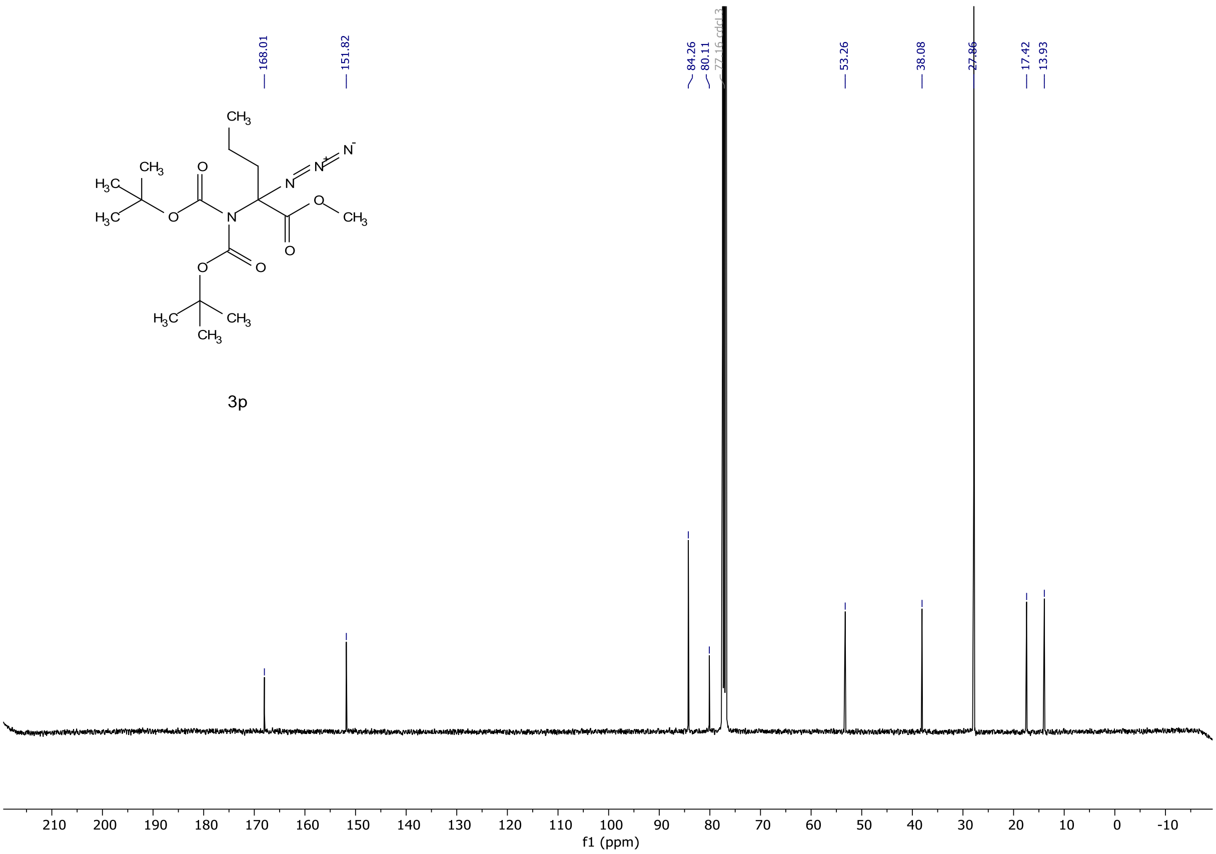

^{1}H NMR (400 MHz, $CDCl_3$) of compound **3q**

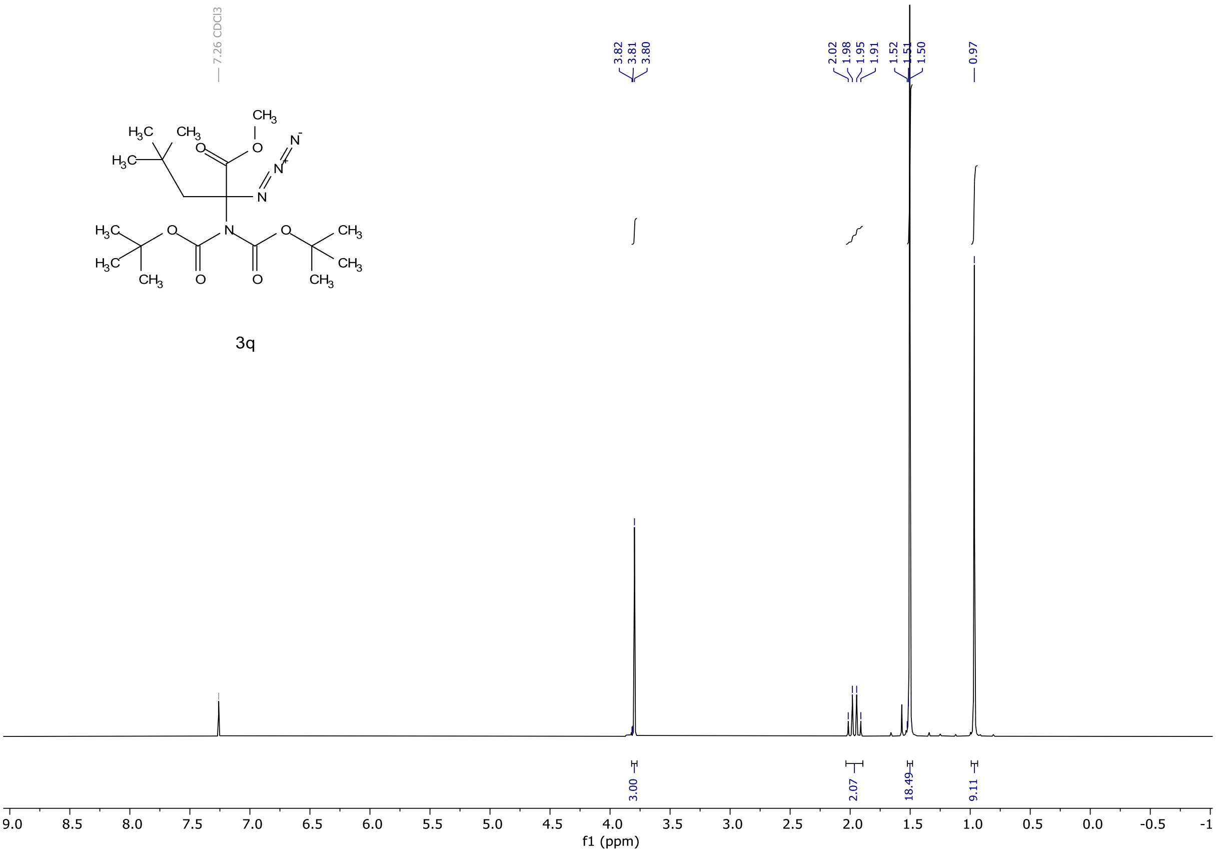

^{13}C NMR (400 MHz, $CDCl_3$) of compound **3q**

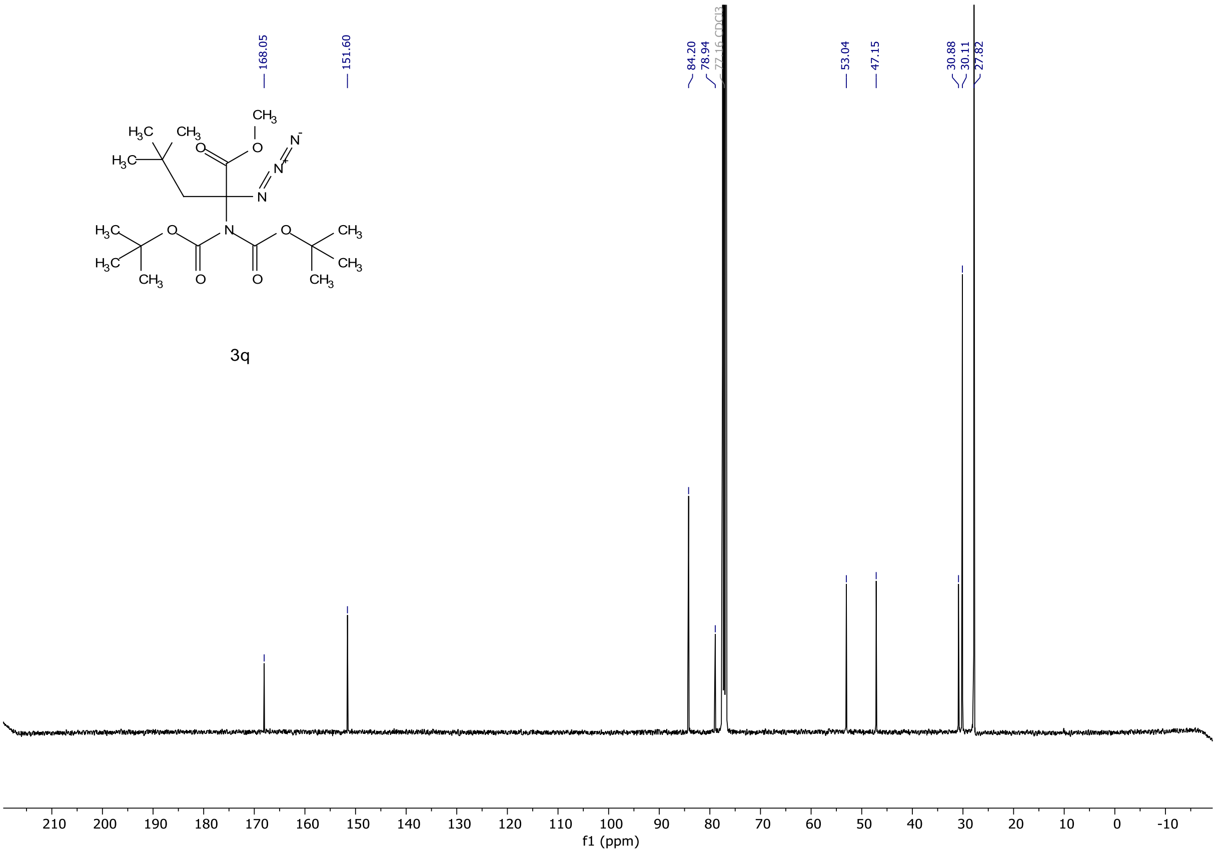

^{1}H NMR (400 MHz, $CDCl_3$) of compound **3r**

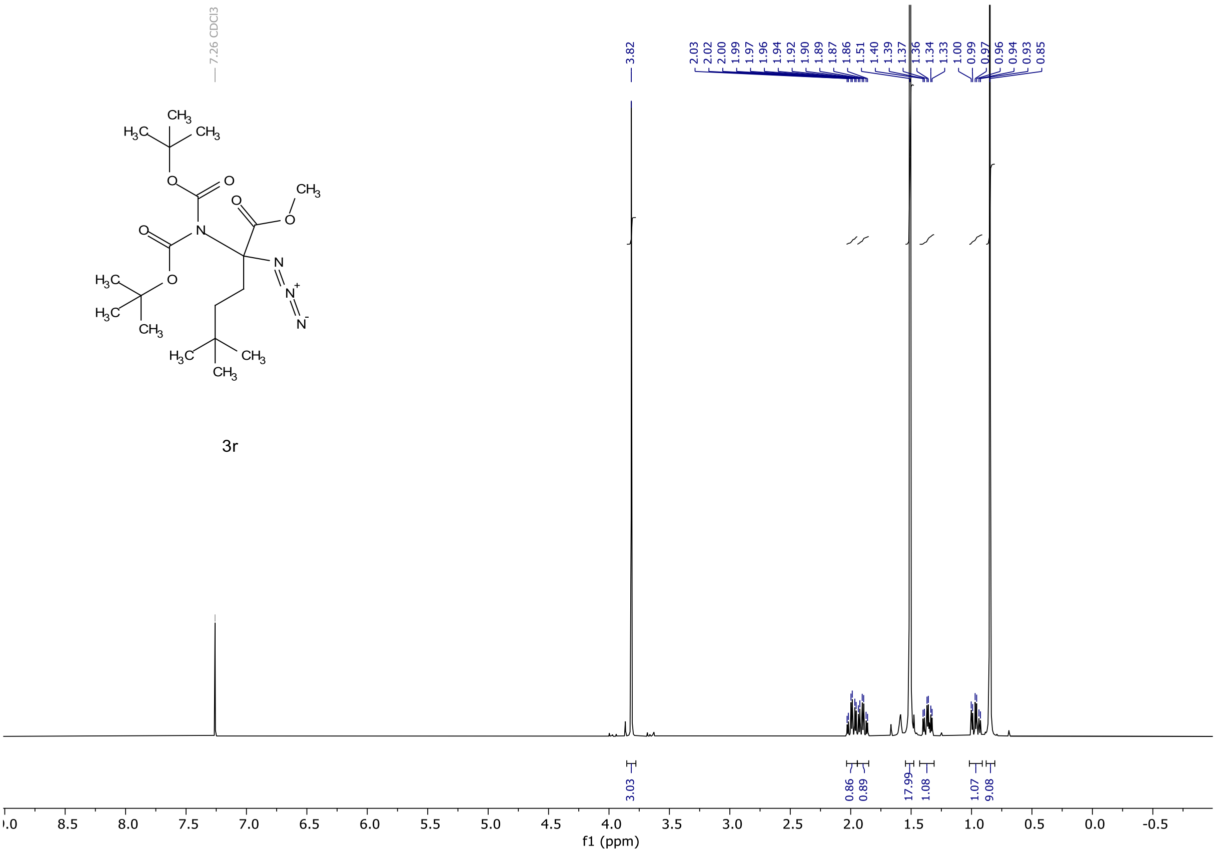

^{13}C NMR (400 MHz, $CDCl_3$) of compound **3r**

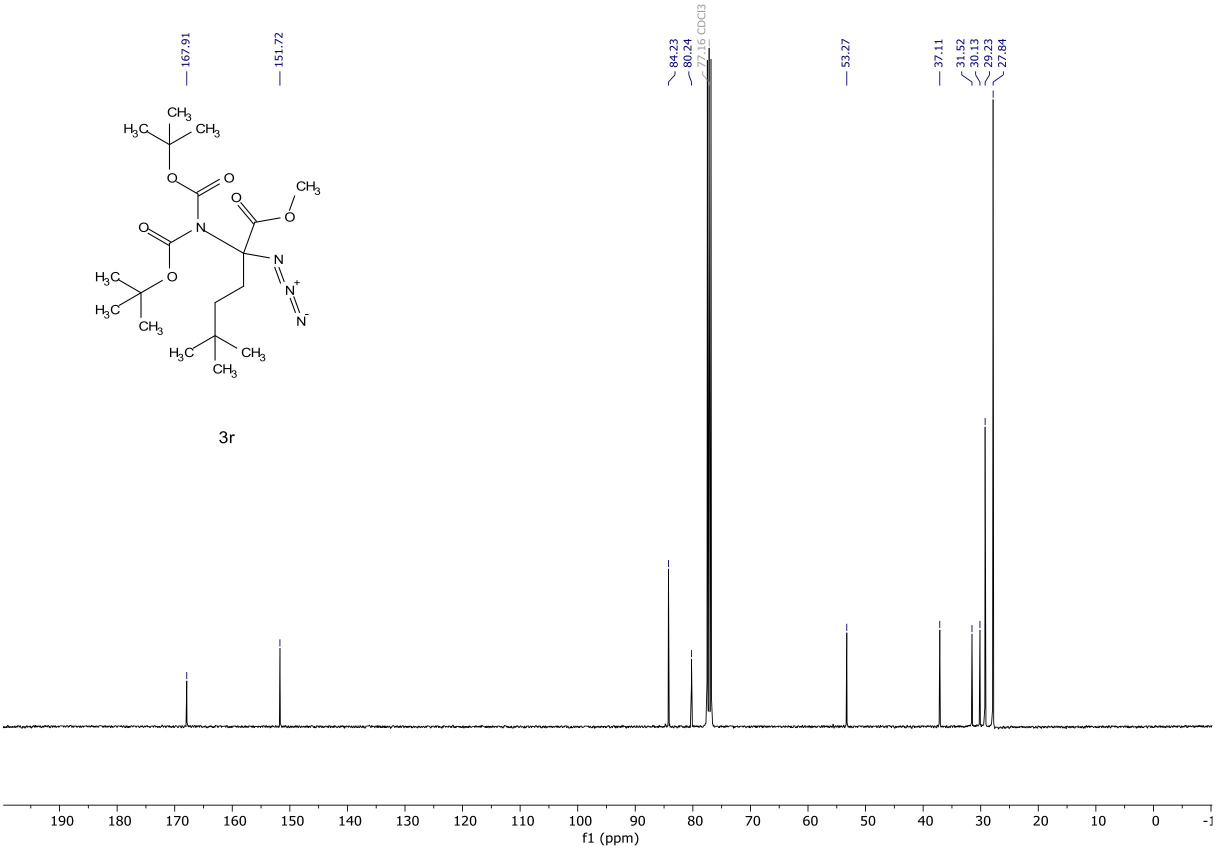

^{1}H NMR (400 MHz, $CDCl_3$) of compound **3s**

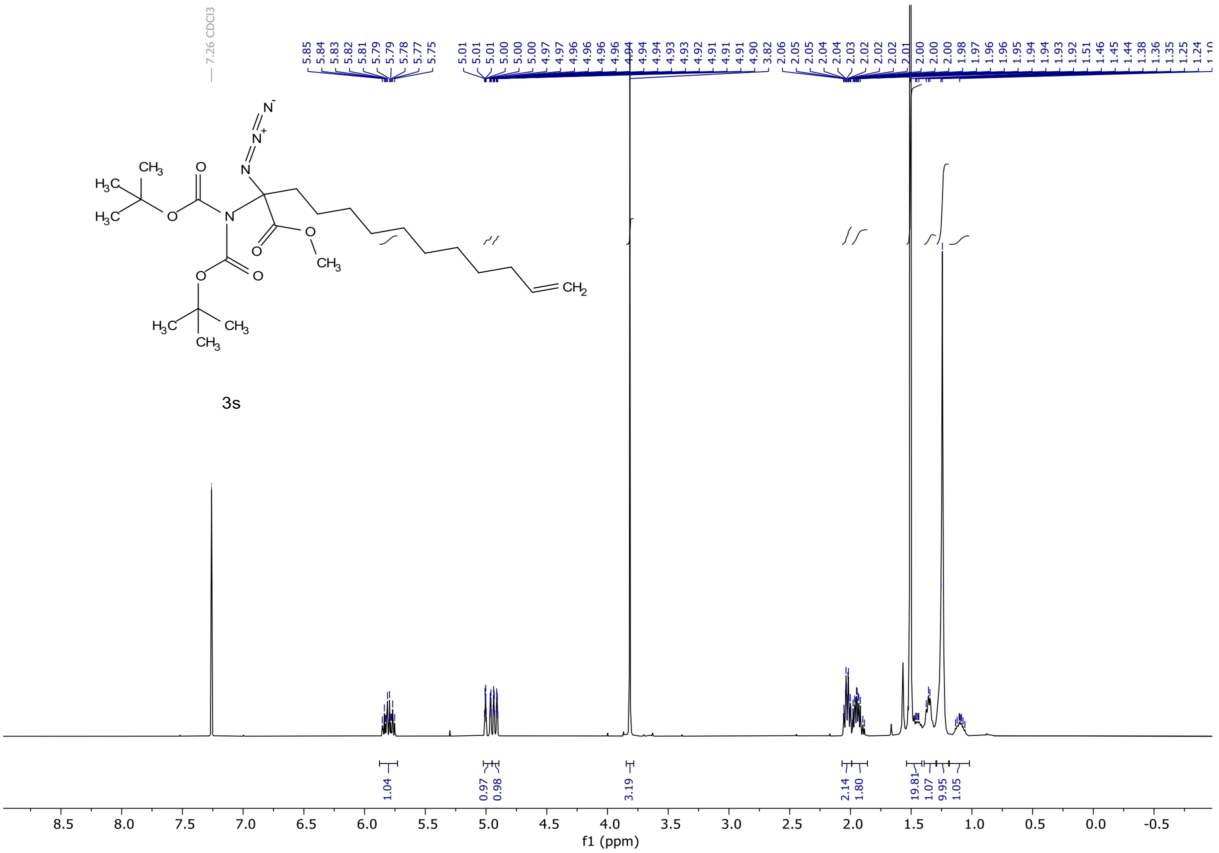

^{13}C NMR (400 MHz, $CDCl_3$) of compound **3s**

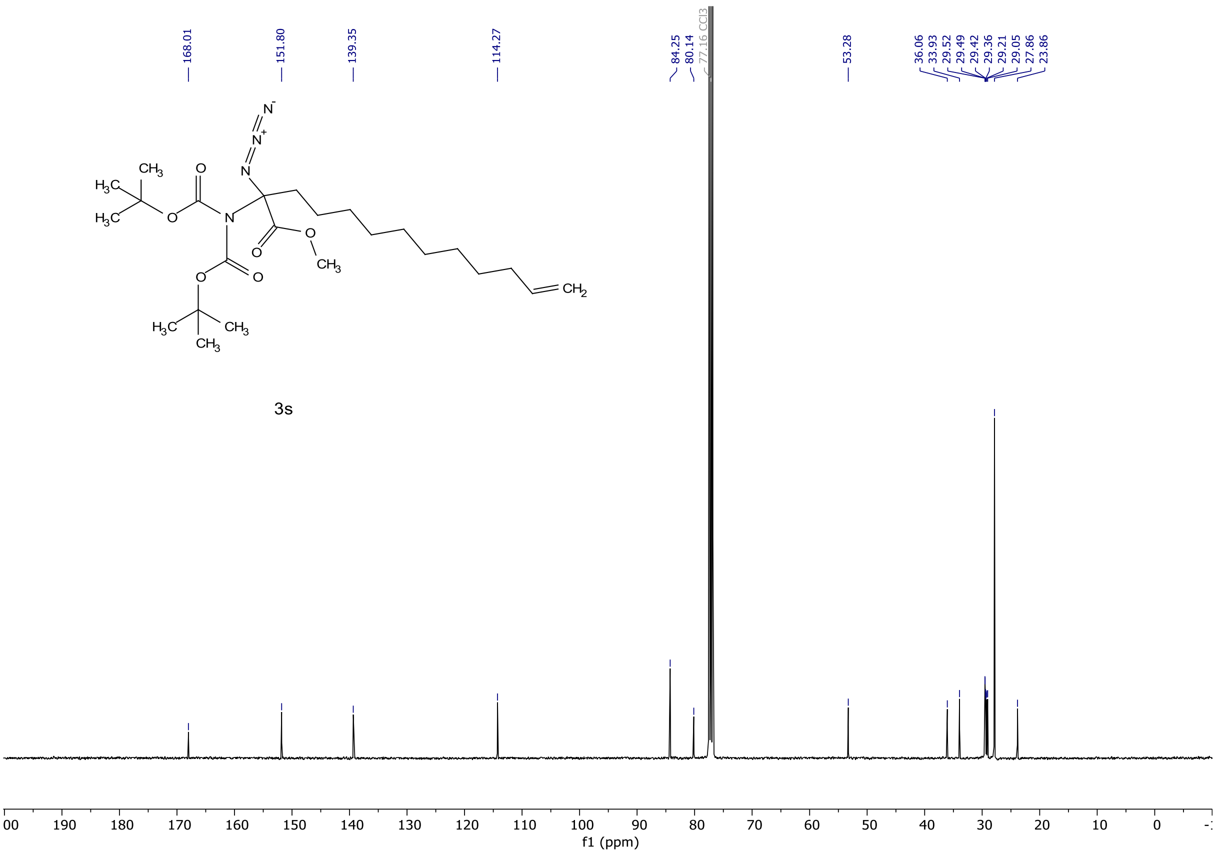

^{1}H NMR (400 MHz, $CDCl_3$) of compound **3t**

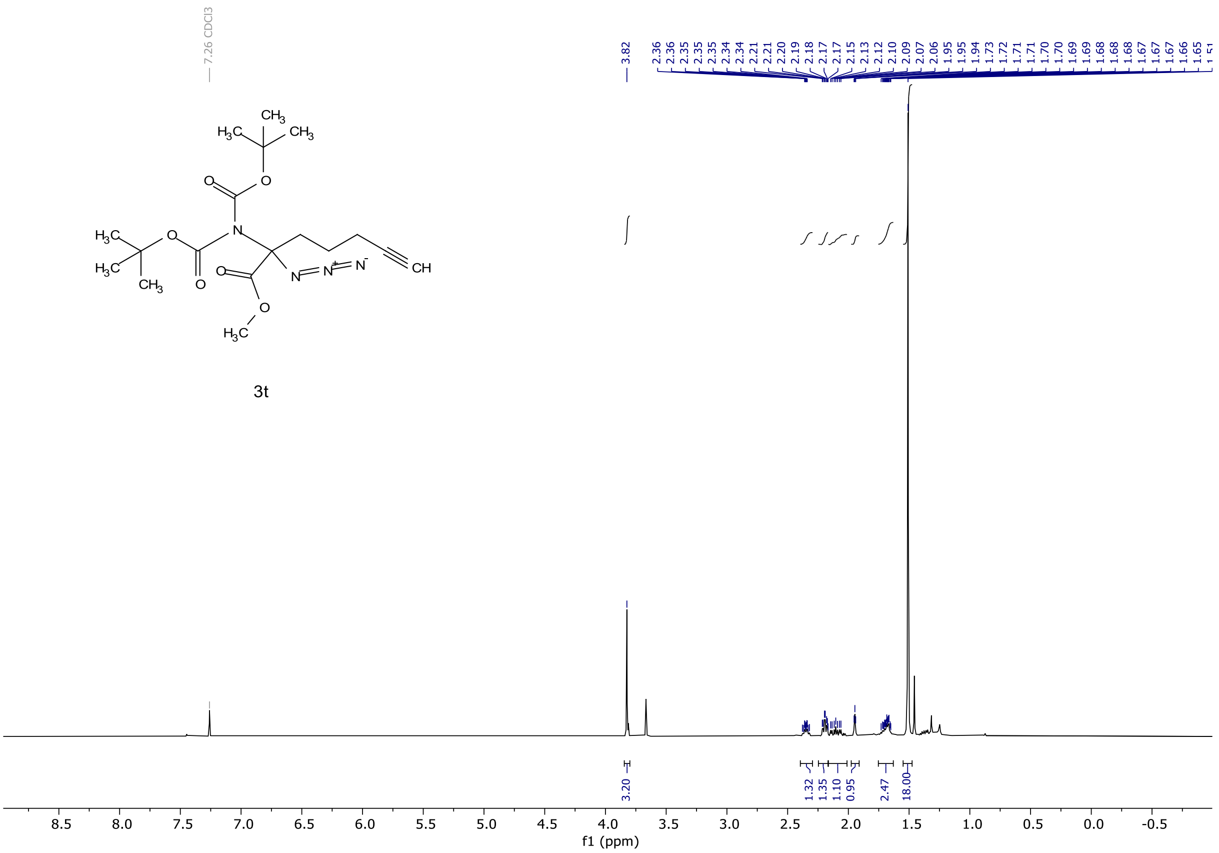

^{13}C NMR (400 MHz, $CDCl_3$) of compound **3t**

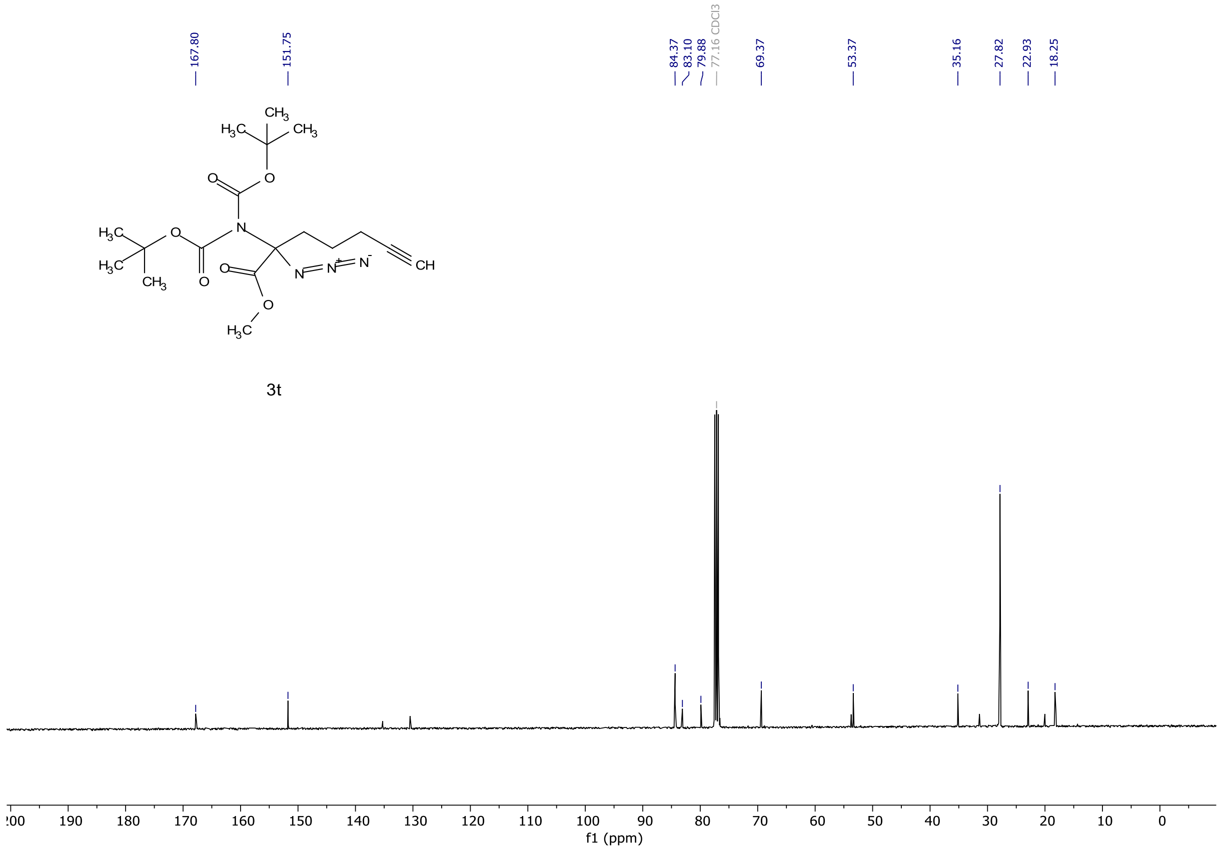

^{1}H NMR (400 MHz, $CDCl_3$) of compound **3u**

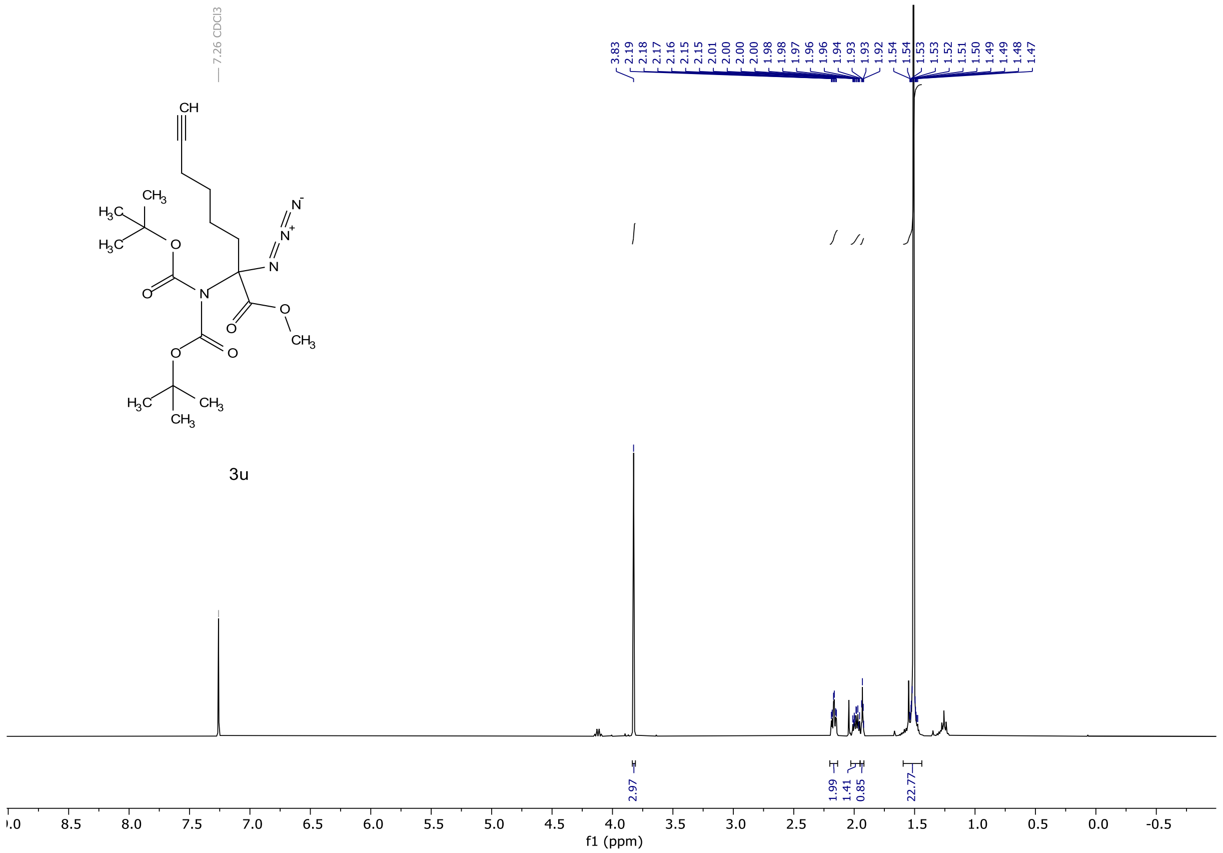

^{13}C NMR (400 MHz, $CDCl_3$) of compound **3u**

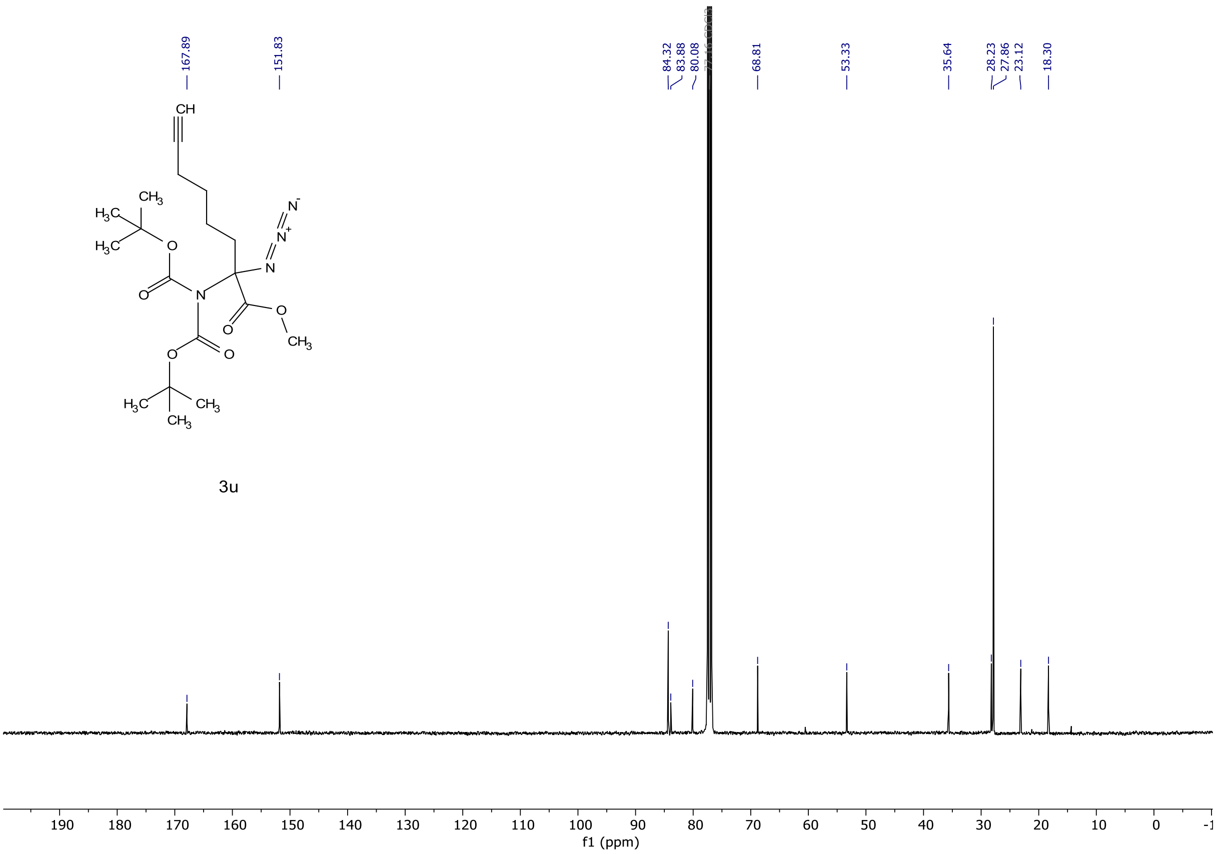

^{1}H NMR (400 MHz, $CDCl_3$) of compound **3v**

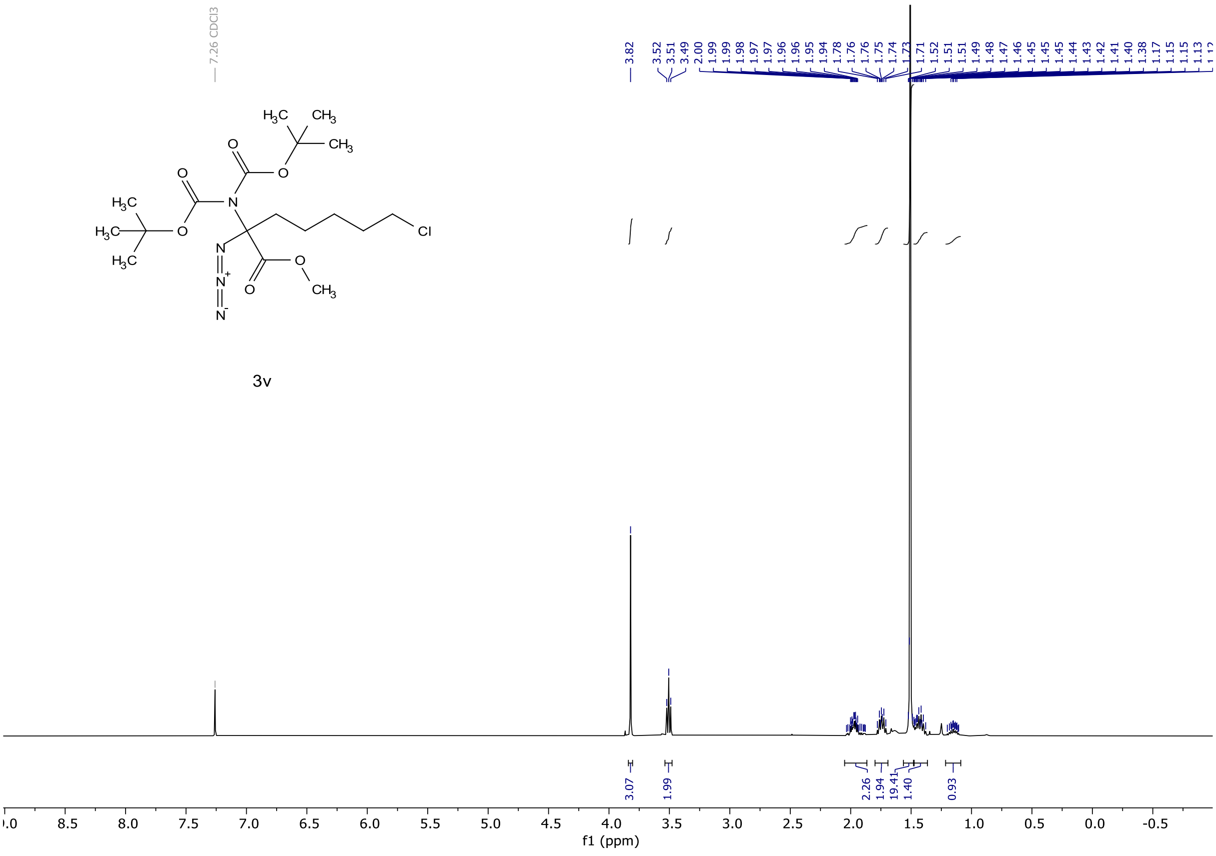

^{13}C NMR (400 MHz, $CDCl_3$) of compound **3v**

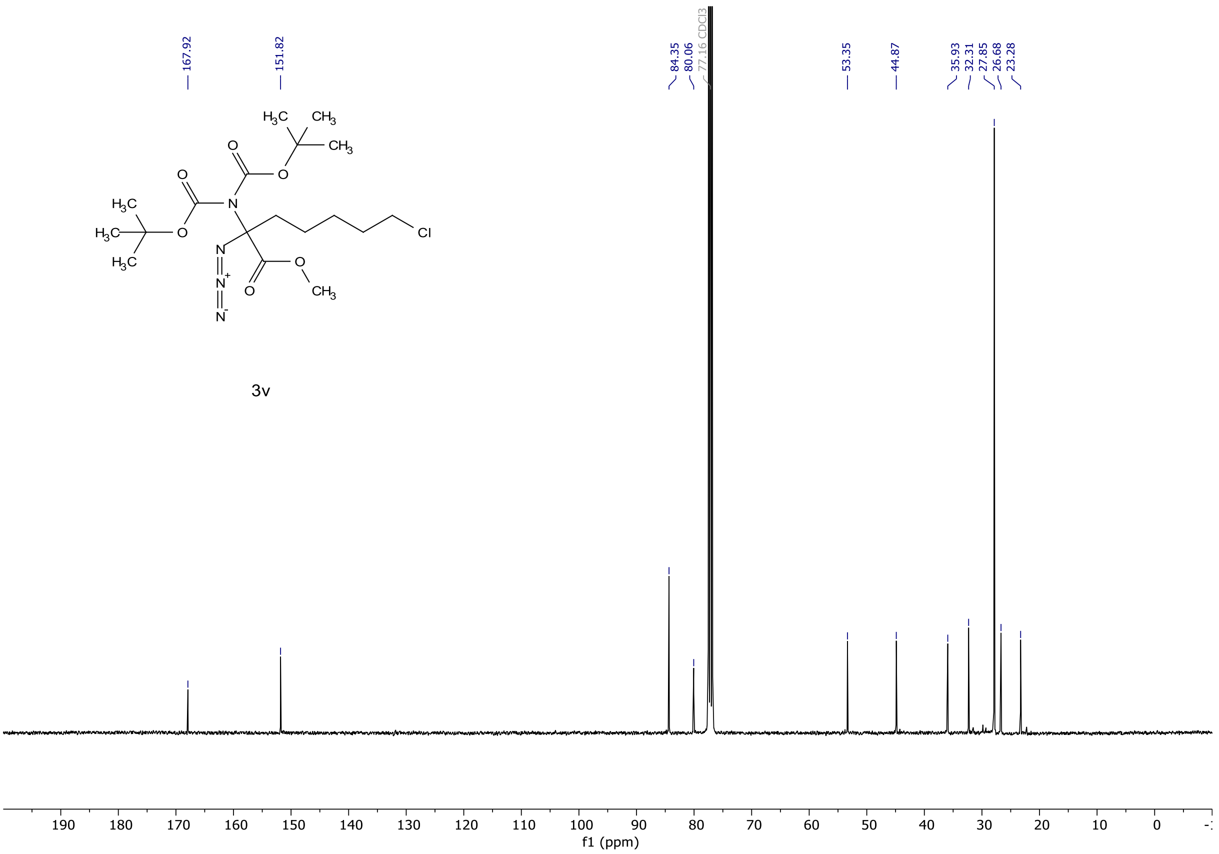

^{1}H NMR (400 MHz, $CDCl_3$) of compound **3w**

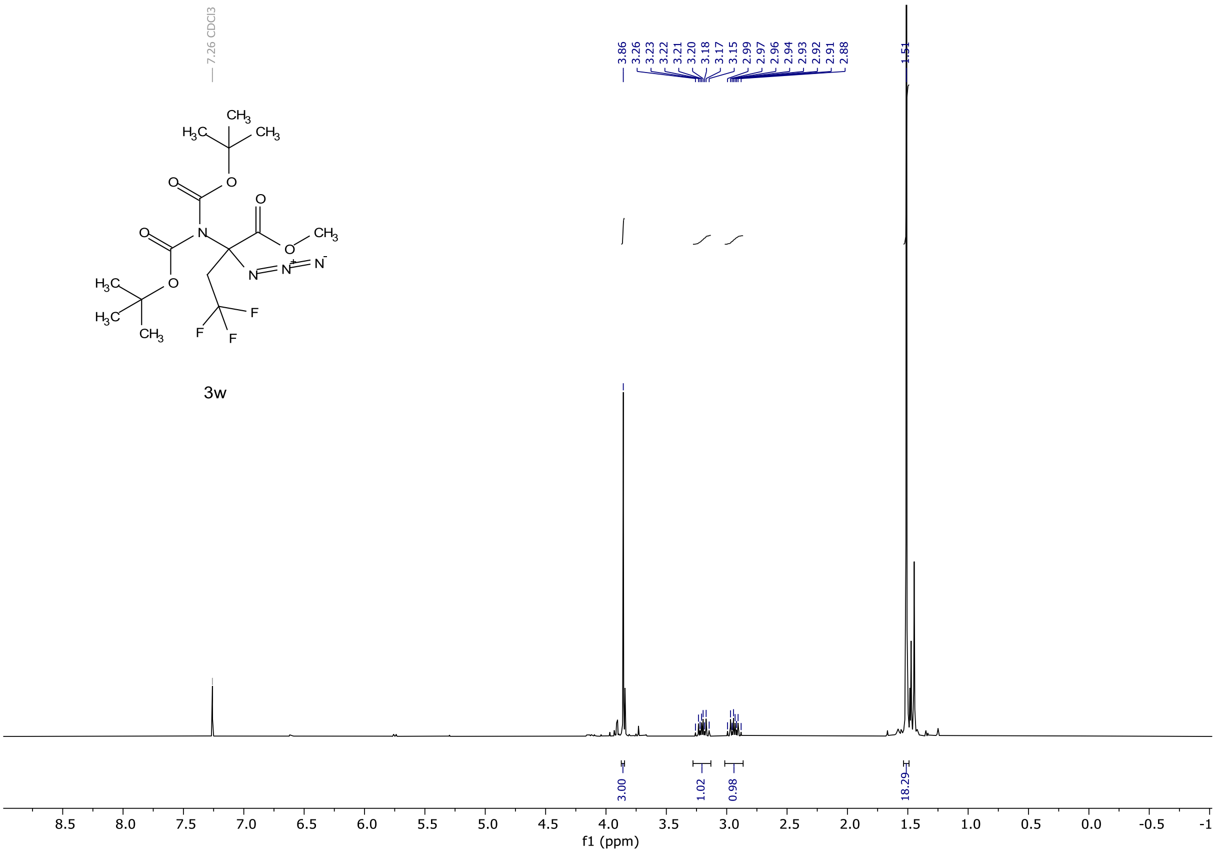

^{13}C NMR (400 MHz, $CDCl_3$) of compound **3w**

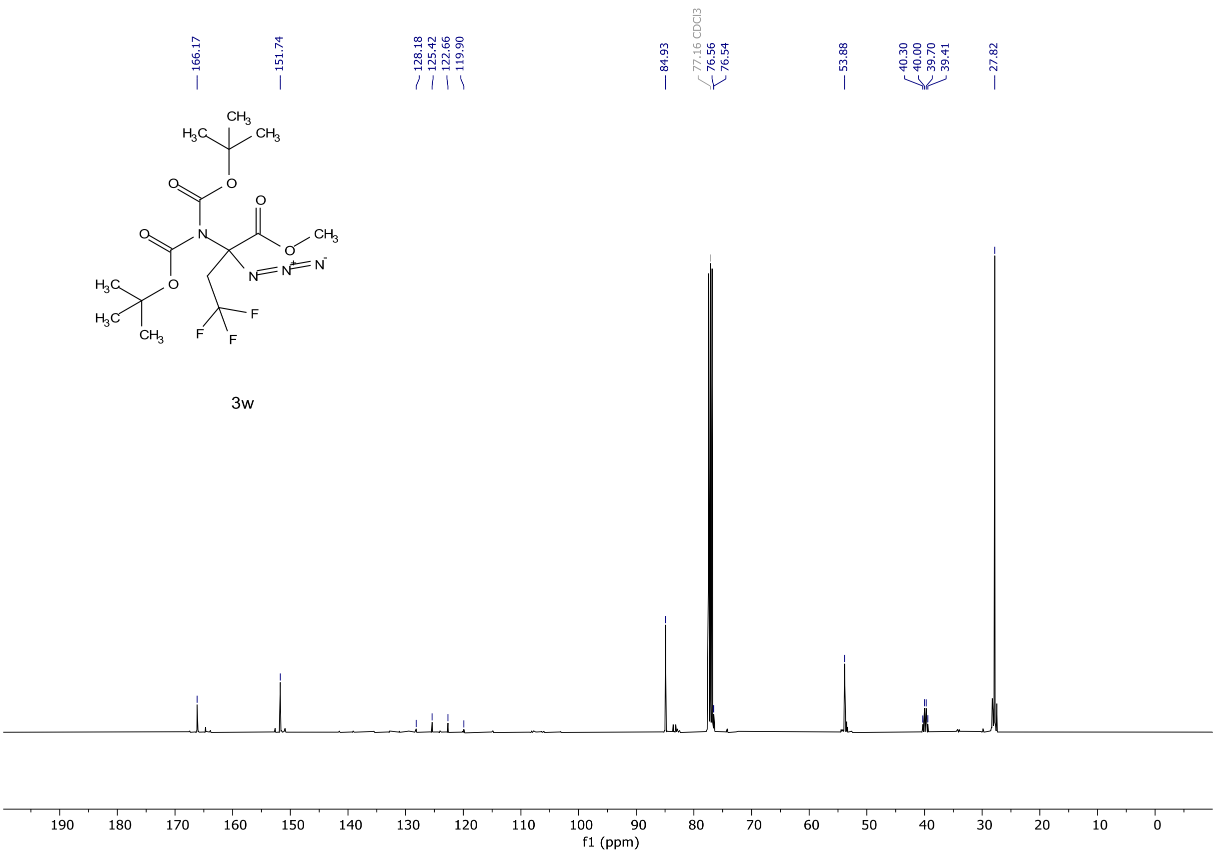

^{19}F NMR (376 MHz, $CDCl_3$) of compound **3w**

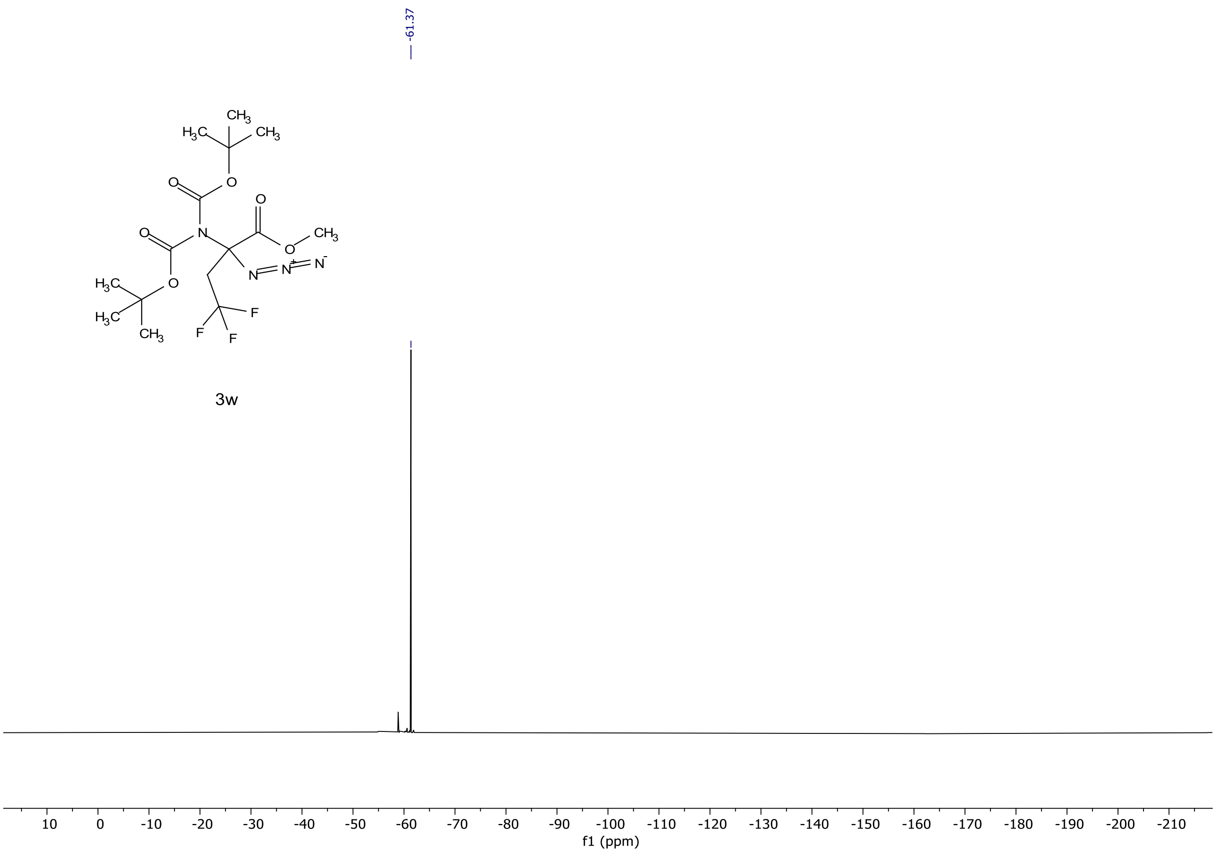

^{1}H NMR (400 MHz, $CDCl_3$) of compound **3x**

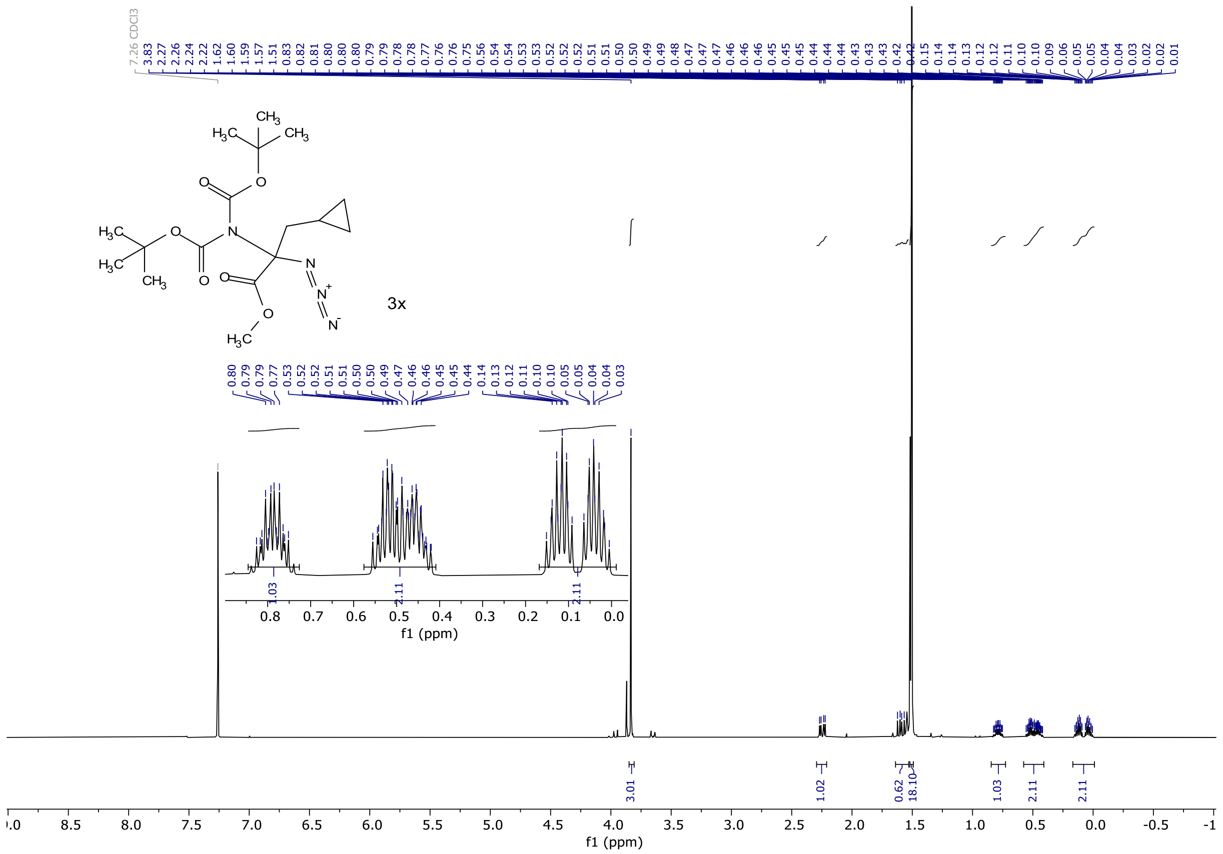

^{13}C NMR (400 MHz, $CDCl_3$) of compound **3x**

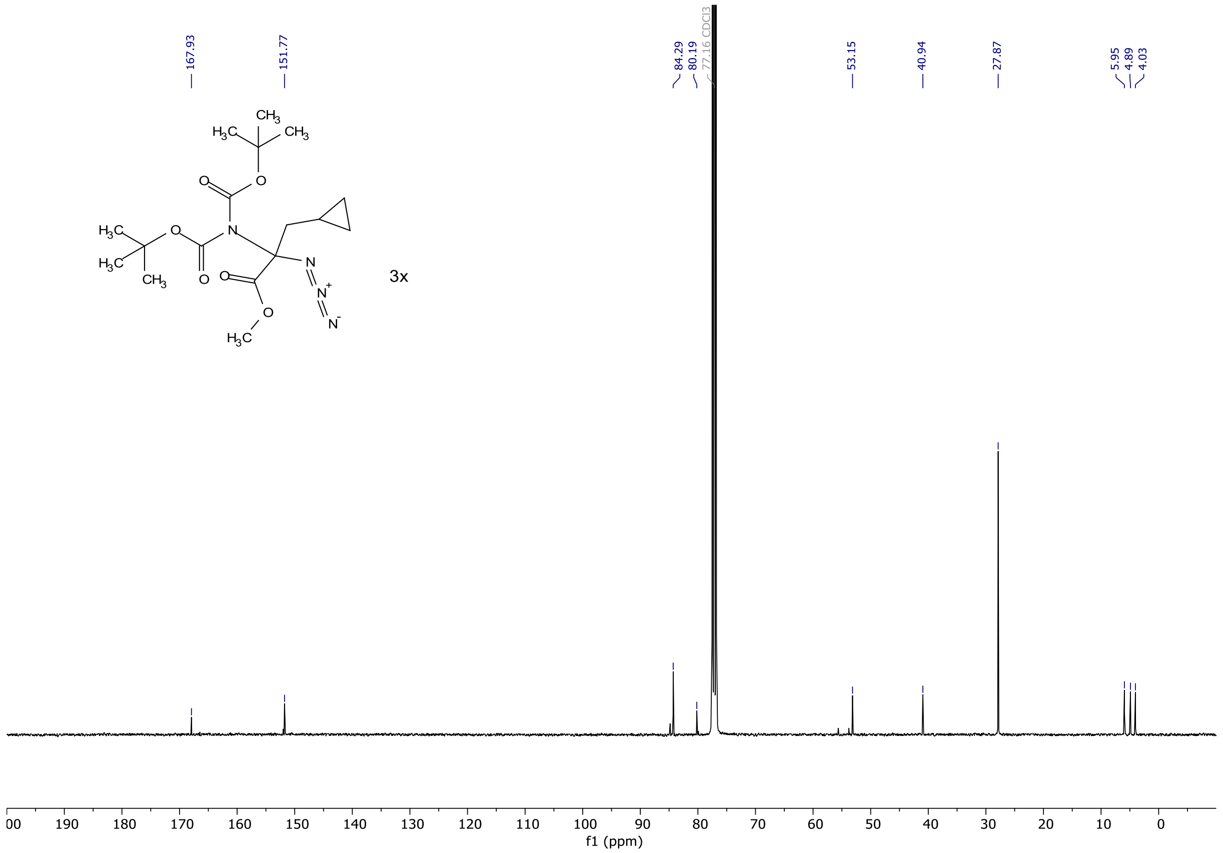

^{1}H NMR (400 MHz, $CDCl_3$) of compound **3y**

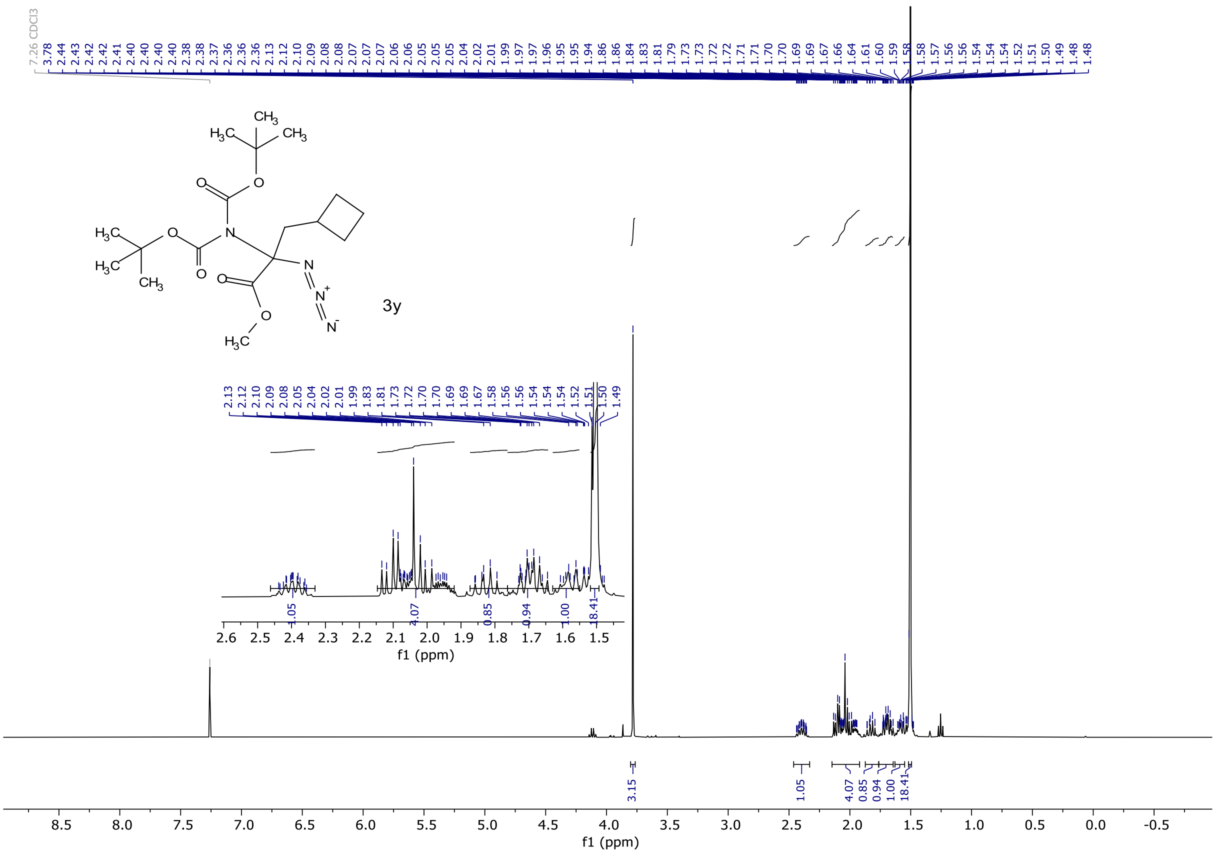

^{13}C NMR (400 MHz, $CDCl_3$) of compound **3y**

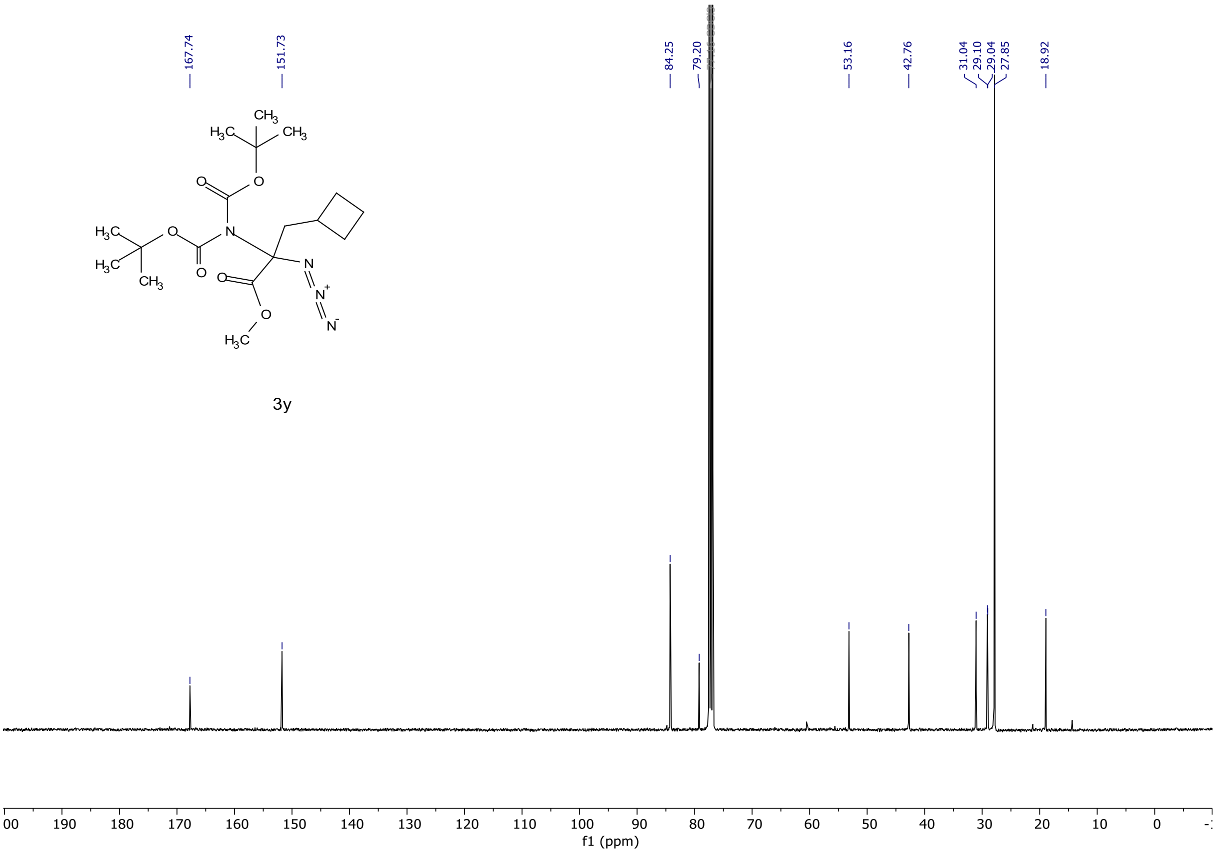

^{1}H NMR (400 MHz, $CDCl_3$) of compound 3z

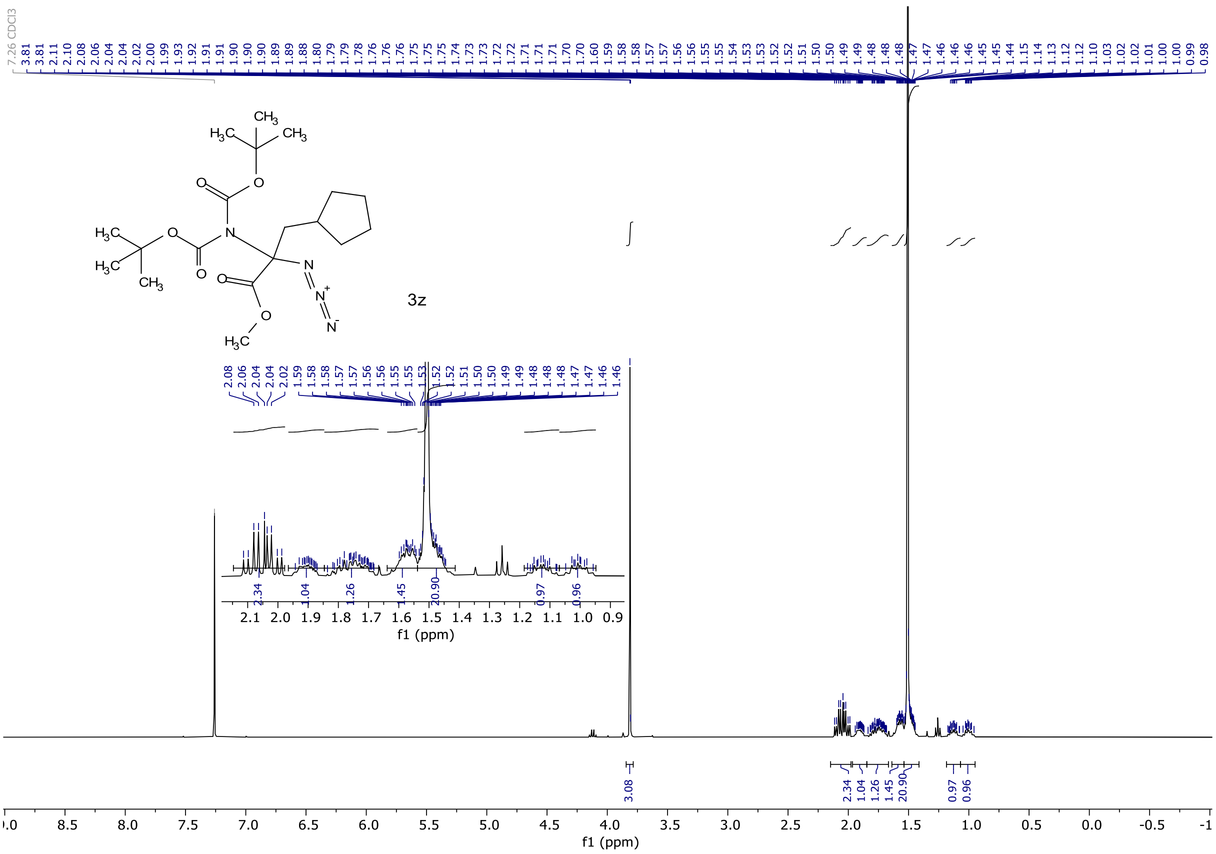

^{13}C NMR (400 MHz, $CDCl_3$) of compound 3z

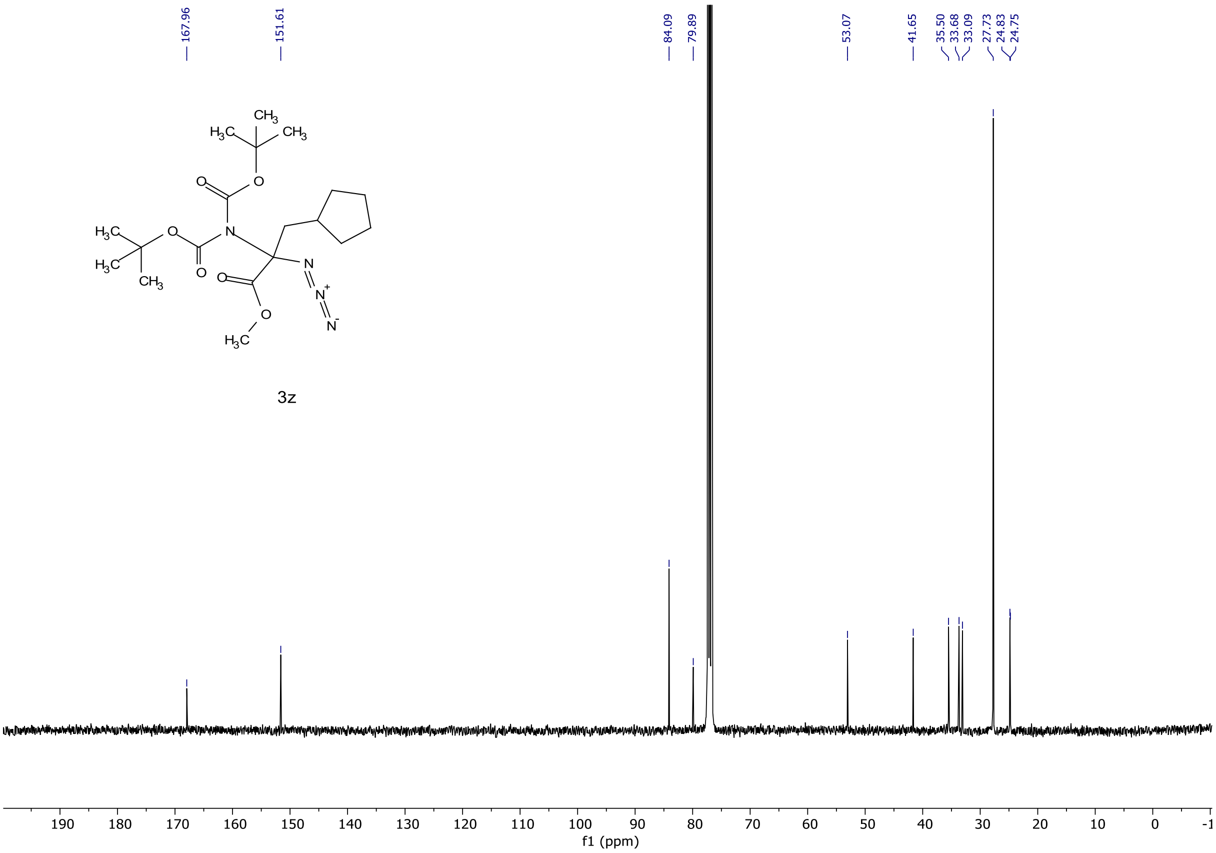

^{1}H NMR (400 MHz, $CDCl_3$) of compound **3aa**

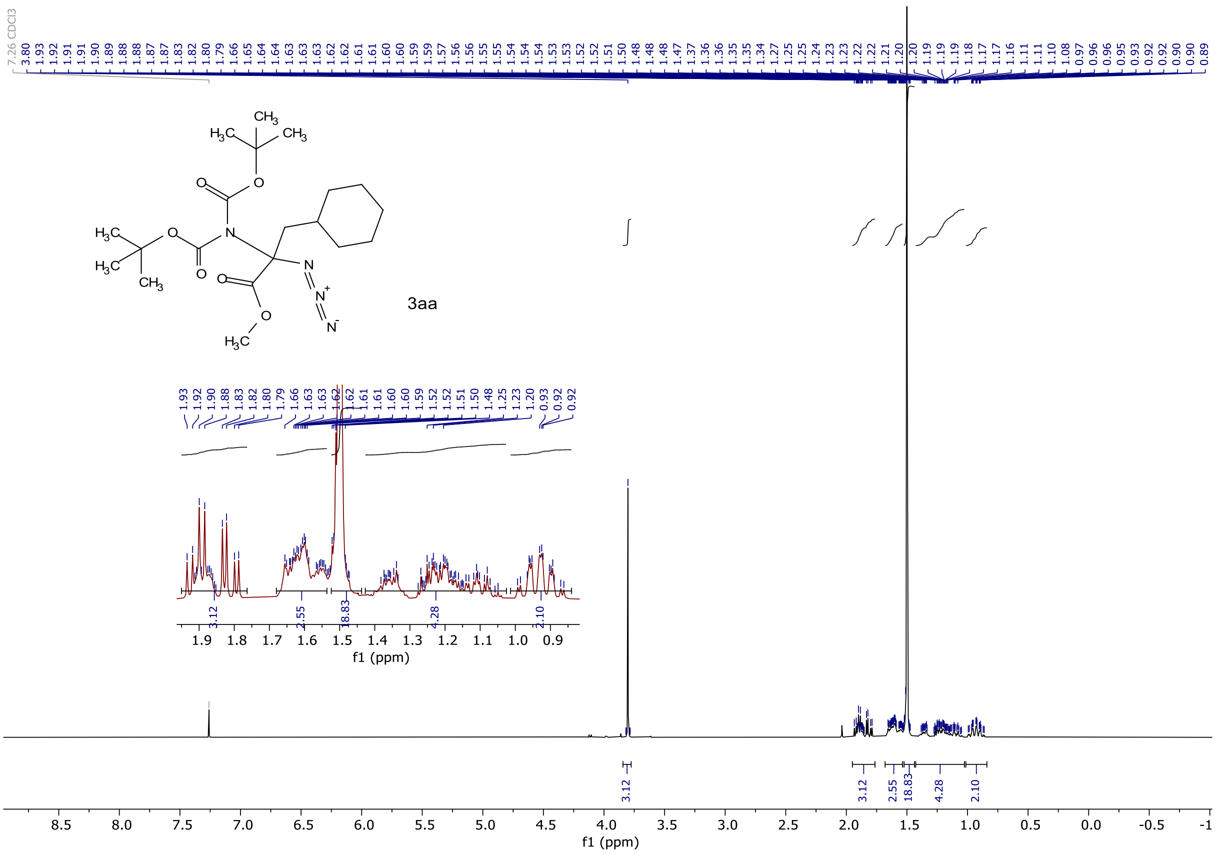

^{13}C NMR (400 MHz, $CDCl_3$) of compound **3aa**

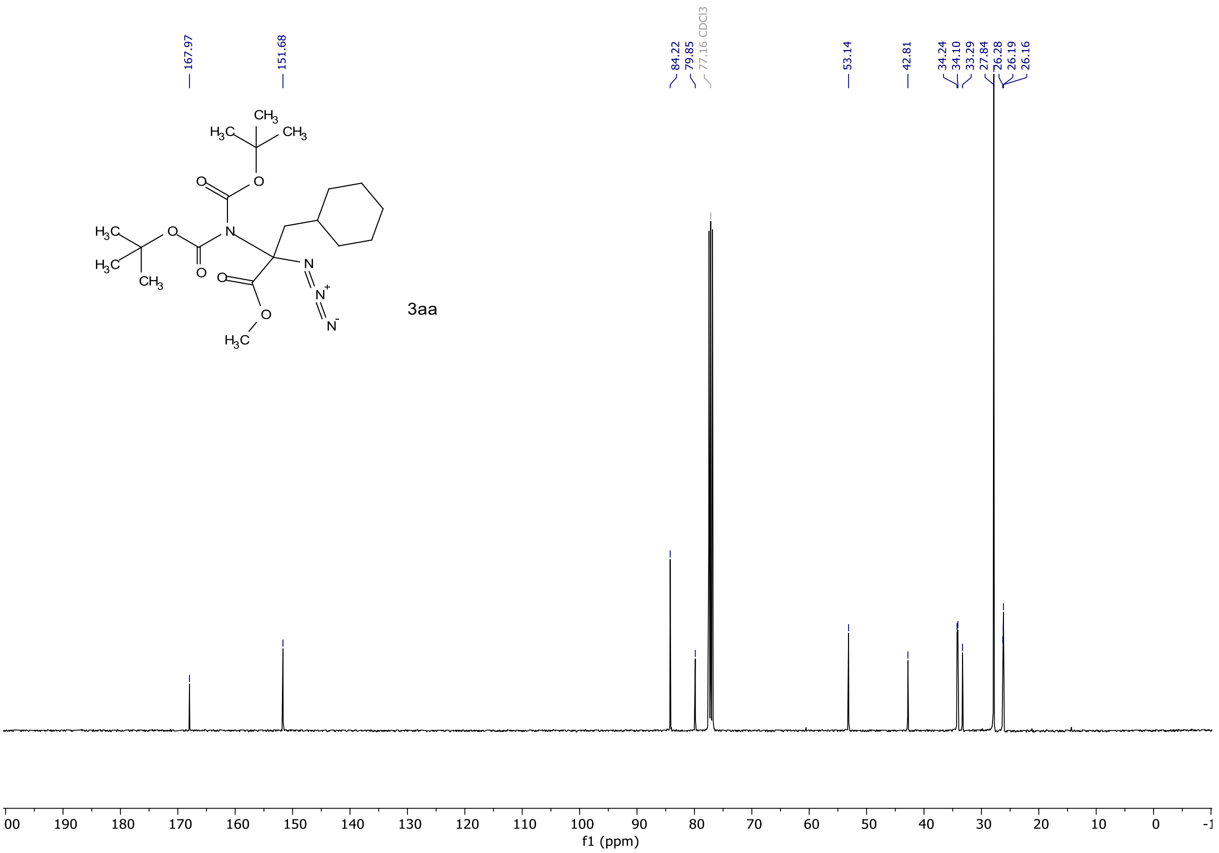

^{1}H NMR (400 MHz, $CDCl_3$) of compound **3ab**

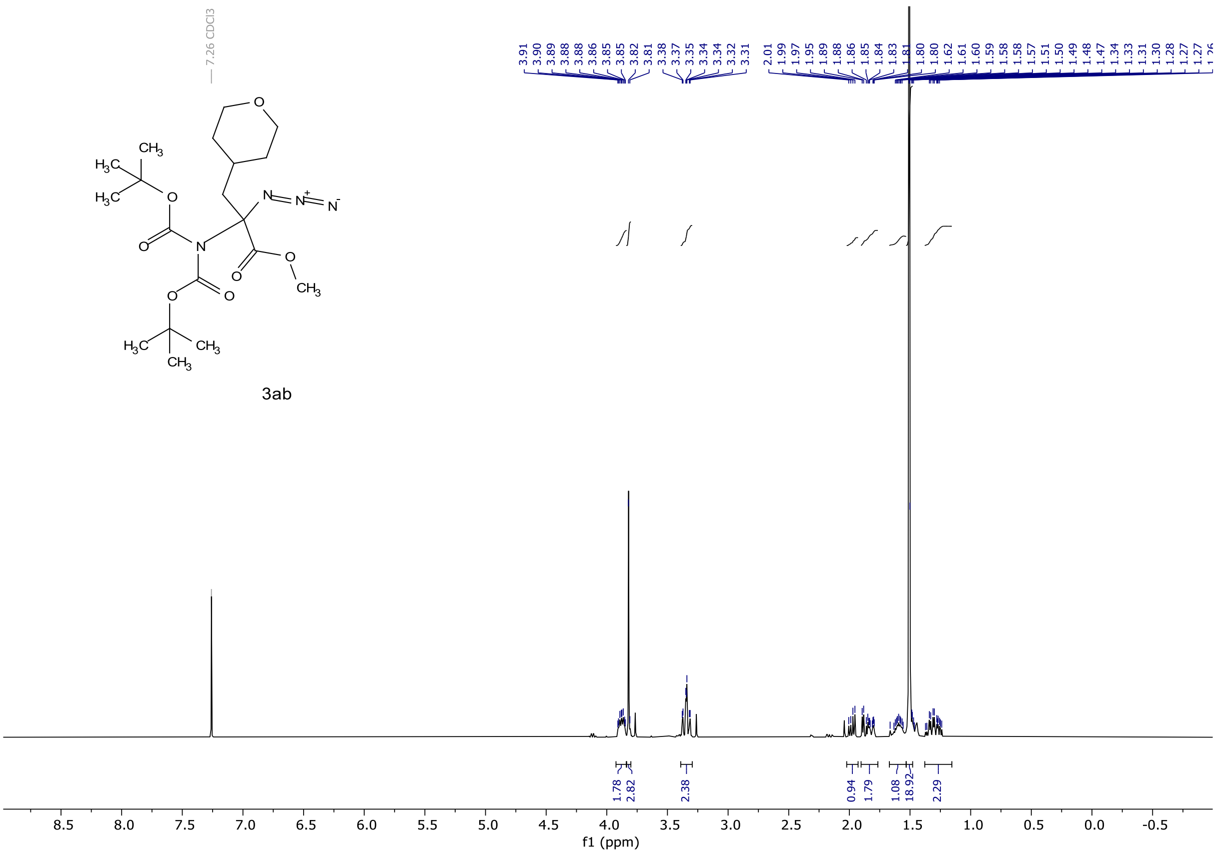

^{13}C NMR (400 MHz, $CDCl_3$) of compound **3ab**

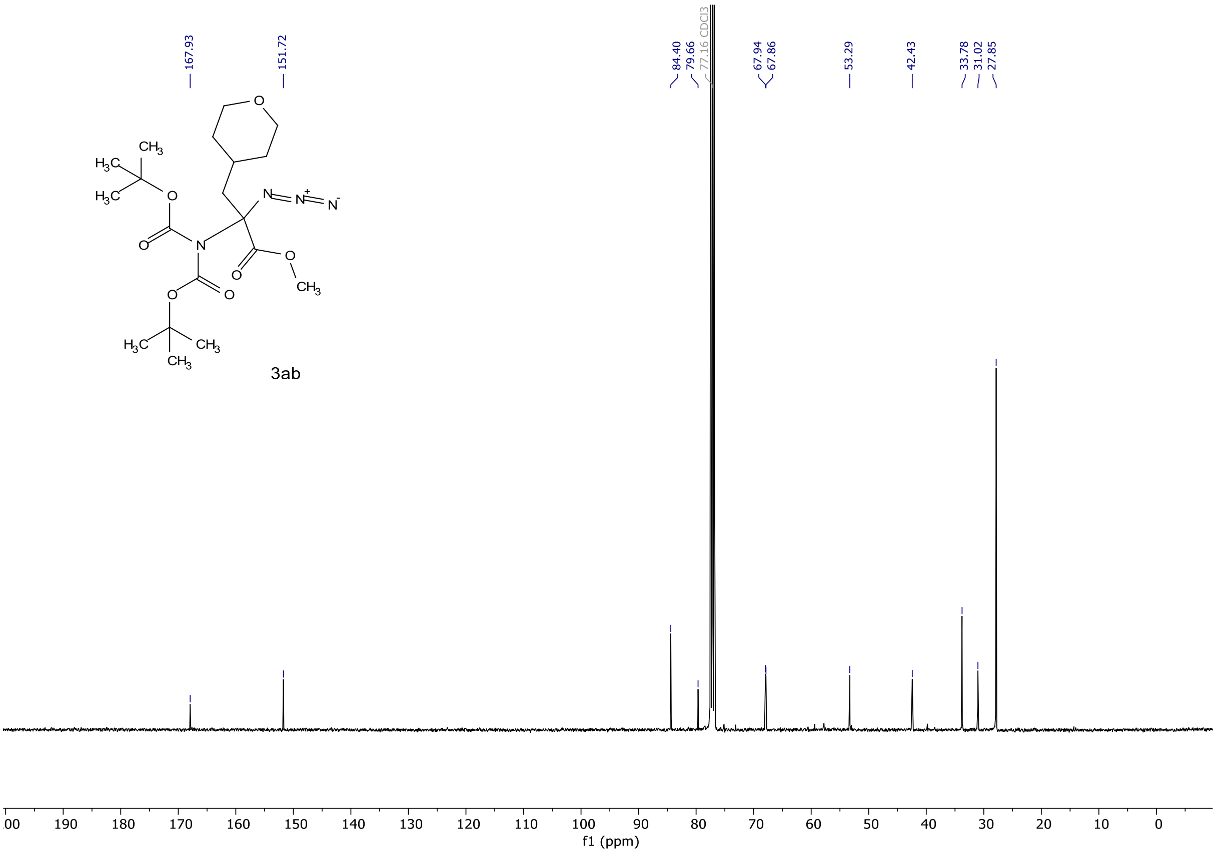

^{1}H NMR (400 MHz, $CDCl_3$) of compound **3ac**

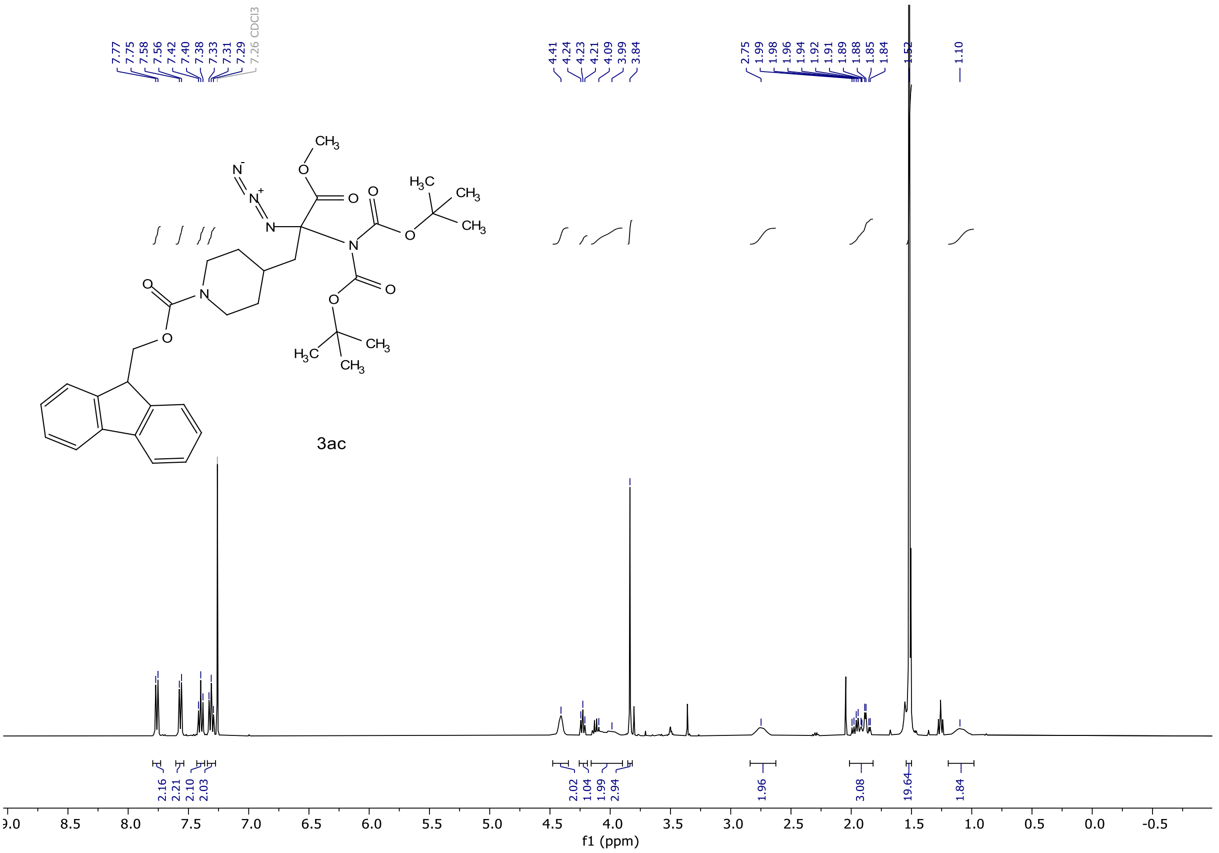

^{13}C NMR (400 MHz, $CDCl_3$) of compound **3ac**

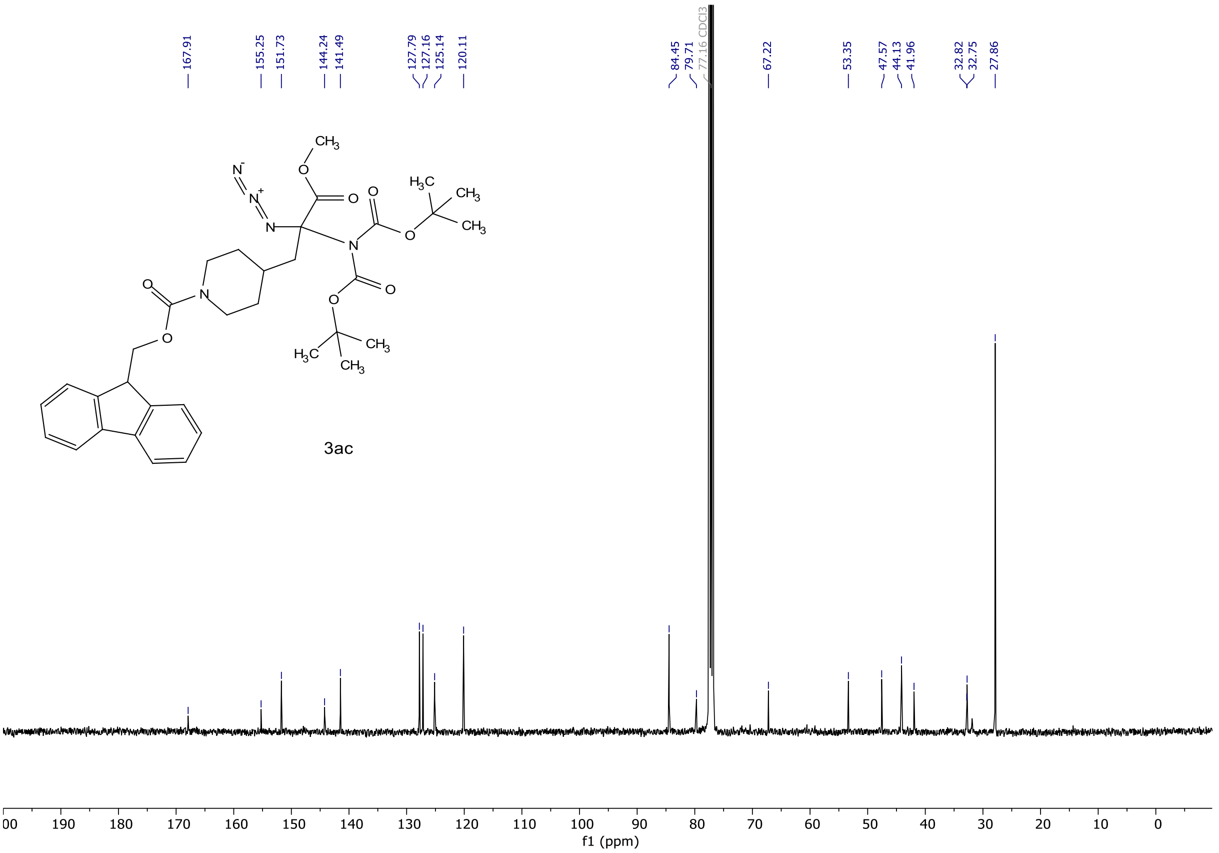

¹H NMR (400 MHz, $CDCl_3$) of compound **3ad**

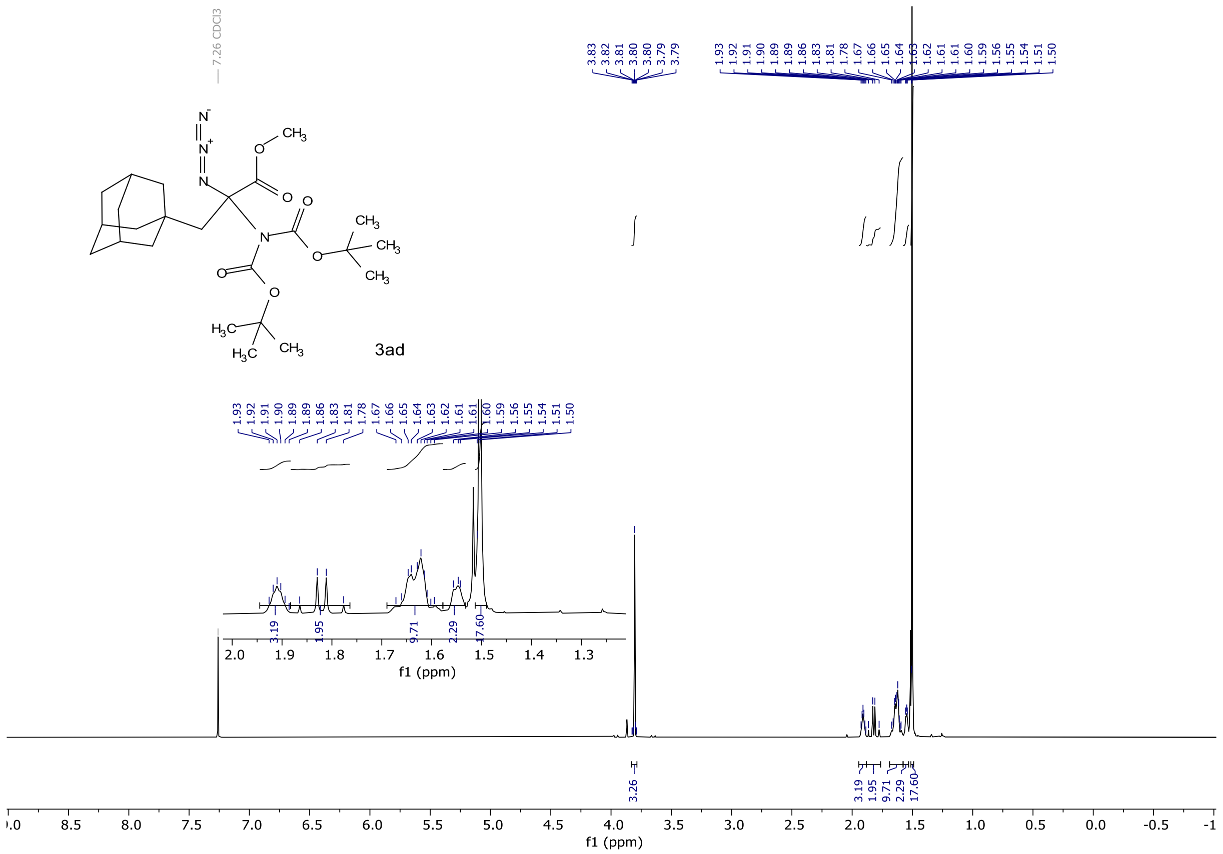

¹³C NMR (400 MHz, $CDCl_3$) of compound **3ad**

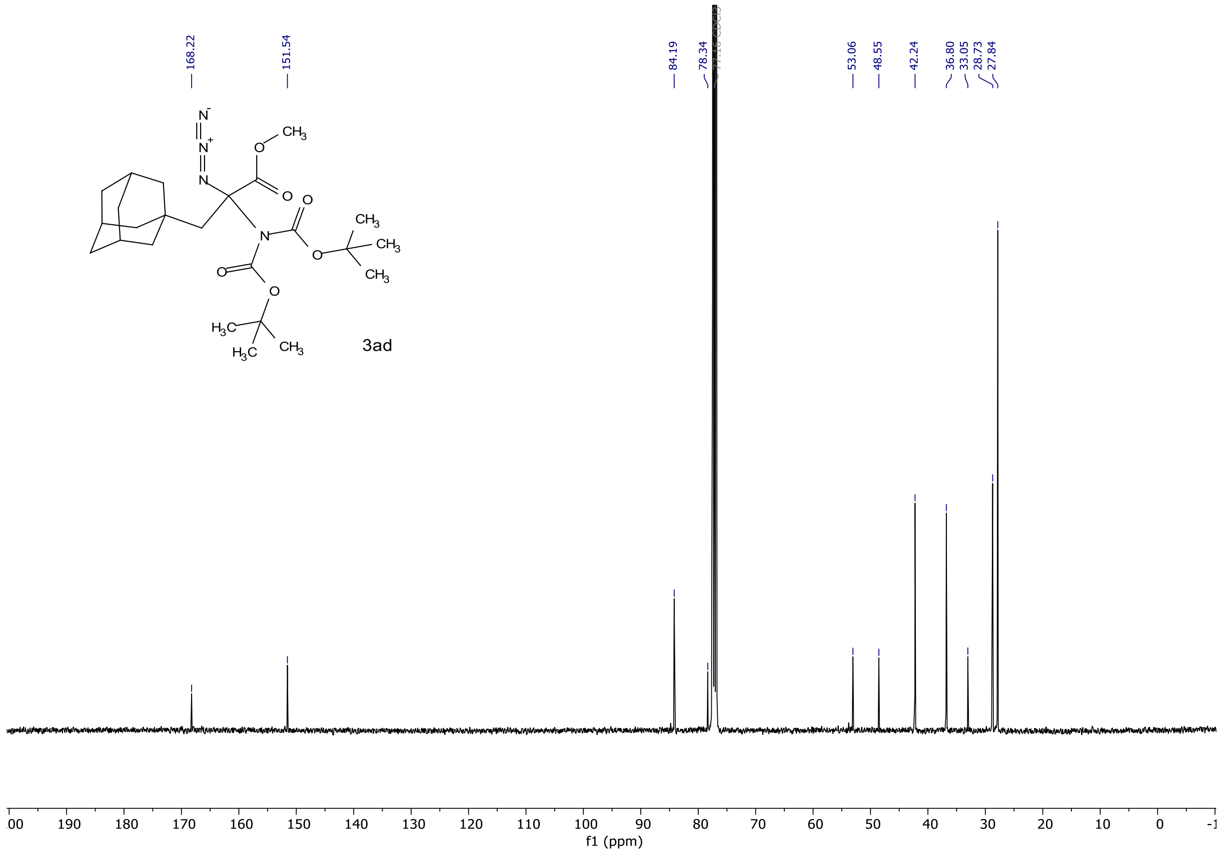

^{1}H NMR (400 MHz, $CDCl_3$) of compound **3ae**

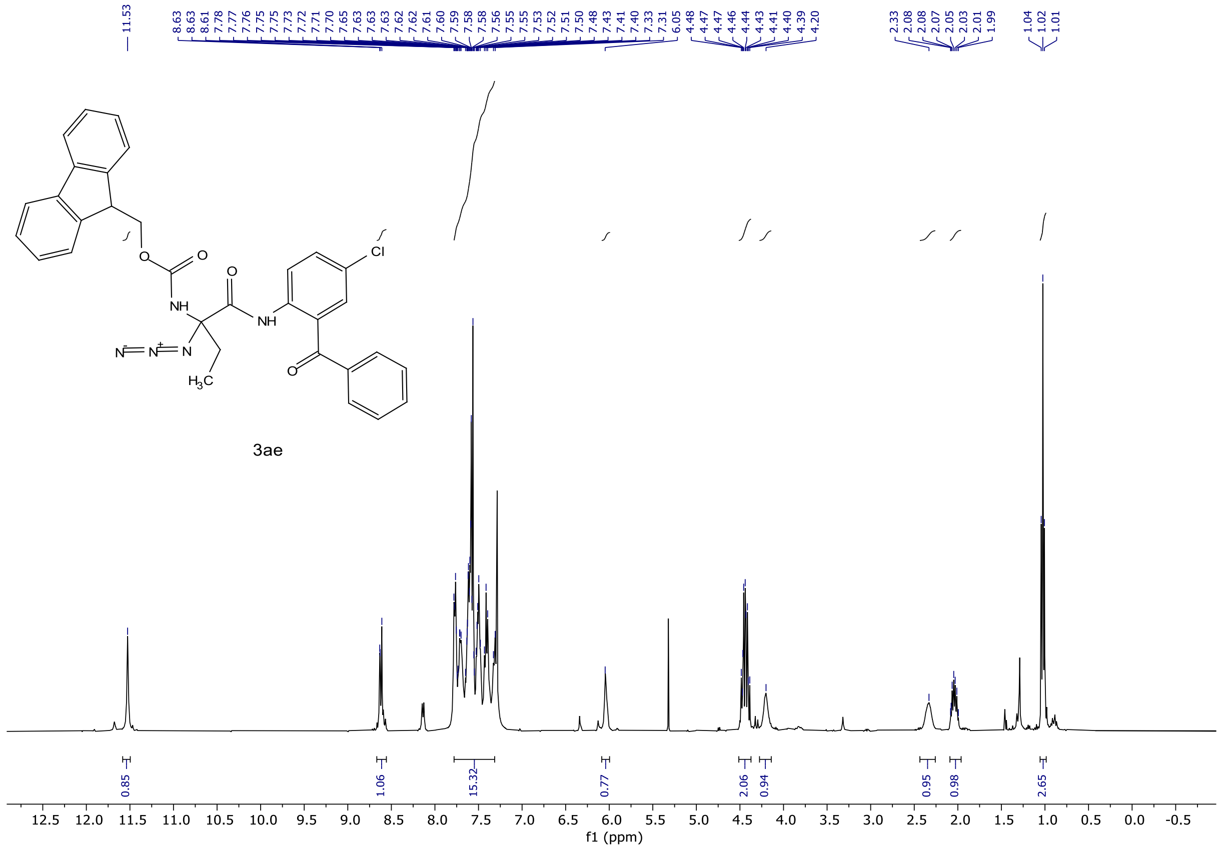

^{13}C NMR (400 MHz, $CDCl_3$) of compound **3ae**

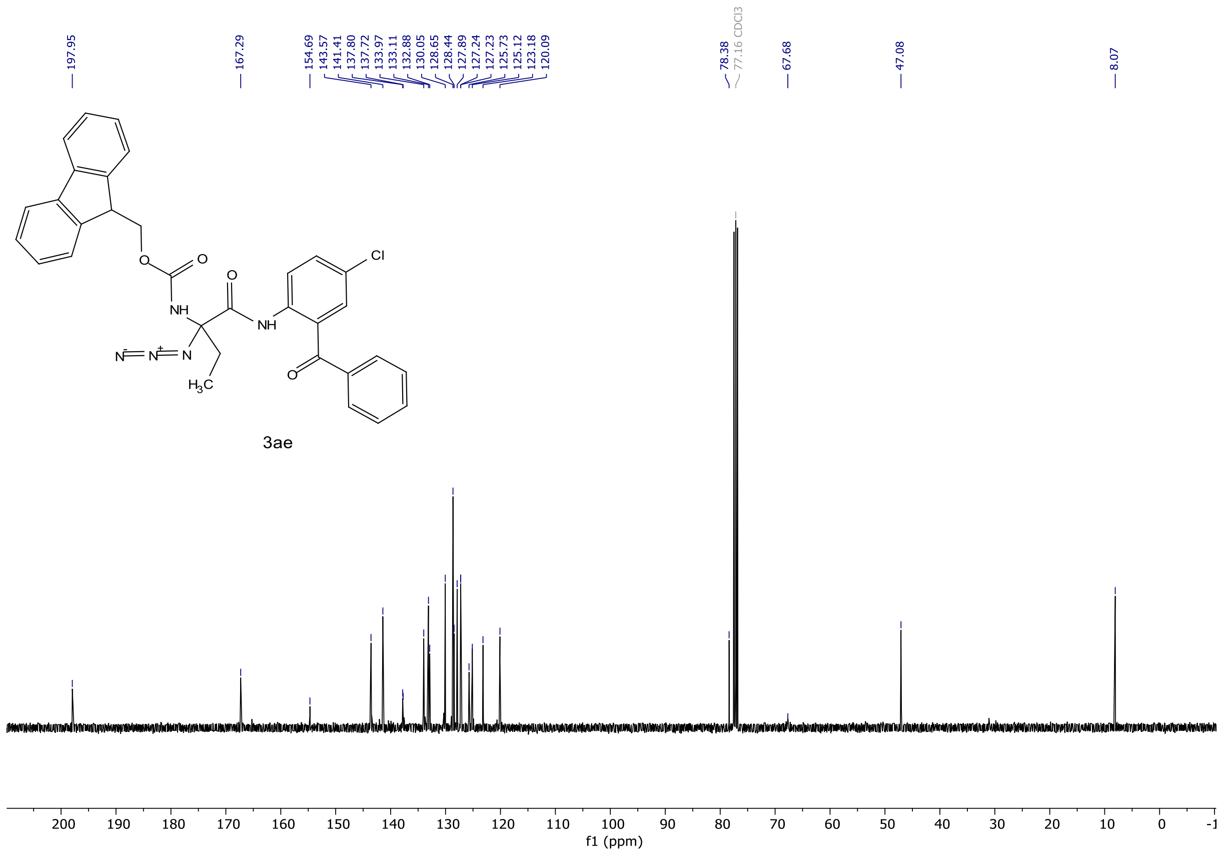

^{1}H NMR (400 MHz, $CDCl_3$) of compound **3af**

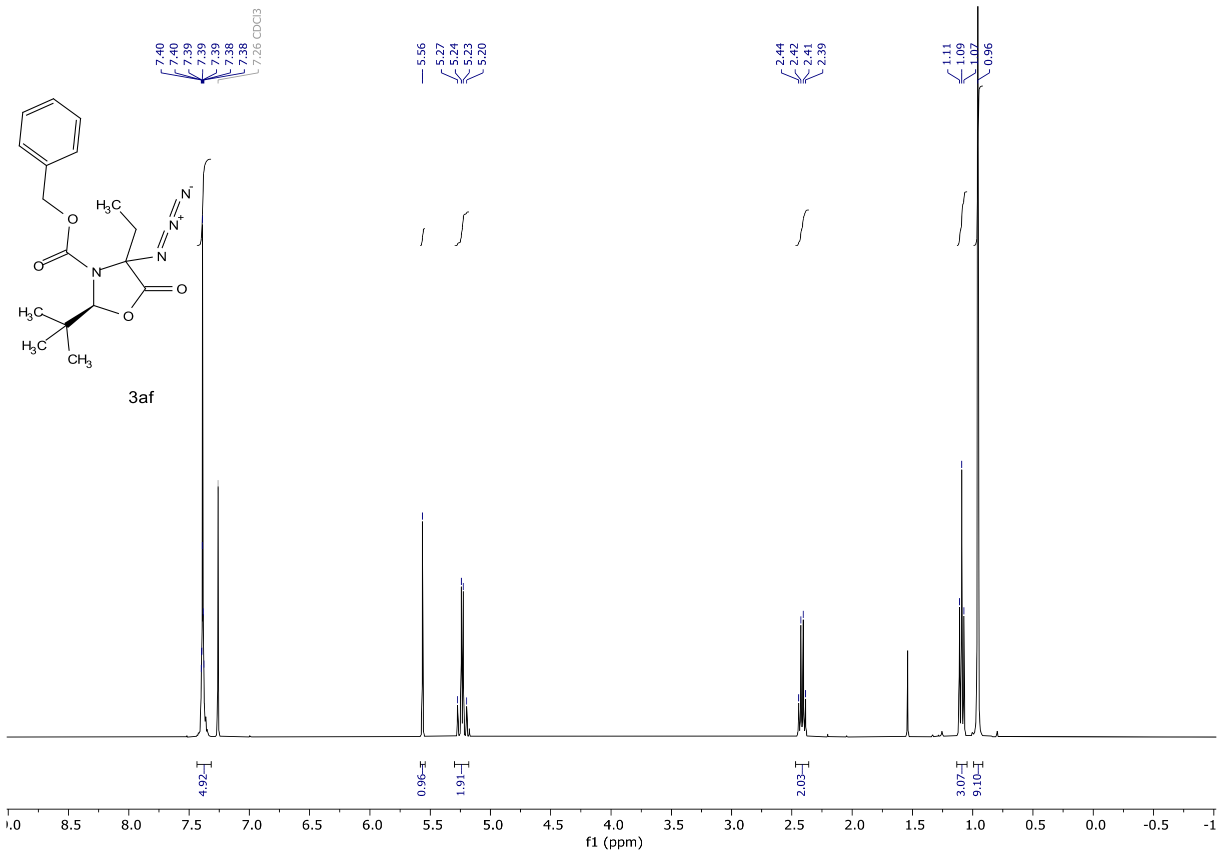

^{13}C NMR (400 MHz, $CDCl_3$) of compound **3af**

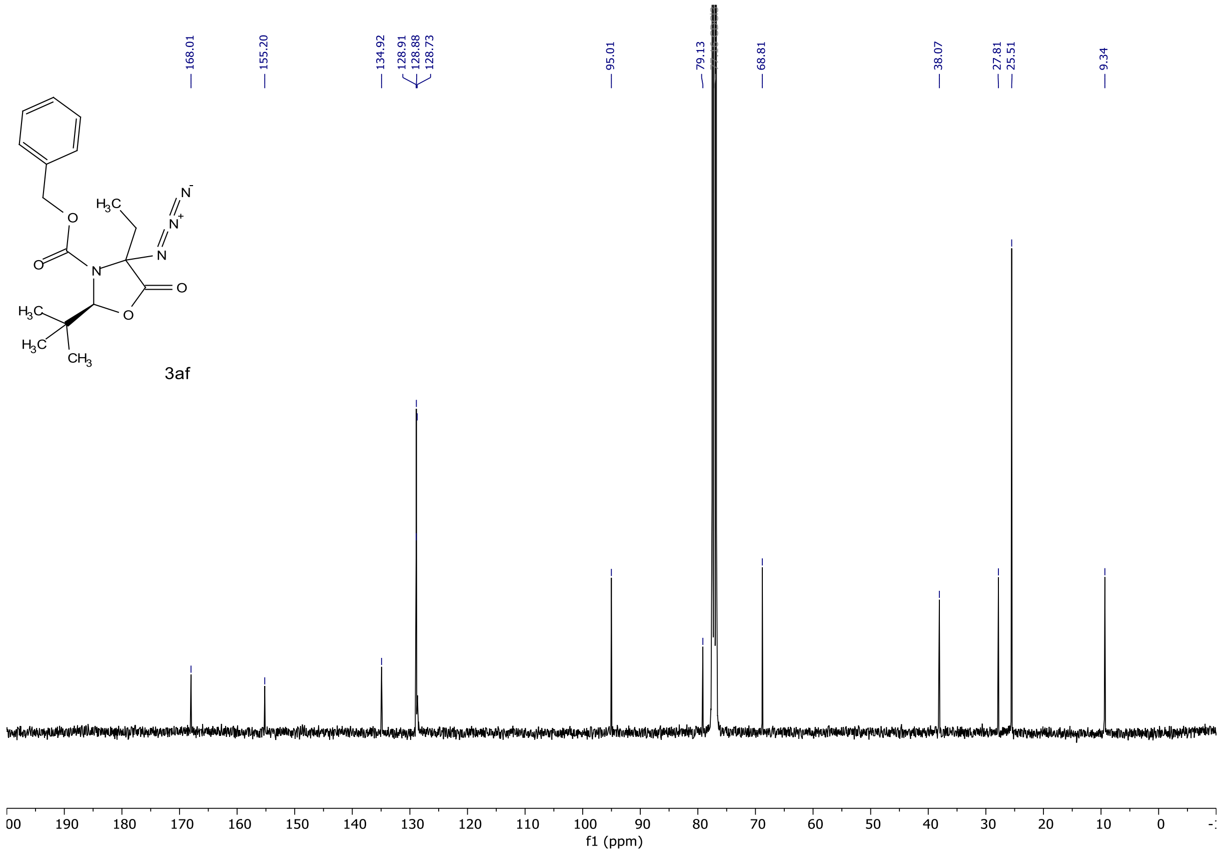

^{1}H NMR (400 MHz, $CDCl_3$) of compound **3ag**

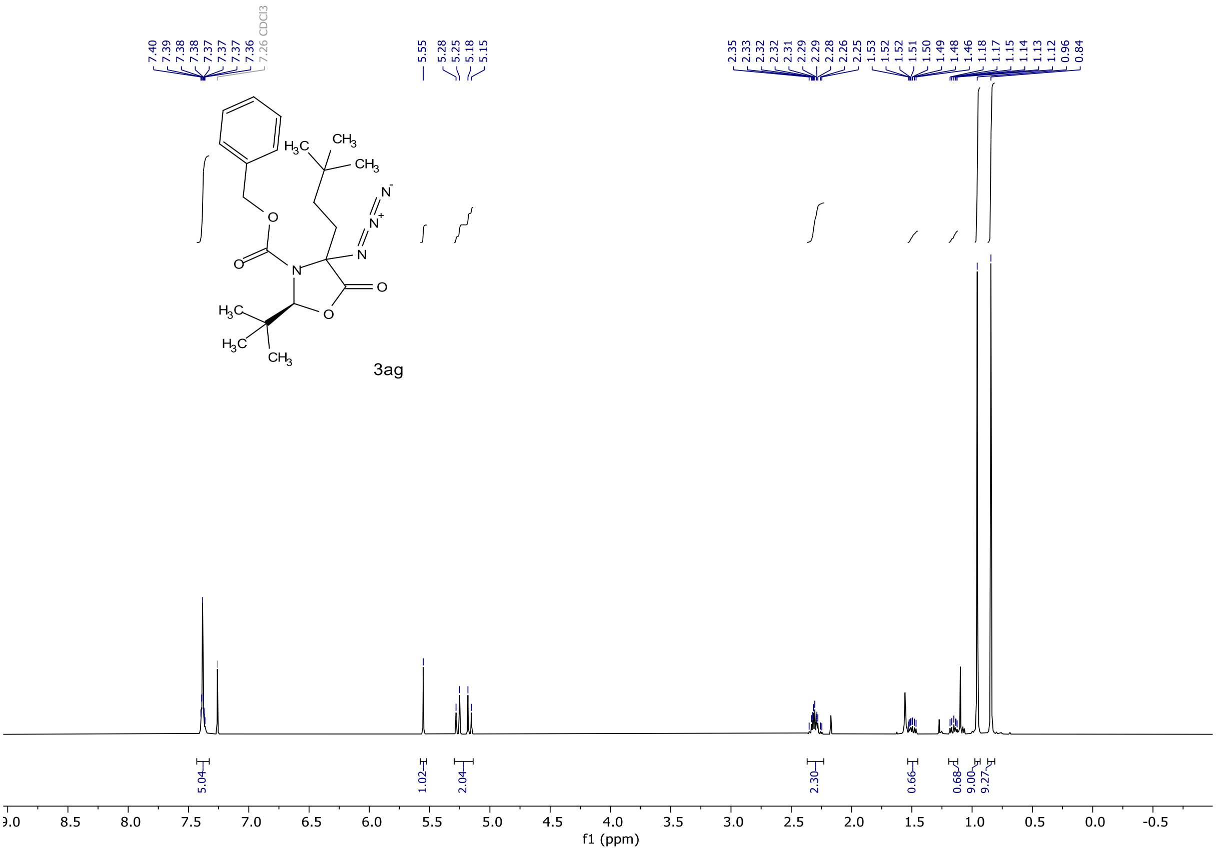

^{13}C NMR (400 MHz, $CDCl_3$) of compound **3ag**

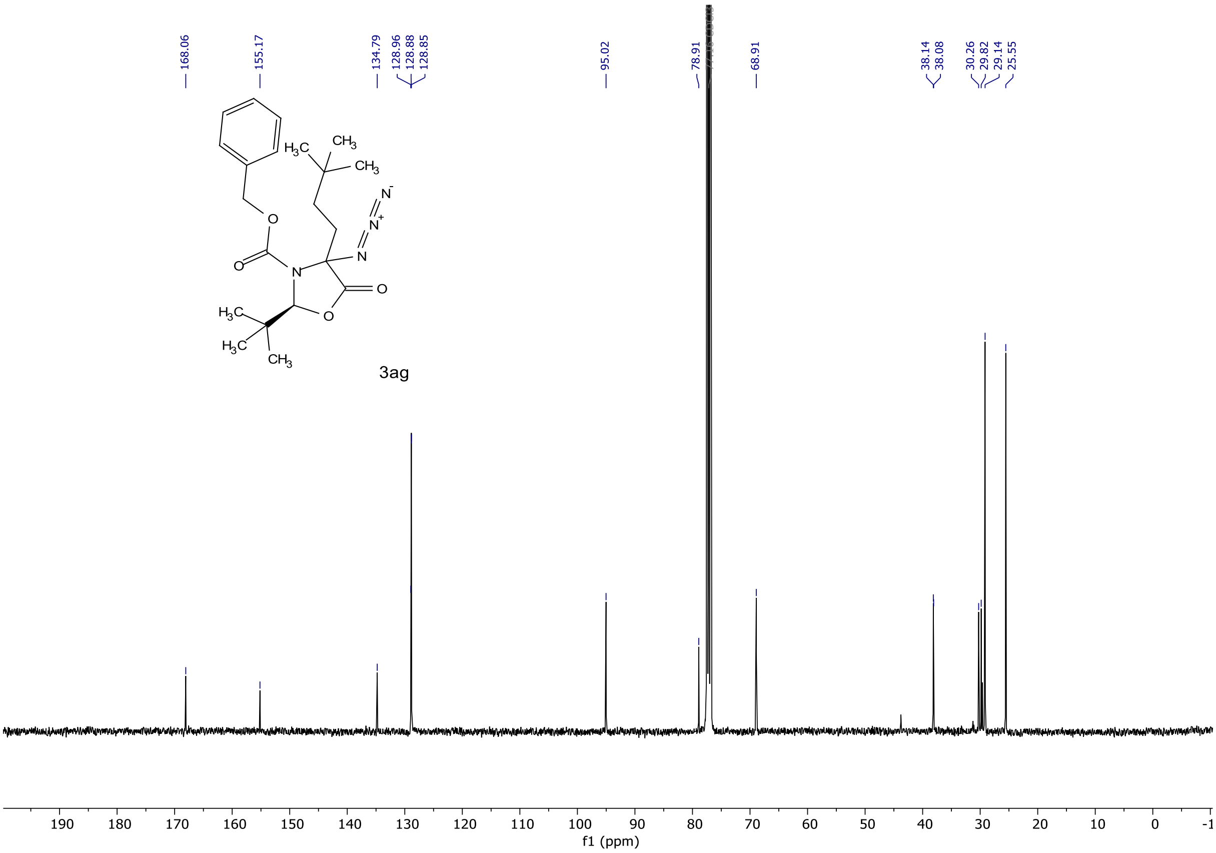

^{1}H NMR (400 MHz, $CDCl_3$) of compound **3ah**

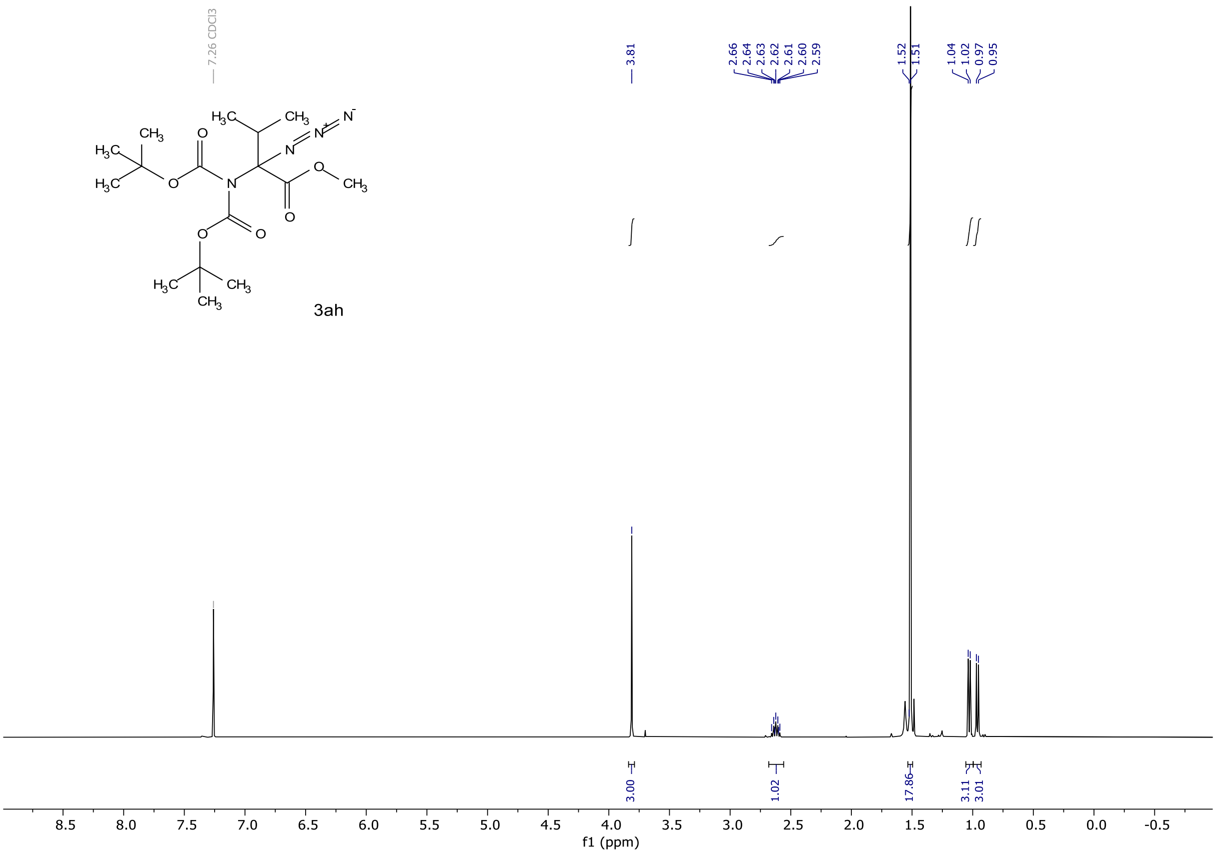

^{13}C NMR (400 MHz, $CDCl_3$) of compound **3ah**

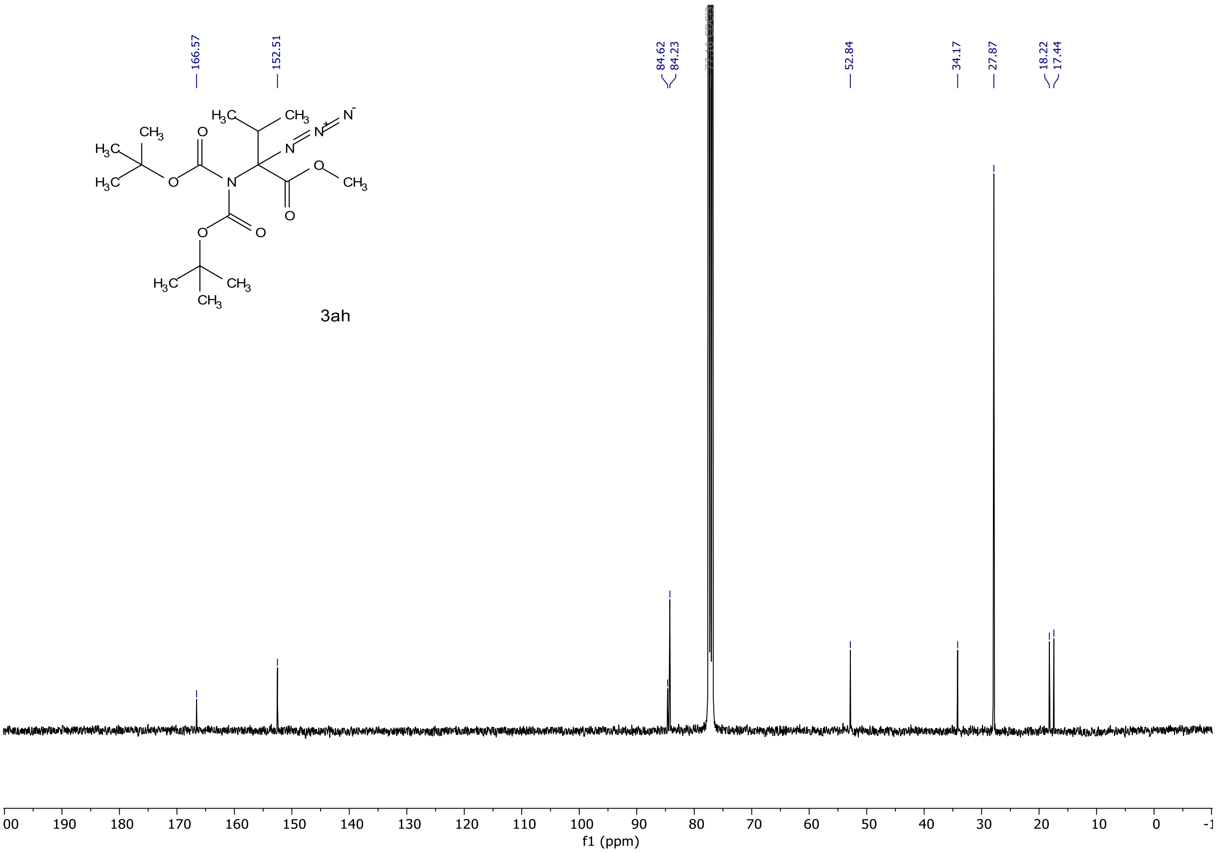

^{1}H NMR (400 MHz, $CDCl_3$) of compound **5a**

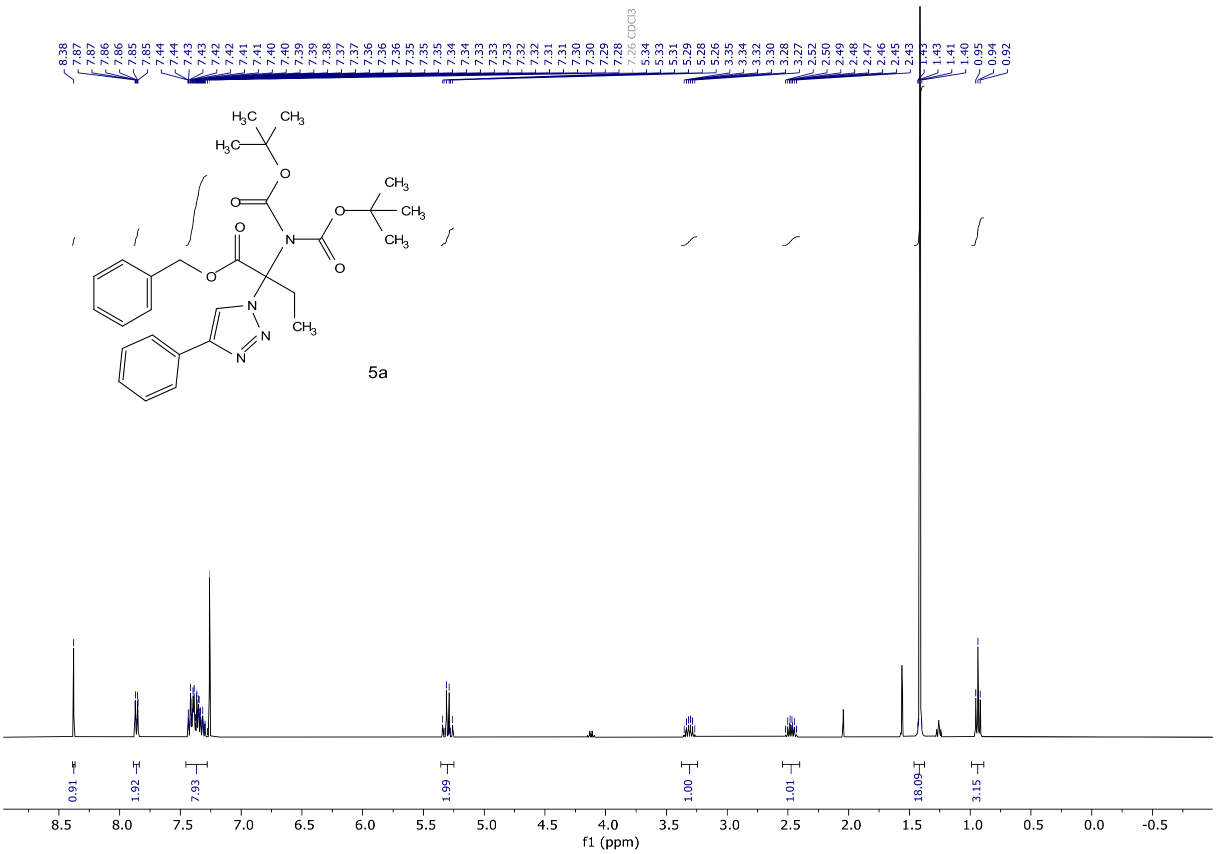

^{13}C NMR (400 MHz, $CDCl_3$) of compound **5a**

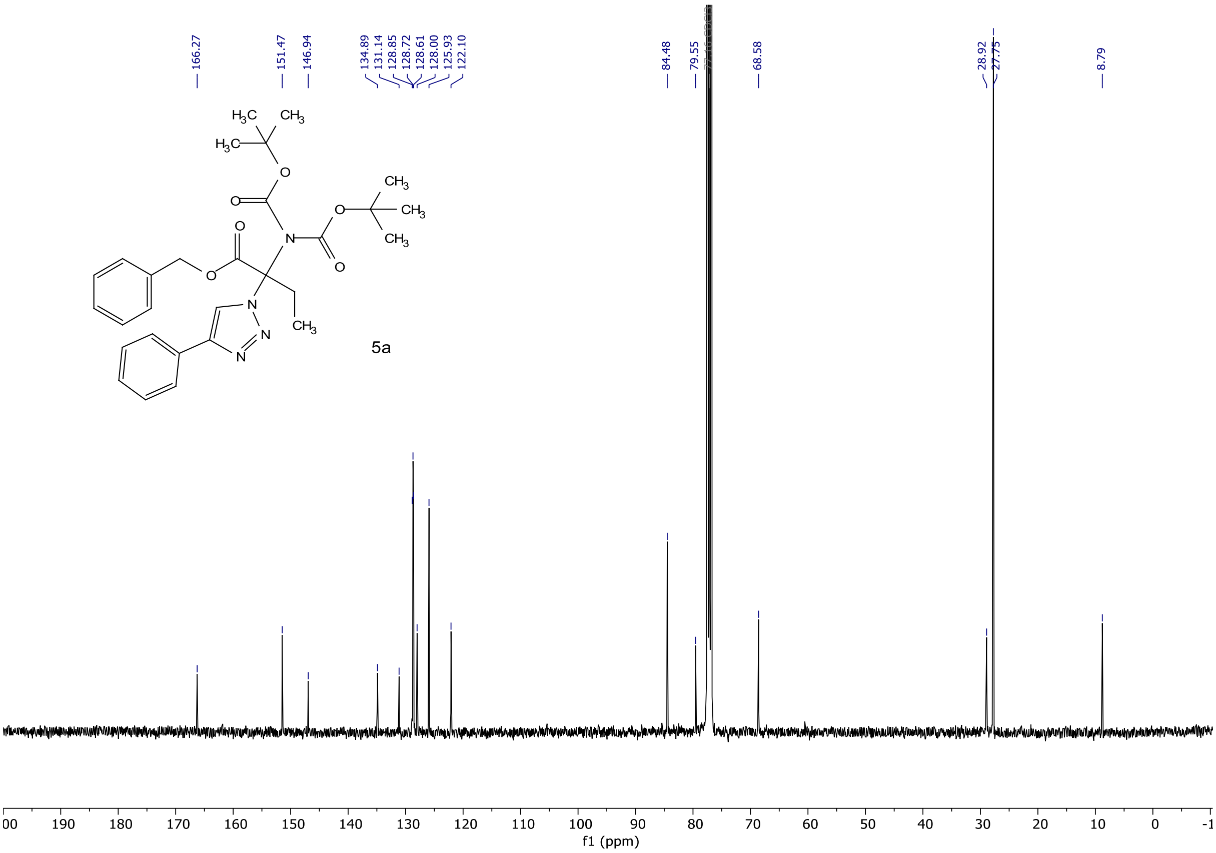

^{1}H NMR (400 MHz, $CDCl_3$) of compound **5b**

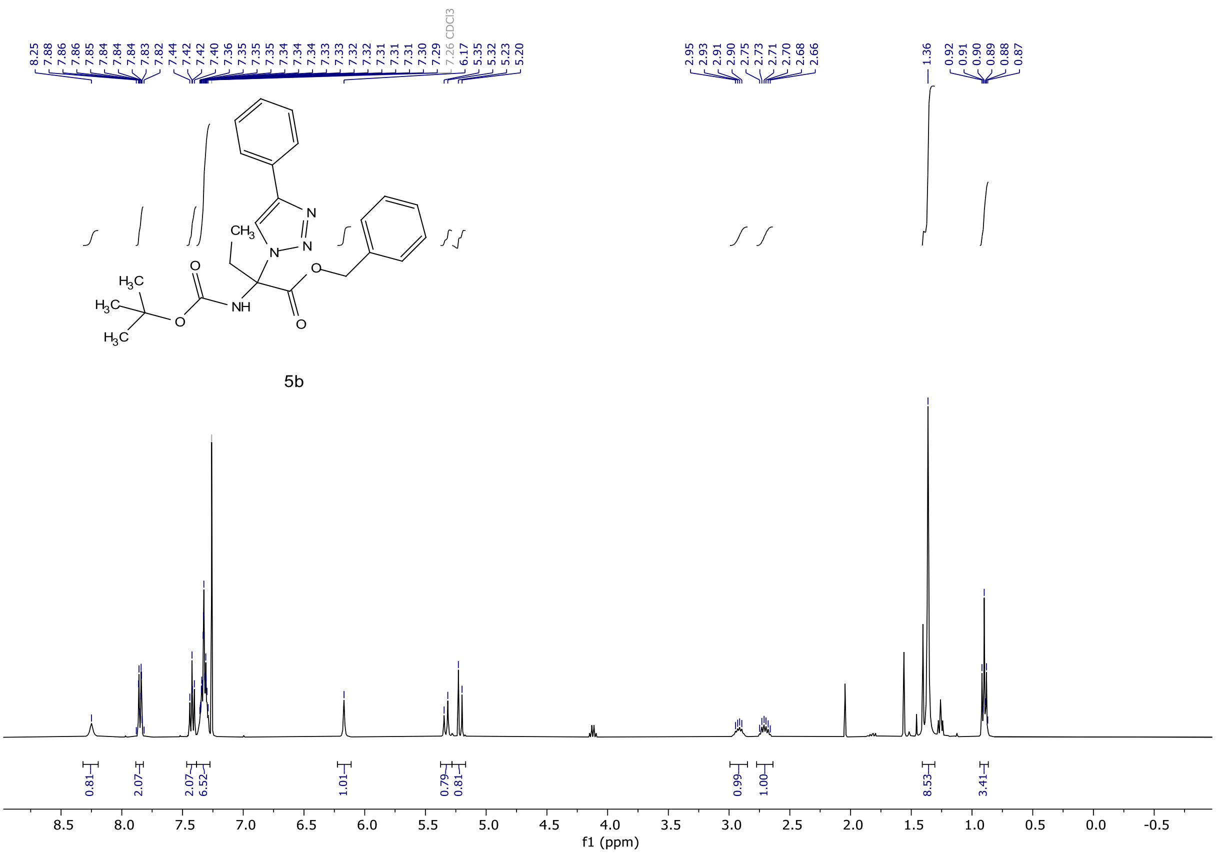

^{13}C NMR (400 MHz, $CDCl_3$) of compound **5b**

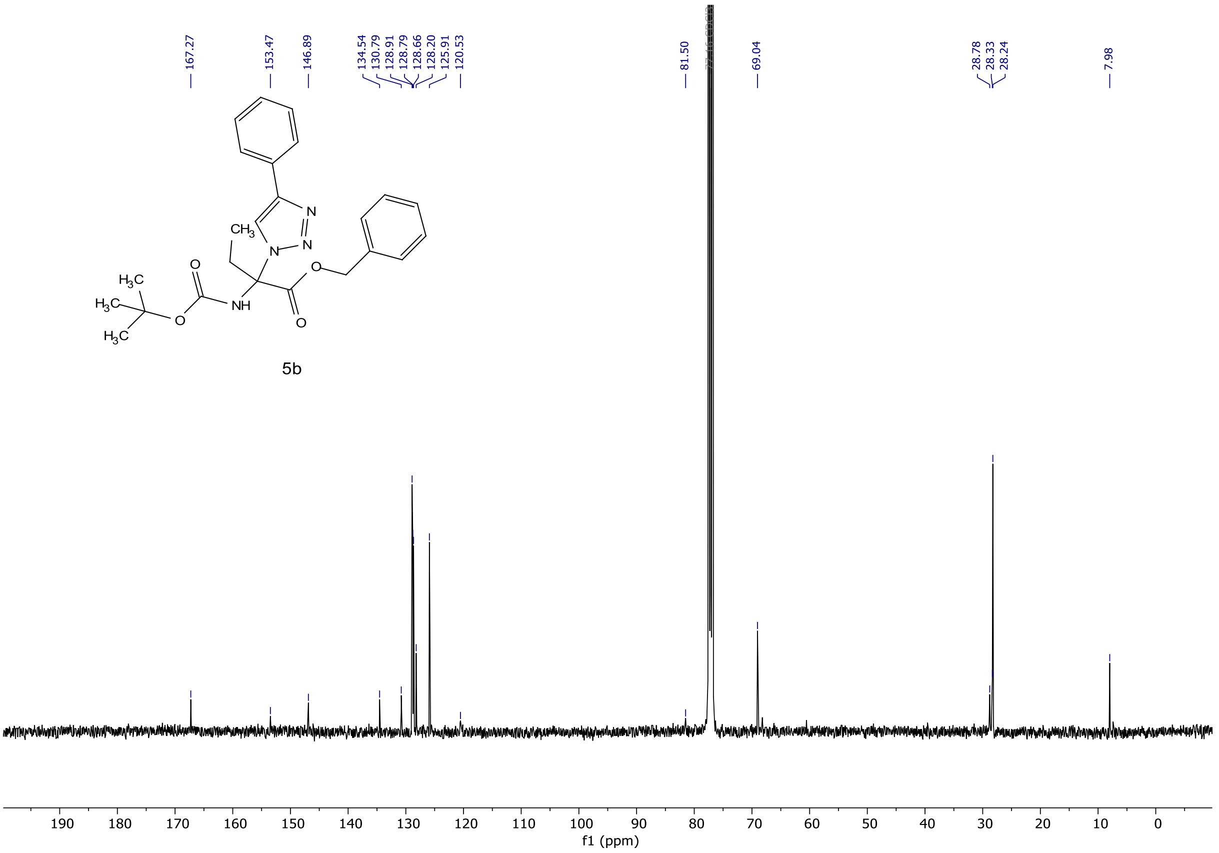

HMBC (400 MHz, $CDCl_3$) of compound **5b**

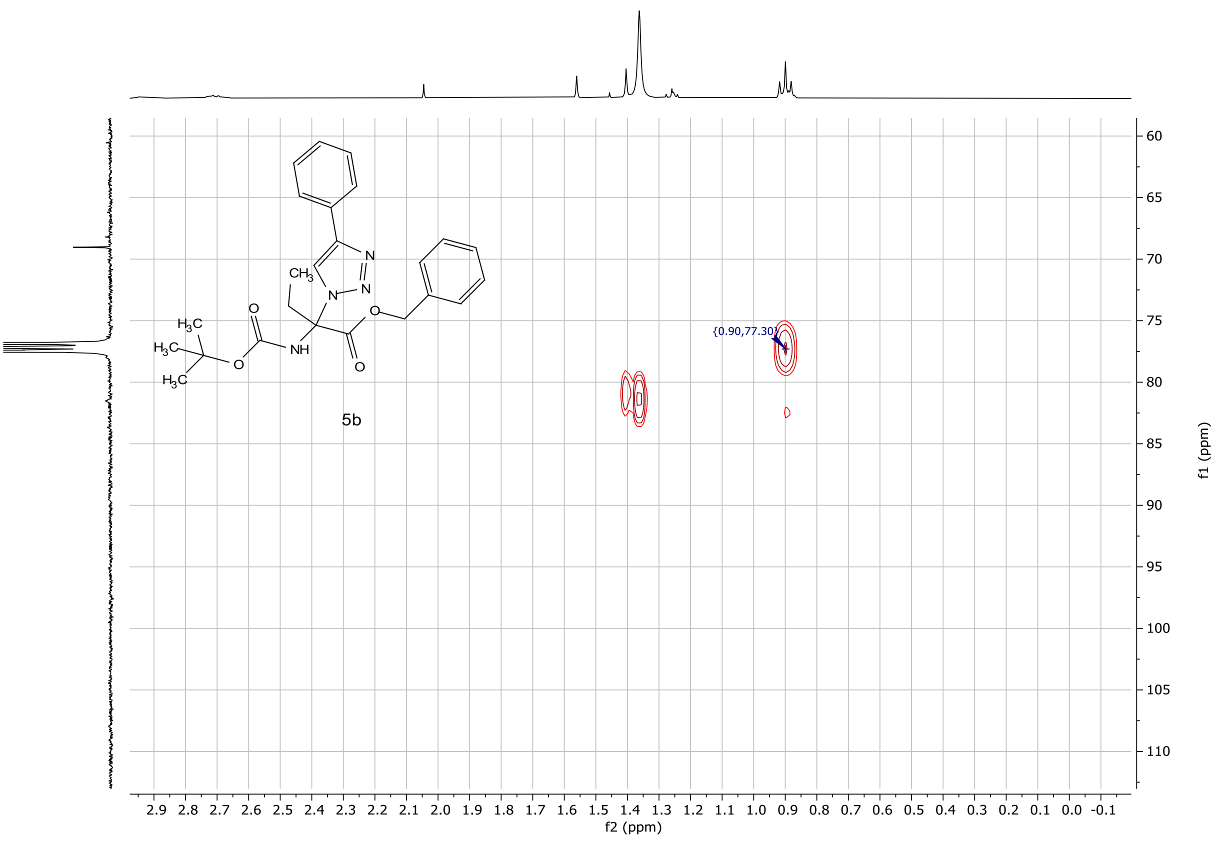

^{1}H NMR (400 MHz, $CDCl_3$) of compound **6**

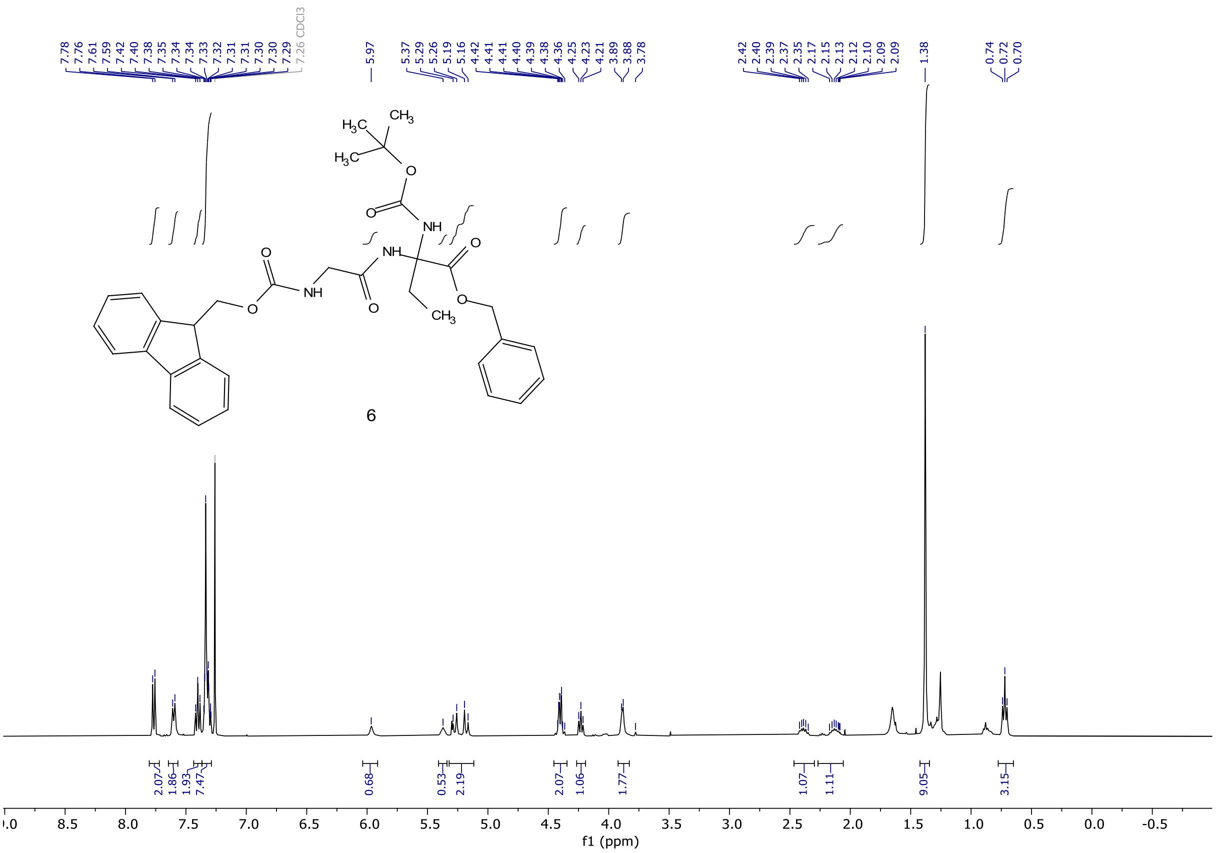

^{13}C NMR (400 MHz, $CDCl_3$) of compound **6**

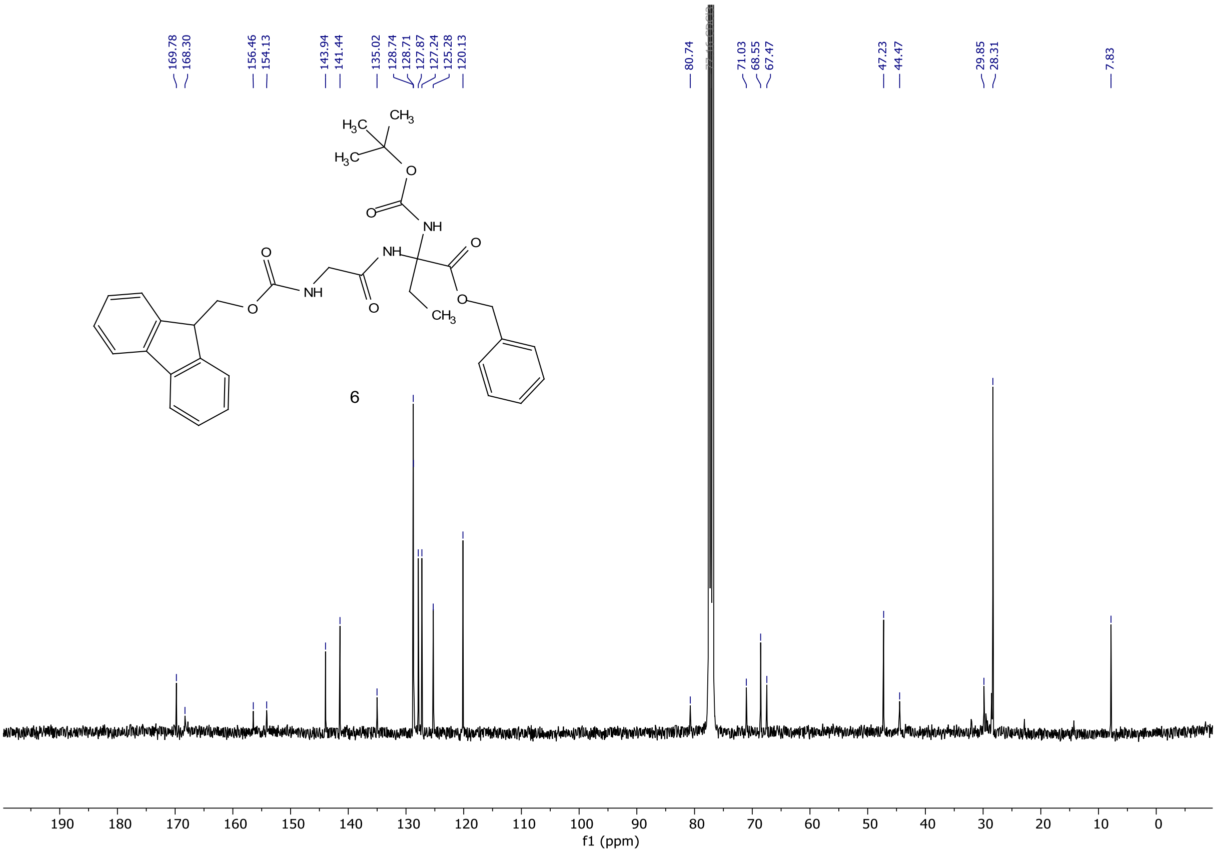

^{1}H NMR (400 MHz, $CDCl_3$) of compound **7**

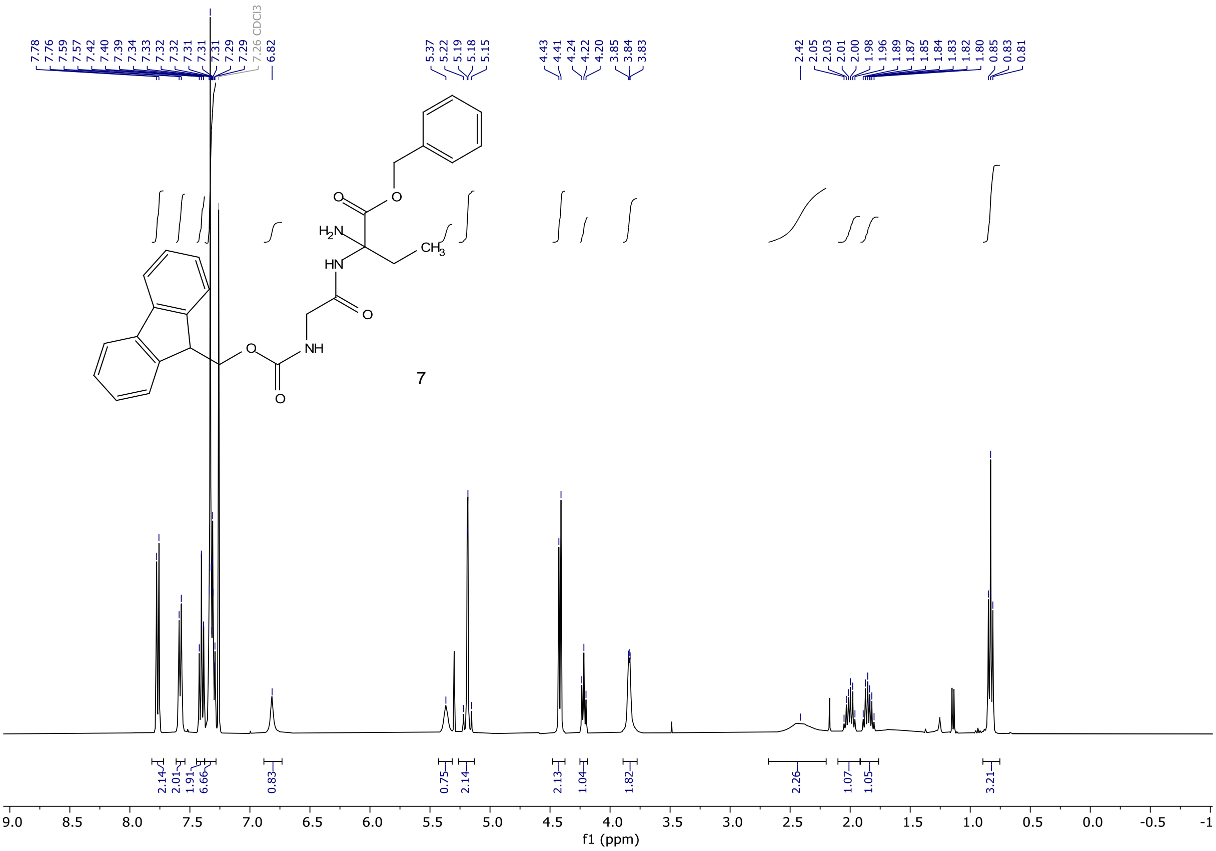

^{13}C NMR (400 MHz, $CDCl_3$) of compound **7**

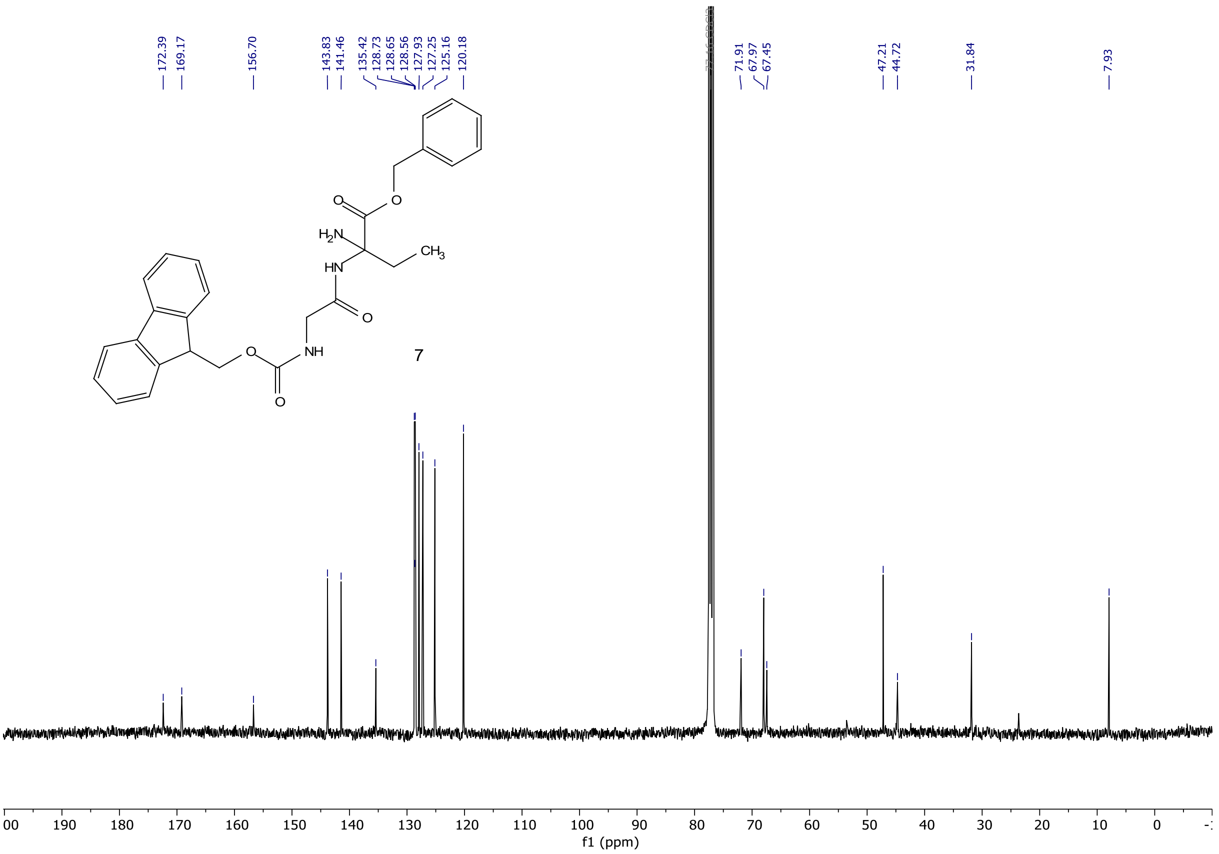

^{1}H NMR (400 MHz, $CDCl_3$) of compound **8**

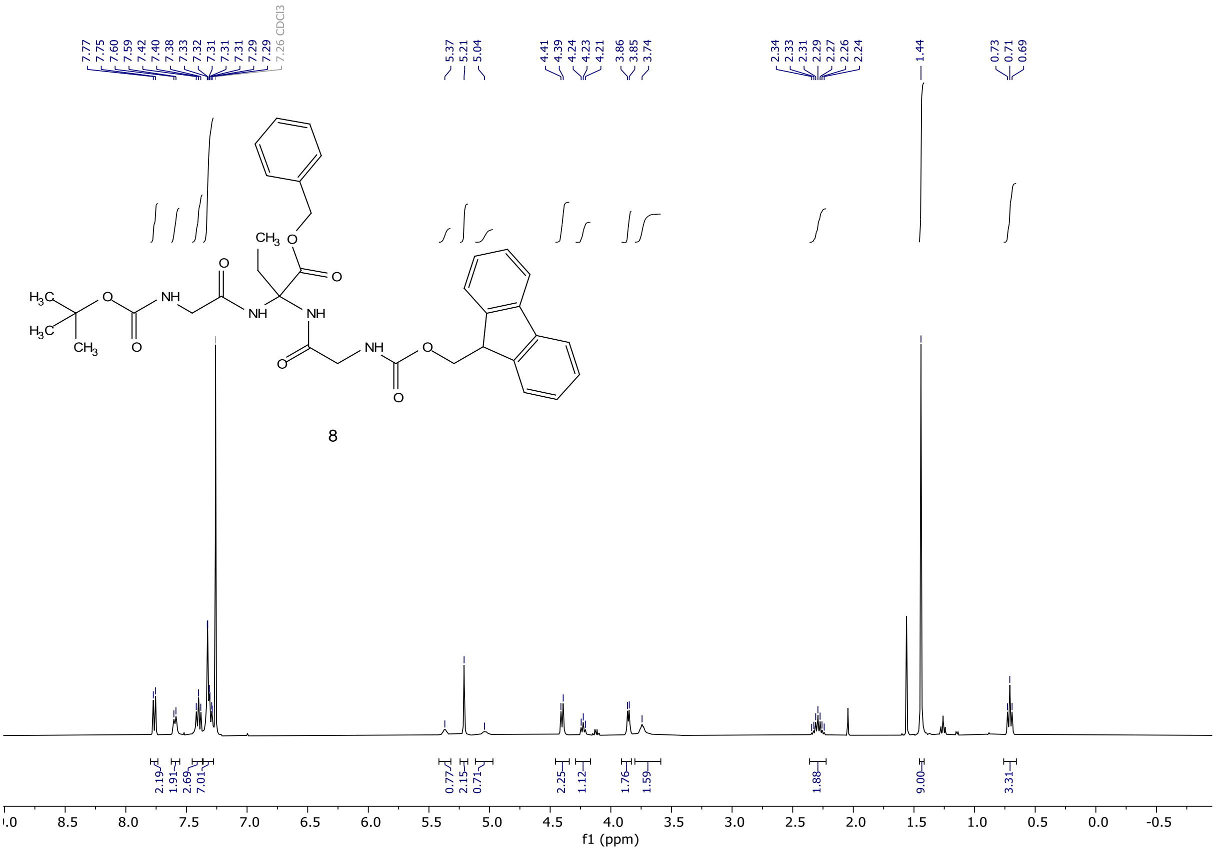

^{13}C NMR (400 MHz, $CDCl_3$) of compound **8**

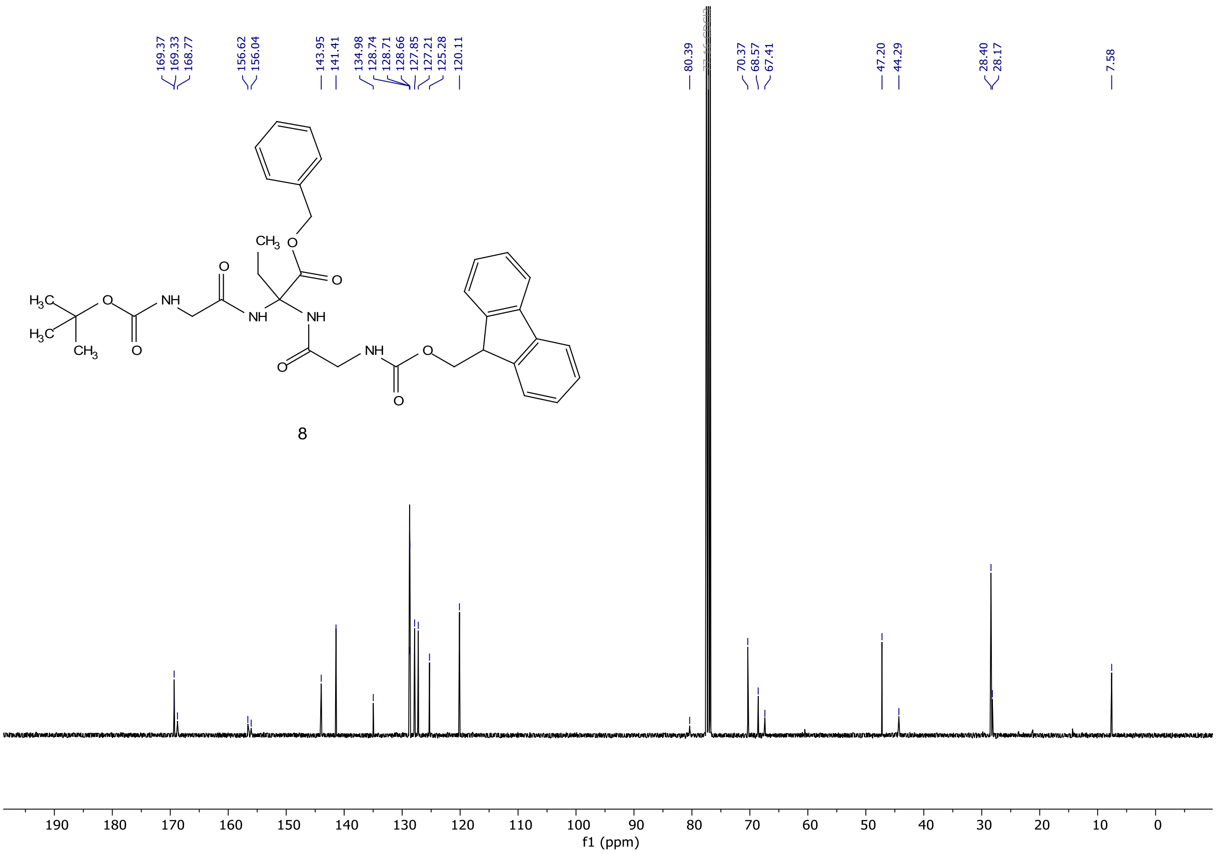

^{1}H NMR (400 MHz, $CDCl_3$) of compound **9**

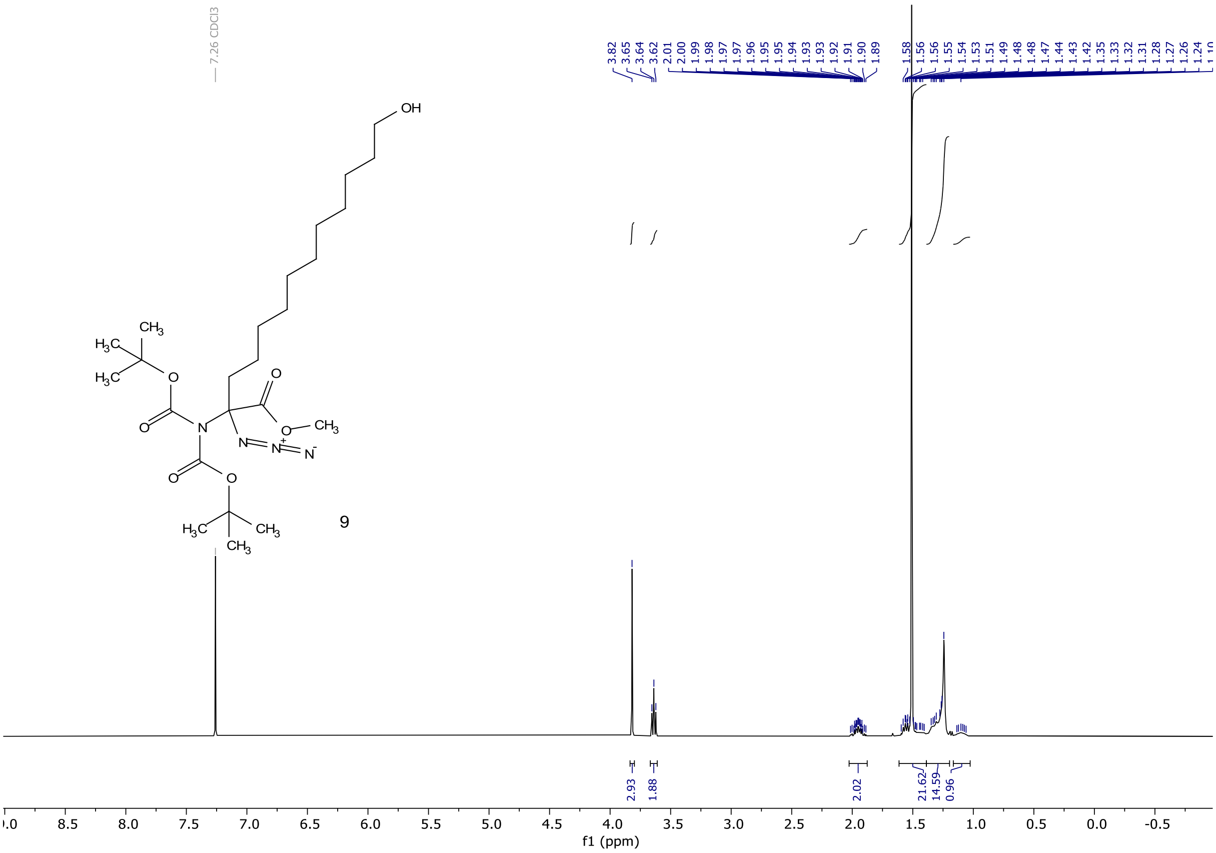

^{13}C NMR (400 MHz, $CDCl_3$) of compound **9**

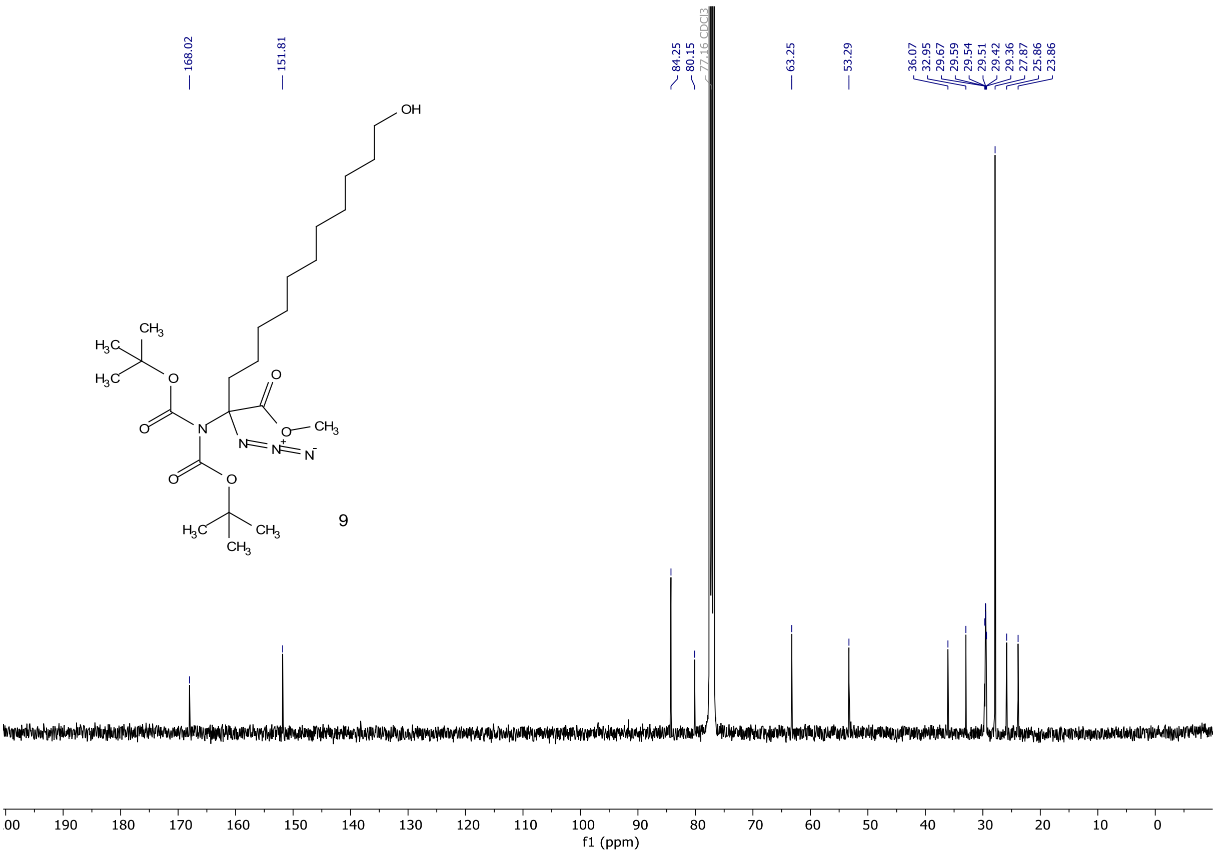

^{1}H NMR (400 MHz, $CDCl_3$) of compound **10**

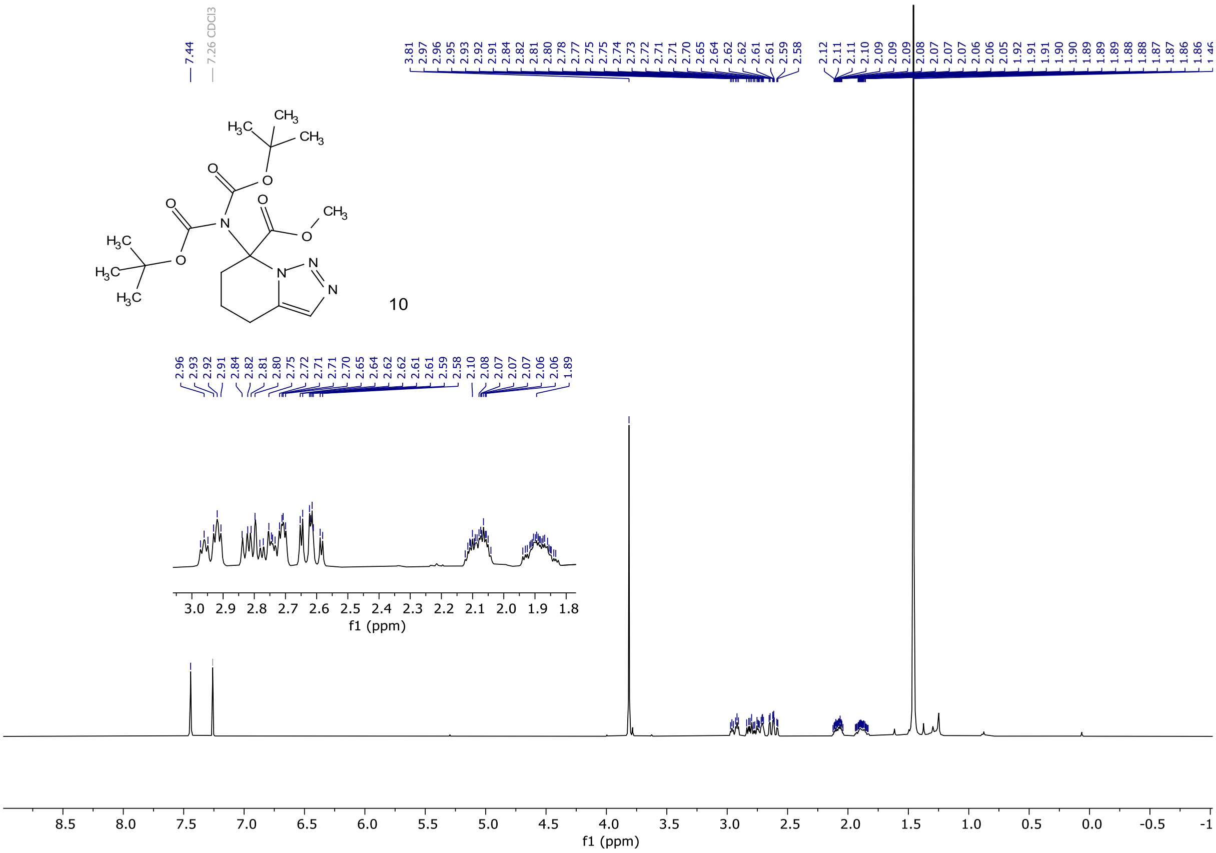

^{13}C NMR (400 MHz, $CDCl_3$) of compound **10**

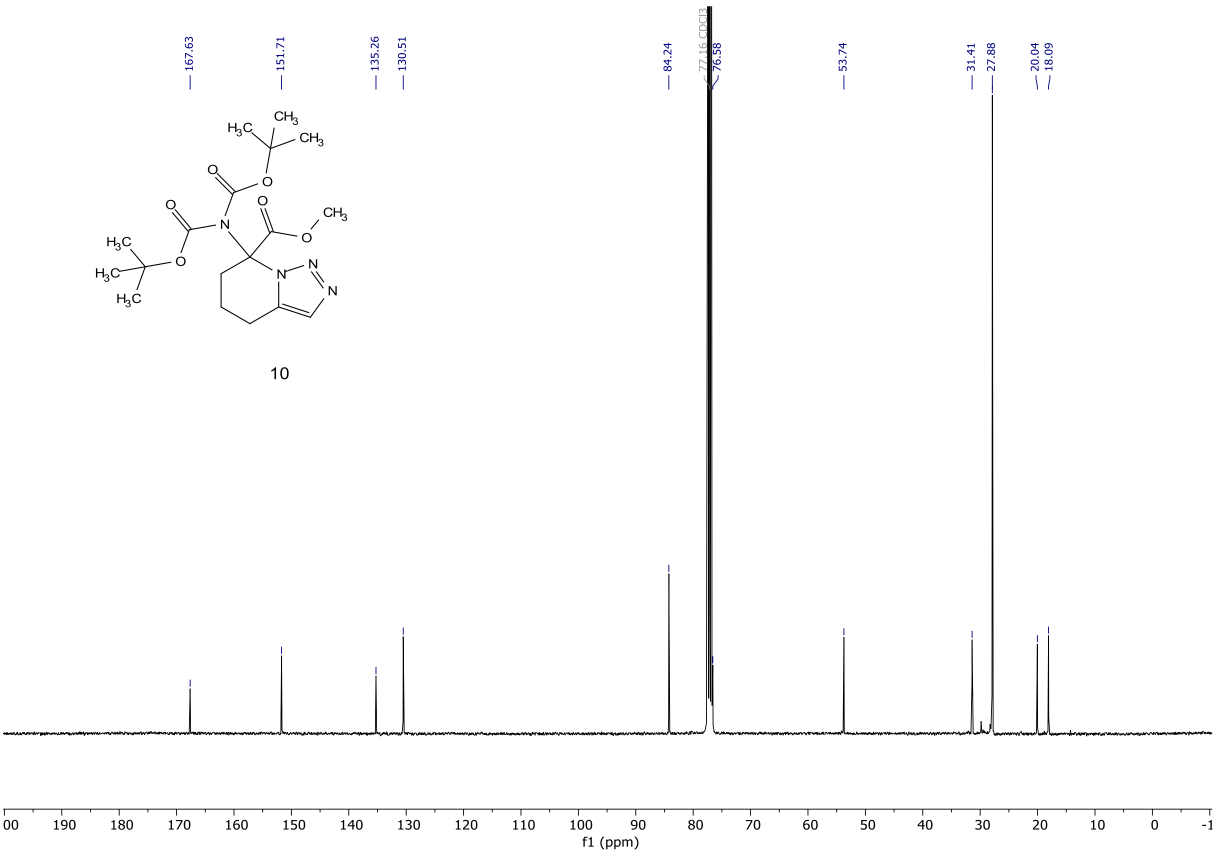

^{1}H NMR (400 MHz, $CDCl_3$) of compound **11**

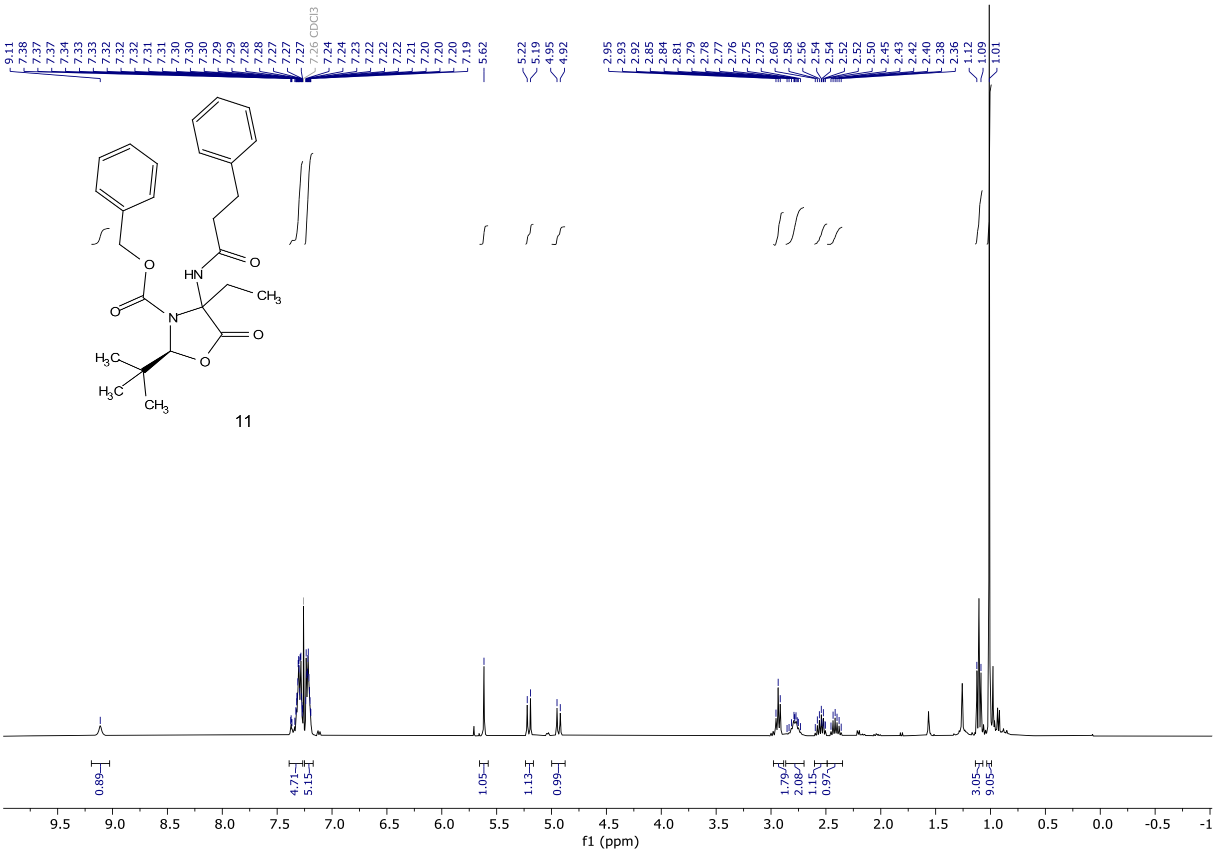

^{13}C NMR (400 MHz, $CDCl_3$) of compound **11**

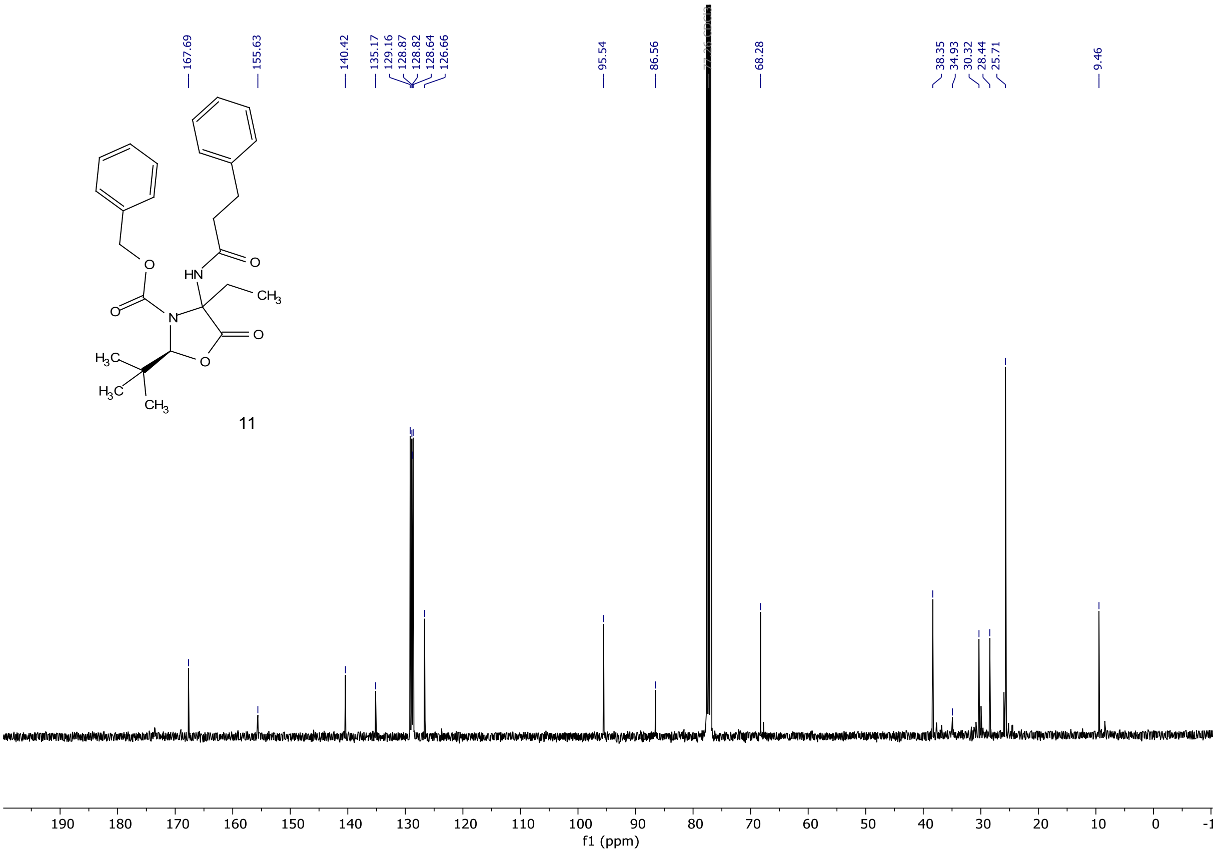

Supplement: Supplementary file 1 — ol3c02153_si_001.pdf [file ol3c02153_si_001.pdf]
